# A comparison of genomic profiles of complex diseases under different models

**DOI:** 10.1186/s12920-015-0157-2

**Published:** 2016-01-19

**Authors:** Víctor Potenciano, María Mar Abad-Grau, Antonio Alcina, Fuencisla Matesanz

**Affiliations:** 1 Departamento de Lenguajes y Sistemas Informáticos, ETSIIT, c/ Periodista Daniel Saucedo Aranda s/n Universidad de Granada, Granada, 18071 Spain; 2 Instituto de Parasitología y Biología Molecular, CSIC, Granada, Spain

## Abstract

**Background:**

Various approaches are being used to predict individual risk to polygenic diseases from data provided by genome-wide association studies. As there are substantial differences between the diseases investigated, the data sets used and the way they are tested, it is difficult to assess which models are more suitable for this task.

**Results:**

We compared different approaches for seven complex diseases provided by the Wellcome Trust Case Control Consortium (WTCCC) under a within-study validation approach. Risk models were inferred using a variety of learning machines and assumptions about the underlying genetic model, including a haplotype-based approach with different haplotype lengths and different thresholds in association levels to choose loci as part of the predictive model. In accordance with previous work, our results generally showed low accuracy considering disease heritability and population prevalence. However, the boosting algorithm returned a predictive area under the ROC curve (AUC) of 0.8805 for Type 1 diabetes (T1D) and 0.8087 for rheumatoid arthritis, both clearly over the AUC obtained by other approaches and over 0.75, which is the minimum required for a disease to be successfully tested on a sample at risk, which means that boosting is a promising approach. Its good performance seems to be related to its robustness to redundant data, as in the case of genome-wide data sets due to linkage disequilibrium.

**Conclusions:**

In view of our results, the boosting approach may be suitable for modeling individual predisposition to Type 1 diabetes and rheumatoid arthritis based on genome-wide data and should be considered for more in-depth research.

**Electronic supplementary material:**

The online version of this article (doi:10.1186/s12920-015-0157-2) contains supplementary material, which is available to authorized users.

## Background

Genome-wide association studies are being used to build multimarker predictive models of individual susceptibility to complex diseases, referred to as genomic profiling or genomic predictors of genetic risk. The most common approach is to use simple logistic regression on a genome-wide genetic risk score (GRS) counting down the number of risk variants an individual has [1–5] or on a weighted GRS (wGRS) [1–8] that uses the log odds ratio (OR) for the disease associated with each position to weigh the effect of each genetic variant on the disease outcome. Logistic regression with wGRS is equivalent to the simpler Bayesian network defined for classification, the naïve Bayes classifier, which has also been used for this purpose [9, 10]. Multiple logistic regression is also an option in genome-wide data [11, 12] if some strong restriction to the number of input variables considered is imposed for them to be computationally feasible.

The most common statistic used to measure the quality of a risk predictor is the C-statistic or the area under the receiver operating characteristic (ROC) curve (AUCROC or AUC), which measures how well it can distinguish prediction rates between the two diagnostic groups (in genomic profiling, disease risk prediction between diseased and normal individuals).

In polygenic diseases, successful assessment of risk prediction is not only a matter of the accuracy of method used but a question of genetic epidemiology (i.e. disease prevalence and heritability). Disease prevalence, *K*, is the proportion of diseased individuals in a population [13]. Heritability may be measured by *λ*_*s*_, the sibling risk ratio or ratio of the prevalence of disease in siblings of affected individuals compared to the prevalence in the population *K* [13]. A highly polygenic disease has a mild genetic component (i.e. modest heritability and high prevalence). The more polygenic a disease, the greater the number of associated variants and the smaller their effect on the disease. Given a value reported by a statistic of fitness, there is no way of knowing which part represents model fitness to the true genetic risk of individuals and which part represents disease epidemiology (i.e. how well the true genetic risk predicts disease status) [13]. In order to help differentiate these two components of the AUC statistic, the *AUC*_*max*_ measure should also be used. The *AUC*_*max*_ measures the genetic component (i.e. the maximum AUC possible for there to be the perfect predictor of disease risk) ([13]) so that it will be lower in highly polygenic diseases.

At times, statistics of fitness such as the AUC are not even reported for genomic predictors possibly because they are much lower than risk predictors built on phenotype and environmental data collected from medical tests or questionnaires. This is the case of studies on depression and anxiety [6]. In other cases, there is no or only a modest AUC improvement (of no more than 0.01) when a GRS is added to traditional risk factors, such as in the case of coronary artery disease (CAD) [5, 14], myocardial infarction in Hispanics [15] or atherosclerosis [16]. Even if there were an improvement of more than 0.01, this is much lower than it should be considering the genetic epidemiology of the disease. This is the case of predictors for rheumatoid arthritis (RA) [17] and lung cancer [4]. By way of example, in the predictor of lung cancer, the predictive AUC in a within-study validation approach using bootstrapping was 0.639 [4] while the *AUC*_*max*_ is 0.98, *AUC*_*half*_ (the AUC when only half the genomic variants are included) is 0.89 and *AUC*_*quar*_ (only a quarter of the variants are included) is 0.80 for lung cancer [13].

Most of these studies showed negative results as their predictive values were too low for them to be considered clinically useful when applied to a sample at risk [18]. Moreover, for highly polygenic diseases, their AUC scores were much lower than they should have been according to disease heritability and prevalence [13].

Additional file 1: Table S1 shows 6 of the 7 diseases from the WTCCC used in this study ordered according to their genetic component or polygenic level in terms of the *AUC*_*max*_ (column four). Prevalence and heritability are also shown (columns 3 and 4, respectively). These results were taken from Wray et al. 2010 [13]. The results shown for irritable bowel disease (IBD) were actually reported very similar disease, Crohn’s disease. There is no information about prevalence and heritability in hypertension (HT).

One interesting exception for a highly polygenic disease (*AUC*_*max*_=0.92) is a predictor of age-related macular degeneration built as a wGRS from only 13 risk variants, reporting an AUC of 0.84 [19], while the *AUC*_*max*_,*AUC*_*half*_ and *AUC*_*quar*_ [13] are 0.92,0.81 and 0.72, respectively. An independent validation data set was not used to confirm this result in order to detect possible AUC over-estimation because of a biased data set, possibly due to cases sharing other traits such as a higher average body mass index than the controls. However, even if the data set were not biased and the AUC had been correctly estimated, it should be noted that only individuals with both eyes affected and at least one having a severe form of age-related macular degeneration were selected. The reference max, half and quart AUCs do not therefore hold but much higher values may be obtained since the authors are considering a very aggressive type of age-related macular degeneration which may have a much higher heritability [20].

The opposite of a highly polygenic disease is one that is not very polygenic (shown in the last rows of Table 1), i.e. those with high heritability and low prevalence and therefore a high *AUC*_*max*_ [13]. Extreme examples of not very polygenic diseases are Type 1 diabetes, Crohn’s disease and systemic lupus erythematosus, which all have *AUC*_*max*_=1 [13]. Two issues in highly polygenic diseases, which have been identified as responsible either for the negative results or for over-estimating classifier performance [2, 3, 7, 15, 21–23], have been successfully handled in not very polygenic diseases.

**Table 1:** Model learned from the T1D data set. Model learned from the T1D data set using 1-e5 as the *p*-value threshold, the holdout approach, AdaBoostM1 as the learning algorithm with default configuration (decision stump as the weak learning algorithm and 10 iterations)

Chr	Chr	SNP	Allele	Allele	Weight	Genotypes	Weight	Genotypes
*#*	Pos		1	2	rule 1	rule 1	rule 2	rule 2
1	77051324	*SNP*_*A*−1827111	A	C	0.0801	{0, missing}		
6	32444658	*SNP*_*A*−1934589	A	G	0.2326	{0}		
6	30135583	*SNP*_*A*−2111335	A	G	0.2171	{0}	0.0543	{1}
6	31395153	*SNP*_*A*−2079423	C	T	0.1525	{0,1}		
6	30390814	*SNP*_*A*−2222387	A	G	0.0672	{0,1}		
6	31112694	*SNP*_*A*−4293786	C	T	0.0517	{0,1}		
19	39266932	*SNP*_*A*−4281637	A	G	0.0904	{0,1}		

Weights and genotypes values are referred to class 1, i.e. absence of disease. Chromosome positions correspond to assembly NCBI dbSNP GRCh38.p2

The first issue relates to the use of prior information from GWAS with low power. Low accuracy was therefore due to the limited number of susceptibility variants detected in previous studies [18], which were used as the only input variables to build the predictor. In order to solve this, many of them selected markers on the basis of predefined thresholds for the *p*-values associated with the disease in the training data set [2, 18]. This approach was expected to perform better and in fact it did for the unpolygenic disease Type 1 diabetes when the algorithms used to model the classifier were robust to redundant or noisy variables, since variables associated with the disease in a real yet low-level way might well be detected through this approach [18].

The second issue, which relates to the testing procedure, consists in using a discovery data set and a validation data set with various individuals in common [3, 7, 21–23], and has been identified as a cause of over-estimated accuracy and considered “cheating” [11]. A similar problem was specifically noticed under the wGRS approach, for which log ORs used as weights were learned from a GWAS with certain samples also included in the validation data set [2, 15] or under any other approach in which the external GWAS used to select single nucleotide polymorphisms (SNPs) shared some samples with the validation data set [1, 24]. Three alternatives to wGRS with weights estimated from other GWAS have been considered: the first is to learn the OR for each marker from the discovery data set, which is equivalent to using the naïve Bayes classifier [25]; the second is to use multimarker logistic regression, which may result in negative results due to overfitting when the power of the study is low [24, 26]; and the third is to use other approaches which are more robust to redundant or noisy variables. The third alternative was somehow successful in a study conducted on Type 1 diabetes [18], as the AUC score in a within-study 5-fold cross-validation approach was 0.89. It should be noted that the *AUC*_*max*_ is 1, *AUC*_*half*_ is 0.93 and *AUC*_*quar*_ is 0.84 for Type 1 diabetes [13]. However, looking in depth at the way the work was conducted, it seems that they added a set of 45 known susceptibility markers for Type 1 diabetes to the prediction model and some of these markers were obtained by a pooling study using, among other things, the analyzed data set [27].

As previously mentioned, however, the current challenge is to handle these issues in highly polygenic diseases. To the best of our knowledge, no sound genetic predictor with a predictive AUC near its expected value [13] in terms of the genetic disease epidemiology has yet been built for any highly polygenic disease.

One possible reason for these negative results relates to the use of too simplistic models that assume marker independence and which may be unable to control redundant or noisy variables. However, there has been no success when predictive models capable of handling marker interactions and variable redundancy have been built under different approaches as a way to improve the predictive capacity, such as support vector machines [18], decision trees, random forests and boosting algorithms [28]. By way of example, in multiple sclerosis (MS), the AUC did not increase when other algorithms building more complex models (e.g. the Tree Augmented naïve Bayes classifier or a random forest) were used instead of a naïve Bayes classifier [28].

To the best of our knowledge, no systematic study with the purpose of building predictors for complex diseases of different epidemiological patterns, under different statistical approaches which is able to represent marker interactions and/or handle redundant or noisy variables, has yet been conducted. We are therefore unable to conclude whether more complex models are the key to turning the current discouraging results into positive ones.

Perhaps, the lack of positive results may be due to a bias in most of the models used so far to represent marker dependencies and the implicit assumptions they rely on should be explored. Consequently, most of the attempts tried so far ignored chromosomal information. One result supporting this hypothesis was undertaken for Crohn’s disease [24] by a haplotype-based predictor. Although the predictive AUC was still too low (0.72) to be considered a positive result (*AUC*_*max*_,*AUC*_*half*_ and *AUC*_*min*_ are 1,0.95 and 0.86 [13], respectively), it was much higher than when haplotype information was ignored (0.655). The authors only used haplotypes of 2-SNP length. There is still the question of whether the AUC would have improved if longer haplotypes had been used. As previously mentioned [24], long haplotypes in case/control data sets may not be a solution as they are inferred with an important lack of accuracy. However, small haplotypes such as those of 3, 4 or 5 SNP length were not tried either. The authors also noted that signal dilution is less severe in shorter haplotypes. However, certain procedures for avoiding signal dilution could be used [29].

Additional file 1: Table S2 shows a summary of the most important studies mentioned. In light of these discouraging results, our objective in this work was to answer the question of whether it is possible to build genomic risk predictors from case/control GWAS. They must therefore be capable of reaching the expected AUC at least in a within-study validation approach, considering disease heritability and population prevalence. Disease heritability can be defined as the proportion of phenotypic variance that is genetic whereas population prevalence can be defined as the marker density of the array used for genotyping and sample size [13, 30]. These last two features affect the efficacy of the genetic profiles, i.e. the proportion of genetic variance that they can explain.

With this purpose, we performed a comparative study of the predictive capacity of disease predictors built with learning algorithms under different statistical approaches for 7 different diseases with different levels of the genetic component (Table 1). We also used a haplotype-based approach with haplotypes of different lengths from 1 to 5 in order to understand the importance of using chromosomal information.

## Results

### Genotype-based predictors

We applied the common genotype-based model on a wide variety of approaches ranging from the most simple (e.g. simple logistic regressions built on weighted or unweighted genetic risk scores and naïve Bayes classifiers) to the more complex (e.g. support vector machines or random forests which are capable of modeling variable interaction and handling redundant or noisy variables). Although multiple logistic regression may also deal with redundant or noisy variables, given their highly time-consuming processes of model building, they are not time-feasible without imposing some limit on the number of input variables used in genome-wide datasets. As the next subsection outlines, this limitation may lead to worse predictive models than machine-learning approaches which are able to handle hundreds of thousands of variables more efficiently.

**Fig. 1:**
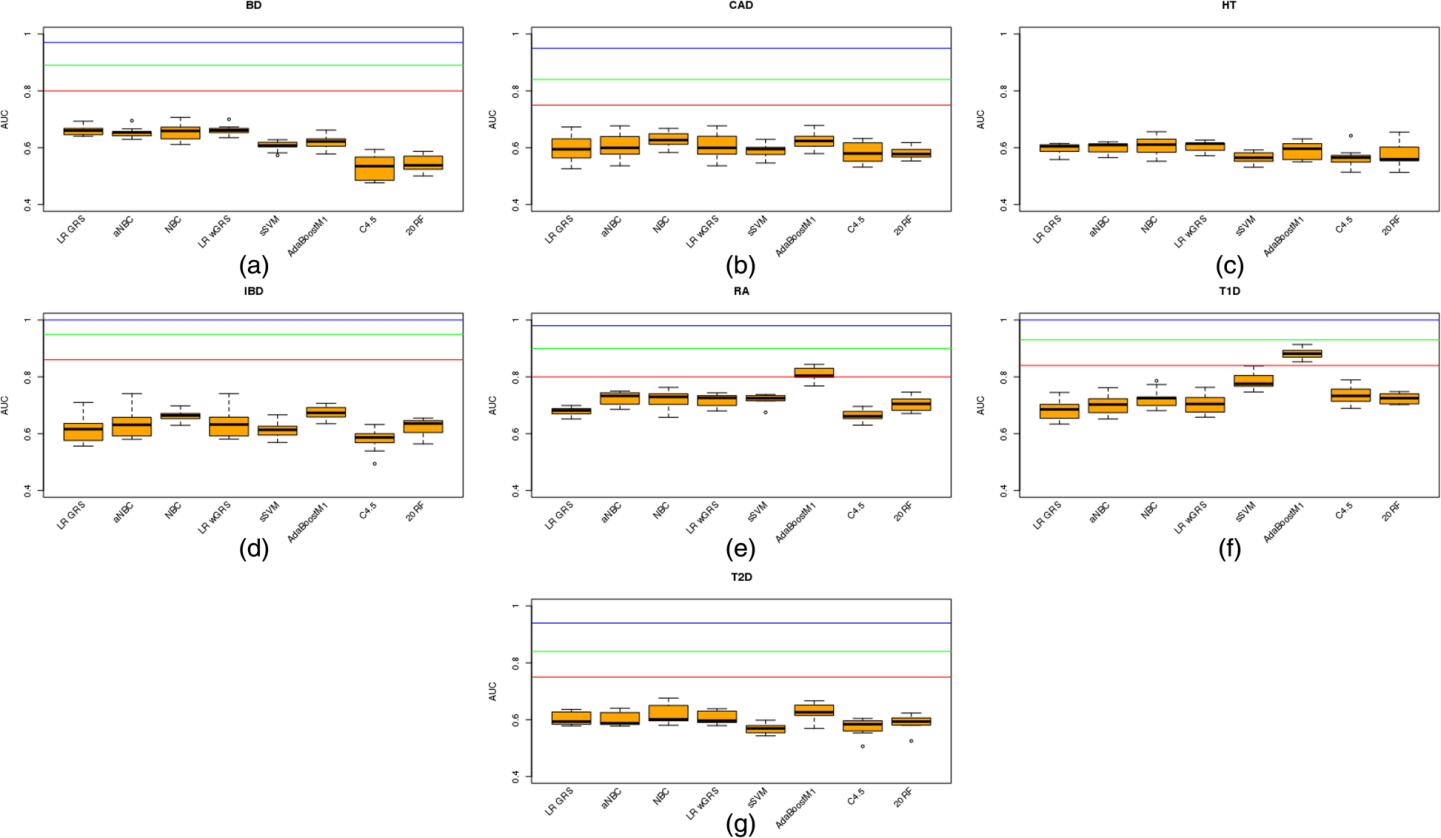
Boxplots for predictive AUC of genotype models. Boxplots for the predictive AUC obtained for each fold in the 10-fold cross-validation (cv) approach used to learn genotype models from data under different algorithms: **a** Results for bipolar disorder (BD), **b** coronary artery disease (CAD), **c** hypertension (HT), **d** irritable bowel disease (IBD), **e** rheumatoid arthritis (RA), **f** Type 1 diabetes (T1D) and **g** Type 2 diabetes (T2D). For each plot (with the exception of hypertension where data was unknown), three horizontal lines are also plotted corresponding to *AUC*_*quar*_ (red line), *AUC*_*half*_ (green line) and *AUC*_*max*_ (blue line) [13]

Figure 1(a)–(g) show the AUC values for each of the 7 diseases used in this work. Each figure compares results using logistic regression on a GRS (LR GRS), logistic regression on a wGRS (LR wGRS), a naïve Bayes classifier (NBC), an allelic naïve Bayes classifier assuming the two allelic variables for each position are identically distributed and conditionally independent given the trait (aNBC) [31], a sigmoid-based support vector machine (sSVM), a boosting algorithm (AdaBoostM1), a decision tree learning algorithm (*c*4.5) and 20 a random forest learning algorithm (20RF) (see Methods for a short description of sSVM, AdaBoostM1, C4.5 and 20RF).

With the exception of the hypertension plot for which we do not have the necessary data, all the plots show three horizontal lines with the expected AUC when all, half and a quarter, respectively, of the known genetic variance is explained by the variants included in the model, as published in [13]. It can be observed how the predictive AUC reached at least *AUC*_*quar*_ for only two diseases (Type 1 diabetes and rheumatoid arthritis). The genetic component of these two diseases is high (*AUC*_*max*_ is 1 and 0.98, respectively). However, the predictive AUC for irritable bowel disease, an extremely unpolygenic disease, which every algorithm obtained, is far lower than *AUC*_*quar*_.

Focusing on the algorithm used, the winning algorithm for these two diseases is AdaBoostM1. AdaBoostM1 is actually the only algorithm that outperforms *AUC*_*quar*_ in two diseases: rheumatoid arthritis (the mean predictive AUC is 0.8087, far higher than 0.7248, the second highest predictive AUC, obtained by allelic NBC) and Type 1 diabetes (the mean predictive AUC is 0.8805, far higher than 0.7849, the second highest predictive AUC, obtained by sSVM, another complex algorithm).

Therefore, for Type 1 diabetes, two algorithms (AdaBoostM1 and sSVM) obtained a predictive AUC of over 0.75, the threshold required for a risk classifier to be clinically useful when applied to a sample at increased risk [32]. However, AUC superiority of AdaBoostM1 was statistically significant (*α* level 0.01, *p*-value was 0.00256 in a Wilcoxon Signed-Rank computed on the 10 folds). For rheumatoid arthritis only AdaBoostM1 managed to build a clinically useful disease predictor. For every other disease, no algorithm would be clinically useful for a sample at risk, even less so for application as a diagnostic test in the general population. It should be noted that in order for it to be useful for the general population, the predictive AUC has been estimated at over 0.99 [32]. The negative results obtained by LR GRS and LR wGRS concur with previously published ones using the same data sets [2].

Freund and Schapire (1996) [33] present a brief example of the models that AdaBoostM1 can learn using the default configuration of decision stumps (i.e. one-level decision trees or 1-rules) as the weak learning algorithm to explain what iterations and weak learning algorithms mean in the AdaBoostM1 algorithm. We subsequently used a holdout approach whereby the original data set was divided into two independent data subsets of equal size: the training data subset used to learn the model and the test data subset used to compute the predictive AUC. We also only chose 10 iterations, which will build models with 10 SNPs at most. The predictive AUC was 0.822, which as expected was lower than the one obtained under the cross-validation approach with 2500 iterations, but still higher than 0.75. The model actually contains 7 different SNPs and is shown in Table 1. Let us try to understand what the model means and how it can be applied to infer the risk of a certain individual having Type 1 diabetes. Class 1 means not having the disease. Table 1 shows the genotype values that increase the probability *p* of being healthy or equally decrease the risk of having the disease. In order to compute *p* for a given individual, the model simply adds the increase in *p* (weights) conferred by each SNP associated with the disease. For each SNP, Table 1 shows the chromosome number (column 1), the chromosome position (column 2) under assembly GRCh38, SNP ID (column 3), allele 1 (column 4), allele 2 (column 5), the increase in *p* (weight) added by the SNP for the genotype in column 6 and in the case of a second genotype configuration increasing *p* in a different way, columns 7 and 8 show the weight for the second configuration and the genotype itself, respectively. By way of example, an individual with the following genotypes 120?021 (where ? means a missing genotype), 0 no copies of allele 1 (i.e. homozygous for allele 2), 1 heterozygous and 2 homozygous for allele 1, will have *p*=0+0+0.2170542636+0+0+0+0.0904392765=0.3074935401 of being healthy or 1−*p*=0.6925064599 probability of having Type 1 diabetes, as the individual only has two genotypes for protection against the disease: *SNP*_*A*_−2111335 at chromosome 6 and *SNP*_*A*_−428163 at chromosome 19 (see columns 6 and 8 for genotype values associated with class 1), thereby increasing the probability of being healthy.

What is interesting from this approach is that this reduced model, which is learned from a training data set of only 1722 individuals, obtained a predictive AUC of 0.822 with only 7 SNPs, still higher than the second best result of 0.8112 obtained by 20RF (see Additional file 1: Table S2) under the same holdout approach. It should be noted that the classic models LR GRS and LR wGRS achieved much lower values: 0.7126 and 0.7294, respectively.

As mentioned previously, however, the more sophisticated model learning approaches (e.g. boosting algorithms, SVM or random forest classifiers) were only clinically useful when applied to a sample at risk in Type 1 diabetes and rheumatoid arthritis.

In order to study model reproducibility, we switched training and test data sets and ran AdaBoostM1 again under the same default configuration. The new model contains 9 different SNPs and is shown in Table 2.

**Table 2:** Study of model reproducibility: model learned from the T1D data set by switching training and test data subsets. Model learned from the T1D data set, with the same configuration as the model in Table 5 but switching training and test data subsets

Chr	Chr	SNP	Allele	Allele	Weight	Genotypes	Weight	Genotypes
*#*	Pos		1	2	rule 1	rule 1	rule 2	rule 2
6	32444658	*SNP*_*A*−1934589	A	G	1.09	{0}	0.22	{1}
6	30135583	*SNP*_*A*−2111335	A	G	0.74	{0}		
6	32444815	*SNP*_*A*−4303523	A	G	0.62	{0, 1}		
6	30726039	*SNP*_*A*−2240847	A	G	0.3	{0, 1, missing}		
19	39266932	*SNP*_*A*−4281637	A	G	0.41	{0,1}		
6	31112694	*SNP*_*A*−4293786	C	T	0.29	{0,1}		
6	31277959	*SNP*_*A*−1863445	A	G	0.31	{0,1}		
6	31278044	*SNP*_*A*−1949560	A	G	0.29	{0,1}		
1	113630788	*SNP*_*A*−2235405	A	G	0.2	{0}		

Weights and genotypes values are referred to as class 1, i.e. absence of disease

In the two models, all SNPs at chromosome 6 belong to the MHC region (chromosome positions from 28510120 to 33480577 under assembly GRCh38.p2).

Table 3 shows the AUC results under the genotype-based approach with 10-fold cross validation (more detailed results are displayed in the Additional file 1: Tables S2 and S5 and other measures of fitness in the Additional file 1: Table S6–S8).

**Table 3:** Highest AUC obtained by the genotype-based approach under the 10-fold cross validation sampling model

Disease	Median AUC	Min AUC	Max AUC	Learning machine	*p*-val threshold
BD	0.6619	0.635	0.7	LR wGRS	1e-2
CAD	0.6293	0.583	0.668	NBC	1e-4
HT	0.6039	0.572	0.627	LR wGRS	1e-2
IBD	0.6732	0.635	0.707	AdaBoostM1	1e-5
RA	0.8087	0.768	0.844	AdaBoostM1	1e-5
T1D	0.8806	0.853	0.914	AdaBoostM1	1e-5
T2D	0.6257	0.569	0.666	AdaBoostM1	1e-5

For comparative purposes with the haplotype-based approach that will be explained below, we have repeated the genotype-based model by using the same sampling approach used by the haplotype-based one: the holdout sampling. Otherwise, the AUC results for the haplotype-based models compared with the genotype-based models could be underestimated since only half the samples (holdout testing approach) were used as the training test while the original genotype-based models used 9/10 of the samples (10-fold cross validation testing approach).

The first four columns of Table 5 show the AUC results under the genotype-based approach with holdout validation. Detailed results can be found in Additional file 1: Tables S3 and S10 and other measures of fitness in Additional file 1: Tables S11–S13.

### Variable selection and multiple logistic regression

We wanted to compare these AUC results with some state-of-the-art regression models, taking into account the fact that for them to be applied in genome-wide data sets we would need to impose some limit to the maximum number of input variables so that they became computationally affordable.

Table 4 shows the AUC under the holdout approach obtained by the different algorithms using only the top 100 SNPs (lowest *p*-value) selected from the training dataset to avoid cheating [11]. We used 100 variables because this was the number of SNPs that achieved the best results in Type 1 diabetes and Crohn’s disease in a similar study [11]. Several multiple logistic regression methods such as penalized regression methods (ridge regression –RR– [34] and the lasso [35]) and stepwise fitting of GLM with AIC to select variables (GLM AIC were used.

**Table 4:** AUC under the holdout approach and different genotype-based predictors learned using only the 100 top SNPs

Disease	NBC	sSVM	AdaBoostM1	C4.5	20RF	lasso	RR	LR GRS	LR wGRS	GLM AIC
BD	0.53	0.551	0.552	0.524	0.535	0.552	**0.553**	0.523	0.534	0.551
CAD	0.573	**0.582**	0.56	0.532	0.574	0.566	0.569	0.556	0.568	0.567
HT	0.541	0.543	0.545	0.538	0.539	**0.559**	0.548	0.527	0.535	0.553
IBD	0.568	0.574	0.587	0.522	0.557	0.585	**0.587**	0.56	0.569	0.577
RA	0.651	0.715	0.734	0.694	0.689	0.725	0.73	0.614	0.638	**0.736**
T1D	0.675	0.79	0.777	0.747	0.771	0.777	0.79	0.669	0.686	**0.793**
T2D	0.566	0.569	0.565	0.541	0.566	0.575	0.571	0.566	**0.576**	0.556

The highest AUC for each disease is shown in boldface

Column 5 of Table 5 shows the best AUC under the holdout approach when only the top 100 SNPs are used. The method that achieved this highest AUC is shown in column 6. It can be seen how the AUC was always below that obtained when the number of variables was not limited (column 2).

**Table 5:** Highest AUC obtained by the genotype and the haplotype-based approaches

	Genotype-based, holdout approach					Haplotype-based, holdout approach			
	*p*-value filtering			Top 100 SNPs					
Disease	AUC	Learning	*p*-value	AUC	Learning	AUC	Learning	Haplotype	Threshold
		machine	threshold		machine		machine	length	*p*-value
BD	0.6222	LR wGRS	15e-2	0.553	RR	0.6873	AdaBoostM1-add.	3	1e-4
CAD	0.611	20RF	1e-5	0.582	sSVM	0.5761	20RF-rec.	3	1e-7
HT	0.5776	AdaBoostM1	15e-2	0.559	lasso	0.5573	NBC-all	5	1e-5
IBD	0.6136	AdaBoostM1	1e-5	0.587	RR	0.6213	AdaBoostM1-rec.	2	1e-5
RA	0.8152	AdaBoostM1	1e-5	0.736	GLM AIC	0.8024	AdaBoostM1-add.	2	1e-5
T1D	0.8615	AdaBoostM1	1e-5	0.793	GLM AIC	0.8682	AdaBoostM1-add.	3	1e-6
T2D	0.6134	AdaBoostM1	1e-3	0.576	LR wGRS	0.6372	AdaBoostM1-add.	2	1e-4

The highest AUC was obtained by the genotype (column 2) and the haplotype-based approaches (column 7) using the same multisampling method for both approaches: holdout. The learning machines used for the haplotype-based approaches include the genetic model used: additive (add.), recessive (rec.), dominant (dom.) or each model returns the same result (all.). Column 5 shows the highest AUC for the genotype approach when the number of input variables is reduced to the top 100 SNPs in order to use the time-consuming generalized linear models

### A haplotype-based approach

In light of the discouraging results when using genotype-based risk predictors in most of the diseases analyzed, even when more sophisticated algorithms were tested, we tried to enhance the information provided to a learning machine to build the risk predictor by keeping as much chromosomal (allelic) association as possible. With this goal, in a second step we built several haplotype-based models as explained in Methods.

**Fig. 2:**
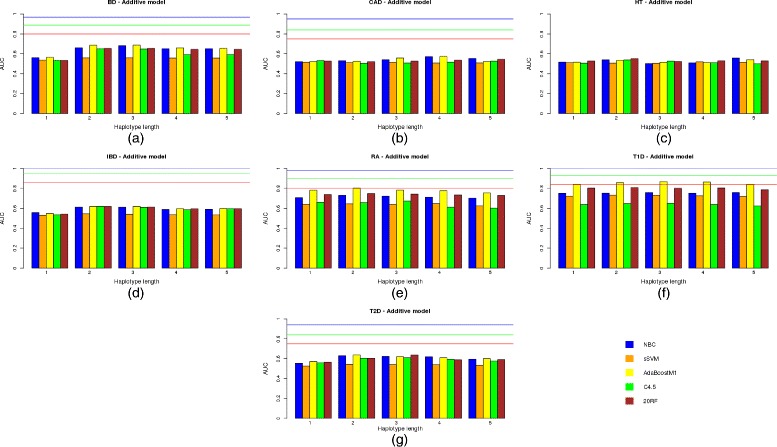
Predictive capacity of the haplotype-based approach under an additive genetic model. Predictive capacity of the haplotype-based approach under an additive genetic model: the AUC in the test data set is shown for different learning machines and the seven diseases of bipolar disorder (**a**), coronary artery disease (**b**), hypertension (**c**), irritable bowel disease (**d**), rheumatoid arthritis (**e**), Type 1 diabetes (**f**) and Type 2 diabetes (**g**)

Figure 2(a)–(g) show bar plots with the AUC values corresponding to the test data set for the 7 diseases and all of the different haplotype lengths used from 1 to 5 and all the learning machines built (i.e. naïve Bayes classifier (NBC), sSVM, AdaBoostM1, C4.5 and 20RF) under an additive genetic model.

**Fig. 3:**
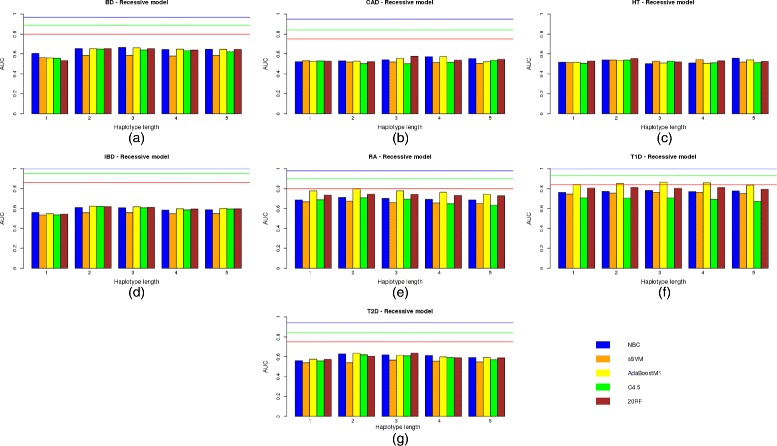
Predictive capacity of the haplotype-based approach under a dominant genetic model. Predictive capacity of the haplotype-based approach under a dominant genetic model: the AUC in the test data set is shown for different learning machines and the seven diseases of bipolar disorder (**a**), coronary artery disease (**b**), hypertension (**c**), irritable bowel disease (**d**), rheumatoid arthritis (**e**), Type 1 diabetes (**f**) and Type 2 diabetes (**g**)

**Fig. 4:**
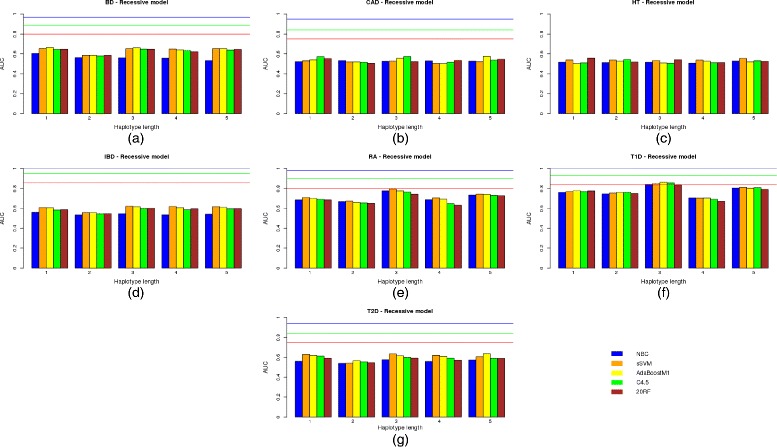
Predictive capacity of the haplotype-based approach under a recessive genetic model. Predictive capacity of the haplotype-based approach under a recessive genetic model: the AUC in the test data set is shown for different learning machines and the seven diseases of bipolar disorder (**a**), coronary artery disease (**b**), hypertension (**c**), irritable bowel disease (**d**), rheumatoid arthritis (**e**), Type 1 diabetes (**f**) and Type 2 diabetes (**g**)

The same results but for a dominant and recessive genetic model are shown in Figs. 3(a)–(g) and 4(a)–(g), respectively.

Regarding the haplotype length, it is interesting to note that the approach based on haplotypes comprising only two SNP positions [24] has been improved by a more general approach in which different numbers of SNPs, i.e. haplotype lengths, were tested. Although the accuracy of inferred haplotypes increased with the number of markers used, a modest number of SNPs of between 2 and 3 seem to obtain the best trade-off between haplotype accuracy and model fitting. For HT, the best solution (AUC 0.5573) was obtained by all the genetic models used with NBC and haplotypes comprising 5 SNPs. However, it should be noted that the AUC is too low and is outperformed by the genotype-based model. Therefore, haplotypes which are greater than 3 do not return better results. As already mentioned [24], this may be due to the decrease in accuracy of the reconstructed haplotypes.

Table 5 shows the best predictive AUC under the genotype-based approach (columns 2 and 5) and the haplotype-based approaches (column 7). More detailed results can be seen in Additional file 1: Table S4 and S15–S29 and other measures of fitness in Additional file 1: Tables S30–S74.

Although multiple logistic regression seems to outperform modern classifiers such as sSVM or AdaBoost in most diseases under the same conditions, they are clearly below the AUC achieved when all the SNPs were used. By comparing results with no variable filtering except *p*-value thresholds, and according to our results, AdaBoostM1 seemed to be the best among all the learning approaches used. It therefore outperformed all the others in five out of the seven diseases for both the genotype-based and haplotype-based models. Regarding the genetic model used, the additive approach outperformed or equaled all the others in five out of the seven diseases, although this result was not statistically significant (*p*-value is 0.4086 in a paired Student t-test on the null hypothesis of no superiority of the additive model over the recessive and dominant models).

The AUC results showed no absolute winner between the genotype and haplotype approaches (*p*-value was 0.8984 in a 2-tail Student t-test). The haplotype-based approach outperformed the genotype-based one in only 4 out of the 7 diseases analyzed, and the differences in AUC were low for most of these. The largest difference in AUC between the two approaches was reached in bipolar disorder (BD) (0.6222 for the genotype-based approach versus 0.6873 for the haplotype-based approach). However, the AUC is still a long way from its expected value even when only a quarter of the known genetic variance is explained by the variants included in the model (*AUC*_*quart*_=0.80), meaning that the potential this approach may have for some diseases is still very limited in terms of practical use, such as medical profiling of highly polygenic diseases.

Comparing the three different genetic models used, there do not seem to be any significant differences between them. Summary Table 6 shows the highest AUC achieved by each genetic model for each disease, among all the predictive methods and haplotype lengths used. Values are very similar. For hypertension, the AUC is exactly the same for the three genetic models (0.5573).

**Table 6:** Highest AUC obtained by the haplotype-based approach for all the genetic models used

Disease	Additive	Dominant	Recessive
BD	0.6873	0.687	0.6649
CAD	0.5736	0.5733	0.5761
HT	0.5573	0.5573	0.5573
IBD	0.6196	0.6184	0.6213
RA	0.8024	0.7968	0.7971
T1D	0.8682	0.8609	0.8633
T2D	0.6372	0.6364	0.6355

The highest AUC of all haplotype lengths and predictive methods used obtained for each disease by the haplotype-based approach for additive, dominant and recessive genetic models

## Discussion

Our starting point was the current lack of predictive models which are good enough [2] to be clinically useful, not even when applied to a sample at risk for which they should obtain an AUC of at least 0.75 [13, 24]. In order to assess whether this was just a problem in the approaches used or a lack of information processed, we conducted a wide analytic study to compare and improve the predictive capacity of different approaches and obtain as much information as possible from the genomic data sets. With this goal in mind, we substantially broadened the classic approaches in this task in two ways. First, by using other predictive models in addition to the classic (unweighted and weighted) GRSs among the state-of-the-art approaches in the machine learning field, including some which are able to consider variable interaction and which are robust to noisy or redundant variables (e.g. support vector machines, decision trees and random forests). Secondly, by using a haplotype-based approach similar to the one already proposed using 2-SNP haplotypes [24] but allowing larger haplotypes (from 2 to 5 SNPs) and different genetic models (additive, recessive and dominant).

In light of our results, it seems that some of the new learning approaches strongly outperform the classic methods for them to be used for diagnosis purposes when used with a sample at risk in two of the seven diseases analyzed: Type 1 diabetes and rheumatoid arthritis. This is the case of boosting methods, random forests algorithms and support vector machines in Type 1 diabetes and only boosting methods and random forests in rheumatoid arthritis. Their ability to model variable interaction, however, seems not to be the reason for them to work, as AdaBoostM1 assumes variable independence in the same way as logistic regression or naïve Bayes classifier do. The reason may be their robustness to noisy or redundant variables [18] as they always include a method for variable selection (sSVM), pruning (20RF, C4.5) or weighting (AdaBoostM1).

The fact that Type 1 diabetes and rheumatoid arthritis are autoimmune diseases may indicate some common genetic cause. Additional file 1: Table S1 shows common SNPs between the winning configuration for both diseases (AdaBoostM1 as machine learning and *p*-value threshold of 1*e*−5). All but four SNPs belong to the major histocompatibility complex (MHC).

**Fig. 5:**
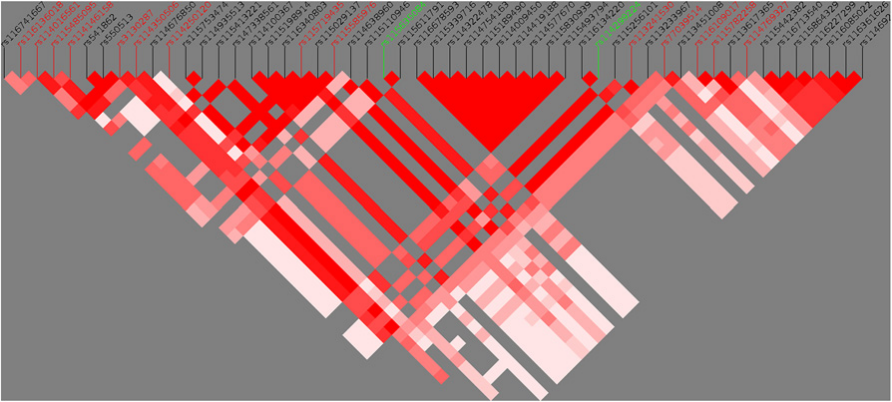
Chromosome 6 LD map with all positions related with autoimmune diseases. *D* ^′^ is used as the LD measure. All positions chosen by both the best Type 1 diabetes and the best rheumatoid arthritis predictive models were displayed (SNP id in black) in addition to all the positions chosen by the best multiple sclerosis model (in red). The color green was used for those positions shared by all

In order to compare these common SNPs with those selected in other autoimmune diseases, we have also added results for multiple sclerosis (MS). With this goal, we used genetic data from the International Multiple Sclerosis Genetic Consortium (IMSGC) [36] comprising 931 family trios, and built models under the same algorithms and *p*-value thresholds as the WTCCC diseases. As individuals are related, the association test used is the transmission-disequilibrium test (TDT) implemented in PLINK [37] so that the transmitted genotypes are considered to be high risk and the non-transmitted ones are considered to be low risk. By using family trios, the genome of each individual can be split into its two genome-wide haplotypes (one inherited from the father, the other inherited from the mother). In order to simplify this, the classifiers do not classify individual risk but genome-wide haplotype risk. The final column of Additional file 1: Table S3 shows the highest AUC for each algorithm among all the *p*-value thresholds used. The highest AUC (0.6167) was reached by 20RF and *p*-value threshold 1*e*−5, and the second highest was very close (AUC = 0.6162) and reached by AdaBoostM1 and *p*-value threshold 1*e*−6. A different microarray was used to genotype individuals in the IMSGC GWAS (Affymetrix 500K Set comprises *Mapping*250*K*_*N*_ *sp* and *Mapping*250*K*_*S*_ *ty* Arrays) from the one used by the WTCCC GWAS. Various positions, therefore, are not in both arrays although they may be in linkage disequilibrium (LD) between them. In order to see LD relationships between positions chosen by the multiple sclerosis model and those positions shared by the Type 1 diabetes and rheumatoid arthritis models, we have built an LD map (the LD statistic used was *D* ^′^). The color red means perfect LD (*D* ^′^=1) whereas white means *D* ^′^=0. Figure 5 shows this map which was built using BmapBuilder [38]. The positions in black are those only in Type 1 diabetes and rheumatoid arthritis models, the positions in red are those only in multiple sclerosis and the positions in green are shared by all of them. The ID position refers to the rs number. It is apparent that certain positions from different data sets share the same high LD block. One example of this are the positions rs115719435 (multiple sclerosis) and rs115029137 (Type 1 diabetes and rheumatoid arthritis).

It should be noted that since there is no clear winner for each disease, differences in model fitness obtained by different algorithms depend on the disease. In this paper, however, we have observed significant AUC superiority of AdaBoostM1, a robust algorithm to redundant or noisy variables, for the two diseases of Type 1 diabetes and rheumatoid arthritis in which AUC levels are high enough for the models to be clinically usable. Although for the other diseases, more classic algorithms (e.g. LR GRS) sometimes achieved the highest AUC (as exemplified by LR wGRS for BD and HT and NBC for CAD and IBD, respectively, in the genotype-based approach and NBC-all in the haplotype-based approach), AUC differences were not always significant and if they were, the AUC was not high enough to be used in medical care. In terms of the genotype versus haplotype approach, there is no clear winner but differences are apparent for highly polygenic diseases. As our expectations of achieving similar AUCs by using random forests, support vector machines and boosting algorithms were not satisfied, we attempted to understand the specific properties of the boosting algorithm AdaBoostM1 that enabled it to obtain the best results.

In this work we have only used a within-study validation approach (10-fold cross validation and holdout). We understand that the problem of spurious associations has not been completely solved [18] since cases and controls might have undergone different DNA preparation protocols, or were genotyped in different batches, or there might have been population stratification, etc. For a model to be thoroughly validated, therefore, it should be tested on an independent data set. However, the main goal of this work was merely to perform a wide comparative study in order to understand whether current methods enable the onset of certain diseases to be predicted from case/control GWAS. For this purpose, we believe that WTCCC data sets together with a within-study validation approach offered the best scenario.

## Conclusions

We have conducted extensive research to explore algorithms under very different approaches to model individual risk to 7 complex diseases from the WTCCC from genome-wide data. Our purpose was to understand whether current tools may be able to build predictive models which are accurate enough for application in medical care. In light of our results, it seems that for only two diseases with a high genetic component (rheumatoid arthritis and Type 1 diabetes) did certain models achieve a high enough predictive capacity for them to be used in clinical practice. The best of these were obtained for these two diseases by a boosting approach which is robust to redundant and noisy variables. Given the good performance of the boosting approach and the fact that we only considered one boosting algorithm (AdaboostM1), we believe that more systematic research of the boosting approach for building genome-wide genetic models could provide interesting insights.

## Methods

For our experiments we used published data from the WTCCC [2, 39], a GWAS from individuals genotyped using the Affymetrix 500K SNP chip and involving 7 different diseases: Bipolar disease (1998 individuals), coronary artery disease (1926 individuals), irritable bowel disease (2005 individuals), hypertension (2001 individuals), rheumatoid arthritis (1999 individuals), Type 1 diabetes (2000 individuals) and Type 2 diabetes (T2D) (1999 individuals). After undergoing quality control, we had genome-wide SNPs genotyped for 1868 individuals with bipolar disease, 1988 with coronary artery disease, 1748 with irritable bowel disease, 1952 with hypetension, 1860 with rheumatoid arthritis, 1963 with Type 1 diabetes and 1924 with Type 2 diabetes. For the control individuals, WTCCC used a data set from the 1958 British Birth Cohort (1504 healthy individuals) which was reduced to 1480 individuals after passing quality control. A rigorous quality control process was performed to remove low quality SNPs and individuals with doubtful ancestry or possible relatedness. The original paper [39] presents a full description of the data sets and quality control procedures.

Additionally and in order to avoid spurious association due to batch effects, genotyping errors and/or population stratification, we applied other more stringent SNP cleaning as performed by Evans et al. [2], excluding any SNPs that were not in Hardy-Weinberg equilibrium (*p*-value *p*<0.05 in cases and controls), those with different missing rates between cases and controls (*p*-value *p*<0.05) and those with a minor allele frequency of less than 1 *%*.

After all the quality controls, we combined all the control individuals with all the cases for each disease and obtained 7 data sets, one for each disease. With these data sets, we performed various analyses within two clearly different approaches regarding how input variables were defined: first, the genotype-based approach, using single SNPs as input variables of three values (homozygous wild-type, homozygous mutant and heterozygous); and second, the haplotype-based approach, using inferred allelic information within each chromosome. This second approach has already been used in case/control genetic predictors for 2-SNP haplotypes [24] and in trio samples for longer haplotypes up to those comprising 150 SNPs [28].

### *p*-value thresholds

The choice of *p*-value threshold to select the SNPs or haplotypes that will be used as input variables in a genetic predictor may influence its performance. At one extreme, too liberal thresholds are supposed to reduce accuracy because of noise [2]. However, most modern approaches for learning models from highly dimensional data introduce a way to increase robustness to noisy data [40]. At the other extreme, very stringent thresholds may discard small effects that contribute to the disease risk. In order to study the true effect of different thresholds in prediction, we chose a wide range of *p*-value thresholds which was similar to Evans et al. (2009) [2]: *α*=0.8;*α*=0.5;*α*=0.1;*α*=0.05;*α*=0.01;*α*=0.001;*α*=0.0001;*α*=0.00001.

### Discriminative ability and generalization capacity (accuracy, recall, precision, sensitivity, specificity and AUC)

In order to measure the predictor performance in terms of discriminative ability, we used six different evaluation metrics: overall accuracy, recall, precision, sensitivity, specificity and AUC. Overall accuracy is the proportion of individuals correctly classified. The main problem of this measure is that its interpretation depends on the marginal distribution of cases and controls. Precision is the positive predictive value, i.e. the proportion of individuals classified as affected that are truly affected. Sensitivity is the true positive rate, i.e. the proportion of affected individuals correctly classified. Specificity is the true negative rate, i.e. the proportion of healthy individuals correctly classified. The AUC measures the discriminative ability regarding the cost of misclassification in cases and controls and the marginal distribution of cases and controls. The receiving operating curve (ROC) plots a false positive rate (1-specificity) on the x-axis and a true positive rate (sensitivity) on the y-axis. A ROC curve on the diagonal means the predictor is as inaccurate as guessing and the AUC will be 0.5. The maximum AUC is 1 and corresponds not to a curve but to a vertical line at x = 0 (specificity = 1) and a horizontal line at y = 1 (sensibility = 1). Any curve above the diagonal will have an AUC greater than 0.5. The AUC merely compares the overall distributions of correctly classified cases and wrongly classified controls.

Measuring the discriminative ability of a genetic predictor through the same data set used to learn it (i.e. the training data set) does not convey its generalization capacity. Very simple models may have a low performance but better generalize when tested on an independent data set. On the other hand, more complex models may show a high performance but have no generalization capacity at all because they overfit to the training data set. Therefore, all the measures used to test discriminative ability have been applied on an independent data set, the test data set. For models learned within a feasible computational time such as the genotype-based predictors in this work, we used a multisample model validation, the 10-fold cross-validation, in which the original data set is randomly split into 10 non-overlapping subsets and for each subset the test data set is one subset and all the remaining subsets comprise the training data set, from which the measures mentioned above were computed. The average results were then calculated. For more time-demanding models, i.e. those representing haplotype-based predictors, we simply randomly divided the original data set into two subsets of equal size and used one as the training set and the other as the test set.

### Learning machines

We used different learning machines or algorithms able to learn models from a training data set.

#### Simple approaches: simple logistic regression and naïve Bayes classifiers

We first built predictive models from each training data set following the state-of-the-art methods based on simple logistic regression

$$\ln O(x)=\ln\frac{p(D\mid x)}{1-p(D\mid x)}=\alpha_{0}+\alpha_{1} g(x) $$

where *g*(*x*) may be a GRS defined as $GRS(x)=\sum _{j=1}^{n} x_{i}$ or a wGRS defined as $wGRS(x)=\sum _{j=1}^{n} w_{i} x_{i}$ with *n* being the total number of selected SNPs, *w*_*i*_ being the allelic odds ratio defined as

$$w_{i}=\ln {OR}_{i}=\ln\frac{p(D\mid h_{i}=1}{p(\bar D\mid h_{i}=1)}\frac{p(\bar D\mid h_{i}=0)}{p(D\mid h_{i}=0)}, $$

*D* and $\bar D$ indicating whether an individual has the disease or not and *h*_*i*_ a binary variable that refers to any of the two alleles *h*_*i*1_,*h*_*i*2_ at position *i* so that *x*_*i*_=*h*_*i*1_+*h*_*i*2_ holds for every *i*=1,…,*n*. The odds ratio required to compute *wGRS* and parameters *α*_0_ and *α*_1_ were all learned from the training data set.

Another simple model used is the naïve Bayes classifier. This model assumes independent input variables (SNPs in our study) given the output variable (the disease outcome in our study) based on genotypes:

$${} p(D\mid x)=\frac{p(D)\prod_{i=1}^{n}(p(x_{i}\mid D)}{p(D)\!\prod_{i=1}^{n} p(x_{i}\mid D)\,+\,(\!1-p(D)\!)\!\prod_{i=1}^{n} p(x_{i}\mid \bar D)} $$

In terms of the AUC, a *wGRS* should be equivalent to a naïve Bayes classifier for any choice of parameters *α*_0_,*α*_1_, as the parameters do not affect the AUC [25].

We also used a naïve Bayes classifier based on alleles and assumed that *h*_*ij*_,*j*=1,2 are identically distributed and are conditionally independent given *D*. This is equivalent to the simple logistic regression with parameters

$$\alpha_{0}=\ln\frac{p(D)}{1-p(D)}+2\sum_{i=1}^{n}\ln\frac{p(h_{1}=0\mid D)}{p(h_{i}=0\mid \bar D)} $$

and *α*_1_=1 [31].

#### More complex approaches: support vector machines, boosting methods, decision trees, random forests

In order to know whether the predictive capacity may be limited by the simplicity of the state-of-the-art genome-based models, we used very different approaches in the machine learning field which were capable of building more complex models from data: support vector machines [40], decision trees, random forests and boosting methods. We chose one algorithm within each approach and its implementations in Weka [41], a software “workbench” implementing several standard machine learning techniques. From the various published support vector machines implemented in Weka, we chose a sigmoid kernel function (sSVM) since it performed best. For the decision tree we chose the leading-edge C4.5 algorithm (called J45 in Weka) [42]. For the approach based on random forests we tried 20 trees (20RF) (default configuration is 10) with a maximum depth of 6 to avoid overfitting (no restriction by default). For the boosting methods we used the trendy AdaBoostM1 [33] using decision stumps, i.e. one-level decision trees or a single decision rule, as weak classifiers (default configuration) and 2500 iterations (the default 10 is too low for models with thousands of low-impact variables, as is the case of predicting complex diseases from GWAS).

### Genotype-based predictors

Original data are genome-wide, three-value variables representing the genotype an individual has at each locus. The output variable is a binary one representing whether the individual is a case or a control. We performed a 10-fold cross-validation approach as explained by [2]. For each fold, only 90 *%* of individuals comprised the training subset, which was used to learn the model. In order to decide whether an SNP should be used as an input variable of the model, the same training subset was used to compute the *p*-value for the Armitage trend test implemented in PLINK [37], and any SNP with a *p*-value below the threshold was selected. The remaining 10 *%* comprised the test data set. The median accuracy, precision, specificity, sensitivity and AUC were estimated from the results of these 10 analyses.

### Haplotype-based predictors

For our study, we used 4 different haplotype sizes (from 2 to 5) and three different genetic models: recessive, dominant and additive models on the absolute risk of the genome-wide haplotypes. We built models for each of the 7 diseases and genotype-based predictors were also built for these. We also used information about chromosome-wide haplotypes to build the models [31]. As previously mentioned, one approach using only the additive model and haplotypes of only 2 SNPs has already been used to predict the risk of Crohn’s disease [24]. Linkage equilibrium was assumed between haplotypes (i.e. no association between haplotypes) so that the model only had a multiplicative effect on the odds of each haplotype (additive on log odds) [31].

For each model we first reconstructed genome-wide haplotypes from genotypes for each individual using Shapeit [43], software for fast and accurate haplotype inference. The second step was to test the haplotype-based association between each locus and the disease. The main problem of using haplotypes as input variables concerns sample reproducibility: the longer the haplotypes, the higher the chances of spurious associations due to a small sample size. In order to avoid this problem we extended the multimarker transmission-disequilibrium test (mTDT) for nuclear families *mTDT*_2*G*_ [29], which is robust to haplotype size and does not overfit to current haplotypes or to case/control data sets. The *mTDT*_2_ *G* statistic for family trios measures the differences in transmissions of *g*_1_, a group of haplotypes comprising the haplotypes that are more often than not transmitted from parents to offspring in an independent data subset versus *g*_2_, a group of haplotypes comprising those haplotypes that are more often not transmitted than transmitted from parents to offspring, and this is defined as

$${mTDT}_{2G}=\frac{\left(n_{g_{1}g2}-n_{g_{2}g_{1}}\right)^{2}}{n_{g}}, $$

with $n_{g_{1}g_{2}}$, $n_{g_{2}g_{1}}$ defined respectively as:

$$n{g_{1}g_{2}}=\sum_{h_{i}\in g_{1}, h_{j}\in g_{2}} n_{ij} \; and $$

$$n{g_{2}g_{1}}=\sum_{h_{i}\in g_{2}, h_{j}\in g_{1}} n_{ij}, $$

where *n*_*ij*_ is the number of parents with genotype (*h*_*i*_/*h*_*j*_) transmitting haplotype *h*_*i*_ to their offspring, *n*_*ji*_ is the number of parents with genotype (*h*_*i*_/*h*_*j*_) transmitting haplotype *h*_*j*_ to their offspring and *n*_*g*_ is the total number of parental genotypes in the data subset with one haplotype in *g*_1_ and the other in *g*_2_. The data set is divided into two equally-sized parts for test application: half of each part is used to form the two groups and the other half to compute statistics. *mTDT*_2*G*_ is a McNemar test (*χ* ^2^) under the null hypothesis of no linkage.

*mAssocTest*_2*G*_, the extension of *mTDT*_2*G*_ to be applied in case/control GWAS, is defined as:

$$\begin{aligned} {mAssocTest}_{2G}&=\frac{(n_{cas-g_{1}}-n_{cont-g_{1}})^{2}}{n_{g_{1}}}\\&\quad+\frac{(n_{cas-g_{2}}-n_{cont-g_{2}})^{2}}{n_{g_{2}}}, \end{aligned} $$

with$n_{cas-g_{1}}$,$n_{cont-g_{1}}$ defined respectively as the number of cases and control haplotypes belonging to group *g*_1_. In a similar way, $n_{cas-g_{2}}$,$n_{cont-g_{2}}$ are also defined.$n_{g_{1}}$ is the total count of haplotypes in *g*_1_ and$n_{g_{2}}$ the total count of haplotypes in *g*_2_. As with *mTDT*_2*G*_, the data set, the training data set in our case, is divided into two equally-sized parts for test application: one part is used to comprise the two groups and the other to compute the statistic. *mTDT*_2*G*_ is a *χ* ^2^ test with 2 degrees of freedom.

For a better understanding of how *mAssocTest*_2*G*_ is computed, let us consider Table 7 of haplotype counts of length 3 obtained from half of a data set analyzed with 100 individuals. For the sake of simplicity, major and minor alleles at all loci are represented as 1 and 0, respectively.

**Table 7:** Understanding *mAssocTest*_2*G*_ (I): a dataset should be split and haplotype counts obtained from half the dataset

Haplotypes	000	001	010	011	100	101	110	111	Total counts
Case	13	8	11	9	14	17	16	12	100
Control	12	11	19	7	16	13	2	20	100
	25	19	30	16	30	30	18	32	200

Example of haplotype counts from half a case/control data set

From this table, group *g*_1_ comprises the haplotypes that are more frequent in cases than in controls: *g*_1_={000,011,101,110}, and therefore all the remaining haplotypes comprise the second group: *g*_2_={001,010,100,111}. From these two groups, the second data subset is used to compute the statistic. Table 8 shows haplotype counts for the two groups from the second data subset.

**Table 8:** Understanding *mAssocTest*_2*G*_ (II): haplotype counts from the other half of the data set are to be used to compute the statistic

	*g*_1_	*g*_2_	
Case	53	47	100
Control	38	62	100
	91	109	200

Example of group counts from the other part of a case/control data set

Thus, ${mAssocTest}_{2G}=\frac {(53-38)^{2} + (47-62)^{2}}{200}=\frac {225}{91}+\frac {225}{109}=4.5368$ and *p*-value is *p*=0.033175.

In the third step, once we had computed *mAssocTest*_2*G*_ on the training data set for sliding windows with an offset of 1 and different haplotype lengths (from 1 to 5), we applied different levels of loci filtering (the 13 *p*-value upper limits previously mentioned) in order to select the input variables of the haplotype-based predictor of individual risk *pInd*_*h*_(*i*).

In the fourth step, we learned the predictors using all of the previously mentioned approaches from the second half of the training data set, i.e. those individuals used to compute the *mAssocTest*_2*G*_ statistic. The haplotype-based predictor is defined on the basis of a predictor of haplotype risk, *pHap*(*h*). The log odds for each genome-wide homologous chromosome of an individual are therefore combined in order to estimate its individual risk. Each genome-wide homologous chromosome comprises one of the two chromosomes for each 22 chromosome pair. The input variables for the predictor of haplotype risk are binary ones, representing whether a haplotype belongs to *g*_1_ or *g*_2_. The output variable for the predictor of haplotype risk is the probability of a given genome-wide haplotype to be a high-risk haplotype. We consider that both genome-wide haplotypes comprising the genome of an unaffected individual must be low-risk haplotypes while both genome-wide haplotypes comprising the genome of a diseased individual must be high-risk ones. Only individuals in the second half of the training data set (i.e. those used to compute the statistic) are used to build the haplotype risk predictor.

**Fig. 6:**
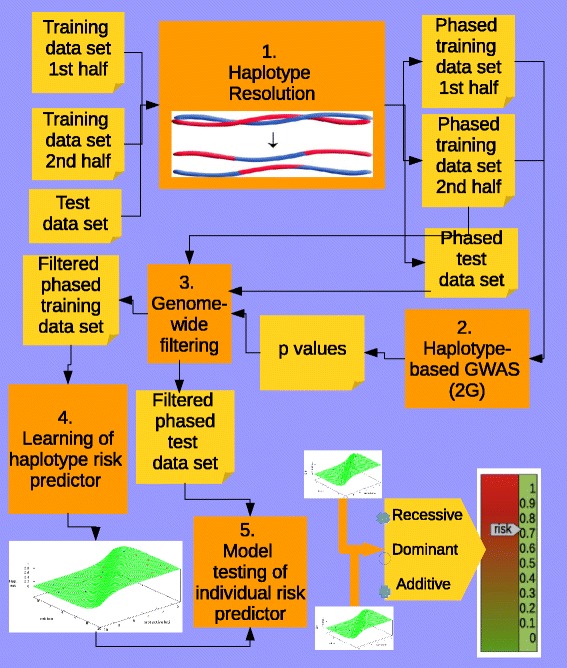
Summary of the five steps followed by the haplotype-based approach. The training data set was split and genotypes within each subset were independently phased using Shapeit (first step). From one half of the training data set, *mAssocTest*_2*G*_ was used to estimate the association between haplotypes (lengths considered were 2 to 4) and phenotype (second step). Different *p*-value thresholds were used to select the input variables (comprising 2 to 4 SNP-length haplotypes) (third step). Models of haplotype risk predictors were built using the second half of the training data set (fourth step). Individual risk was assessed by combining the two genome-wide haplotypes each individual has (fifth step)

In the fifth and final step, we used the predictors to measure their generalization capacity, by feeding them with individuals from the test data set. It should be noted that for small data sets and haplotypes comprising a few positions there may be variants in the test data set that are not present in the training data set. In order to decide whether a haplotype at a given sliding window was a high (1) or low (0) risk one, we computed the similarity between it and each haplotype in the list of high risk and low risk haplotypes for the corresponding sliding window in the training data set. We therefore classified it as 1 or 0 depending on whether the closest haplotype belonged to the set of high or low risk haplotypes, respectively [31]. We used the length measure as the similarity measure [44], which computes the largest number of consecutive matching alleles. Figure 6 summarizes the entire procedure of our haplotype-based approach.

## Additional file

Additional file 1 **Supplementary material [** 45 **].** (PDF 502 kb)
