# Supplementary material for: A comparison of genomic profiles of complex diseases under different models
Source: BMC Med Genomics. 2016 Jan 19;9:3. doi: 10.1186/s12920-015-0157-2 (PMC4717655; doi:10.1186/s12920-015-0157-2)
Supplement: Supplementary file 1 — Supplementary material [ 45 ]. (PDF 502 kb) [file 12920_2015_157_MOESM1_ESM.pdf]

A comparison of genomic profiles of complex diseases under different models
Supplementary material

Víctor Potenciano[1], María M. Abad-Grau*[1] , Antonio Alcina[2] and Fuencisla Matesanz[2]

[1]Departamento de Lenguajes y Sistemas Informáticos, ETSIIT,
c/ Periodista Daniel Saucedo Aranda s/n Universidad de Granada, Granada 18071, Spain
[2]Instituto de Parasitología y Biología Molecular, CSIC, Granada, Spain

Email: Víctor Potenciano - vpoten@correo.ugr.es; María M. Abad-Grau*- mabad@ugr.es; Antonio Alcina - pulgoso@ipbln.csic.es; Fuencisla Matesanz - lindo@ipbln.csic.es; Víctor Potenciano - vpoten@correo.ugr.es; María M. Abad-Grau*- mabad@ugr.es; Antonio Alcina - pulgoso@ipbln.csic.es; Fuencisla Matesanz - lindo@ipbln.csic.es;

*Corresponding author

1 Diseases under study ordered by their genetic component or polygenic level, measured by AUC_{max} (source: [13]).

Disease	disease prevalence ($K \times 100$)	heritability (siblings recurrence risk λ_s)	AUC_{max}
T2D	3	3.5	0.94
CAD	5.6	3.2	0.95
BD	1	6.8	0.97
RA	0.75	8	0.98
T1D	0.54	13.7	1
IBD	0.1	26	1

2 Some AUC results of genome-wide predictors for different complex diseases found in the literature and mentioned in the Introduction.

Acronyms: ds (derivation sample), MS (multiple sclerosis), LC (lung cancer), CD (Crohn's disease), AD (Alzheimer's disease), AMD (age-related macular degeneration), ids (independent data set), ss (shared samples between selection and validation sets).

Disease	predictive AUC	AUC_{quar}	learning machine	case/control sample size (ds)	validation approach	reference	issues
MS	0.70 (cv), 0.64 (ids)	N/A	wGRS LR	2000/ 1500	10-fold cv and ids	[1]	ss
MS	N/A	N/A	OR	931/2431	training	[36]	
LC	0.639	0.80	wGRS LR			[4]	
CD	0.72	0.86	GRS LR, SVM, wGRS LR			[24]	ss
MS	0.76	N/A	GRS LR	1618/1988	ids	[7]	gender used in predictor
AD	0.73		GRS LR, NBC, EBMC, SVM	861/550	5-fold cv	[45]	
T1D	0.89 (cv), 0.84	0.84 0.84	SVM	1529/1458	5-fold cv, ids	[18]	ss
AMD	0.82	0.72	wGRS LR	986/796	10-fold cv	[19]	results for extreme AMD

3 Acronyms used

List of achronyms used for diseases (left panel) and predictive methods (right panel).

Diseases		Predictive methods	
Acronym	full text	Acronym	full text
BD	bipolar disorder	GLM AIC	stepwise generalized linear models with AIC to select the number of SNPs
CAD	coronary artery disease	LR GRS	logistic regression genetic risk score
HT	hypertension	LR wGRS	logistic regression weighted genetic risk score
IBD	irritable bowel disease	NBC	naïve Bayes classifier
MS	multiple sclerosis	aNBC	allelic naïve Bayes classifier
RA	rheumatoid arthritis	sSVM	sigmoid support vector machine
T1D	type I diabetes	20RF	20 random forest
T2D	type II diabetes	RR	ridge regression

4 List of common SNPs between T1D, RA and MS

Chromosome positions correspond to assembly NCBI dbSNP GRCh38.p2.

chr	SNP id	chr pos	chr	SNP id	chr pos
19	$SNP_A - 4281637$	39266932	6	$SNP_A - 2024935$	32345320
1	$SNP_A - 1827111$	77051324	6	$SNP_A - 2029466$	32744005
1	$SNP_A - 2000304$	113872746	6	$SNP_A - 2063969$	32420083
1	$SNP_A - 2235405$	113630788	6	$SNP_A - 2073634$	32441065
6	$SNP_A - 1780811$	32396039	6	$SNP_A - 2087041$	32721752
6	$SNP_A - 1802332$	32320413	6	$SNP_A - 2174854$	32337913
6	$SNP_A - 1815451$	32394044	6	$SNP_A - 2177365$	32421528
6	$SNP_A - 1826835$	32417322	6	$SNP_A - 2182330$	32312814
6	$SNP_A - 1841603$	32768367	6	$SNP_A - 2185852$	32627306
6	$SNP_A - 1844183$	32419975	6	$SNP_A - 2215422$	31952910
6	$SNP_A - 1854393$	32722224	6	$SNP_A - 2228002$	32268365
6	$SNP_A - 1856736$	32305381	6	$SNP_A - 2254578$	31949174
6	$SNP_A - 1912260$	32417693	6	$SNP_A - 2281364$	32240547
6	$SNP_A - 1946221$	32320685	6	$SNP_A - 2301910$	32415633
6	$SNP_A - 1956551$	32275378	6	$SNP_A - 4206382$	31275393
6	$SNP_A - 1985623$	32415856	6	$SNP_A - 4225595$	**32421735**
6	$SNP_A - 1985624$	32416081	6	$SNP_A - 4240800$	32723168
6	$SNP_A - 1985626$	32420531	6	$SNP_A - 4251181$	32702467
6	$SNP_A - 1985640$	32770338	6	$SNP_A - 4252433$	32768326
6	$SNP_A - 2011688$	**32395750**	6	$SNP_A - 4269701$	32420797
6	$SNP_A - 2011689$	32420932	6	$SNP_A - 4303523$	32444815
6	$SNP_A - 2011691$	32481273			

Table S1: List of common SNPs between T1D and RA in the winner models (AdaBoostM1 with p-value threshold $1e-5$) of both diseases. In bold those SNPs also in common with MS winner model (RF and p-value threshold $1e-5$). See Supplementary Table S3.

5 Summary of best AUC results under the genotype-based approach using cross validation

Algorithm	BD	CAD	HT	IBD	RA	T1D	T2D
GRS LR	0.6614	0.597	0.5691	0.6144	0.6786	0.6813	0.6024
wGRS LR	0.6618	0.607	0.6039	0.6352	0.718	0.7045	0.6051
NBC	0.6541	0.6292	0.6036	0.6637	0.6653	0.726	0.6184
aNBC	0.653	0.6071	0.6003	0.6347	0.7248	0.7013	0.6001
sSVM	0.6067	0.5898	0.565	0.6109	0.7216	0.7849	0.568
AdaBoostM1	0.6205	0.6233	0.5929	0.6732	0.8087	0.8805	0.6257
C4.5	0.562	0.5865	0.5636	0.5793	0.6635	0.7262	0.5743
20RF	0.568	0.5856	0.5728	0.6234	0.7045	0.7337	0.5902

Table S2: Summary of best AUC results among all p-value thresholds under the genotype-based approach using 10-fold cross validation

6 Summary of best AUC results under the genotype-based approach using holdout

Algorithm	BD	CAD	HT	IBD	RA	T1D	T2D	MS
GRS LR	0.6215	0.6034	0.5672	0.5874	0.6724	0.7126	0.5789	-
wGRS LR	0.6222	0.6013	0.5728	0.5905	0.7002	0.7294	0.5868	-
NBC	0.6111	0.7653	0.5742	0.5916	0.7119	0.5719	0.5832	0.6138
sSVM	0.5617	0.5747	0.54	0.5731	0.7147	0.7874	0.5829	0.59
AdaBoostM1	0.5951	0.6028	0.5776	0.6136	0.8152	0.8615	0.6134	0.6162
C4.5	0.5592	0.5727	0.549	0.5762	0.7335	0.7278	0.5739	0.611
20RF	0.5393	0.611	0.5396	0.5921	0.7592	0.8112	0.5843	0.6167

Table S3: Summary of best AUC results among all p-value thresholds under the genotype-based approach using holdout

7 Summary of best AUC results under the haplotype-based approach

Algorithm	BD	CAD	HT	IBD	RA	T1D	T2D
NBC	0.6819	0.5724	0.5573	0.6125	0.7439	0.7799	0.6299
sSVM	0.5862	0.5316	0.5438	0.5587	0.6731	0.7608	0.5668
AdaBoostM1	0.6873	0.5736	0.5409	0.6213	0.8024	0.8682	0.6372
C4.5	0.6511	0.5341	0.5371	0.6198	0.7068	0.7057	0.6194
20RF	0.6564	0.5751	0.5544	0.6188	0.7517	0.8121	0.6367

Table S4: Summary of best AUC results among all p-value thresholds, genetic model and haplotype lengths under the haplotype-based approach using holdout

8 Detailed results under the genotype-based approach using cross validation

Supplementary Tables S5 to S9 give respectively detailed results of AUC, sensitivity, specificity, precision and overall accuracy returned by all the algorithms used and all p-value thresholds for the 7 diseases when using a 10-fold cross validation approach.

p-value threshold	BD	CAD	HT	IBD	RA	T1D	T2D
			GRS LR				
0.8	0.6539	0.5930	0.5907	0.6388	0.5852	0.5841	0.6009
0.5	0.6592	0.5995	0.5925	0.6467	0.5843	0.5980	0.5970
0.1	0.6486	0.5743	0.5904	0.6216	0.6114	0.6339	0.6038
0.05	0.6409	0.5638	0.5815	0.6133	0.6241	0.6425	0.5941
0.01	0.5973	0.5891	0.5699	0.6105	0.6512	0.6617	0.5858
0.0010	0.5427	0.5987	0.5733	0.6268	0.6680	0.6919	0.5904
1.0e-4	0.5403	0.6089	0.5626	0.6374	0.6705	0.6971	0.5934
1.0e-5	0.5582	0.5977	0.5369	0.6403	0.6685	0.7032	0.5842
			wGRS LR				
0.8	0.6687	0.6049	0.6028	0.6513	0.6367	0.6648	0.6184
0.5	0.6664	0.6062	0.6023	0.6517	0.6383	0.6694	0.6160
0.1	0.6519	0.5883	0.5985	0.6329	0.6725	0.6918	0.6114
0.05	0.6416	0.5802	0.5887	0.6278	0.6883	0.7008	0.6015
0.01	0.6019	0.6042	0.5774	0.6309	0.7067	0.7118	0.5993
0.0010	0.5539	0.6175	0.5770	0.6514	0.7136	0.7251	0.6060
1.0e-4	0.5540	0.6280	0.5658	0.6639	0.7098	0.7254	0.6102
1.0e-5	0.5650	0.6151	0.5425	0.6641	0.7064	0.7293	0.6140
			aNBC				
0.8	0.6455	0.5853	0.5930	0.6317	0.6214	0.6412	0.6036
0.5	0.6438	0.5888	0.5936	0.6347	0.6222	0.6490	0.6004
0.1	0.6438	0.5846	0.5920	0.6258	0.6690	0.6777	0.6072
0.05	0.6378	0.5795	0.5865	0.6255	0.6888	0.6906	0.6004
0.01	0.6018	0.6043	0.5774	0.6306	0.7116	0.7043	0.5990
0.0010	0.5538	0.6174	0.5770	0.6511	0.7196	0.7211	0.6057
1.0e-4	0.5540	0.6279	0.5658	0.6634	0.7162	0.7234	0.6099
1.0e-5	0.5650	0.6149	0.5423	0.6635	0.7132	0.7282	0.6136

Table S5: AUC obtained by all the algorithms under the genotype-based approach with 10-fold cross validation. Results for each p-value threshold (column 1) are shown. For those algorithms demanding more computational time (sSVM, AdaBoostM1, C4.5 and 20RF), the maximum p-value threshold used was 0.1.

Table S5 (cont.)

p-value threshold	BD	CAD	HT	IBD	RA	T1D	T2D
NBC							
0.8	0.6531	0.6050	0.6010	0.6279	0.6450	0.6488	0.6184
0.5	0.6541	0.6035	0.6036	0.6299	0.6446	0.6515	0.6127
0.1	0.6470	0.5899	0.6031	0.6273	0.6754	0.6822	0.6134
0.05	0.6400	0.5838	0.5970	0.6242	0.6904	0.6940	0.6042
0.01	0.6085	0.6050	0.5814	0.6277	0.7146	0.7077	0.5985
0.0010	0.5601	0.6181	0.5837	0.6505	0.7233	0.7208	0.6046
1.0e-4	0.5526	0.6292	0.5757	0.6637	0.7191	0.7220	0.6080
1.0e-5	0.5646	0.6186	0.5525	0.6634	0.7170	0.7260	0.6122
sSVM							
0.1	0.6067	0.5426	0.5650	0.5707	0.5725	0.6084	0.5680
0.05	0.6024	0.5365	0.5549	0.5688	0.5830	0.6321	0.5635
0.01	0.5765	0.5485	0.5536	0.5611	0.6089	0.6579	0.5525
0.0010	0.5484	0.5678	0.5746	0.5558	0.6416	0.7158	0.5452
1.0e-4	0.5486	0.5898	0.5525	0.5982	0.6957	0.7652	0.5542
1.0e-5	0.5565	0.5806	0.5389	0.6109	0.7216	0.7849	0.5638
AdaBoostM1							
0.1	0.6086	0.6213	0.5625	0.6283	0.7680	0.8475	0.5862
0.05	0.6073	0.5975	0.5633	0.6361	0.7654	0.8379	0.5920
0.01	0.6205	0.5951	0.5540	0.6199	0.7479	0.8258	0.5996
0.0010	0.6015	0.6233	0.5861	0.5990	0.7717	0.8329	0.5726
1.0e-4	0.5665	0.6208	0.5929	0.6546	0.7931	0.8702	0.5956
1.0e-5	0.5771	0.6231	0.5782	0.6732	0.8087	0.8805	0.6257
C4.5							
0.1	0.5256	0.5150	0.5198	0.5236	0.5932	0.6531	0.5302
0.05	0.5065	0.5055	0.5149	0.5284	0.6175	0.6562	0.5236
0.01	0.5253	0.5351	0.5234	0.5297	0.6257	0.6638	0.5185
0.0010	0.5167	0.5192	0.5312	0.5382	0.6223	0.7030	0.5256
1.0e-4	0.5302	0.5462	0.5410	0.5642	0.6607	0.7210	0.5463
1.0e-5	0.5620	0.5865	0.5636	0.5793	0.6635	0.7262	0.5743
20RF							
0.1	0.5096	0.5035	0.4996	0.4974	0.5053	0.5143	0.5065
0.05	0.4995	0.5057	0.5192	0.5012	0.5330	0.5188	0.5115
0.01	0.5138	0.5079	0.5312	0.5010	0.5190	0.5511	0.5135
0.0010	0.5407	0.5307	0.5287	0.5541	0.6154	0.6611	0.5185
1.0e-4	0.5455	0.5662	0.5647	0.6015	0.6865	0.7320	0.5522
1.0e-5	0.5680	0.5856	0.5728	0.6234	0.7045	0.7337	0.5902

p-value threshold	BD	CAD	HT	IBD	RA	T1D	T2D
				GRS LR			
0.8	0.7422	0.7135	0.7316	0.6474	0.6978	0.7320	0.7368
0.5	0.7481	0.7244	0.7439	0.6594	0.7059	0.7411	0.7295
0.1	0.7321	0.6927	0.7199	0.6720	0.7038	0.7178	0.7171
0.05	0.7070	0.6803	0.6985	0.6606	0.7097	0.6949	0.6865
0.01	0.6567	0.6658	0.6633	0.6503	0.6618	0.6716	0.6725
0.0010	0.6032	0.6777	0.6740	0.6657	0.6790	0.6944	0.6440
1.0e-4	0.6433	0.7290	0.7107	0.6983	0.7140	0.7198	0.6959
1.0e-5	0.7626	0.7886	0.8071	0.7291	0.7478	0.7310	0.7482
				wGRS LR			
0.8	0.7524	0.7394	0.7582	0.6354	0.7409	0.7701	0.7544
0.5	0.7535	0.7409	0.7582	0.6611	0.7382	0.7695	0.7591
0.1	0.7455	0.7062	0.7429	0.6691	0.7554	0.7025	0.7363
0.05	0.7123	0.6974	0.7066	0.6669	0.7226	0.7015	0.7010
0.01	0.6583	0.6850	0.6760	0.6749	0.7048	0.6964	0.6943
0.0010	0.6118	0.6959	0.6888	0.6760	0.7140	0.7107	0.6674
1.0e-4	0.6775	0.7399	0.7408	0.7103	0.7280	0.7310	0.7233
1.0e-5	0.7722	0.7762	0.8316	0.7269	0.7409	0.7360	0.7585
				aNBC			
0.8	0.7396	0.7275	0.7474	0.6851	0.7333	0.7579	0.7466
0.5	0.7401	0.7254	0.7408	0.6811	0.7323	0.7533	0.7466
0.1	0.7262	0.6979	0.7209	0.6594	0.7317	0.7066	0.7192
0.05	0.6979	0.6881	0.6954	0.6571	0.7113	0.6736	0.6860
0.01	0.6337	0.6725	0.6505	0.6606	0.6785	0.6249	0.6689
0.0010	0.5957	0.6560	0.6362	0.6343	0.6747	0.5822	0.6358
1.0e-4	0.6053	0.6415	0.6531	0.6543	0.6720	0.5716	0.6259
1.0e-5	0.6620	0.6513	0.7464	0.6560	0.6806	0.5919	0.6332
				NBC			
0.8	0.8107	0.8238	0.8372	0.7360	0.8134	0.8310	0.8145
0.5	0.7936	0.7948	0.8046	0.7183	0.7898	0.8102	0.7896
0.1	0.7278	0.7285	0.7403	0.6897	0.7527	0.7244	0.7399
0.05	0.7118	0.7073	0.7255	0.6720	0.7323	0.6944	0.7114
0.01	0.6540	0.6741	0.6577	0.6583	0.6892	0.6234	0.6684
0.0010	0.6107	0.6570	0.6464	0.6400	0.6823	0.5761	0.6228
1.0e-4	0.6214	0.6627	0.6577	0.6600	0.6806	0.5680	0.6280
1.0e-5	0.6631	0.6762	0.7459	0.6554	0.6866	0.5787	0.6378

Table S6: Sensitivity obtained by all the algorithms under the genotype-based approach with 10-fold cross validation. Results for each p-value threshold (column 1) are shown. For those algorithms demanding more computational time (sSVM, AdaBoostM1, C4.5 and 20RF), the maximum p-value threshold used was 0.1.

Table S6 (cont.)

p-value threshold	BD	CAD	HT	IBD	RA	T1D	T2D
			sSVM				
0.1	0.7775	0.7554	0.7760	0.6954	0.7430	0.7944	0.7658
0.05	0.7642	0.7285	0.7362	0.6714	0.7247	0.7959	0.7311
0.01	0.6888	0.7052	0.6923	0.6526	0.7204	0.7868	0.6860
0.0010	0.6583	0.7005	0.6923	0.6286	0.7237	0.8168	0.6580
1.0e-4	0.6775	0.7404	0.7036	0.6720	0.7522	0.8452	0.6922
1.0e-5	0.8535	0.8057	0.8750	0.7171	0.7554	0.8645	0.8114
			AdaBoostM1				
0.1	0.6626	0.6896	0.6536	0.6463	0.7301	0.8325	0.6746
0.05	0.6802	0.6544	0.6454	0.6537	0.7274	0.8330	0.6731
0.01	0.6759	0.6523	0.6638	0.6337	0.7306	0.8299	0.6699
0.0010	0.6444	0.6601	0.6526	0.6040	0.7446	0.8234	0.6503
1.0e-4	0.6364	0.6746	0.6867	0.6737	0.7355	0.8508	0.6772
1.0e-5	0.7465	0.7197	0.7551	0.7069	0.7430	0.8599	0.7482
			C4.5				
0.1	0.5781	0.5642	0.5663	0.5589	0.6323	0.7340	0.5912
0.05	0.5610	0.5855	0.5827	0.5674	0.6597	0.7340	0.5736
0.01	0.5636	0.5886	0.5857	0.5703	0.6661	0.7452	0.5953
0.0010	0.5824	0.5922	0.5852	0.5909	0.6887	0.7797	0.5834
1.0e-4	0.5920	0.6093	0.6219	0.6097	0.7011	0.8056	0.6363
1.0e-5	0.7374	0.7005	0.7429	0.6343	0.6957	0.8041	0.6819
			20RF				
0.1	0.9251	0.9497	0.9551	0.8326	0.9253	0.9528	0.9477
0.05	0.9251	0.9446	0.9485	0.8377	0.9328	0.9533	0.9394
0.01	0.9225	0.9394	0.9515	0.8463	0.9172	0.9599	0.9435
0.0010	0.9219	0.9446	0.9454	0.8383	0.9086	0.9426	0.9368
1.0e-4	0.9316	0.9052	0.9480	0.7931	0.8586	0.9178	0.9155
1.0e-5	0.9021	0.8617	0.9327	0.7686	0.8323	0.8853	0.8782

p-value threshold	BD	CAD	HT	IBD	RA	T1D	T2D
GRS LR							
0.8	0.4581	0.4047	0.3872	0.5534	0.4047	0.3838	0.4061
0.5	0.4561	0.4014	0.3723	0.5453	0.4081	0.3905	0.4000
0.1	0.4561	0.3939	0.4041	0.5000	0.4446	0.4588	0.4223
0.05	0.4885	0.3818	0.4122	0.4851	0.4662	0.4993	0.4453
0.01	0.4757	0.4534	0.4378	0.4858	0.5385	0.5628	0.4405
0.0010	0.4311	0.4615	0.4277	0.5101	0.5507	0.5878	0.4818
1.0e-4	0.4034	0.4088	0.3845	0.4953	0.5115	0.5655	0.4358
1.0e-5	0.3209	0.3122	0.2432	0.4507	0.4743	0.5757	0.3689
wGRS LR							
0.8	0.4608	0.3851	0.3730	0.5757	0.4331	0.4534	0.3946
0.5	0.4588	0.3743	0.3669	0.5507	0.4351	0.4696	0.3905
0.1	0.4588	0.3973	0.3872	0.5020	0.4716	0.5655	0.4149
0.05	0.4757	0.3912	0.4122	0.4926	0.5277	0.5912	0.4324
0.01	0.4736	0.4500	0.4365	0.4973	0.5770	0.6169	0.4453
0.0010	0.4392	0.4764	0.4149	0.5453	0.5919	0.6230	0.4838
1.0e-4	0.3865	0.4284	0.3574	0.5189	0.5520	0.6088	0.4318
1.0e-5	0.3311	0.3608	0.2365	0.4885	0.5230	0.6115	0.4027
aNBC							
0.8	0.4716	0.3973	0.3858	0.5243	0.4412	0.4736	0.4074
0.5	0.4818	0.3905	0.3885	0.5351	0.4473	0.4865	0.4068
0.1	0.4797	0.4020	0.4108	0.5041	0.5108	0.5655	0.4324
0.05	0.4892	0.4061	0.4250	0.5027	0.5439	0.6135	0.4527
0.01	0.4932	0.4723	0.4595	0.5203	0.6149	0.6757	0.4649
0.0010	0.4635	0.5176	0.4682	0.5703	0.6534	0.7074	0.5203
1.0e-4	0.4507	0.5358	0.4520	0.5784	0.6324	0.7074	0.5405
1.0e-5	0.4182	0.5209	0.3216	0.5696	0.6182	0.7182	0.5155
NBC							
0.8	0.3932	0.3101	0.3000	0.4358	0.3811	0.3709	0.3514
0.5	0.4135	0.3291	0.3243	0.4547	0.4007	0.4108	0.3622
0.1	0.4615	0.3804	0.3932	0.4858	0.4926	0.5365	0.4088
0.05	0.4851	0.3892	0.4216	0.5054	0.5277	0.5980	0.4331
0.01	0.4973	0.4723	0.4514	0.5162	0.6135	0.6784	0.4757
0.0010	0.4662	0.5169	0.4622	0.5750	0.6520	0.7108	0.5169
1.0e-4	0.4514	0.5176	0.4581	0.5784	0.6257	0.7027	0.5277
1.0e-5	0.4162	0.4926	0.3318	0.5716	0.6101	0.7182	0.5034

Table S7: Specificity obtained by all the algorithms under the genotype-based approach with 10-fold cross validation. Results for each p-value threshold (column 1) are shown. For those algorithms demanding more computational time (sSVM, AdaBoostM1, C4.5 and 20RF), the maximum p-value threshold used was 0.1.

Table S7 (cont.)

p-value threshold	BD	CAD	HT	IBD	RA	T1D	T2D
			sSVM				
0.1	0.4358	0.3297	0.3541	0.4459	0.4020	0.4223	0.3703
0.05	0.4405	0.3446	0.3736	0.4662	0.4412	0.4682	0.3959
0.01	0.4642	0.3919	0.4149	0.4696	0.4973	0.5291	0.4189
0.0010	0.4385	0.4351	0.4568	0.4831	0.5595	0.6149	0.4324
1.0e-4	0.4196	0.4392	0.4014	0.5243	0.6392	0.6851	0.4162
1.0e-5	0.2595	0.3554	0.2027	0.5047	0.6878	0.7054	0.3162
			AdaBoostM1				
0.1	0.4669	0.4892	0.4318	0.5291	0.6514	0.7054	0.4378
0.05	0.4757	0.4797	0.4412	0.5399	0.6189	0.6797	0.4514
0.01	0.4838	0.4716	0.4257	0.5358	0.6135	0.6676	0.4615
0.0010	0.4919	0.5020	0.4615	0.5345	0.6378	0.6885	0.4473
1.0e-4	0.4500	0.4804	0.4500	0.5507	0.6804	0.7392	0.4527
1.0e-5	0.3608	0.4466	0.3439	0.5439	0.7061	0.7439	0.4047
			C4.5				
0.1	0.4541	0.4669	0.4689	0.4959	0.5595	0.6095	0.4595
0.05	0.4453	0.4345	0.4466	0.4899	0.5736	0.6169	0.4615
0.01	0.4818	0.4831	0.4561	0.5007	0.5770	0.6291	0.4541
0.0010	0.4439	0.4696	0.4743	0.4939	0.5601	0.6757	0.4899
1.0e-4	0.4696	0.4649	0.4608	0.5277	0.5878	0.6845	0.4480
1.0e-5	0.3507	0.4405	0.3574	0.5250	0.6142	0.6777	0.4311
			20RF				
0.1	0.0784	0.0649	0.0473	0.1595	0.0824	0.0419	0.0608
0.05	0.0946	0.0608	0.0514	0.1642	0.0946	0.0405	0.0655
0.01	0.0892	0.0689	0.0541	0.1655	0.1014	0.0676	0.0649
0.0010	0.1061	0.0953	0.0777	0.2095	0.1736	0.1791	0.0777
1.0e-4	0.1311	0.1676	0.1203	0.3068	0.3466	0.3365	0.1324
1.0e-5	0.1784	0.2405	0.1331	0.3899	0.4250	0.4095	0.1953

p-value threshold	BD	CAD	HT	IBD	RA	T1D	T2D
GRS LR							
0.8	0.6121	0.5717	0.5740	0.6038	0.5604	0.5722	0.5856
0.5	0.6143	0.5758	0.5729	0.6058	0.5670	0.5809	0.5790
0.1	0.6054	0.5547	0.5751	0.5906	0.5840	0.6005	0.5820
0.05	0.6065	0.5416	0.5673	0.5774	0.5979	0.6074	0.5764
0.01	0.5734	0.5692	0.5606	0.5721	0.6064	0.6250	0.5670
0.0010	0.5242	0.5793	0.5616	0.5924	0.6211	0.6487	0.5718
1.0e-4	0.5309	0.5825	0.5602	0.6027	0.6213	0.6514	0.5765
1.0e-5	0.5567	0.5712	0.5453	0.5989	0.6231	0.6623	0.5743
wGRS LR							
0.8	0.6193	0.5769	0.5822	0.6096	0.5994	0.6284	0.5911
0.5	0.6194	0.5722	0.5791	0.6097	0.5990	0.6359	0.5919
0.1	0.6137	0.5644	0.5801	0.5901	0.6269	0.6426	0.5902
0.05	0.6030	0.5557	0.5717	0.5845	0.6338	0.6541	0.5784
0.01	0.5736	0.5779	0.5667	0.5908	0.6476	0.6634	0.5812
0.0010	0.5325	0.5963	0.5631	0.6145	0.6589	0.6736	0.5847
1.0e-4	0.5407	0.5988	0.5643	0.6206	0.6479	0.6776	0.5899
1.0e-5	0.5671	0.5878	0.5585	0.6155	0.6417	0.6815	0.5965
aNBC							
0.8	0.6169	0.5759	0.5822	0.6095	0.5990	0.6306	0.5927
0.5	0.6220	0.5713	0.5794	0.6124	0.6014	0.6338	0.5924
0.1	0.6127	0.5617	0.5788	0.5858	0.6310	0.6445	0.5886
0.05	0.6016	0.5580	0.5717	0.5841	0.6352	0.6506	0.5801
0.01	0.5703	0.5820	0.5645	0.5943	0.6519	0.6575	0.5769
0.0010	0.5364	0.5951	0.5615	0.6051	0.6685	0.6550	0.5860
1.0e-4	0.5349	0.5966	0.5623	0.6192	0.6570	0.6503	0.5909
1.0e-5	0.5486	0.5943	0.5488	0.6158	0.6543	0.6650	0.5830
NBC							
0.8	0.6253	0.5941	0.6004	0.5955	0.6216	0.6308	0.6096
0.5	0.6231	0.5832	0.5877	0.5943	0.6144	0.6350	0.5982
0.1	0.6051	0.5681	0.5814	0.5937	0.6351	0.6406	0.5891
0.05	0.6073	0.5602	0.5873	0.5936	0.6391	0.6538	0.5844
0.01	0.5825	0.5829	0.5644	0.5911	0.6566	0.6583	0.5814
0.0010	0.5452	0.5953	0.5637	0.6103	0.6717	0.6539	0.5778
1.0e-4	0.5435	0.5989	0.5677	0.6223	0.6580	0.6462	0.5856
1.0e-5	0.5481	0.5939	0.5541	0.6165	0.6534	0.6592	0.5792

Table S8: Precision obtained by all the algorithms under the genotype-based approach with 10-fold cross validation. Results for each p-value threshold (column 1) are shown. For those algorithms demanding more computational time (sSVM, AdaBoostM1, C4.5 and 20RF), the maximum p-value threshold used was 0.1.

Table S8 (cont.)

p-value threshold	BD	CAD	HT	IBD	RA	T1D	T2D
sSVM							
0.1	0.6352	0.5950	0.6141	0.5975	0.6097	0.6468	0.6134
0.05	0.6335	0.5916	0.6089	0.5982	0.6198	0.6661	0.6122
0.01	0.6192	0.6020	0.6103	0.5927	0.6430	0.6902	0.6063
0.0010	0.5977	0.6180	0.6277	0.5898	0.6749	0.7389	0.6022
1.0e-4	0.5963	0.6329	0.6090	0.6257	0.7245	0.7813	0.6072
1.0e-5	0.5932	0.6200	0.5924	0.6315	0.7527	0.7963	0.6075
AdaBoostM1							
0.1	0.5722	0.5986	0.5525	0.5915	0.6954	0.7777	0.5657
0.05	0.5858	0.5754	0.5528	0.6008	0.6785	0.7669	0.5715
0.01	0.5876	0.5706	0.5548	0.5883	0.6780	0.7603	0.5750
0.0010	0.5747	0.5897	0.5665	0.5726	0.6964	0.7649	0.5575
1.0e-4	0.5501	0.5863	0.5790	0.6162	0.7121	0.8031	0.5741
1.0e-5	0.5669	0.5946	0.5657	0.6312	0.7282	0.8104	0.5910
C4.5							
0.1	0.5722	0.5799	0.5859	0.5673	0.6430	0.7156	0.5881
0.05	0.5615	0.5746	0.5830	0.5680	0.6602	0.7195	0.5814
0.01	0.5784	0.5979	0.5881	0.5745	0.6648	0.7283	0.5870
0.0010	0.5694	0.5928	0.5958	0.5802	0.6636	0.7625	0.5991
1.0e-4	0.5856	0.5975	0.6043	0.6045	0.6813	0.7734	0.6010
1.0e-5	0.5892	0.6199	0.6052	0.6117	0.6943	0.7688	0.6106
20RF							
0.1	0.5591	0.5698	0.5704	0.5393	0.5590	0.5697	0.5682
0.05	0.5635	0.5674	0.5697	0.5424	0.5643	0.5695	0.5672
0.01	0.5613	0.5682	0.5712	0.5453	0.5621	0.5781	0.5682
0.0010	0.5658	0.5766	0.5759	0.5563	0.5803	0.6046	0.5698
1.0e-4	0.5754	0.5865	0.5881	0.5752	0.6229	0.6484	0.5792
1.0e-5	0.5814	0.5967	0.5880	0.5986	0.6452	0.6666	0.5874

p-value threshold	BD	CAD	HT	IBD	RA	T1D	T2D
				GRS LR			
0.8	0.6167	0.5795	0.5834	0.6043	0.5680	0.5826	0.5933
0.5	0.6191	0.5842	0.5840	0.6071	0.5740	0.5907	0.5865
0.1	0.6101	0.5630	0.5840	0.5932	0.5889	0.6067	0.5891
0.05	0.6104	0.5507	0.5753	0.5802	0.6018	0.6110	0.5818
0.01	0.5767	0.5736	0.5663	0.5749	0.6072	0.6249	0.5718
0.0010	0.5272	0.5839	0.5680	0.5944	0.6222	0.6487	0.5736
1.0e-4	0.5373	0.5900	0.5703	0.6053	0.6243	0.6536	0.5830
1.0e-5	0.5675	0.5818	0.5645	0.6015	0.6266	0.6643	0.5836
				wGRS LR			
0.8	0.6236	0.5856	0.5924	0.6080	0.6045	0.6342	0.5982
0.5	0.6233	0.5818	0.5898	0.6105	0.6039	0.6409	0.5991
0.1	0.6188	0.5721	0.5898	0.5926	0.6296	0.6438	0.5968
0.05	0.6078	0.5645	0.5799	0.5870	0.6362	0.6542	0.5845
0.01	0.5767	0.5830	0.5730	0.5935	0.6482	0.6623	0.5862
0.0010	0.5355	0.6006	0.5709	0.6161	0.6599	0.6730	0.5877
1.0e-4	0.5490	0.6047	0.5759	0.6226	0.6500	0.6786	0.5968
1.0e-5	0.5773	0.5959	0.5756	0.6176	0.6443	0.6826	0.6041
				aNBC			
0.8	0.6212	0.5842	0.5919	0.6115	0.6039	0.6359	0.5994
0.5	0.6260	0.5801	0.5892	0.6142	0.6060	0.6388	0.5991
0.1	0.6173	0.5695	0.5875	0.5882	0.6338	0.6461	0.5947
0.05	0.6057	0.5657	0.5791	0.5864	0.6371	0.6478	0.5848
0.01	0.5716	0.5856	0.5683	0.5963	0.6503	0.6467	0.5804
0.0010	0.5373	0.5959	0.5640	0.6050	0.6653	0.6359	0.5856
1.0e-4	0.5370	0.5956	0.5666	0.6195	0.6545	0.6299	0.5889
1.0e-5	0.5543	0.5947	0.5637	0.6164	0.6530	0.6461	0.5821
				NBC			
0.8	0.6263	0.6009	0.6061	0.5985	0.6219	0.6336	0.6135
0.5	0.6257	0.5927	0.5980	0.5975	0.6174	0.6388	0.6041
0.1	0.6101	0.5774	0.5910	0.5963	0.6374	0.6438	0.5962
0.05	0.6116	0.5692	0.5948	0.5957	0.6416	0.6530	0.5906
0.01	0.5848	0.5865	0.5689	0.5932	0.6557	0.6470	0.5848
0.0010	0.5469	0.5962	0.5672	0.6102	0.6689	0.6339	0.5768
1.0e-4	0.5463	0.5997	0.5718	0.6226	0.6563	0.6258	0.5845
1.0e-5	0.5540	0.5965	0.5677	0.6170	0.6527	0.6386	0.5795

Table S9: Overall accuracy obtained by all the algorithms under the genotype-based approach with 10-fold cross validation. Results for each p-value threshold (column 1) are shown. For those algorithms demanding more computational time (sSVM, AdaBoostM1, C4.5 and 20RF), the maximum p-value threshold used was 0.1.

Table S9 (cont.)

p-value threshold	BD	CAD	HT	IBD	RA	T1D	T2D
				sSVM			
0.1	0.6352	0.5950	0.6141	0.5975	0.6097	0.6468	0.6134
0.05	0.6335	0.5916	0.6089	0.5982	0.6198	0.6661	0.6122
0.01	0.6192	0.6020	0.6103	0.5927	0.6430	0.6902	0.6063
0.0010	0.5977	0.6180	0.6277	0.5898	0.6749	0.7389	0.6022
1.0e-4	0.5963	0.6329	0.6090	0.6257	0.7245	0.7813	0.6072
1.0e-5	0.5932	0.6200	0.5924	0.6315	0.7527	0.7963	0.6075
				AdaBoostM1			
0.1	0.5761	0.6026	0.5581	0.5926	0.6952	0.7780	0.5718
0.05	0.5899	0.5786	0.5576	0.6015	0.6793	0.7672	0.5768
0.01	0.5910	0.5739	0.5613	0.5889	0.6787	0.7603	0.5795
0.0010	0.5770	0.5915	0.5703	0.5721	0.6973	0.7655	0.5622
1.0e-4	0.5540	0.5903	0.5849	0.6173	0.7111	0.8029	0.5798
1.0e-5	0.5761	0.6012	0.5782	0.6322	0.7266	0.8101	0.5991
				C4.5			
0.1	0.5233	0.5220	0.5244	0.5300	0.6000	0.6806	0.5340
0.05	0.5099	0.5199	0.5241	0.5319	0.6216	0.6838	0.5249
0.01	0.5275	0.5428	0.5299	0.5384	0.6266	0.6954	0.5340
0.0010	0.5212	0.5390	0.5375	0.5464	0.6317	0.7351	0.5428
1.0e-4	0.5379	0.5466	0.5526	0.5721	0.6509	0.7536	0.5545
1.0e-5	0.5666	0.5877	0.5770	0.5842	0.6596	0.7499	0.5730
				20RF			
0.1	0.5510	0.5657	0.5645	0.5241	0.5518	0.5620	0.5628
0.05	0.5582	0.5610	0.5625	0.5291	0.5614	0.5617	0.5601
0.01	0.5543	0.5616	0.5654	0.5344	0.5557	0.5771	0.5622
0.0010	0.5615	0.5760	0.5721	0.5502	0.5829	0.6151	0.5639
1.0e-4	0.5779	0.5850	0.5919	0.5703	0.6317	0.6684	0.5757
1.0e-5	0.5824	0.5921	0.5887	0.5950	0.6518	0.6812	0.5818

9 Detailed results under the genotype-based approach using holdout

Supplementary Tables S10 to S14 show respectively detailed results of AUC, sensitivity, specificity, precision and overall accuracy returned by all the algorithms used and all p-value thresholds for the 7 diseases when using a holdout approach.

p-value threshold	BD	CAD	HT	IBD	RA	T1D	T2D
GRS LR							
0.15	0.6215	0.5563	0.5661	0.5874	0.5875	0.6109	0.5789
0.1	0.6103	0.5485	0.5672	0.5825	0.5891	0.6155	0.5761
0.05	0.5945	0.5519	0.5561	0.5682	0.6000	0.6243	0.5722
0.01	0.5647	0.5353	0.5458	0.5743	0.6188	0.6567	0.5740
0.001	0.5293	0.5365	0.5468	0.5643	0.6532	0.6929	0.5686
1.0e-4	0.5269	0.5641	0.5431	0.5824	0.6642	0.7060	0.5769
1.0e-5	0.5116	0.6034	0.5251	0.5711	0.6693	0.7046	0.5463
1.0e-6	NA	0.5389	0.5268	0.5642	0.6724	0.7126	0.5786
wGRS LR							
0.15	0.6222	0.5645	0.5725	0.5932	0.6304	0.6630	0.5868
0.1	0.6117	0.5595	0.5728	0.5901	0.6348	0.6678	0.5836
0.05	0.5953	0.5626	0.5617	0.5800	0.6463	0.6789	0.5793
0.01	0.5711	0.5461	0.5581	0.5899	0.6702	0.7062	0.5816
0.001	0.5367	0.5501	0.5569	0.5829	0.6898	0.7223	0.5784
1.0e-4	0.5420	0.5774	0.5539	0.5905	0.6936	0.7245	0.5847
1.0e-5	0.5322	0.6013	0.5395	0.5821	0.6969	0.7239	0.5562
1.0e-6	NA	0.5389	0.5256	0.5721	0.7002	0.7294	0.5764
NBC							
0.15	0.6111	0.5588	0.5706	0.5890	0.6195	0.6449	0.5809
0.1	0.6115	0.5608	0.5742	0.5847	0.6278	0.6528	0.5788
0.05	0.5997	0.5657	0.5707	0.5769	0.6442	0.6653	0.5727
0.01	0.5765	0.5485	0.5602	0.5869	0.6789	0.6955	0.5770
0.001	0.5370	0.5549	0.5610	0.5832	0.7002	0.7141	0.5762
1.0e-4	0.5423	0.5737	0.5516	0.5916	0.7059	0.7201	0.5832
1.0e-5	0.5340	0.6041	0.5375	0.5819	0.7088	0.7205	0.5539
1.0e-6	NA	0.5407	0.5264	0.5705	0.7119	0.7278	0.5782

Table S10: AUC obtained by all the algorithms under the genotype-based approach and holdout sampling. Results for each p-value threshold (column 1) are shown. The maximum p-value threshold used was 0.15.

Table S10 (cont.)

p-value threshold	BD	CAD	HT	IBD	RA	T1D	T2D
			sSVM				
0.15	0.5614	0.5394	0.5400	0.5427	0.5634	0.5791	0.5376
0.1	0.5498	0.5372	0.5395	0.5331	0.5634	0.5883	0.5331
0.05	0.5617	0.5383	0.5300	0.5398	0.5656	0.6026	0.5323
0.01	0.5521	0.5155	0.5372	0.5432	0.5754	0.6425	0.5517
0.001	0.5286	0.5271	0.5255	0.5400	0.6120	0.7190	0.5595
1.0e-4	0.5299	0.5747	0.5362	0.5731	0.6514	0.7695	0.5619
1.0e-5	0.5130	0.5584	0.5168	0.5618	0.7051	0.7857	0.5704
1.0e-6	NA	0.5417	0.5242	0.5707	0.7147	0.7874	0.5829
			AdaBoostM1				
0.15	0.5798	0.5683	0.5776	0.5796	0.7362	0.8163	0.6003
0.1	0.5852	0.5629	0.5685	0.5645	0.7407	0.8192	0.5979
0.05	0.5951	0.5700	0.5524	0.6038	0.7419	0.8056	0.6095
0.01	0.5732	0.5689	0.5741	0.5857	0.7324	0.7746	0.6134
0.001	0.5346	0.5618	0.5532	0.5642	0.7047	0.7626	0.5795
1.0e-4	0.5670	0.5930	0.5647	0.6062	0.7669	0.8354	0.5889
1.0e-5	0.5454	0.6028	0.5413	0.6136	0.8152	0.8615	0.5704
1.0e-6	NA	0.5407	0.5261	0.6081	0.8075	0.8605	0.5873
			C4.5				
0.15	0.5090	0.5021	0.5076	0.5050	0.6072	0.5660	0.5026
0.1	0.4937	0.4951	0.5139	0.5080	0.5943	0.5771	0.4909
0.05	0.4994	0.4934	0.5277	0.5122	0.5587	0.5997	0.5091
0.01	0.4851	0.5287	0.5105	0.5090	0.5794	0.6207	0.5178
0.001	0.5233	0.5411	0.5115	0.5501	0.6169	0.6452	0.5296
1.0e-4	0.5313	0.5186	0.5438	0.5252	0.6771	0.6921	0.5659
1.0e-5	0.5592	0.5727	0.5490	0.5567	0.6913	0.6988	0.5721
1.0e-6	NA	0.5407	0.5259	0.5762	0.7335	0.7278	0.5739
			20RF				
0.15	0.5144	0.5095	0.4991	0.4884	0.5037	0.4965	0.4893
0.1	0.5390	0.4840	0.4925	0.4969	0.4934	0.5043	0.5080
0.05	0.5317	0.5049	0.4874	0.5266	0.5171	0.5094	0.4979
0.01	0.4981	0.4877	0.5175	0.5263	0.5305	0.5349	0.5048
0.001	0.5188	0.5448	0.5287	0.5672	0.6525	0.7015	0.5427
1.0e-4	0.5393	0.5681	0.5318	0.5659	0.7084	0.7713	0.5577
1.0e-5	0.5343	0.6110	0.5396	0.5921	0.7413	0.7889	0.5611
1.0e-6	NA	0.5407	0.5266	0.5906	0.7592	0.8112	0.5843

p-value threshold	BD	CAD	HT	IBD	RA	T1D	T2D
				GRS LR			
0.15	0.7666	0.7601	0.7900	0.6751	0.7462	0.7717	0.7495
0.1	0.7463	0.7414	0.7766	0.6648	0.7516	0.7584	0.7464
0.05	0.7248	0.7051	0.7346	0.6430	0.7161	0.7278	0.7214
0.01	0.6456	0.6615	0.6670	0.6327	0.6925	0.6840	0.7006
0.001	0.6028	0.6137	0.6332	0.6247	0.6839	0.6769	0.6279
1.0e-4	0.6317	0.6937	0.6486	0.6590	0.7323	0.6932	0.6580
1.0e-5	0.6949	0.8754	0.6895	0.6922	0.7495	0.7044	0.6497
1.0e-6	NA	0.8120	0.8176	0.6201	0.7366	0.6911	0.7983
				wGRS LR			
0.15	0.7687	0.7518	0.8043	0.6957	0.7710	0.7819	0.7734
0.1	0.7655	0.7414	0.7838	0.6796	0.7613	0.7655	0.7692
0.05	0.7334	0.7227	0.7449	0.6636	0.7323	0.7166	0.7370
0.01	0.6531	0.6594	0.6783	0.6487	0.7161	0.6993	0.6975
0.001	0.6263	0.6147	0.6557	0.6407	0.7140	0.6942	0.6331
1.0e-4	0.6413	0.6906	0.6537	0.6659	0.7344	0.7085	0.6674
1.0e-5	0.7024	0.8702	0.7736	0.6899	0.7495	0.7136	0.7744
1.0e-6	NA	0.8120	0.8176	0.6327	0.7419	0.7146	0.7983
				NBC			
0.15	0.7859	0.7809	0.8094	0.7185	0.7989	0.7951	0.8046
0.1	0.7709	0.7632	0.8002	0.6865	0.7742	0.7706	0.7838
0.05	0.7495	0.7269	0.7643	0.6796	0.7398	0.7258	0.7380
0.01	0.6456	0.6417	0.6742	0.6362	0.7194	0.6473	0.6726
0.001	0.5953	0.5826	0.6127	0.6144	0.6903	0.5698	0.6143
1.0e-4	0.5610	0.5981	0.6189	0.6098	0.6849	0.5515	0.6424
1.0e-5	0.6692	0.7653	0.7387	0.6041	0.6882	0.5484	0.6975
1.0e-6	NA	0.8266	0.8197	0.5686	0.6882	0.5719	0.7838

Table S 11: Sensitivity obtained by all the algorithms under the genotype-based approach and holdout sampling. Results for each p-value threshold (column 1) are shown. The maximum p-value threshold used was 0.15.

Table S11 (cont.)

p-value threshold	BD	CAD	HT	IBD	RA	T1D	T2D
			sSVM				
0.15	0.7998	0.8100	0.8340	0.7071	0.8065	0.8298	0.7942
0.1	0.7698	0.7799	0.8115	0.6716	0.7903	0.8186	0.7703
0.05	0.7666	0.7549	0.7695	0.6648	0.7731	0.8012	0.7443
0.01	0.7002	0.7040	0.7326	0.6419	0.7387	0.7890	0.7277
0.001	0.6531	0.6231	0.6619	0.6407	0.7172	0.7920	0.6663
1.0e-4	0.6638	0.6656	0.6588	0.6476	0.7151	0.8634	0.6819
1.0e-5	0.7923	0.7830	0.8186	0.7426	0.7763	0.8552	0.8191
1.0e-6	NA	0.8266	0.8186	0.8158	0.7484	0.8614	0.8212
			AdaBoostM1				
0.15	0.6370	0.7092	0.6895	0.6430	0.7301	0.8073	0.6871
0.1	0.6499	0.6750	0.6762	0.6304	0.7333	0.8165	0.6788
0.05	0.6767	0.6978	0.6486	0.6751	0.7538	0.8043	0.6788
0.01	0.6424	0.6874	0.6844	0.6568	0.7333	0.8124	0.7069
0.001	0.6188	0.6251	0.6148	0.6087	0.7054	0.7533	0.6195
1.0e-4	0.6574	0.6532	0.6404	0.5995	0.7161	0.8073	0.6632
1.0e-5	0.6831	0.7664	0.7500	0.6487	0.7505	0.8379	0.7422
1.0e-6	NA	0.8266	0.8197	0.7483	0.7430	0.8338	0.8015
			C4.5				
0.15	0.5546	0.5618	0.5236	0.5503	0.6656	0.6779	0.5738
0.1	0.5567	0.5213	0.5686	0.5343	0.6430	0.6728	0.5738
0.05	0.5610	0.5306	0.5707	0.5767	0.6376	0.6891	0.5676
0.01	0.5289	0.5659	0.5686	0.5629	0.6151	0.7125	0.5624
0.001	0.5878	0.5836	0.5799	0.5526	0.6602	0.7676	0.5956
1.0e-4	0.6306	0.5587	0.6332	0.5789	0.7032	0.7839	0.6175
1.0e-5	0.7559	0.9138	0.7305	0.6533	0.7237	0.8043	0.8659
1.0e-6	NA	0.8266	0.8197	0.8936	0.7473	0.8073	0.7297
			20RF				
0.15	0.8276	0.8775	0.8781	0.7506	0.8344	0.8746	0.8524
0.1	0.8576	0.8463	0.8893	0.7449	0.8043	0.8654	0.8617
0.05	0.8298	0.8692	0.8637	0.7437	0.8312	0.8756	0.8482
0.01	0.8255	0.8671	0.8648	0.7540	0.8161	0.8848	0.8493
0.001	0.8126	0.8390	0.8566	0.7357	0.8161	0.8502	0.8597
1.0e-4	0.7752	0.8069	0.8453	0.7586	0.7903	0.8542	0.8378
1.0e-5	0.7623	0.8930	0.7961	0.7185	0.7978	0.8461	0.7765
1.0e-6	NA	0.8266	0.8197	0.6979	0.7882	0.8451	0.8326

p-value threshold	BD	CAD	HT	IBD	RA	T1D	T2D
				GRS LR			
0.15	0.3932	0.3122	0.3068	0.4230	0.3635	0.3581	0.3473
0.1	0.3838	0.3338	0.3324	0.4432	0.3635	0.3905	0.3378
0.05	0.4135	0.3541	0.3284	0.4324	0.4081	0.4392	0.3824
0.01	0.4324	0.4054	0.3757	0.4730	0.4514	0.5446	0.4189
0.001	0.4257	0.4459	0.4257	0.4527	0.5149	0.6149	0.4784
1.0e-4	0.3932	0.3838	0.4095	0.4541	0.4689	0.6189	0.4730
1.0e-5	0.2986	0.2716	0.3405	0.3986	0.4527	0.6081	0.4027
1.0e-6	NA	0.2676	0.2324	0.4541	0.4595	0.6108	0.3027
				wGRS LR			
0.15	0.3959	0.3189	0.3000	0.4297	0.4122	0.4419	0.3351
0.1	0.3824	0.3351	0.3162	0.4459	0.4203	0.4905	0.3270
0.05	0.4054	0.3662	0.3162	0.4257	0.4405	0.5459	0.3811
0.01	0.4392	0.4081	0.3824	0.4824	0.5135	0.6135	0.4189
0.001	0.4135	0.4554	0.4257	0.4635	0.5338	0.6459	0.4716
1.0e-4	0.4054	0.4000	0.4311	0.4743	0.5081	0.6284	0.4541
1.0e-5	0.3257	0.2919	0.2608	0.4081	0.5000	0.6297	0.2946
1.0e-6	NA	0.2676	0.2324	0.4419	0.4959	0.6270	0.3027
				NBC			
0.15	0.3635	0.2946	0.2784	0.3973	0.3797	0.4230	0.3243
0.1	0.3622	0.3108	0.3068	0.4216	0.4000	0.4689	0.3351
0.05	0.3905	0.3608	0.3365	0.4351	0.4378	0.5270	0.3676
0.01	0.4500	0.4243	0.3892	0.4865	0.5351	0.6459	0.4446
0.001	0.4568	0.5108	0.4568	0.5027	0.5865	0.7243	0.5000
1.0e-4	0.4743	0.4946	0.4676	0.5243	0.5865	0.7257	0.4676
1.0e-5	0.3514	0.3716	0.3027	0.5081	0.5865	0.7311	0.3824
1.0e-6	NA	0.2568	0.2311	0.5027	0.5919	0.7311	0.3149

Table S 12: Specificity obtained by all the algorithms under the genotype-based approach and holdout sampling. Results for each p-value threshold (column 1) are shown. The maximum p-value threshold used was 0.15.

Table S12 (cont.)

p-value threshold	BD	CAD	HT	IBD	RA	T1D	T2D
			sSVM				
0.15	0.3230	0.2689	0.2459	0.3784	0.3203	0.3284	0.2811
0.1	0.3297	0.2946	0.2676	0.3946	0.3365	0.3581	0.2959
0.05	0.3568	0.3216	0.2905	0.4149	0.3581	0.4041	0.3203
0.01	0.4041	0.3270	0.3419	0.4446	0.4122	0.4959	0.3757
0.001	0.4041	0.4311	0.3892	0.4392	0.5068	0.6459	0.4527
1.0e-4	0.3959	0.4838	0.4135	0.4986	0.5878	0.6757	0.4419
1.0e-5	0.2338	0.3338	0.2149	0.3811	0.6338	0.7162	0.3216
1.0e-6	NA	0.2568	0.2297	0.3257	0.6811	0.7135	0.3446
			AdaBoostM1				
0.15	0.4581	0.4135	0.4014	0.4811	0.5973	0.6622	0.4392
0.1	0.4378	0.4149	0.4068	0.4446	0.5959	0.6635	0.4595
0.05	0.4622	0.4162	0.4203	0.4784	0.5892	0.6514	0.4797
0.01	0.4595	0.4054	0.4311	0.4649	0.5757	0.6014	0.4568
0.001	0.4365	0.4716	0.4622	0.4703	0.5635	0.6351	0.4986
1.0e-4	0.4473	0.5000	0.4270	0.5595	0.6459	0.7162	0.4595
1.0e-5	0.3432	0.3716	0.2784	0.4838	0.7000	0.7216	0.3716
1.0e-6	NA	0.2568	0.2284	0.4068	0.6892	0.7176	0.3000
			C4.5				
0.15	0.4500	0.4405	0.4595	0.4405	0.5176	0.5365	0.4459
0.1	0.4486	0.4730	0.4689	0.4662	0.5257	0.5514	0.4338
0.05	0.4270	0.4419	0.4514	0.4541	0.5405	0.5770	0.4554
0.01	0.4486	0.4703	0.4500	0.4541	0.5568	0.5838	0.4824
0.001	0.4459	0.4932	0.4581	0.5108	0.6081	0.6108	0.4514
1.0e-4	0.4108	0.4905	0.4338	0.4649	0.6405	0.6919	0.5000
1.0e-5	0.2905	0.2216	0.3284	0.4649	0.6500	0.6689	0.2568
1.0e-6	NA	0.2568	0.2311	0.2405	0.6878	0.6649	0.4149
			20RF				
0.15	0.1689	0.1405	0.1081	0.2378	0.1716	0.1135	0.1257
0.1	0.1703	0.1446	0.1216	0.2797	0.1973	0.1270	0.1392
0.05	0.2108	0.1324	0.1041	0.2838	0.1811	0.1338	0.1595
0.01	0.1635	0.1284	0.1500	0.2703	0.2189	0.1405	0.1378
0.001	0.2230	0.1770	0.1703	0.3473	0.3689	0.3797	0.1797
1.0e-4	0.2689	0.2757	0.1838	0.3446	0.4595	0.5365	0.2378
1.0e-5	0.2689	0.2716	0.2405	0.3959	0.5081	0.5500	0.2946
1.0e-6	NA	0.2568	0.2311	0.4311	0.5324	0.6027	0.2730

p-value threshold	BD	CAD	HT	IBD	RA	T1D	T2D
			GRS LR				
0.15	0.5956	0.5508	0.5681	0.5545	0.5678	0.5833	0.5628
0.1	0.5782	0.5509	0.5730	0.5590	0.5711	0.5911	0.5560
0.05	0.5803	0.5404	0.5447	0.5421	0.5724	0.5965	0.5641
0.01	0.5462	0.5427	0.5315	0.5567	0.5803	0.6225	0.5705
0.001	0.5209	0.5381	0.5388	0.5426	0.6061	0.6523	0.5610
1.0e-4	0.5194	0.5492	0.5387	0.5613	0.6111	0.6624	0.5739
1.0e-5	0.5031	0.6170	0.5255	0.5518	0.6136	0.6630	0.5351
1.0e-6	NA	0.5610	0.5442	0.5409	0.6091	0.6575	0.5711
			wGRS LR				
0.15	0.5983	0.5493	0.5745	0.5693	0.6075	0.6304	0.5716
0.1	0.5896	0.5516	0.5691	0.5684	0.6053	0.6427	0.5646
0.05	0.5813	0.5567	0.5443	0.5497	0.5976	0.6404	0.5726
0.01	0.5534	0.5429	0.5409	0.5697	0.6230	0.6629	0.5687
0.001	0.5268	0.5433	0.5505	0.5562	0.6313	0.6758	0.5604
1.0e-4	0.5305	0.5556	0.5520	0.5749	0.6307	0.6746	0.5696
1.0e-5	0.5222	0.6232	0.5308	0.5553	0.6357	0.6779	0.5502
1.0e-6	NA	0.5610	0.5442	0.5414	0.6294	0.6772	0.5711
			NBC				
0.15	0.5934	0.5546	0.5660	0.5663	0.6103	0.6302	0.5872
0.1	0.5828	0.5520	0.5752	0.5602	0.6037	0.6359	0.5785
0.05	0.5836	0.5564	0.5672	0.5631	0.6007	0.6366	0.5664
0.01	0.5548	0.5418	0.5421	0.5651	0.6349	0.6530	0.5678
0.001	0.5325	0.5543	0.5437	0.5619	0.6435	0.6582	0.5647
1.0e-4	0.5241	0.5541	0.5520	0.5702	0.6407	0.6508	0.5632
1.0e-5	0.5176	0.5852	0.5332	0.5593	0.6424	0.6525	0.5506
1.0e-6	NA	0.5657	0.5449	0.5388	0.6450	0.6628	0.5677

Table S13: Precision obtained by all the algorithms under the genotype-based approach and holdout sampling. Results for each p-value threshold (column 1) are shown. The maximum p-value threshold used was 0.15.

Table S13 (cont.)

p-value threshold	BD	CAD	HT	IBD	RA	T1D	T2D
			sSVM				
0.15	0.5820	0.5603	0.5656	0.5499	0.5852	0.6088	0.5559
0.1	0.5652	0.5539	0.5612	0.5383	0.5824	0.6154	0.5483
0.05	0.5773	0.5527	0.5455	0.5449	0.5820	0.6254	0.5453
0.01	0.5615	0.5255	0.5506	0.5475	0.5876	0.6596	0.5643
0.001	0.5360	0.5356	0.5356	0.5442	0.6204	0.7276	0.5684
1.0e-4	0.5376	0.5831	0.5461	0.5770	0.6571	0.7833	0.5715
1.0e-5	0.5243	0.5773	0.5334	0.5725	0.7119	0.7950	0.5964
1.0e-6	NA	0.5657	0.5432	0.5934	0.7185	0.7976	0.6099
			AdaBoostM1				
0.15	0.5543	0.5727	0.5565	0.5660	0.6696	0.7436	0.5729
0.1	0.5511	0.5545	0.5519	0.5415	0.6708	0.7495	0.5783
0.05	0.5771	0.5676	0.5441	0.5817	0.6790	0.7370	0.5880
0.01	0.5578	0.5566	0.5683	0.5653	0.6614	0.7202	0.5922
0.001	0.5344	0.5564	0.5474	0.5430	0.6406	0.7013	0.5666
1.0e-4	0.5596	0.5844	0.5432	0.5823	0.6851	0.7675	0.5700
1.0e-5	0.5208	0.5859	0.5263	0.5704	0.7286	0.7871	0.5709
1.0e-6	NA	0.5657	0.5431	0.5889	0.7195	0.7830	0.5718
			C4.5				
0.15	0.5090	0.5097	0.5012	0.4989	0.5977	0.6155	0.5182
0.1	0.5094	0.5058	0.5278	0.5037	0.5900	0.6199	0.5122
0.05	0.5008	0.4951	0.5203	0.5188	0.5944	0.6404	0.5198
0.01	0.4957	0.5263	0.5186	0.5119	0.5910	0.6558	0.5304
0.001	0.5234	0.5462	0.5281	0.5349	0.6386	0.6981	0.5317
1.0e-4	0.5277	0.5327	0.5428	0.5253	0.6759	0.7437	0.5663
1.0e-5	0.5345	0.6303	0.5422	0.5635	0.6909	0.7448	0.5994
1.0e-6	NA	0.5657	0.5449	0.6161	0.7211	0.7447	0.5850
			20RF				
0.15	0.5039	0.5262	0.4947	0.4960	0.5114	0.4975	0.4880
0.1	0.5309	0.5006	0.5212	0.5185	0.5076	0.5026	0.5094
0.05	0.5371	0.5101	0.4765	0.5201	0.5162	0.5190	0.5151
0.01	0.4978	0.5041	0.5229	0.5186	0.5317	0.5350	0.4969
0.001	0.5320	0.5215	0.5322	0.5507	0.6172	0.6500	0.5417
1.0e-4	0.5344	0.5619	0.5327	0.5636	0.6422	0.7206	0.5630
1.0e-5	0.5260	0.6349	0.5337	0.5656	0.6690	0.7204	0.5516
1.0e-6	NA	0.5657	0.5449	0.5713	0.6737	0.7415	0.5801

p-value threshold	BD	CAD	HT	IBD	RA	T1D	T2D
				GRS LR			
0.15	0.6016	0.5655	0.5816	0.5595	0.5766	0.5938	0.5746
0.1	0.5860	0.5643	0.5851	0.5632	0.5796	0.6002	0.5687
0.05	0.5872	0.5526	0.5594	0.5465	0.5796	0.6037	0.5740
0.01	0.5514	0.5502	0.5414	0.5595	0.5856	0.6241	0.5781
0.001	0.5245	0.5408	0.5437	0.5458	0.6090	0.6502	0.5629
1.0e-4	0.5263	0.5590	0.5455	0.5651	0.6156	0.6612	0.5776
1.0e-5	0.5197	0.6130	0.5390	0.5576	0.6180	0.6630	0.5423
1.0e-6	NA	0.5755	0.5653	0.5440	0.6138	0.6566	0.5828
				wGRS LR			
0.15	0.6039	0.5637	0.5868	0.5737	0.6120	0.6357	0.5828
0.1	0.5962	0.5649	0.5822	0.5725	0.6102	0.6473	0.5770
0.05	0.5884	0.5678	0.5600	0.5545	0.6030	0.6432	0.5823
0.01	0.5585	0.5502	0.5507	0.5725	0.6263	0.6624	0.5764
0.001	0.5323	0.5455	0.5565	0.5595	0.6341	0.6734	0.5629
1.0e-4	0.5370	0.5643	0.5577	0.5781	0.6341	0.6740	0.5746
1.0e-5	0.5358	0.6189	0.5524	0.5607	0.6389	0.6775	0.5658
1.0e-6	NA	0.5755	0.5653	0.5452	0.6329	0.6769	0.5828
				NBC			
0.15	0.5992	0.5696	0.5804	0.5713	0.6132	0.6351	0.5958
0.1	0.5902	0.5666	0.5874	0.5651	0.6084	0.6409	0.5887
0.05	0.5908	0.5678	0.5798	0.5675	0.6060	0.6403	0.5770
0.01	0.5591	0.5473	0.5513	0.5675	0.6377	0.6467	0.5734
0.001	0.5341	0.5514	0.5455	0.5632	0.6443	0.6363	0.5646
1.0e-4	0.5227	0.5531	0.5536	0.5706	0.6413	0.6264	0.5664
1.0e-5	0.5287	0.5942	0.5507	0.5601	0.6431	0.6270	0.5605
1.0e-6	NA	0.5790	0.5659	0.5384	0.6455	0.6403	0.5799

Table S14: Overall accuracy obtained by all the algorithms under the genotype-based approach and holdout sampling. Results for each p-value threshold (column 1) are shown. The maximum p-value threshold used was 0.15.

Table S14 (cont.)

p-value threshold	BD	CAD	HT	IBD	RA	T1D	T2D
			sSVM				
0.15	0.5890	0.5749	0.5804	0.5564	0.5910	0.6142	0.5711
0.1	0.5753	0.5690	0.5769	0.5446	0.5892	0.6206	0.5640
0.05	0.5854	0.5666	0.5629	0.5502	0.5892	0.6304	0.5599
0.01	0.5693	0.5402	0.5641	0.5514	0.5940	0.6630	0.5746
0.001	0.5430	0.5396	0.5443	0.5483	0.6240	0.7292	0.5734
1.0e-4	0.5454	0.5866	0.5530	0.5793	0.6587	0.7827	0.5776
1.0e-5	0.5454	0.5878	0.5583	0.5768	0.7132	0.7955	0.6028
1.0e-6	NA	0.5790	0.5647	0.5911	0.7186	0.7978	0.6140
			AdaBoostM1				
0.15	0.5579	0.5807	0.5653	0.5688	0.6713	0.7449	0.5793
0.1	0.5562	0.5620	0.5600	0.5452	0.6725	0.7507	0.5834
0.05	0.5818	0.5755	0.5501	0.5849	0.6808	0.7385	0.5922
0.01	0.5615	0.5649	0.5752	0.5688	0.6635	0.7217	0.5981
0.001	0.5382	0.5584	0.5490	0.5452	0.6425	0.7025	0.5670
1.0e-4	0.5645	0.5866	0.5484	0.5812	0.6850	0.7682	0.5746
1.0e-5	0.5329	0.5948	0.5466	0.5731	0.7281	0.7879	0.5811
1.0e-6	NA	0.5790	0.5647	0.5917	0.7192	0.7838	0.5834
			C4.5				
0.15	0.5084	0.5091	0.4959	0.5000	0.6000	0.6171	0.5182
0.1	0.5090	0.5003	0.5256	0.5031	0.5910	0.6206	0.5129
0.05	0.5018	0.4921	0.5192	0.5204	0.5946	0.6409	0.5188
0.01	0.4934	0.5244	0.5175	0.5130	0.5892	0.6572	0.5276
0.001	0.5251	0.5443	0.5274	0.5335	0.6371	0.7002	0.5329
1.0e-4	0.5335	0.5291	0.5472	0.5266	0.6754	0.7443	0.5664
1.0e-5	0.5502	0.6130	0.5571	0.5669	0.6910	0.7461	0.6011
1.0e-6	NA	0.5790	0.5659	0.5942	0.7210	0.7461	0.5928
			20RF				
0.15	0.5364	0.5573	0.5460	0.5155	0.5407	0.5474	0.5364
0.1	0.5538	0.5414	0.5583	0.5316	0.5353	0.5479	0.5476
0.05	0.5562	0.5490	0.5361	0.5328	0.5431	0.5567	0.5488
0.01	0.5329	0.5461	0.5565	0.5322	0.5515	0.5648	0.5400
0.001	0.5520	0.5514	0.5606	0.5576	0.6180	0.6479	0.5640
1.0e-4	0.5514	0.5760	0.5600	0.5688	0.6437	0.7176	0.5770
1.0e-5	0.5442	0.6230	0.5565	0.5706	0.6695	0.7188	0.5670
1.0e-6	NA	0.5790	0.5659	0.5756	0.6749	0.7408	0.5893

10 Detailed results under the haplotype-based approach using holdout

Supplementary Tables S15 to S17 show respectively detailed results of AUC under the additive, dominant and recessive genetic models returned by all the algorithms used and all p-value thresholds for the 7 diseases when using a holdout approach and haplotypes of size 1.

Equivalently, for haplotypes of size 2, 3, 4 and 5, Supplementary Tables S18 to S20, S21 to S23, S24 to S26 and S27 to S29 show results of AUC under the additive, dominant and recessive genetic models returned by all the algorithms used and all p-value thresholds for the 7 diseases when using a holdout approach and haplotypes of sizes $2, 3, 4$ and 5.

In the same manner, results of sensitivity, specificity, precision and overall accuracy are respectively shown in Tables S30 to S44, S45 to S59, S60 to S74 and S75 to S89.

p-value threshold	BD	CAD	HT	IBD	RA	T1D	T2D
				NBC			
0.15	0.5557	0.5000	0.5000	0.5578	0.5807	0.6192	0.5475
0.1	0.5540	0.5000	0.5000	0.5521	0.6000	0.6399	0.5464
0.05	0.5612	0.5000	0.5000	0.5511	0.6094	0.6586	0.5453
0.01	0.5595	0.5015	0.5162	0.5494	0.6602	0.6928	0.5235
0.0010	0.5232	0.5207	0.5083	0.5357	0.6917	0.7125	0.5207
1.0e-4	0.5353	0.5003	0.4940	0.5354	0.7079	0.7261	0.5378
1.0e-5	0.5298	0.5005	0.5158	0.5383	0.7055	0.7349	0.5530
1.0e-6	NA	0.4941	NA	NA	0.6954	0.7374	0.5338
1.0e-7	NA	0.4995	NA	NA	0.6823	0.7515	NA
				sSVM			
0.15	0.5224	0.5000	0.5000	0.5292	0.5135	0.5384	0.5193
0.1	0.5353	0.5000	0.5000	0.5301	0.5282	0.5562	0.5241
0.05	0.5344	0.5000	0.5000	0.5183	0.5293	0.5635	0.5157
0.01	0.5289	0.5017	0.5014	0.5118	0.5430	0.6006	0.5096
0.0010	0.5127	0.5103	0.5100	0.5147	0.5726	0.6791	0.5143
1.0e-4	0.5078	0.4994	0.4967	0.5070	0.6250	0.7156	0.5259
1.0e-5	0.5079	0.5113	0.5000	0.5223	0.6405	0.7236	0.5246
1.0e-6	NA	0.5154	NA	NA	0.6366	0.7226	0.5159
1.0e-7	NA	0.5072	NA	NA	0.6331	0.7188	NA
				AdaBoostM1			
0.15	0.5570	0.5216	0.5107	0.5084	0.6915	0.7687	0.5429
0.1	0.5637	0.5188	0.4896	0.5272	0.6974	0.7718	0.5335
0.05	0.5631	0.5156	0.4947	0.5343	0.6714	0.7576	0.5272
0.01	0.5610	0.5071	0.5129	0.5483	0.6439	0.7209	0.5292
0.0010	0.5358	0.5150	0.5054	0.5346	0.7125	0.7512	0.5272
1.0e-4	0.5354	0.5040	0.4914	0.5349	0.7631	0.8226	0.5513
1.0e-5	0.5301	0.4958	0.5157	0.5422	0.7818	0.8313	0.5721
1.0e-6	NA	0.5098	NA	NA	0.7647	0.8399	0.5338
1.0e-7	NA	0.4993	NA	NA	0.7631	0.8403	NA
				C4.5			
0.15	0.5050	0.5119	0.5032	0.5027	0.5579	0.5069	0.5050
0.1	0.5028	0.5079	0.4965	0.4817	0.5791	0.5080	0.5218
0.05	0.5021	0.5037	0.5065	0.5013	0.5761	0.5119	0.5130
0.01	0.5264	0.5052	0.4935	0.5220	0.6019	0.5576	0.5107
0.0010	0.5326	0.4890	0.5051	0.5076	0.5724	0.5528	0.5273
1.0e-4	0.5260	0.4862	0.4903	0.5204	0.6002	0.6155	0.5468
1.0e-5	0.5211	0.4668	0.4902	0.5350	0.6607	0.6079	0.5579
1.0e-6	NA	0.5309	NA	NA	0.6237	0.6271	0.5383
1.0e-7	NA	0.5168	NA	NA	0.6250	0.6385	NA
				20RF			
0.15	0.5078	0.4883	0.5061	0.5106	0.4928	0.5041	0.5088
0.1	0.5106	0.5264	0.4933	0.4776	0.5541	0.5163	0.4985
0.05	0.5224	0.5184	0.4929	0.5168	0.5197	0.5715	0.5141
0.01	0.5005	0.4859	0.4714	0.4743	0.5688	0.6070	0.5228
0.0010	0.5105	0.5069	0.5037	0.5329	0.6590	0.6991	0.5232
1.0e-4	0.5304	0.4781	0.4730	0.5206	0.7227	0.7883	0.5346
1.0e-5	0.5327	0.4720	0.5268	0.5425	0.7321	0.7819	0.5639
1.0e-6	NA	0.4985	NA	NA	0.7306	0.7832	0.5325
1.0e-7	NA	0.4983	NA	NA	0.7393	0.8033	NA

Table S15: AUC obtained by all the algorithms under the haplotype-based approach with 1-SNP haplotype length, additive genetic model and holdout sampling. Results for each p-value threshold (column 1) are shown. The maximum p-value threshold used was 0.15.

p-value threshold	BD	CAD	HT	IBD	RA	T1D	T2D
				NBC			
0.15	0.5531	0.5000	0.5000	0.5480	0.5806	0.6182	0.5447
0.1	0.5503	0.5000	0.5000	0.5466	0.5979	0.6386	0.5411
0.05	0.5563	0.5000	0.5114	0.5276	0.6045	0.6628	0.5415
0.01	0.5512	0.5013	0.5160	0.5329	0.6537	0.6874	0.5264
0.0010	0.5213	0.5151	0.5086	0.5354	0.7066	0.7040	0.5149
1.0e-4	0.5310	0.5031	0.4933	0.5348	0.7182	0.7139	0.5292
1.0e-5	0.5333	0.5017	0.5157	0.5373	0.7125	0.7211	0.5453
1.0e-6	NA	0.4944	NA	NA	0.7041	0.7232	0.5209
1.0e-7	NA	0.4997	NA	NA	0.6933	0.7371	NA
				sSVM			
0.15	0.5081	0.5000	0.5000	0.5257	0.5029	0.5181	0.5118
0.1	0.5161	0.5000	0.5000	0.5301	0.5111	0.5416	0.5153
0.05	0.5223	0.5000	0.5000	0.5165	0.5169	0.5532	0.5108
0.01	0.5225	0.5013	0.5018	0.5183	0.5314	0.5810	0.5111
0.0010	0.5170	0.5058	0.5084	0.5062	0.5498	0.6645	0.5038
1.0e-4	0.4971	0.5078	0.4994	0.4966	0.5975	0.6971	0.5194
1.0e-5	0.5023	0.5088	0.4902	0.5194	0.6237	0.7112	0.5162
1.0e-6	NA	0.5038	NA	NA	0.6226	0.7072	0.4997
1.0e-7	NA	0.4995	NA	NA	0.6228	0.7026	NA
				AdaBoostM1			
0.15	0.5414	0.5168	0.5111	0.5074	0.6912	0.7558	0.5475
0.1	0.5598	0.5154	0.4924	0.5224	0.6947	0.7604	0.5283
0.05	0.5574	0.5169	0.4856	0.5422	0.6737	0.7416	0.5252
0.01	0.5495	0.4999	0.5078	0.5365	0.6397	0.7059	0.5336
0.0010	0.5300	0.5100	0.5033	0.5397	0.7053	0.7310	0.5234
1.0e-4	0.5306	0.5086	0.4920	0.5323	0.7627	0.8174	0.5429
1.0e-5	0.5335	0.4981	0.5157	0.5413	0.7823	0.8274	0.5627
1.0e-6	NA	0.5097	NA	NA	0.7642	0.8342	0.5209
1.0e-7	NA	0.4989	NA	NA	0.7659	0.8330	NA
				C4.5			
0.15	0.5014	0.5129	0.5007	0.4967	0.5687	0.5254	0.5242
0.1	0.5027	0.5057	0.5023	0.4847	0.5924	0.5049	0.5191
0.05	0.5143	0.5015	0.5110	0.4957	0.5924	0.5357	0.5221
0.01	0.5337	0.5003	0.4926	0.5296	0.6078	0.5779	0.5164
0.0010	0.5152	0.4881	0.5079	0.5067	0.5679	0.5845	0.5055
1.0e-4	0.5300	0.4841	0.4903	0.5172	0.5980	0.6317	0.5390
1.0e-5	0.5211	0.4673	0.4902	0.5360	0.6731	0.6317	0.5548
1.0e-6	NA	0.5309	NA	NA	0.6450	0.6508	0.5383
1.0e-7	NA	0.5168	NA	NA	0.6493	0.6653	NA
				20RF			
0.15	0.5076	0.4886	0.5048	0.5103	0.4923	0.5037	0.5087
0.1	0.5105	0.5264	0.4932	0.4774	0.5540	0.5152	0.4987
0.05	0.5224	0.5168	0.4916	0.5178	0.5198	0.5724	0.5137
0.01	0.5005	0.4867	0.4709	0.4754	0.5704	0.6066	0.5235
0.0010	0.5117	0.5086	0.5055	0.5331	0.6603	0.6984	0.5224
1.0e-4	0.5303	0.4790	0.4738	0.5214	0.7241	0.7835	0.5306
1.0e-5	0.5326	0.4683	0.5158	0.5417	0.7323	0.7774	0.5575
1.0e-6	NA	0.4974	NA	NA	0.7321	0.7781	0.5209
1.0e-7	NA	0.4983	NA	NA	0.7411	0.8000	NA

Table S16: AUC obtained by all the algorithms under the haplotype-based approach with 1-SNP haplotype length, dominant genetic model and holdout sampling. Results for each p-value threshold (column 1) are shown. The maximum p-value threshold used was 0.15.

p-value threshold	BD	CAD	HT	IBD	RA	T1D	T2D
				NBC			
0.15	0.6053	0.5000	0.5000	0.5608	0.6046	0.6128	0.5480
0.1	0.5977	0.5000	0.5000	0.5550	0.6154	0.6194	0.5494
0.05	0.5780	0.5000	0.5000	0.5574	0.6234	0.6358	0.5516
0.01	0.5585	0.5015	0.5162	0.5501	0.6531	0.6888	0.5161
0.0010	0.5216	0.5207	0.5083	0.5328	0.6719	0.7289	0.5244
1.0e-4	0.5336	0.5002	0.4944	0.5348	0.6853	0.7422	0.5462
1.0e-5	0.5299	0.5006	0.5158	0.5387	0.6833	0.7511	0.5608
1.0e-6	NA	0.4941	NA	NA	0.6730	0.7532	0.5358
1.0e-7	NA	0.4993	NA	NA	0.6604	0.7610	NA
				sSVM			
0.15	0.5420	0.5000	0.5000	0.5335	0.5278	0.5695	0.5296
0.1	0.5631	0.5000	0.5000	0.5303	0.5524	0.5778	0.5364
0.05	0.5512	0.5000	0.5000	0.5204	0.5464	0.5783	0.5222
0.01	0.5374	0.5022	0.5007	0.5044	0.5591	0.6312	0.5077
0.0010	0.5074	0.5166	0.5122	0.5255	0.6075	0.7007	0.5293
1.0e-4	0.5226	0.4892	0.4933	0.5203	0.6683	0.7438	0.5347
1.0e-5	0.5152	0.5146	0.5000	0.5257	0.6642	0.7414	0.5364
1.0e-6	NA	0.5316	NA	NA	0.6561	0.7453	0.5397
1.0e-7	NA	0.5177	NA	NA	0.6469	0.7428	NA
				AdaBoostM1			
0.15	0.5604	0.5218	0.5140	0.5101	0.6668	0.7475	0.5292
0.1	0.5556	0.5253	0.4907	0.5254	0.6704	0.7514	0.5282
0.05	0.5632	0.5152	0.5004	0.5249	0.6477	0.7353	0.5319
0.01	0.5612	0.5111	0.5113	0.5468	0.6304	0.7044	0.5177
0.0010	0.5328	0.5167	0.5071	0.5202	0.6939	0.7430	0.5269
1.0e-4	0.5385	0.5010	0.4919	0.5381	0.7528	0.8153	0.5602
1.0e-5	0.5299	0.4967	0.5158	0.5412	0.7767	0.8271	0.5763
1.0e-6	NA	0.5098	NA	NA	0.7592	0.8379	0.5358
1.0e-7	NA	0.4993	NA	NA	0.7527	0.8414	NA
				C4.5			
0.15	0.5091	0.5102	0.5065	0.5102	0.5523	0.5545	0.4869
0.1	0.5101	0.5106	0.4893	0.4767	0.5873	0.5688	0.5281
0.05	0.5085	0.5068	0.5007	0.5082	0.5762	0.5739	0.5000
0.01	0.5267	0.5118	0.4947	0.5153	0.6240	0.6048	0.5060
0.0010	0.5569	0.4974	0.5038	0.5013	0.6158	0.6055	0.5441
1.0e-4	0.5270	0.4897	0.4903	0.5197	0.6566	0.6645	0.5514
1.0e-5	0.5211	0.4683	0.4902	0.5350	0.6872	0.6619	0.5593
1.0e-6	NA	0.5282	NA	NA	0.6489	0.6838	0.5383
1.0e-7	NA	0.5168	NA	NA	0.6542	0.7057	NA
				20RF			
0.15	0.5082	0.4884	0.5073	0.5111	0.4938	0.5044	0.5090
0.1	0.5106	0.5263	0.4932	0.4776	0.5539	0.5175	0.4983
0.05	0.5225	0.5201	0.4942	0.5153	0.5191	0.5701	0.5147
0.01	0.5007	0.4853	0.4723	0.4734	0.5668	0.6067	0.5222
0.0010	0.5092	0.5047	0.5020	0.5327	0.6571	0.6998	0.5242
1.0e-4	0.5301	0.4765	0.4721	0.5195	0.7204	0.7932	0.5402
1.0e-5	0.5324	0.4757	0.5268	0.5422	0.7304	0.7861	0.5737
1.0e-6	NA	0.4985	NA	NA	0.7268	0.7877	0.5352
1.0e-7	NA	0.4983	NA	NA	0.7344	0.8053	NA

Table S17: AUC obtained by all the algorithms under the haplotype-based approach with 1-SNP haplotype length, recessive genetic model and holdout sampling. Results for each p-value threshold (column 1) are shown. The maximum p-value threshold used was 0.15.

p-value threshold	BD	CAD	HT	IBD	RA	T1D	T2D
				NBC			
0.15	0.5897	0.5000	0.5000	0.5717	0.6184	0.6514	0.5838
0.1	0.6120	0.5000	0.5000	0.5726	0.6392	0.6647	0.5968
0.05	0.6341	0.4974	0.5230	0.5494	0.6643	0.6941	0.6007
0.01	0.6540	0.4968	0.5166	0.5371	0.7086	0.7195	0.6030
0.0010	0.6606	0.5287	0.5384	0.5568	0.7318	0.7429	0.6183
1.0e-4	0.6595	0.5068	0.5203	0.5912	0.7288	0.7482	0.6299
1.0e-5	0.6593	0.4846	0.5305	0.6125	0.7211	0.7493	0.5600
1.0e-6	0.6230	0.5014	0.5076	0.5535	0.7104	0.7486	0.5196
1.0e-7	0.6019	0.4677	NA	0.5347	0.7010	0.7531	NA
				sSVM			
0.15	0.5284	0.5000	0.5000	0.5154	0.5203	0.5414	0.5233
0.1	0.5344	0.5000	0.5000	0.5331	0.5289	0.5527	0.5259
0.05	0.5325	0.5000	0.5000	0.5162	0.5277	0.5626	0.5275
0.01	0.5324	0.5058	0.5008	0.4979	0.5459	0.5986	0.5142
0.0010	0.5344	0.5111	0.5015	0.5249	0.5849	0.6666	0.5214
1.0e-4	0.5461	0.5000	0.5068	0.5169	0.6271	0.7196	0.5429
1.0e-5	0.5597	0.4772	0.5000	0.5460	0.6458	0.7339	0.5327
1.0e-6	0.5550	0.5036	0.5000	0.5096	0.6428	0.7326	0.5000
1.0e-7	0.5285	0.4772	NA	0.5000	0.6395	0.7288	NA
				AdaBoostM1			
0.15	0.6211	0.5172	0.4947	0.5735	0.7124	0.7941	0.5827
0.1	0.6411	0.4939	0.4936	0.5505	0.7118	0.7768	0.5707
0.05	0.6328	0.5250	0.4999	0.5457	0.7086	0.7775	0.5610
0.01	0.6296	0.5232	0.5050	0.5434	0.6866	0.7522	0.5882
0.0010	0.6866	0.5056	0.5331	0.5854	0.7658	0.8104	0.6252
1.0e-4	0.6746	0.5126	0.5219	0.6041	0.8020	0.8567	0.6372
1.0e-5	0.6509	0.4710	0.5155	0.6192	0.8024	0.8522	0.5681
1.0e-6	0.6237	0.5115	0.5076	0.5546	0.7827	0.8550	0.5204
1.0e-7	0.6019	0.4677	NA	0.5347	0.7783	0.8506	NA
				C4.5			
0.15	0.5794	0.5000	0.5000	0.5000	0.5952	0.5457	0.5760
0.1	0.5966	0.5000	0.5000	0.5000	0.5928	0.6237	0.6043
0.05	0.6180	0.5000	0.5000	0.5000	0.5603	0.6105	0.5815
0.01	0.6101	0.5000	0.5000	0.5074	0.6318	0.6315	0.5481
0.0010	0.5941	0.4989	0.5012	0.5030	0.6343	0.6152	0.5454
1.0e-4	0.6258	0.4993	0.5115	0.5590	0.5966	0.6013	0.5970
1.0e-5	0.6511	0.4828	0.5371	0.6196	0.6579	0.6347	0.5626
1.0e-6	0.5867	0.5045	0.5371	0.5213	0.6343	0.5928	0.5183
1.0e-7	0.5628	0.4450	NA	0.5000	0.6495	0.6488	NA
				20RF			
0.15	0.5006	0.4860	0.5203	0.5122	0.5099	0.5156	0.5211
0.1	0.4883	0.4767	0.4935	0.5070	0.4912	0.5324	0.5044
0.05	0.5126	0.5200	0.5088	0.4826	0.5479	0.5502	0.5064
0.01	0.5490	0.4901	0.4935	0.4951	0.5924	0.5865	0.5361
0.0010	0.6038	0.4789	0.4993	0.5596	0.7071	0.7341	0.5544
1.0e-4	0.6346	0.4910	0.5393	0.5740	0.7267	0.7882	0.6039
1.0e-5	0.6544	0.4082	0.5512	0.6188	0.7415	0.7837	0.5601
1.0e-6	0.6230	0.4632	0.5077	0.5546	0.7448	0.7978	0.5204
1.0e-7	0.6019	0.4703	NA	0.5347	0.7493	0.8089	NA

Table S18: AUC obtained by all the algorithms under the haplotype-based approach with 2-SNP haplotype length, additive genetic model and holdout sampling. Results for each p-value threshold (column 1) are shown. The maximum p-value threshold used was 0.15.

p-value threshold	BD	CAD	HT	IBD	RA	T1D	T2D
			NBC				
0.15	0.5834	0.5000	0.5000	0.5578	0.6160	0.6523	0.5840
0.1	0.6034	0.5000	0.4977	0.5593	0.6388	0.6655	0.5974
0.05	0.6275	0.5001	0.5121	0.5384	0.6599	0.6907	0.5987
0.01	0.6463	0.4993	0.5150	0.5238	0.7114	0.7089	0.5999
0.0010	0.6612	0.5192	0.5335	0.5543	0.7439	0.7292	0.6094
1.0e-4	0.6596	0.4961	0.5207	0.5906	0.7365	0.7322	0.6180
1.0e-5	0.6571	0.4841	0.5313	0.6074	0.7271	0.7327	0.5543
1.0e-6	0.6229	0.5022	0.5076	0.5546	0.7180	0.7315	0.5155
1.0e-7	0.6019	0.4676	NA	0.5347	0.7106	0.7369	NA
			sSVM				
0.15	0.5072	0.5000	0.5000	0.5042	0.5066	0.5206	0.5193
0.1	0.5184	0.5000	0.5000	0.5146	0.5124	0.5364	0.5157
0.05	0.5215	0.5000	0.5000	0.5119	0.5176	0.5475	0.5218
0.01	0.5211	0.5017	0.4980	0.4927	0.5333	0.5772	0.5132
0.0010	0.5246	0.5061	0.5000	0.5193	0.5689	0.6469	0.5192
1.0e-4	0.5404	0.5000	0.5096	0.5104	0.6033	0.7000	0.5351
1.0e-5	0.5414	0.4602	0.5000	0.5430	0.6277	0.7201	0.5288
1.0e-6	0.5340	0.5124	0.5000	0.5001	0.6221	0.7195	0.5000
1.0e-7	0.5047	0.4602	NA	0.5000	0.6256	0.7171	NA
			AdaBoostM1				
0.15	0.6148	0.5136	0.4978	0.5706	0.7091	0.7738	0.5821
0.1	0.6351	0.5010	0.4955	0.5513	0.7037	0.7598	0.5657
0.05	0.6325	0.5210	0.4863	0.5493	0.7003	0.7592	0.5526
0.01	0.6190	0.5056	0.4995	0.5392	0.6807	0.7357	0.5961
0.0010	0.6836	0.4991	0.5292	0.5771	0.7565	0.7961	0.6116
1.0e-4	0.6695	0.4994	0.5247	0.5991	0.7957	0.8515	0.6282
1.0e-5	0.6477	0.4729	0.5155	0.6176	0.7968	0.8485	0.5665
1.0e-6	0.6239	0.5122	0.5076	0.5546	0.7795	0.8496	0.5204
1.0e-7	0.6020	0.4676	NA	0.5347	0.7779	0.8442	NA
			C4.5				
0.15	0.6004	0.5000	0.5000	0.5347	0.6104	0.5625	0.5889
0.1	0.6164	0.5000	0.5000	0.5347	0.6245	0.6563	0.6085
0.05	0.6317	0.5000	0.5000	0.5359	0.5925	0.6282	0.5846
0.01	0.6174	0.5000	0.5000	0.5011	0.6491	0.6470	0.5544
0.0010	0.6088	0.5042	0.5048	0.4966	0.6454	0.6444	0.5580
1.0e-4	0.6428	0.4939	0.5114	0.5670	0.6101	0.6371	0.5935
1.0e-5	0.6502	0.4778	0.5371	0.6184	0.6652	0.6676	0.5613
1.0e-6	0.5873	0.5044	0.5371	0.5213	0.6366	0.6374	0.5183
1.0e-7	0.5628	0.4450	NA	0.5000	0.6588	0.6664	NA
			20RF				
0.15	0.5002	0.4867	0.5194	0.5126	0.5089	0.5156	0.5213
0.1	0.4877	0.4774	0.4944	0.5061	0.4905	0.5325	0.5041
0.05	0.5122	0.5186	0.5099	0.4834	0.5472	0.5498	0.5060
0.01	0.5486	0.4902	0.4930	0.4946	0.5916	0.5873	0.5364
0.0010	0.6037	0.4800	0.4987	0.5601	0.7076	0.7303	0.5539
1.0e-4	0.6340	0.4899	0.5389	0.5752	0.7277	0.7837	0.6031
1.0e-5	0.6538	0.4072	0.5447	0.6175	0.7428	0.7795	0.5599
1.0e-6	0.6231	0.4614	0.5077	0.5546	0.7460	0.7927	0.5204
1.0e-7	0.6019	0.4636	NA	0.5347	0.7517	0.8044	NA

Table S19: AUC obtained by all the algorithms under the haplotype-based approach with 2-SNP haplotype length, dominant genetic model and holdout sampling. Results for each p-value threshold (column 1) are shown. The maximum p-value threshold used was 0.15.

p-value threshold	BD	CAD	HT	IBD	RA	T1D	T2D
				NBC			
0.15	0.6470	0.5000	0.5000	0.5859	0.6489	0.6564	0.5939
0.1	0.6475	0.5000	0.5000	0.5760	0.6623	0.6656	0.5924
0.05	0.6521	0.4970	0.5230	0.5528	0.6717	0.6859	0.5960
0.01	0.6431	0.4968	0.5166	0.5373	0.6932	0.7347	0.5912
0.0010	0.6397	0.5287	0.5384	0.5510	0.7099	0.7709	0.6121
1.0e-4	0.6366	0.5098	0.5204	0.5802	0.7080	0.7703	0.6277
1.0e-5	0.6542	0.4833	0.5312	0.6086	0.6992	0.7685	0.5602
1.0e-6	0.6211	0.5011	0.5076	0.5548	0.6864	0.7660	0.5197
1.0e-7	0.6002	0.4677	NA	0.5347	0.6770	0.7643	NA
				sSVM			
0.15	0.5589	0.5000	0.5000	0.5295	0.5390	0.5732	0.5286
0.1	0.5572	0.5000	0.5000	0.5587	0.5523	0.5772	0.5404
0.05	0.5474	0.5000	0.5000	0.5215	0.5414	0.5852	0.5352
0.01	0.5480	0.5111	0.5045	0.5042	0.5635	0.6332	0.5155
0.0010	0.5479	0.5179	0.5036	0.5319	0.6079	0.6975	0.5242
1.0e-4	0.5537	0.5041	0.5032	0.5250	0.6636	0.7497	0.5537
1.0e-5	0.5853	0.5019	0.5371	0.5495	0.6717	0.7542	0.5378
1.0e-6	0.5849	0.4928	0.5371	0.5213	0.6731	0.7516	0.5183
1.0e-7	0.5629	0.5019	NA	0.5000	0.6588	0.7455	NA
				AdaBoostM1			
0.15	0.6058	0.5197	0.4925	0.5589	0.6859	0.7809	0.5744
0.1	0.6129	0.4957	0.4902	0.5415	0.6950	0.7614	0.5712
0.05	0.6081	0.5264	0.5093	0.5398	0.6905	0.7618	0.5601
0.01	0.6155	0.5257	0.5097	0.5395	0.6625	0.7339	0.5691
0.0010	0.6533	0.5057	0.5333	0.5747	0.7484	0.7983	0.6226
1.0e-4	0.6518	0.5175	0.5220	0.6004	0.7916	0.8460	0.6342
1.0e-5	0.6485	0.4702	0.5144	0.6213	0.7971	0.8472	0.5658
1.0e-6	0.6219	0.5105	0.5076	0.5548	0.7776	0.8510	0.5204
1.0e-7	0.6002	0.4677	NA	0.5347	0.7675	0.8490	NA
				C4.5			
0.15	0.5783	0.5000	0.5000	0.5000	0.6086	0.5794	0.5982
0.1	0.5874	0.5000	0.5000	0.5000	0.6110	0.6327	0.6194
0.05	0.6235	0.5000	0.5000	0.5000	0.5912	0.6327	0.5976
0.01	0.6180	0.5000	0.5000	0.5122	0.6590	0.6619	0.5572
0.0010	0.5982	0.4933	0.4986	0.5139	0.6689	0.6705	0.5559
1.0e-4	0.6147	0.4993	0.5237	0.5605	0.6564	0.6682	0.5994
1.0e-5	0.6493	0.4730	0.5371	0.6198	0.7068	0.6868	0.5609
1.0e-6	0.5867	0.5035	0.5371	0.5213	0.6866	0.6557	0.5183
1.0e-7	0.5628	0.4450	NA	0.5000	0.6908	0.7034	NA
				20RF			
0.15	0.5008	0.4852	0.5207	0.5118	0.5113	0.5160	0.5208
0.1	0.4888	0.4770	0.4924	0.5081	0.4920	0.5323	0.5049
0.05	0.5130	0.5210	0.5077	0.4814	0.5489	0.5504	0.5070
0.01	0.5489	0.4900	0.4941	0.4957	0.5935	0.5856	0.5355
0.0010	0.6035	0.4774	0.5000	0.5591	0.7058	0.7388	0.5551
1.0e-4	0.6352	0.4925	0.5393	0.5717	0.7244	0.7924	0.6044
1.0e-5	0.6536	0.4090	0.5544	0.6177	0.7387	0.7870	0.5591
1.0e-6	0.6221	0.4638	0.5077	0.5548	0.7418	0.8024	0.5204
1.0e-7	0.6002	0.4703	NA	0.5347	0.7436	0.8121	NA

Table S20: AUC obtained by all the algorithms under the haplotype-based approach with 2-SNP haplotype length, recessive genetic model and holdout sampling. Results for each p-value threshold (column 1) are shown. The maximum p-value threshold used was 0.15.

p-value threshold	BD	CAD	HT	IBD	RA	T1D	T2D
			NBC				
0.15	0.6035	0.5000	0.5000	0.5457	0.6221	0.6730	0.5880
0.1	0.6096	0.4969	0.4957	0.5375	0.6453	0.6823	0.5915
0.05	0.6349	0.4949	0.4974	0.5520	0.6680	0.7027	0.5958
0.01	0.6462	0.5145	0.5020	0.5467	0.7056	0.7189	0.5981
0.0010	0.6819	0.5400	0.5041	0.5395	0.7241	0.7356	0.6217
1.0e-4	0.6650	0.4985	0.4656	0.5981	0.7220	0.7470	0.6225
1.0e-5	0.6578	0.5252	0.4628	0.6120	0.7081	0.7546	0.5985
1.0e-6	0.6208	0.5118	0.5023	0.5627	0.7034	0.7541	0.5381
1.0e-7	0.5980	0.5345	NA	0.5347	0.6943	0.7587	NA
			sSVM				
0.15	0.5280	0.5000	0.5000	0.5252	0.5303	0.5513	0.5124
0.1	0.5323	0.5000	0.5000	0.5172	0.5393	0.5619	0.5220
0.05	0.5386	0.5000	0.5000	0.5192	0.5413	0.5709	0.5235
0.01	0.5332	0.5135	0.4913	0.5193	0.5523	0.5818	0.5069
0.0010	0.5442	0.5136	0.5050	0.5105	0.5845	0.6644	0.5300
1.0e-4	0.5576	0.5081	0.4836	0.5405	0.6230	0.7173	0.5394
1.0e-5	0.5608	0.5011	0.5000	0.5398	0.6391	0.7329	0.5409
1.0e-6	0.5562	0.4955	0.5000	0.5168	0.6372	0.7292	0.5048
1.0e-7	0.5264	0.5000	NA	0.5000	0.6399	0.7295	NA
			AdaBoostM1				
0.15	0.6369	0.5054	0.4988	0.5565	0.6991	0.7799	0.5571
0.1	0.6416	0.4843	0.4961	0.5604	0.6980	0.7799	0.5551
0.05	0.6282	0.4974	0.4995	0.5617	0.7090	0.7732	0.5809
0.01	0.6189	0.5259	0.4878	0.5571	0.6719	0.7314	0.5558
0.0010	0.6873	0.5574	0.5147	0.5635	0.7313	0.7928	0.6056
1.0e-4	0.6781	0.5135	0.4525	0.6121	0.7807	0.8619	0.6188
1.0e-5	0.6538	0.5327	0.4582	0.6193	0.7822	0.8595	0.6141
1.0e-6	0.6228	0.5360	0.5023	0.5635	0.7791	0.8682	0.5381
1.0e-7	0.5980	0.4983	NA	0.5347	0.7630	0.8543	NA
			C4.5				
0.15	0.5947	0.5000	0.5000	0.5000	0.6262	0.5865	0.5626
0.1	0.6190	0.5000	0.5000	0.5000	0.6290	0.5865	0.5493
0.05	0.6180	0.5000	0.5000	0.5158	0.6362	0.6169	0.5697
0.01	0.6001	0.5000	0.5000	0.5202	0.6196	0.5841	0.5290
0.0010	0.5905	0.5075	0.4978	0.5284	0.6086	0.6080	0.5481
1.0e-4	0.5922	0.5000	0.5018	0.5595	0.6293	0.6518	0.5721
1.0e-5	0.6490	0.4822	0.4986	0.6103	0.6537	0.6477	0.6100
1.0e-6	0.5861	0.4930	0.5261	0.5635	0.6738	0.6255	0.5392
1.0e-7	0.5582	0.5000	NA	0.5000	0.6545	0.6409	NA
			20RF				
0.15	0.4893	0.4963	0.5049	0.5079	0.5235	0.5218	0.4811
0.1	0.5129	0.4806	0.5098	0.4978	0.5329	0.5338	0.5040
0.05	0.5183	0.5084	0.5028	0.5343	0.5323	0.5443	0.5446
0.01	0.5374	0.4780	0.5218	0.4990	0.5753	0.6102	0.5418
0.0010	0.5979	0.5264	0.4986	0.5179	0.6957	0.7292	0.5689
1.0e-4	0.6151	0.4758	0.4989	0.5835	0.7246	0.7854	0.6367
1.0e-5	0.6549	0.4736	0.4718	0.6132	0.7398	0.7918	0.6028
1.0e-6	0.6227	0.4618	0.5023	0.5627	0.7442	0.7875	0.5373
1.0e-7	0.5980	0.5603	NA	0.5347	0.7410	0.8015	NA

Table S21: AUC obtained by all the algorithms under the haplotype-based approach with 3-SNP haplotype length, additive genetic model and holdout sampling. Results for each p-value threshold (column 1) are shown. The maximum p-value threshold used was 0.15.

p-value threshold	BD	CAD	HT	IBD	RA	T1D	T2D
NBC							
0.15	0.5982	0.4966	0.5045	0.5455	0.6187	0.6729	0.5919
0.1	0.6053	0.4917	0.4945	0.5308	0.6439	0.6818	0.5921
0.05	0.6245	0.4966	0.4957	0.5442	0.6644	0.6995	0.5865
0.01	0.6361	0.5047	0.4975	0.5393	0.7066	0.7122	0.5916
0.0010	0.6757	0.5221	0.5053	0.5326	0.7374	0.7261	0.6079
1.0e-4	0.6617	0.4949	0.4606	0.5909	0.7320	0.7334	0.6151
1.0e-5	0.6587	0.5203	0.4619	0.6083	0.7148	0.7358	0.5964
1.0e-6	0.6215	0.5089	0.5023	0.5632	0.7105	0.7359	0.5390
1.0e-7	0.5980	0.5245	NA	0.5347	0.7023	0.7409	NA
sSVM							
0.15	0.5108	0.5000	0.5000	0.5265	0.5107	0.5288	0.5078
0.1	0.5180	0.5000	0.5000	0.5098	0.5187	0.5455	0.5171
0.05	0.5249	0.5000	0.5000	0.5124	0.5298	0.5520	0.5106
0.01	0.5152	0.5128	0.4860	0.5223	0.5363	0.5552	0.5044
0.0010	0.5362	0.5185	0.5100	0.5131	0.5723	0.6487	0.5271
1.0e-4	0.5557	0.5007	0.4853	0.5329	0.6119	0.6964	0.5327
1.0e-5	0.5426	0.5064	0.4656	0.5262	0.6239	0.7145	0.5235
1.0e-6	0.5367	0.5016	0.5000	0.5096	0.6216	0.7129	0.5003
1.0e-7	0.5042	0.5000	NA	0.5000	0.6272	0.7171	NA
AdaBoostM1							
0.15	0.6259	0.5086	0.5059	0.5528	0.6831	0.7594	0.5476
0.1	0.6388	0.4895	0.5089	0.5561	0.6893	0.7570	0.5445
0.05	0.6160	0.4960	0.5050	0.5643	0.7006	0.7533	0.5654
0.01	0.6192	0.5113	0.4845	0.5472	0.6538	0.7114	0.5470
0.0010	0.6870	0.5475	0.5148	0.5570	0.7236	0.7825	0.5987
1.0e-4	0.6744	0.5106	0.4509	0.6055	0.7771	0.8497	0.6154
1.0e-5	0.6564	0.5233	0.4583	0.6153	0.7794	0.8503	0.6139
1.0e-6	0.6230	0.5296	0.5023	0.5631	0.7808	0.8609	0.5386
1.0e-7	0.5980	0.4983	NA	0.5347	0.7657	0.8482	NA
C4.5							
0.15	0.6108	0.5000	0.5000	0.5347	0.6339	0.5802	0.5638
0.1	0.6257	0.5000	0.5000	0.5347	0.6255	0.5877	0.5510
0.05	0.6307	0.5000	0.5000	0.5247	0.6480	0.6377	0.5749
0.01	0.6063	0.5000	0.5000	0.5293	0.6423	0.6027	0.5322
0.0010	0.5905	0.5120	0.4955	0.5309	0.6091	0.6337	0.5569
1.0e-4	0.6115	0.4993	0.5078	0.5549	0.6342	0.6920	0.5807
1.0e-5	0.6483	0.4833	0.4463	0.6090	0.6547	0.6760	0.6065
1.0e-6	0.5865	0.4914	0.5261	0.5635	0.6905	0.6635	0.5392
1.0e-7	0.5582	0.5000	NA	0.5000	0.6852	0.6611	NA
20RF							
0.15	0.4887	0.4956	0.5063	0.5080	0.5233	0.5218	0.4817
0.1	0.5135	0.4823	0.5103	0.4984	0.5330	0.5339	0.5030
0.05	0.5194	0.5082	0.5016	0.5347	0.5332	0.5445	0.5445
0.01	0.5374	0.4783	0.5234	0.4985	0.5751	0.6099	0.5417
0.0010	0.5974	0.5253	0.4976	0.5172	0.6969	0.7254	0.5688
1.0e-4	0.6155	0.4753	0.4988	0.5818	0.7256	0.7796	0.6364
1.0e-5	0.6564	0.4700	0.4641	0.6111	0.7401	0.7860	0.6025
1.0e-6	0.6227	0.4644	0.5023	0.5635	0.7445	0.7832	0.5377
1.0e-7	0.5981	0.5524	NA	0.5347	0.7424	0.7974	NA

Table S22: AUC obtained by all the algorithms under the haplotype-based approach with 3-SNP haplotype length, dominant genetic model and holdout sampling. Results for each p-value threshold (column 1) are shown. The maximum p-value threshold used was 0.15.

p-value threshold	BD	CAD	HT	IBD	RA	T1D	T2D
				NBC			
0.15	0.6408	0.5000	0.5000	0.5458	0.6596	0.6751	0.5819
0.1	0.6381	0.4969	0.4957	0.5373	0.6696	0.6824	0.5817
0.05	0.6408	0.4949	0.4974	0.5537	0.6767	0.7081	0.5970
0.01	0.6399	0.5145	0.5020	0.5451	0.6906	0.7427	0.5966
0.0010	0.6649	0.5401	0.5023	0.5369	0.7004	0.7725	0.6176
1.0e-4	0.6475	0.5006	0.4676	0.5917	0.6992	0.7799	0.6159
1.0e-5	0.6509	0.5330	0.4626	0.6069	0.6871	0.7759	0.5895
1.0e-6	0.6197	0.5160	0.5023	0.5637	0.6802	0.7726	0.5348
1.0e-7	0.5962	0.5345	NA	0.5347	0.6721	0.7691	NA
				sSVM			
0.15	0.5524	0.5000	0.5000	0.5237	0.5582	0.5864	0.5184
0.1	0.5524	0.5000	0.5000	0.5265	0.5693	0.5867	0.5286
0.05	0.5577	0.5000	0.5000	0.5276	0.5570	0.6002	0.5422
0.01	0.5597	0.5143	0.4987	0.5159	0.5753	0.6272	0.5101
0.0010	0.5550	0.5076	0.4986	0.5075	0.6016	0.6880	0.5338
1.0e-4	0.5602	0.5181	0.4813	0.5502	0.6381	0.7496	0.5486
1.0e-5	0.5862	0.4945	0.5000	0.5574	0.6603	0.7608	0.5668
1.0e-6	0.5837	0.4879	0.5261	0.5258	0.6592	0.7533	0.5109
1.0e-7	0.5581	0.5000	NA	0.5000	0.6572	0.7472	NA
				AdaBoostM1			
0.15	0.6232	0.5032	0.4923	0.5510	0.6851	0.7625	0.5545
0.1	0.6173	0.4842	0.4902	0.5475	0.6773	0.7694	0.5592
0.05	0.6152	0.5019	0.4973	0.5465	0.6910	0.7589	0.5879
0.01	0.5978	0.5295	0.4883	0.5481	0.6689	0.7182	0.5576
0.0010	0.6554	0.5550	0.5079	0.5542	0.7137	0.7670	0.5981
1.0e-4	0.6611	0.5144	0.4527	0.6052	0.7669	0.8531	0.6141
1.0e-5	0.6471	0.5341	0.4603	0.6174	0.7767	0.8556	0.6110
1.0e-6	0.6208	0.5352	0.5023	0.5637	0.7721	0.8633	0.5362
1.0e-7	0.5962	0.4983	NA	0.5347	0.7522	0.8515	NA
				C4.5			
0.15	0.5963	0.5000	0.5000	0.5000	0.6392	0.6203	0.5767
0.1	0.6359	0.5000	0.5000	0.5000	0.6603	0.6311	0.5681
0.05	0.6263	0.5000	0.5000	0.5041	0.6542	0.6422	0.5844
0.01	0.6109	0.5000	0.5000	0.5102	0.6243	0.6234	0.5438
0.0010	0.6054	0.5019	0.5008	0.5284	0.6496	0.6482	0.5664
1.0e-4	0.5909	0.5000	0.4953	0.5662	0.6787	0.6673	0.5722
1.0e-5	0.6408	0.4764	0.4986	0.6070	0.6928	0.7008	0.6085
1.0e-6	0.5860	0.4930	0.5261	0.5637	0.6913	0.6958	0.5378
1.0e-7	0.5582	0.5000	NA	0.5000	0.6679	0.7049	NA
				20RF			
0.15	0.4900	0.4971	0.5035	0.5076	0.5235	0.5216	0.4805
0.1	0.5124	0.4793	0.5100	0.4967	0.5332	0.5336	0.5052
0.05	0.5173	0.5095	0.5049	0.5340	0.5311	0.5438	0.5444
0.01	0.5374	0.4772	0.5185	0.4996	0.5752	0.6101	0.5416
0.0010	0.5983	0.5275	0.4996	0.5184	0.6940	0.7334	0.5683
1.0e-4	0.6143	0.4763	0.4984	0.5839	0.7225	0.7906	0.6355
1.0e-5	0.6531	0.4769	0.4762	0.6107	0.7380	0.7969	0.6002
1.0e-6	0.6217	0.4609	0.5023	0.5637	0.7417	0.7908	0.5354
1.0e-7	0.5962	0.5761	NA	0.5347	0.7372	0.8041	NA

Table S23: AUC obtained by all the algorithms under the haplotype-based approach with 3-SNP haplotype length, recessive genetic model and holdout sampling. Results for each p-value threshold (column 1) are shown. The maximum p-value threshold used was 0.15.

p-value threshold	BD	CAD	HT	IBD	RA	T1D	T2D
				NBC			
0.15	0.6053	0.4918	0.5087	0.5640	0.6162	0.6703	0.5928
0.1	0.6109	0.4926	0.5049	0.5524	0.6362	0.6873	0.5965
0.05	0.6271	0.5025	0.5004	0.5600	0.6666	0.7115	0.6066
0.01	0.6269	0.4987	0.4914	0.5477	0.7019	0.7261	0.6009
0.0010	0.6394	0.5204	0.5032	0.5502	0.7133	0.7344	0.6188
1.0e-4	0.6576	0.5369	0.4912	0.5625	0.7089	0.7336	0.5917
1.0e-5	0.6440	0.5011	0.4762	0.5892	0.7007	0.7423	0.5962
1.0e-6	0.6065	0.5380	NA	0.5483	0.6942	0.7505	0.5486
1.0e-7	0.5665	0.5723	NA	0.5366	0.6991	0.7524	NA
				sSVM			
0.15	0.5320	0.5000	0.5000	0.5247	0.5353	0.5556	0.5300
0.1	0.5345	0.5000	0.5000	0.5156	0.5400	0.5696	0.5384
0.05	0.5367	0.5000	0.5000	0.5067	0.5424	0.5774	0.5311
0.01	0.5326	0.4937	0.4972	0.5172	0.5433	0.5979	0.5185
0.0010	0.5346	0.5052	0.5082	0.5056	0.5729	0.6605	0.5183
1.0e-4	0.5575	0.5076	0.4854	0.5226	0.6236	0.7101	0.5260
1.0e-5	0.5540	0.4978	0.5184	0.5358	0.6347	0.7266	0.5273
1.0e-6	0.5383	0.4981	NA	0.5093	0.6467	0.7265	0.5151
1.0e-7	0.5000	0.5000	NA	0.5000	0.6479	0.7264	NA
				AdaBoostM1			
0.15	0.6137	0.5215	0.4852	0.5471	0.7124	0.7969	0.5789
0.1	0.6294	0.5122	0.5114	0.5448	0.7029	0.7801	0.5722
0.05	0.6140	0.4859	0.4993	0.5509	0.6983	0.7786	0.5708
0.01	0.6154	0.4977	0.5041	0.5355	0.6816	0.7402	0.5850
0.0010	0.6644	0.5262	0.5068	0.5274	0.7041	0.7812	0.6096
1.0e-4	0.6648	0.5281	0.5014	0.5573	0.7766	0.8425	0.5936
1.0e-5	0.6410	0.5008	0.5019	0.5952	0.7613	0.8581	0.6018
1.0e-6	0.6074	0.5318	NA	0.5483	0.7748	0.8632	0.5488
1.0e-7	0.5665	0.5736	NA	0.5366	0.7665	0.8452	NA
				C4.5			
0.15	0.6042	0.5000	0.5000	0.5000	0.6217	0.5702	0.5509
0.1	0.5964	0.5000	0.5000	0.5000	0.6264	0.5492	0.5539
0.05	0.6353	0.5000	0.5000	0.5171	0.5871	0.5593	0.5534
0.01	0.6048	0.5000	0.5000	0.5213	0.6040	0.6388	0.5476
0.0010	0.5794	0.5036	0.5097	0.5208	0.5931	0.6083	0.5429
1.0e-4	0.6145	0.5021	0.4915	0.5614	0.5898	0.6122	0.5468
1.0e-5	0.6380	0.5157	0.4921	0.5864	0.5913	0.6078	0.5934
1.0e-6	0.5685	0.5048	NA	0.5483	0.5982	0.6139	0.5449
1.0e-7	0.5236	0.4899	NA	0.5000	0.6120	0.6314	NA
				20RF			
0.15	0.5036	0.4809	0.5196	0.5047	0.5080	0.5222	0.4826
0.1	0.5267	0.5095	0.4953	0.4908	0.5155	0.5310	0.4973
0.05	0.5324	0.5003	0.5001	0.5027	0.5269	0.5577	0.5088
0.01	0.5143	0.5055	0.5063	0.5090	0.5749	0.6703	0.5395
0.0010	0.5765	0.4734	0.4991	0.5467	0.6604	0.7202	0.5694
1.0e-4	0.6340	0.5355	0.4868	0.5528	0.7204	0.7827	0.5661
1.0e-5	0.6417	0.5051	0.5283	0.5956	0.7252	0.7895	0.5888
1.0e-6	0.6072	0.4879	NA	0.5483	0.7232	0.8044	0.5533
1.0e-7	0.5665	0.4247	NA	0.5366	0.7355	0.7904	NA

Table S24: AUC obtained by all the algorithms under the haplotype-based approach with 4-SNP haplotype length, additive genetic model and holdout sampling. Results for each p-value threshold (column 1) are shown. The maximum p-value threshold used was 0.15.

p-value threshold	BD	CAD	HT	IBD	RA	T1D	T2D
				NBC			
0.15	0.5980	0.4941	0.5059	0.5598	0.6131	0.6688	0.5919
0.1	0.6032	0.4956	0.5058	0.5470	0.6309	0.6837	0.5930
0.05	0.6167	0.4997	0.5024	0.5549	0.6651	0.7081	0.6009
0.01	0.6152	0.5017	0.4879	0.5362	0.7006	0.7204	0.5913
0.0010	0.6299	0.5234	0.4931	0.5432	0.7223	0.7233	0.6099
1.0e-4	0.6463	0.5366	0.4883	0.5552	0.7185	0.7231	0.5849
1.0e-5	0.6426	0.5007	0.4762	0.5904	0.7081	0.7267	0.5926
1.0e-6	0.6065	0.5366	NA	0.5483	0.7027	0.7307	0.5523
1.0e-7	0.5665	0.5716	NA	0.5366	0.7089	0.7322	NA
				sSVM			
0.15	0.5105	0.5000	0.5000	0.5130	0.5153	0.5295	0.5217
0.1	0.5132	0.5000	0.5000	0.5073	0.5257	0.5447	0.5264
0.05	0.5228	0.5000	0.5000	0.5045	0.5253	0.5556	0.5213
0.01	0.5237	0.4911	0.4984	0.5085	0.5298	0.5753	0.5110
0.0010	0.5273	0.5087	0.5073	0.4980	0.5553	0.6391	0.5123
1.0e-4	0.5462	0.5023	0.4822	0.5195	0.6084	0.6866	0.5190
1.0e-5	0.5363	0.5073	0.5008	0.5275	0.6212	0.7046	0.5214
1.0e-6	0.5187	0.5075	NA	0.5066	0.6408	0.7126	0.5087
1.0e-7	0.5000	0.5000	NA	0.5000	0.6424	0.7133	NA
				AdaBoostM1			
0.15	0.6104	0.5201	0.4862	0.5356	0.7030	0.7771	0.5683
0.1	0.6249	0.4918	0.5151	0.5395	0.6952	0.7633	0.5671
0.05	0.6137	0.4926	0.5012	0.5526	0.6894	0.7609	0.5699
0.01	0.6119	0.4988	0.5035	0.5452	0.6687	0.7214	0.5786
0.0010	0.6611	0.5283	0.5080	0.5252	0.7085	0.7658	0.5983
1.0e-4	0.6569	0.5280	0.5010	0.5521	0.7759	0.8337	0.5887
1.0e-5	0.6410	0.5009	0.5019	0.5930	0.7593	0.8473	0.6004
1.0e-6	0.6074	0.5317	NA	0.5483	0.7753	0.8573	0.5526
1.0e-7	0.5665	0.5733	NA	0.5366	0.7692	0.8398	NA
				C4.5			
0.15	0.6175	0.5000	0.5000	0.5366	0.6370	0.5912	0.5463
0.1	0.6075	0.5000	0.5000	0.5386	0.6354	0.5596	0.5638
0.05	0.6404	0.5000	0.5000	0.5297	0.5989	0.5875	0.5645
0.01	0.6046	0.5000	0.4997	0.5330	0.6171	0.6521	0.5448
0.0010	0.5924	0.4971	0.5103	0.5413	0.5885	0.6242	0.5440
1.0e-4	0.6230	0.5038	0.4943	0.5609	0.5961	0.6288	0.5479
1.0e-5	0.6360	0.5157	0.4921	0.5870	0.6051	0.6243	0.5906
1.0e-6	0.5686	0.5048	NA	0.5483	0.6200	0.6418	0.5448
1.0e-7	0.5237	0.4899	NA	0.5000	0.6288	0.6470	NA
				20RF			
0.15	0.5027	0.4788	0.5208	0.5045	0.5076	0.5224	0.4826
0.1	0.5263	0.5090	0.4972	0.4902	0.5148	0.5304	0.4979
0.05	0.5323	0.4994	0.5010	0.5040	0.5264	0.5583	0.5091
0.01	0.5140	0.5044	0.5061	0.5098	0.5742	0.6703	0.5389
0.0010	0.5770	0.4727	0.4980	0.5472	0.6612	0.7172	0.5689
1.0e-4	0.6331	0.5353	0.4854	0.5534	0.7208	0.7769	0.5651
1.0e-5	0.6416	0.5035	0.5222	0.5939	0.7253	0.7851	0.5878
1.0e-6	0.6073	0.4891	NA	0.5483	0.7241	0.7986	0.5542
1.0e-7	0.5665	0.4248	NA	0.5366	0.7371	0.7845	NA

Table S25: AUC obtained by all the algorithms under the haplotype-based approach with 4-SNP haplotype length, dominant genetic model and holdout sampling. Results for each p-value threshold (column 1) are shown. The maximum p-value threshold used was 0.15.

p-value threshold	BD	CAD	HT	IBD	RA	T1D	T2D
				NBC			
0.15	0.6412	0.4918	0.5087	0.5592	0.6567	0.6866	0.5908
0.1	0.6369	0.4926	0.5049	0.5505	0.6634	0.6955	0.5954
0.05	0.6339	0.5025	0.5004	0.5390	0.6668	0.7188	0.5948
0.01	0.6273	0.4987	0.4916	0.5511	0.6836	0.7527	0.5961
0.0010	0.6307	0.5185	0.5055	0.5443	0.6916	0.7687	0.6118
1.0e-4	0.6455	0.5399	0.4937	0.5585	0.6866	0.7666	0.5881
1.0e-5	0.6393	0.5005	0.4763	0.5837	0.6781	0.7664	0.5927
1.0e-6	0.6056	0.5368	NA	0.5483	0.6717	0.7677	0.5439
1.0e-7	0.5665	0.5724	NA	0.5366	0.6739	0.7681	NA
				sSVM			
0.15	0.5635	0.5000	0.5000	0.5399	0.5638	0.5976	0.5415
0.1	0.5663	0.5000	0.5000	0.5261	0.5599	0.6101	0.5558
0.05	0.5561	0.5014	0.5000	0.5094	0.5671	0.6121	0.5449
0.01	0.5447	0.4972	0.4955	0.5283	0.5623	0.6348	0.5288
0.0010	0.5444	0.5007	0.5094	0.5152	0.5985	0.6944	0.5265
1.0e-4	0.5733	0.5146	0.4898	0.5263	0.6450	0.7474	0.5357
1.0e-5	0.5790	0.4862	0.5438	0.5464	0.6535	0.7606	0.5355
1.0e-6	0.5659	0.4865	NA	0.5126	0.6543	0.7469	0.5238
1.0e-7	0.5236	0.5088	NA	0.5000	0.6551	0.7453	NA
				AdaBoostM1			
0.15	0.5918	0.5084	0.4911	0.5428	0.6911	0.7796	0.5781
0.1	0.6032	0.5119	0.5008	0.5395	0.6832	0.7676	0.5675
0.05	0.5955	0.4836	0.5001	0.5362	0.6811	0.7607	0.5629
0.01	0.5900	0.4981	0.5061	0.5228	0.6612	0.7246	0.5841
0.0010	0.6328	0.5130	0.5016	0.5209	0.6788	0.7582	0.5999
1.0e-4	0.6488	0.5299	0.5056	0.5495	0.7597	0.8291	0.5896
1.0e-5	0.6356	0.5025	0.5033	0.5978	0.7539	0.8538	0.5984
1.0e-6	0.6065	0.5306	NA	0.5483	0.7633	0.8575	0.5448
1.0e-7	0.5665	0.5735	NA	0.5366	0.7541	0.8435	NA
				C4.5			
0.15	0.6137	0.5000	0.5000	0.5000	0.6251	0.5962	0.5544
0.1	0.6213	0.5000	0.5000	0.5000	0.6473	0.5928	0.5628
0.05	0.6326	0.5000	0.5000	0.5031	0.6135	0.6151	0.5617
0.01	0.6227	0.5000	0.5000	0.5085	0.6196	0.6613	0.5649
0.0010	0.5866	0.5124	0.5094	0.5066	0.6383	0.6472	0.5443
1.0e-4	0.6122	0.4999	0.4935	0.5510	0.6445	0.6673	0.5475
1.0e-5	0.6331	0.5157	0.4921	0.5864	0.6412	0.6616	0.5918
1.0e-6	0.5685	0.5060	NA	0.5483	0.6380	0.6889	0.5449
1.0e-7	0.5236	0.4899	NA	0.5000	0.6487	0.6918	NA
				20RF			
0.15	0.5049	0.4840	0.5181	0.5049	0.5088	0.5220	0.4825
0.1	0.5268	0.5098	0.4937	0.4915	0.5164	0.5315	0.4968
0.05	0.5327	0.5010	0.4998	0.5009	0.5277	0.5569	0.5081
0.01	0.5148	0.5065	0.5069	0.5081	0.5754	0.6698	0.5400
0.0010	0.5759	0.4739	0.5002	0.5456	0.6591	0.7244	0.5700
1.0e-4	0.6348	0.5359	0.4890	0.5513	0.7189	0.7891	0.5671
1.0e-5	0.6394	0.5067	0.5293	0.5949	0.7234	0.7931	0.5891
1.0e-6	0.6063	0.4877	NA	0.5483	0.7207	0.8100	0.5493
1.0e-7	0.5665	0.4247	NA	0.5366	0.7315	0.7958	NA

Table S26: AUC obtained by all the algorithms under the haplotype-based approach with 4-SNP haplotype length, recessive genetic model and holdout sampling. Results for each p-value threshold (column 1) are shown. The maximum p-value threshold used was 0.15.

p-value threshold	BD	CAD	HT	IBD	RA	T1D	T2D
				NBC			
0.15	0.5926	0.4927	0.4928	0.5589	0.6390	0.6788	0.5933
0.1	0.5999	0.5047	0.5034	0.5524	0.6547	0.6909	0.5886
0.05	0.5994	0.4928	0.5002	0.5497	0.6745	0.7101	0.5865
0.01	0.6153	0.4896	0.4974	0.5357	0.6993	0.7272	0.5893
0.0010	0.6410	0.5105	0.4825	0.5477	0.7033	0.7328	0.5911
1.0e-4	0.6425	0.4820	0.5359	0.5699	0.6979	0.7363	0.5786
1.0e-5	0.6512	0.4793	0.5573	0.5903	0.6910	0.7375	0.5956
1.0e-6	0.6157	0.5021	NA	0.5499	0.6874	0.7564	0.5340
1.0e-7	0.5821	0.5535	NA	0.5347	0.6986	0.7592	NA
				sSVM			
0.15	0.5306	0.5000	0.5000	0.5143	0.5388	0.5624	0.5309
0.1	0.5381	0.5000	0.5000	0.5125	0.5493	0.5712	0.5341
0.05	0.5298	0.5000	0.5000	0.5170	0.5502	0.5685	0.5183
0.01	0.5323	0.4965	0.5138	0.5140	0.5514	0.5926	0.5212
0.0010	0.5292	0.5007	0.4970	0.5196	0.5758	0.6603	0.5307
1.0e-4	0.5445	0.5082	0.5077	0.5198	0.6022	0.7023	0.5215
1.0e-5	0.5568	0.5000	0.5000	0.5344	0.6255	0.7195	0.5269
1.0e-6	0.5449	0.5000	NA	0.5096	0.6232	0.7205	0.5000
1.0e-7	0.5211	0.5000	NA	0.5000	0.6193	0.7171	NA
				AdaBoostM1			
0.15	0.6179	0.5187	0.5087	0.5509	0.6749	0.7745	0.5654
0.1	0.6155	0.5191	0.4784	0.5768	0.6737	0.7800	0.5725
0.05	0.5974	0.4994	0.5105	0.5396	0.6725	0.7666	0.5516
0.01	0.5870	0.4975	0.5110	0.5138	0.6496	0.7215	0.5714
0.0010	0.6545	0.4981	0.4675	0.5440	0.6910	0.7400	0.6009
1.0e-4	0.6476	0.4753	0.5372	0.5649	0.7454	0.8253	0.5820
1.0e-5	0.6494	0.4652	0.5409	0.5978	0.7501	0.8408	0.5948
1.0e-6	0.6154	0.4830	NA	0.5499	0.7526	0.8397	0.5340
1.0e-7	0.5821	0.5213	NA	0.5347	0.7554	0.8366	NA
				C4.5			
0.15	0.5826	0.5000	0.5000	0.5188	0.5599	0.5818	0.5223
0.1	0.5630	0.5000	0.5000	0.5082	0.6021	0.6122	0.5226
0.05	0.5969	0.5000	0.5000	0.5186	0.5987	0.6050	0.5420
0.01	0.5877	0.4992	0.5000	0.5106	0.5811	0.6053	0.5199
0.0010	0.5510	0.5000	0.5000	0.5244	0.5780	0.6135	0.5327
1.0e-4	0.5550	0.4984	0.4982	0.5084	0.5612	0.6247	0.5329
1.0e-5	0.5913	0.5258	0.5000	0.5954	0.5377	0.6222	0.5769
1.0e-6	0.5799	0.5000	NA	0.5499	0.5595	0.5977	0.5000
1.0e-7	0.5424	0.5000	NA	0.5000	0.6015	0.5893	NA
				20RF			
0.15	0.5192	0.5038	0.5018	0.5096	0.4903	0.5299	0.5007
0.1	0.4870	0.4873	0.4962	0.5085	0.5154	0.5538	0.4986
0.05	0.5120	0.5111	0.5108	0.5354	0.5370	0.5179	0.4935
0.01	0.5052	0.5022	0.4615	0.5174	0.5509	0.6436	0.5131
0.0010	0.5547	0.5050	0.5279	0.5040	0.6782	0.7570	0.5674
1.0e-4	0.6142	0.5294	0.5058	0.5586	0.6990	0.7683	0.5633
1.0e-5	0.6450	0.5447	0.5244	0.5964	0.7154	0.7775	0.5900
1.0e-6	0.6151	0.5092	NA	0.5499	0.7075	0.7863	0.5340
1.0e-7	0.5820	0.4441	NA	0.5347	0.7311	0.7790	NA

Table S27: AUC obtained by all the algorithms under the haplotype-based approach with 5-SNP haplotype length, additive genetic model and holdout sampling. Results for each p-value threshold (column 1) are shown. The maximum p-value threshold used was 0.15.

p-value threshold	BD	CAD	HT	IBD	RA	T1D	T2D
				NBC			
0.15	0.5872	0.4908	0.4893	0.5530	0.6386	0.6788	0.5894
0.1	0.5924	0.5100	0.4995	0.5439	0.6523	0.6882	0.5876
0.05	0.5940	0.5002	0.5045	0.5379	0.6700	0.7039	0.5819
0.01	0.6049	0.4801	0.4951	0.5420	0.6959	0.7195	0.5792
0.0010	0.6394	0.5064	0.4868	0.5513	0.7090	0.7206	0.5867
1.0e-4	0.6326	0.4808	0.5322	0.5699	0.7038	0.7229	0.5757
1.0e-5	0.6480	0.4773	0.5573	0.5883	0.6956	0.7237	0.5912
1.0e-6	0.6156	0.5013	NA	0.5500	0.6951	0.7346	0.5340
1.0e-7	0.5821	0.5535	NA	0.5347	0.7024	0.7391	NA
				sSVM			
0.15	0.5116	0.5000	0.5000	0.5072	0.5207	0.5359	0.5196
0.1	0.5151	0.5000	0.5000	0.5031	0.5290	0.5484	0.5254
0.05	0.5199	0.5000	0.5000	0.5067	0.5281	0.5524	0.5155
0.01	0.5252	0.4970	0.5125	0.5109	0.5359	0.5657	0.5166
0.0010	0.5226	0.4973	0.5020	0.5228	0.5535	0.6423	0.5278
1.0e-4	0.5326	0.5189	0.5007	0.5131	0.5750	0.6829	0.5190
1.0e-5	0.5368	0.5000	0.5000	0.5241	0.6075	0.7011	0.5206
1.0e-6	0.5218	0.5000	NA	0.5041	0.6055	0.7009	0.5000
1.0e-7	0.5058	0.5000	NA	0.5000	0.6009	0.7014	NA
				AdaBoostM1			
0.15	0.6144	0.5243	0.4969	0.5507	0.6672	0.7546	0.5623
0.1	0.6053	0.5234	0.4879	0.5708	0.6601	0.7622	0.5663
0.05	0.5931	0.5058	0.5125	0.5410	0.6729	0.7477	0.5432
0.01	0.5808	0.5025	0.5058	0.5293	0.6444	0.7010	0.5741
0.0010	0.6529	0.4942	0.4797	0.5529	0.6840	0.7248	0.5936
1.0e-4	0.6379	0.4742	0.5314	0.5640	0.7384	0.8160	0.5785
1.0e-5	0.6476	0.4612	0.5409	0.5940	0.7514	0.8318	0.5920
1.0e-6	0.6154	0.4806	NA	0.5499	0.7544	0.8321	0.5340
1.0e-7	0.5821	0.5213	NA	0.5347	0.7572	0.8296	NA
				C4.5			
0.15	0.5846	0.5000	0.5000	0.5348	0.5697	0.6061	0.5289
0.1	0.5672	0.5000	0.5000	0.5022	0.6091	0.6421	0.5232
0.05	0.6096	0.5000	0.5000	0.5296	0.5979	0.6404	0.5377
0.01	0.5884	0.4947	0.5015	0.5078	0.5930	0.6089	0.5155
0.0010	0.5650	0.4991	0.5002	0.5223	0.5754	0.6345	0.5323
1.0e-4	0.5640	0.4940	0.4974	0.5134	0.5695	0.6392	0.5280
1.0e-5	0.5915	0.5255	0.5000	0.5888	0.5314	0.6542	0.5728
1.0e-6	0.5800	0.5000	NA	0.5499	0.5597	0.6353	0.5000
1.0e-7	0.5423	0.5000	NA	0.5000	0.6158	0.6249	NA
				20RF			
0.15	0.5196	0.5049	0.5014	0.5096	0.4899	0.5296	0.5004
0.1	0.4869	0.4871	0.4962	0.5086	0.5153	0.5538	0.4982
0.05	0.5131	0.5111	0.5114	0.5340	0.5376	0.5188	0.4934
0.01	0.5047	0.5021	0.4620	0.5155	0.5506	0.6443	0.5128
0.0010	0.5547	0.5058	0.5269	0.5042	0.6785	0.7531	0.5665
1.0e-4	0.6134	0.5299	0.5050	0.5583	0.6997	0.7617	0.5634
1.0e-5	0.6421	0.5422	0.5243	0.5940	0.7152	0.7714	0.5888
1.0e-6	0.6152	0.5070	NA	0.5499	0.7076	0.7801	0.5340
1.0e-7	0.5820	0.4441	NA	0.5347	0.7317	0.7740	NA

Table S28: AUC obtained by all the algorithms under the haplotype-based approach with 5-SNP haplotype length, dominant genetic model and holdout sampling. Results for each p-value threshold (column 1) are shown. The maximum p-value threshold used was 0.15.

p-value threshold	BD	CAD	HT	IBD	RA	T1D	T2D
			NBC				
0.15	0.6304	0.4927	0.4928	0.5641	0.6587	0.6923	0.5911
0.1	0.6253	0.5047	0.5034	0.5619	0.6617	0.6993	0.5816
0.05	0.6087	0.4934	0.5002	0.5501	0.6727	0.7201	0.5760
0.01	0.6120	0.4903	0.4974	0.5305	0.6851	0.7555	0.5803
0.0010	0.6224	0.5106	0.4826	0.5372	0.6852	0.7680	0.5850
1.0e-4	0.6347	0.4827	0.5371	0.5601	0.6784	0.7708	0.5767
1.0e-5	0.6470	0.4791	0.5573	0.5873	0.6702	0.7714	0.5912
1.0e-6	0.6157	0.5019	NA	0.5499	0.6660	0.7758	0.5340
1.0e-7	0.5821	0.5535	NA	0.5347	0.6765	0.7757	NA
			sSVM				
0.15	0.5578	0.5000	0.5000	0.5230	0.5643	0.6055	0.5468
0.1	0.5728	0.5000	0.5000	0.5241	0.5790	0.6077	0.5463
0.05	0.5432	0.5000	0.5000	0.5301	0.5836	0.5929	0.5221
0.01	0.5419	0.4958	0.5154	0.5177	0.5735	0.6387	0.5273
0.0010	0.5380	0.5053	0.4906	0.5159	0.6099	0.6881	0.5347
1.0e-4	0.5611	0.4955	0.5174	0.5282	0.6449	0.7320	0.5247
1.0e-5	0.5850	0.5000	0.5000	0.5479	0.6514	0.7472	0.5354
1.0e-6	0.5780	0.5000	NA	0.5163	0.6484	0.7501	0.5000
1.0e-7	0.5420	0.5000	NA	0.5000	0.6456	0.7403	NA
			AdaBoostM1				
0.15	0.5981	0.5117	0.5077	0.5405	0.6522	0.7672	0.5569
0.1	0.6024	0.5160	0.4771	0.5638	0.6600	0.7654	0.5696
0.05	0.5854	0.4984	0.5065	0.5270	0.6346	0.7554	0.5480
0.01	0.5655	0.4979	0.5095	0.5004	0.6254	0.7103	0.5544
0.0010	0.6294	0.5025	0.4662	0.5336	0.6729	0.7245	0.5920
1.0e-4	0.6417	0.4798	0.5376	0.5499	0.7371	0.8143	0.5805
1.0e-5	0.6475	0.4681	0.5409	0.6009	0.7391	0.8334	0.5912
1.0e-6	0.6154	0.4834	NA	0.5499	0.7394	0.8350	0.5340
1.0e-7	0.5821	0.5213	NA	0.5347	0.7431	0.8339	NA
			C4.5				
0.15	0.6017	0.5000	0.5000	0.5006	0.5733	0.6205	0.5374
0.1	0.5817	0.5000	0.5000	0.5205	0.6321	0.6326	0.5461
0.05	0.6038	0.5000	0.5000	0.5137	0.6234	0.6178	0.5630
0.01	0.6223	0.4992	0.5000	0.4974	0.6204	0.6356	0.5386
0.0010	0.5641	0.5000	0.5000	0.5281	0.6042	0.6314	0.5460
1.0e-4	0.5856	0.4993	0.5116	0.5049	0.6070	0.6674	0.5533
1.0e-5	0.5925	0.5341	0.5000	0.5954	0.6185	0.6717	0.5709
1.0e-6	0.5799	0.5000	NA	0.5499	0.6062	0.6467	0.5000
1.0e-7	0.5424	0.5000	NA	0.5000	0.6266	0.6479	NA
			20RF				
0.15	0.5187	0.5020	0.5024	0.5097	0.4909	0.5302	0.5010
0.1	0.4873	0.4874	0.4967	0.5084	0.5155	0.5537	0.4990
0.05	0.5108	0.5107	0.5103	0.5370	0.5366	0.5168	0.4937
0.01	0.5063	0.5023	0.4611	0.5194	0.5514	0.6427	0.5133
0.0010	0.5548	0.5039	0.5284	0.5036	0.6777	0.7618	0.5684
1.0e-4	0.6140	0.5292	0.5070	0.5573	0.6969	0.7756	0.5622
1.0e-5	0.6452	0.5457	0.5246	0.5971	0.7141	0.7833	0.5886
1.0e-6	0.6151	0.5087	NA	0.5499	0.7059	0.7918	0.5340
1.0e-7	0.5820	0.4441	NA	0.5347	0.7277	0.7837	NA

Table S29: AUC obtained by all the algorithms under the haplotype-based approach with 5-SNP haplotype length, recessive genetic model and holdout sampling. Results for each p-value threshold (column 1) are shown. The maximum p-value threshold used was 0.15.

p-value threshold	BD	CAD	HT	IBD	RA	T1D	T2D
				NBC			
0.15	0.8480	0.0000	0.0000	0.4176	0.8624	0.7859	0.7422
0.1	0.8298	0.0000	0.0000	0.1167	0.8452	0.7829	0.7256
0.05	0.8073	0.0000	0.0000	0.0858	0.8161	0.7431	0.6840
0.01	0.7302	0.0000	0.0000	0.0069	0.7591	0.6585	0.6414
0.0010	0.6381	0.0000	0.0000	0.5767	0.7097	0.5953	0.5759
1.0e-4	0.6424	0.0000	0.0000	0.6224	0.6763	0.5953	0.6123
1.0e-5	0.7505	0.5794	0.0000	0.5503	0.6720	0.6310	0.6902
1.0e-6	NA	0.9481	NA	NA	0.6742	0.6442	0.8628
1.0e-7	NA	0.7321	NA	NA	0.6688	0.6942	NA
				sSVM			
0.15	0.9938	1,0000	1,0000	0.8818	0.9926	0.9910	0.9376
0.1	0.9671	1,0000	1,0000	0.4107	0.9679	0.9556	0.8794
0.05	0.9309	1,0000	1,0000	0.3567	0.9288	0.9258	0.8360
0.01	0.8720	0.9950	0.9965	0.0361	0.8807	0.8863	0.7996
0.0010	0.7490	0.4494	0.1956	0.7615	0.8515	0.8833	0.7000
1.0e-4	0.8468	0.7830	0.7329	0.7158	0.8353	0.8956	0.8164
1.0e-5	0.9331	0.9437	0.0000	0.7318	0.8267	0.8713	0.6133
1.0e-6	NA	0.9920	NA	NA	0.8353	0.8775	0.9498
1.0e-7	NA	0.9989	NA	NA	0.8248	0.8735	NA
				AdaBoostM1			
0.15	0.7056	0.0997	0.0400	0.0538	0.7699	0.7910	0.6310
0.1	0.7141	0.9003	0.2285	0.0938	0.7645	0.7941	0.6528
0.05	0.7002	0.8930	0.4098	0.2574	0.7602	0.7880	0.6424
0.01	0.7088	0.1589	0.0676	0.4371	0.7441	0.7788	0.6372
0.0010	0.6531	0.6957	0.2428	0.8318	0.7527	0.7441	0.5894
1.0e-4	0.7430	0.3313	0.0164	0.6259	0.7785	0.8247	0.6809
1.0e-5	0.7505	0.8172	0.0000	0.8066	0.7667	0.8277	0.7017
1.0e-6	NA	0.7269	NA	NA	0.7495	0.8318	0.8701
1.0e-7	NA	0.7321	NA	NA	0.7559	0.8400	NA
				C4.5			
0.15	0.5426	0.3061	1,0000	0.6512	0.6614	0.5855	0.5487
0.1	0.5531	0.9731	0.8559	0.4541	0.6924	0.6712	0.6192
0.05	0.5904	0.9989	0.0046	0.3818	0.6477	0.6811	0.5179
0.01	0.5762	0.9929	0.2110	0.4512	0.6583	0.5937	0.5648
0.0010	0.5840	0.6102	0.8357	0.1879	0.7041	0.6439	0.5929
1.0e-4	0.6419	0.4864	0.0000	0.5530	0.7554	0.7132	0.6414
1.0e-5	0.8405	0.8265	0.0000	0.8513	0.7338	0.6982	0.7609
1.0e-6	NA	0.9969	NA	NA	0.7431	0.7223	0.6497
1.0e-7	NA	0.9595	NA	NA	0.7410	0.7322	NA
				20RF			
0.15	0.9893	0.5701	0.4693	0.9245	0.9914	0.9898	0.9844
0.1	0.9914	0.7726	0.7930	0.8902	0.9914	0.9990	0.9854
0.05	0.9914	0.8422	0.7746	0.9485	0.9925	0.9939	0.9740
0.01	0.9882	0.8505	0.5666	0.8078	0.9935	0.9918	0.9761
0.0010	0.9711	0.7591	0.7797	0.9931	0.9473	0.9460	0.9158
1.0e-4	0.9069	0.9128	0.3432	0.7643	0.8634	0.9021	0.7245
1.0e-5	0.9090	0.6802	0.0000	0.8089	0.8301	0.8746	0.7048
1.0e-6	NA	1,0000	NA	NA	0.8129	0.8756	0.8628
1.0e-7	NA	0.9688	NA	NA	0.8441	0.8634	NA

Table S 30: sensitivity obtained by all the algorithms under the haplotype-based approach with 1-SNP haplotype length, additive genetic model and holdout sampling. Results for each p-value threshold (column 1) are shown. The maximum p-value threshold used was 0.15.

p-value threshold	BD	CAD	HT	IBD	RA	T1D	T2D
				NBC			
0.15	0.9336	0.0000	0.0000	0.6636	0.9452	0.9042	0.8773
0.1	0.9315	0.0000	0.0000	0.2998	0.9409	0.8940	0.8680
0.05	0.9229	0.0000	0.0000	0.2883	0.9237	0.8797	0.8617
0.01	0.8822	0.0000	0.0000	0.0618	0.8882	0.8043	0.8451
0.0010	0.8490	0.0000	0.0000	0.8204	0.8462	0.6911	0.8150
1.0e-4	0.9293	0.0509	0.0113	0.8993	0.8140	0.6840	0.9449
1.0e-5	0.9989	0.9875	0.0359	0.9817	0.8065	0.7176	0.9958
1.0e-6	NA	1,0000	NA	NA	0.8065	0.7492	1,0000
1.0e-7	NA	1,0000	NA	NA	0.8151	0.8063	NA
				sSVM			
0.15	0.9946	1,0000	1,0000	0.9325	0.9935	0.9929	0.9574
0.1	0.9754	1,0000	1,0000	0.6979	0.9763	0.9684	0.9252
0.05	0.9540	1,0000	1,0000	0.6533	0.9527	0.9511	0.9054
0.01	0.9261	0.9958	0.9969	0.2975	0.9290	0.9256	0.8898
0.0010	0.8651	0.7061	0.5492	0.8638	0.9065	0.9195	0.8347
1.0e-4	0.9090	0.8858	0.8596	0.8432	0.8828	0.9225	0.8929
1.0e-5	0.9518	0.9595	0.0359	0.8524	0.8731	0.9021	0.8067
1.0e-6	NA	0.9927	NA	NA	0.8817	0.9062	0.9657
1.0e-7	NA	0.9990	NA	NA	0.8753	0.9052	NA
				AdaBoostM1			
0.15	0.8726	0.3074	0.2162	0.2506	0.9118	0.9062	0.8337
0.1	0.8876	0.9688	0.5020	0.3021	0.8935	0.9164	0.8295
0.05	0.8715	0.9637	0.6752	0.5744	0.8925	0.9134	0.8586
0.01	0.8897	0.4226	0.2869	0.7048	0.8935	0.8960	0.8462
0.0010	0.8694	0.9117	0.6230	0.9439	0.9194	0.9093	0.8514
1.0e-4	0.9754	0.8453	0.2766	0.9542	0.9688	0.9511	0.9896
1.0e-5	0.9989	0.9979	0.0359	1,0000	0.9957	0.9633	0.9990
1.0e-6	NA	1,0000	NA	NA	0.9860	0.9704	1,0000
1.0e-7	NA	1,0000	NA	NA	0.9957	0.9745	NA
				C4.5			
0.15	0.8009	0.3198	1,0000	0.8192	0.8323	0.8675	0.8035
0.1	0.8287	0.9803	0.9180	0.7414	0.8473	0.8685	0.8274
0.05	0.8458	0.9990	0.1045	0.6888	0.8570	0.8583	0.8025
0.01	0.8169	0.9938	0.5594	0.7449	0.8269	0.8481	0.7890
0.0010	0.7966	0.9304	0.9898	0.4222	0.8763	0.8542	0.8326
1.0e-4	0.9336	0.8255	0.0359	0.8719	0.8796	0.8695	0.9543
1.0e-5	1,0000	0.9772	0.0359	1,0000	0.9065	0.9144	1,0000
1.0e-6	NA	1,0000	NA	NA	0.9022	0.9001	1,0000
1.0e-7	NA	1,0000	NA	NA	0.9290	0.9246	NA
				20RF			
0.15	1,0000	1,0000	1,0000	1,0000	1,0000	1,0000	1,0000
0.1	1,0000	1,0000	1,0000	1,0000	1,0000	1,0000	1,0000
0.05	1,0000	1,0000	1,0000	1,0000	1,0000	1,0000	1,0000
0.01	1,0000	1,0000	1,0000	1,0000	1,0000	1,0000	1,0000
0.0010	1,0000	1,0000	1,0000	1,0000	1,0000	1,0000	1,0000
1.0e-4	1,0000	1,0000	1,0000	1,0000	1,0000	1,0000	1,0000
1.0e-5	0.9989	0.9917	0.0359	1,0000	1,0000	1,0000	0.9979
1.0e-6	NA	1,0000	NA	NA	1,0000	1,0000	1,0000
1.0e-7	NA	1,0000	NA	NA	1,0000	0.9990	NA

Table S 31: sensitivity obtained by all the algorithms under the haplotype-based approach with 1-SNP haplotype length, dominant genetic model and holdout sampling. Results for each p-value threshold (column 1) are shown. The maximum p-value threshold used was 0.15.

p-value threshold	BD	CAD	HT	IBD	RA	T1D	T2D
				NBC			
0.15	0.6103	0.0000	0.0000	0.1911	0.6000	0.5301	0.4397
0.1	0.5889	0.0000	0.0000	0.0332	0.5946	0.4944	0.4387
0.05	0.5107	0.0000	0.0000	0.0263	0.5731	0.4536	0.4148
0.01	0.4368	0.0000	0.0000	0.0046	0.5312	0.4037	0.3617
0.0010	0.3158	0.0000	0.0000	0.2712	0.5559	0.3670	0.3077
1.0e-4	0.2173	0.0000	0.0000	0.2815	0.5484	0.4057	0.1518
1.0e-5	0.0000	0.0000	0.0000	0.0000	0.5258	0.4353	0.0717
1.0e-6	NA	0.0000	NA	NA	0.5247	0.4455	0.0000
1.0e-7	NA	0.0000	NA	NA	0.5172	0.4832	NA
				sSVM			
0.15	0.8651	1,0000	1,0000	0.5034	0.8624	0.7849	0.6403
0.1	0.7248	1,0000	1,0000	0.2105	0.7129	0.6799	0.5457
0.05	0.6199	1,0000	1,0000	0.1922	0.6172	0.6106	0.4823
0.01	0.5032	0.8328	0.8852	0.0263	0.5237	0.5800	0.4397
0.0010	0.4026	0.2399	0.1096	0.4348	0.5366	0.6096	0.3857
1.0e-4	0.5032	0.4123	0.3852	0.3947	0.5946	0.6646	0.4761
1.0e-5	0.6724	0.6791	0.0000	0.4027	0.6054	0.6626	0.3067
1.0e-6	NA	0.9024	NA	NA	0.6000	0.6718	0.6497
1.0e-7	NA	0.9678	NA	NA	0.5871	0.6544	NA
				AdaBoostM1			
0.15	0.4272	0.0322	0.0072	0.0195	0.5280	0.5321	0.3534
0.1	0.4272	0.6864	0.0768	0.0309	0.5011	0.5484	0.3565
0.05	0.4122	0.6656	0.1895	0.0892	0.5011	0.5209	0.3669
0.01	0.4047	0.0530	0.0225	0.1911	0.4817	0.5311	0.3669
0.0010	0.3458	0.3645	0.0492	0.5789	0.4774	0.4557	0.2859
1.0e-4	0.1542	0.0343	0.0000	0.1281	0.3871	0.5046	0.1008
1.0e-5	0.0000	0.0332	0.0000	0.0011	0.3011	0.4964	0.0353
1.0e-6	NA	0.0000	NA	NA	0.2742	0.4893	0.0000
1.0e-7	NA	0.0000	NA	NA	0.2538	0.4944	NA
				C4.5			
0.15	0.3116	0.0353	0.9252	0.3375	0.3688	0.3741	0.2942
0.1	0.3094	0.7113	0.4867	0.2208	0.4032	0.3833	0.3576
0.05	0.3469	0.9470	0.0041	0.1922	0.3892	0.4220	0.2827
0.01	0.3469	0.8723	0.1178	0.2517	0.4032	0.3986	0.2931
0.0010	0.3405	0.5421	0.3863	0.0469	0.4484	0.4149	0.3441
1.0e-4	0.3704	0.3250	0.0000	0.3330	0.4720	0.4832	0.1570
1.0e-5	0.0000	0.0768	0.0000	0.0000	0.5280	0.6055	0.0052
1.0e-6	NA	0.2565	NA	NA	0.4839	0.5607	0.0000
1.0e-7	NA	0.0000	NA	NA	0.4763	0.5963	NA
				20RF			
0.15	0.0000	0.0000	0.0000	0.0000	0.0000	0.0000	0.0000
0.1	0.0000	0.0000	0.0010	0.0000	0.0000	0.0000	0.0000
0.05	0.0000	0.0010	0.0000	0.0000	0.0000	0.0000	0.0000
0.01	0.0000	0.0010	0.0000	0.0000	0.0000	0.0000	0.0000
0.0010	0.0000	0.0021	0.0010	0.0000	0.0011	0.0071	0.0000
1.0e-4	0.0086	0.0052	0.0000	0.0000	0.0817	0.0785	0.0208
1.0e-5	0.0278	0.0322	0.0000	0.0000	0.1968	0.1417	0.0509
1.0e-6	NA	0.4029	NA	NA	0.1978	0.1213	0.0000
1.0e-7	NA	0.0000	NA	NA	0.2484	0.1886	NA

Table S 32: Sensitivity obtained by all the algorithms under the haplotype-based approach with 1-SNP haplotype length, recessive genetic model and holdout sampling. Results for each p-value threshold (column 1) are shown. The maximum p-value threshold used was 0.15.

p-value threshold	BD	CAD	HT	IBD	RA	T1D	T2D
				NBC			
0.15	0.8672	0.0000	0.0000	0.5892	0.8667	0.8379	0.7484
0.1	0.8469	0.0000	0.0000	0.3787	0.8570	0.8124	0.7588
0.05	0.8276	0.0000	0.0000	0.0561	0.8237	0.7819	0.7464
0.01	0.7794	0.0000	0.0000	0.0011	0.7645	0.6871	0.6819
0.0010	0.7537	0.0000	0.0000	0.6968	0.7161	0.6055	0.6559
1.0e-4	0.7580	0.0021	0.0410	0.6625	0.6828	0.6188	0.6840
1.0e-5	0.8597	0.3313	0.9795	0.7883	0.6806	0.6422	0.6944
1.0e-6	0.9069	0.1890	0.9795	0.9897	0.6753	0.6534	0.6486
1.0e-7	0.9400	0.0145	NA	1,0000	0.6785	0.6779	NA
				sSVM			
0.15	0.9832	1,0000	1,0000	0.9683	0.9881	0.9873	0.9412
0.1	0.9628	1,0000	1,0000	0.8173	0.9544	0.9644	0.8900
0.05	0.9094	1,0000	1,0000	0.3701	0.9210	0.9331	0.8670
0.01	0.8400	0.9857	0.9227	0.0274	0.8936	0.8934	0.7834
0.0010	0.7707	0.8657	0.0081	0.8376	0.8554	0.8690	0.7412
1.0e-4	0.8618	1,0000	0.1702	0.7364	0.8460	0.8994	0.8339
1.0e-5	0.9864	0.0176	1,0000	0.7059	0.8229	0.9031	0.7902
1.0e-6	0.9929	0.0277	1,0000	0.9988	0.8228	0.8898	1,0000
1.0e-7	1,0000	0.0176	NA	1,0000	0.8193	0.8813	NA
				AdaBoostM1			
0.15	0.7323	0.8141	0.5184	0.1945	0.7710	0.8114	0.6622
0.1	0.7655	0.1236	0.9457	0.1682	0.7742	0.8104	0.6445
0.05	0.7591	0.0758	0.2080	0.1064	0.7839	0.8073	0.6559
0.01	0.7206	0.0031	0.1527	0.1201	0.7484	0.7829	0.6694
0.0010	0.7452	0.0010	0.0010	0.6007	0.7796	0.7666	0.6632
1.0e-4	0.7441	0.0395	0.5256	0.6533	0.8022	0.8206	0.7131
1.0e-5	0.8448	0.0000	0.9816	0.7632	0.7925	0.8196	0.8046
1.0e-6	0.9079	0.2887	0.9795	0.9908	0.7806	0.8257	0.9938
1.0e-7	0.9400	0.0145	NA	1,0000	0.7624	0.8359	NA
				C4.5			
0.15	0.5556	0.0000	0.0000	0.0000	0.6027	0.5303	0.6405
0.1	0.6979	0.0000	0.0000	0.0000	0.6005	0.6032	0.6450
0.05	0.6704	0.0000	0.0000	0.0000	0.5661	0.6343	0.6782
0.01	0.6776	0.0000	0.0000	0.9953	0.6674	0.7154	0.6605
0.0010	0.6840	0.3064	0.0153	0.6141	0.7623	0.6512	0.6025
1.0e-4	0.6999	0.0000	0.8483	0.5565	0.6903	0.6840	0.6907
1.0e-5	0.8330	0.6767	0.9795	0.8066	0.7252	0.6780	0.8191
1.0e-6	0.9036	0.8370	0.9795	0.9886	0.7433	0.6489	0.9948
1.0e-7	0.9379	0.0187	NA	1,0000	0.7356	0.6990	NA
				20RF			
0.15	0.9914	0.9450	0.9559	0.7071	0.9946	0.9929	0.9636
0.1	0.9893	0.5379	0.6455	0.8924	0.9914	0.9867	0.9699
0.05	0.9893	0.9450	0.7992	0.4451	0.9925	0.9980	0.9636
0.01	0.9882	0.5691	0.9785	0.7609	0.9871	0.9857	0.9751
0.0010	0.9850	0.4673	0.4785	0.9691	0.9581	0.9307	0.9491
1.0e-4	0.9529	0.7362	0.2725	0.8009	0.8624	0.9235	0.8815
1.0e-5	0.9443	0.1817	0.9836	0.8249	0.8495	0.8919	0.7796
1.0e-6	0.9079	0.1412	0.9795	0.9908	0.8419	0.8940	0.9938
1.0e-7	0.9400	0.0187	NA	1,0000	0.8344	0.8879	NA

Table S 33: sensitivity obtained by all the algorithms under the haplotype-based approach with 2-SNP haplotype length, additive genetic model and holdout sampling. Results for each p-value threshold (column 1) are shown. The maximum p-value threshold used was 0.15.

p-value threshold	BD	CAD	HT	IBD	RA	T1D	T2D
				NBC			
0.15	0.9411	0.0000	0.0000	0.7792	0.9419	0.9276	0.8992
0.1	0.9454	0.0000	0.0000	0.6190	0.9505	0.9134	0.8992
0.05	0.9315	0.0000	0.0000	0.2094	0.9355	0.8919	0.8846
0.01	0.9047	0.0000	0.0000	0.0366	0.8968	0.8308	0.8576
0.0010	0.9015	0.0000	0.0000	0.8856	0.8495	0.7125	0.8732
1.0e-4	0.9240	0.0789	0.3043	0.9188	0.8237	0.6993	0.9470
1.0e-5	0.9839	0.9034	1,0000	0.9760	0.8108	0.7187	0.9938
1.0e-6	0.9946	0.9242	1,0000	1,0000	0.8086	0.7390	0.9969
1.0e-7	1,0000	1,0000	NA	1,0000	0.8151	0.7737	NA
				sSVM			
0.15	0.9861	1,0000	1,0000	0.9760	0.9903	0.9898	0.9615
0.1	0.9732	1,0000	1,0000	0.8913	0.9667	0.9755	0.9314
0.05	0.9390	1,0000	1,0000	0.6670	0.9473	0.9572	0.9220
0.01	0.9058	0.9886	0.9488	0.2677	0.9355	0.9327	0.8805
0.0010	0.8694	0.9190	0.1230	0.9062	0.9108	0.9113	0.8628
1.0e-4	0.9186	1,0000	0.5205	0.8558	0.8957	0.9256	0.9054
1.0e-5	0.9882	0.1880	1,0000	0.8455	0.8688	0.9266	0.8888
1.0e-6	0.9936	0.2721	1,0000	0.9989	0.8699	0.9174	1,0000
1.0e-7	1,0000	0.1880	NA	1,0000	0.8688	0.9113	NA
				AdaBoostM1			
0.15	0.9069	0.9398	0.7664	0.4828	0.9054	0.9205	0.8638
0.1	0.9036	0.3936	0.9867	0.4405	0.8968	0.9164	0.8482
0.05	0.9026	0.2991	0.4652	0.3375	0.9011	0.9083	0.8482
0.01	0.8726	0.0758	0.4262	0.3696	0.9022	0.9032	0.8597
0.0010	0.9079	0.0623	0.0164	0.8227	0.9280	0.9154	0.8898
1.0e-4	0.9475	0.3292	0.9887	0.9737	0.9527	0.9562	0.9802
1.0e-5	0.9904	0.0021	1,0000	0.9977	0.9806	0.9694	1,0000
1.0e-6	0.9957	0.9429	1,0000	1,0000	0.9710	0.9745	1,0000
1.0e-7	1,0000	1,0000	NA	1,0000	0.9785	0.9725	NA
				C4.5			
0.15	0.8662	0.0000	0.0000	0.0721	0.8871	0.8705	0.8295
0.1	0.8469	0.0000	0.0000	0.0721	0.8796	0.8736	0.8067
0.05	0.8576	0.0000	0.0000	0.0812	0.8839	0.8838	0.8004
0.01	0.8373	0.0000	0.0000	0.9954	0.8634	0.8502	0.7900
0.0010	0.8522	0.6262	0.2018	0.7838	0.8849	0.9062	0.8233
1.0e-4	0.8908	0.0000	0.9877	0.8593	0.9000	0.8950	0.8971
1.0e-5	0.9872	0.9979	1,0000	0.9954	0.9054	0.9042	0.9990
1.0e-6	0.9946	0.9979	1,0000	0.9989	0.8892	0.9021	1,0000
1.0e-7	1,0000	1,0000	NA	1,0000	0.8839	0.9266	NA
				20RF			
0.15	1,0000	1,0000	1,0000	1,0000	1,0000	1,0000	1,0000
0.1	1,0000	0.9990	1,0000	1,0000	1,0000	1,0000	1,0000
0.05	1,0000	1,0000	1,0000	1,0000	1,0000	1,0000	1,0000
0.01	1,0000	1,0000	1,0000	1,0000	1,0000	1,0000	1,0000
0.0010	1,0000	1,0000	1,0000	1,0000	1,0000	1,0000	1,0000
1.0e-4	1,0000	1,0000	1,0000	1,0000	1,0000	1,0000	1,0000
1.0e-5	0.9979	1,0000	1,0000	0.9966	1,0000	1,0000	1,0000
1.0e-6	0.9957	1,0000	1,0000	1,0000	0.9989	1,0000	1,0000
1.0e-7	1,0000	0.9823	NA	1,0000	1,0000	0.9980	NA

Table S 34: Sensitivity obtained by all the algorithms under the haplotype-based approach with 2-SNP haplotype length, dominant genetic model and holdout sampling. Results for each p-value threshold (column 1) are shown. The maximum p-value threshold used was 0.15.

p-value threshold	BD	CAD	HT	IBD	RA	T1D	T2D
				NBC			
0.15	0.6445	0.0000	0.0000	0.3215	0.6312	0.5780	0.4958
0.1	0.6221	0.0000	0.0000	0.1728	0.6290	0.5433	0.4917
0.05	0.5996	0.0000	0.0000	0.0183	0.6065	0.5015	0.4927
0.01	0.5128	0.0000	0.0000	0.0011	0.5667	0.4444	0.4335
0.0010	0.4615	0.0000	0.0000	0.3947	0.5785	0.4016	0.3701
1.0e-4	0.3844	0.0000	0.0000	0.2860	0.5570	0.4200	0.2277
1.0e-5	0.2773	0.0083	0.0061	0.0549	0.5398	0.4424	0.1040
1.0e-6	0.0921	0.0000	0.0000	0.0698	0.5247	0.4546	0.0000
1.0e-7	0.0931	0.0000	NA	0.0721	0.5237	0.4720	NA
				sSVM			
0.15	0.8137	1,0000	1,0000	0.7334	0.8065	0.7910	0.6154
0.1	0.6927	1,0000	1,0000	0.4863	0.6978	0.6626	0.5551
0.05	0.6124	1,0000	1,0000	0.1957	0.6140	0.5973	0.5083
0.01	0.4946	0.7871	0.6117	0.0206	0.5419	0.5637	0.4324
0.0010	0.4390	0.5223	0.0072	0.4840	0.5280	0.5882	0.3929
1.0e-4	0.5075	0.9367	0.0984	0.4027	0.5731	0.6656	0.4751
1.0e-5	0.8544	0.0145	0.9795	0.3707	0.6097	0.6840	0.4189
1.0e-6	0.9036	0.0208	0.9795	0.9886	0.6043	0.6667	0.9948
1.0e-7	0.9379	0.0145	NA	1,0000	0.5946	0.6585	NA
				AdaBoostM1			
0.15	0.4443	0.5421	0.2643	0.0629	0.5301	0.5810	0.3846
0.1	0.4593	0.0436	0.7480	0.0618	0.5441	0.5647	0.3888
0.05	0.4690	0.0197	0.0809	0.0297	0.5398	0.5759	0.3971
0.01	0.4507	0.0010	0.0523	0.0366	0.5032	0.5138	0.3805
0.0010	0.4347	0.0000	0.0000	0.3112	0.5323	0.5066	0.3451
1.0e-4	0.2976	0.0010	0.0881	0.1373	0.4796	0.5260	0.1840
1.0e-5	0.2366	0.0000	0.1773	0.0412	0.4022	0.5178	0.0333
1.0e-6	0.0921	0.0135	0.0000	0.0709	0.3763	0.5168	0.0062
1.0e-7	0.0931	0.0000	NA	0.0721	0.3376	0.5127	NA
				C4.5			
0.15	0.4079	0.0000	0.0000	0.0000	0.4903	0.4659	0.3981
0.1	0.3897	0.0000	0.0000	0.0000	0.4323	0.4241	0.3805
0.05	0.3940	0.0000	0.0000	0.0000	0.4237	0.4322	0.3441
0.01	0.4197	0.0000	0.0000	0.8822	0.4441	0.3986	0.3139
0.0010	0.3940	0.1433	0.0133	0.3192	0.5054	0.5005	0.3514
1.0e-4	0.4293	0.0000	0.8248	0.3558	0.5011	0.4801	0.3451
1.0e-5	0.2141	0.1952	0.0000	0.0412	0.5624	0.5382	0.0156
1.0e-6	0.0000	0.3406	0.0000	0.0000	0.5323	0.5494	0.0000
1.0e-7	0.0000	0.0000	NA	0.0000	0.4978	0.5831	NA
				20RF			
0.15	0.0000	0.0083	0.0215	0.0000	0.0000	0.0000	0.0000
0.1	0.0000	0.0000	0.0010	0.0000	0.0000	0.0000	0.0000
0.05	0.0000	0.0073	0.0010	0.0000	0.0000	0.0000	0.0000
0.01	0.0000	0.0000	0.0225	0.0000	0.0000	0.0000	0.0000
0.0010	0.0000	0.0000	0.0000	0.0011	0.0000	0.0122	0.0000
1.0e-4	0.0000	0.0000	0.0000	0.0011	0.0817	0.0418	0.0031
1.0e-5	0.0375	0.0083	0.0379	0.0400	0.1548	0.1111	0.0198
1.0e-6	0.0910	0.0000	0.0000	0.0709	0.1968	0.1223	0.0062
1.0e-7	0.0931	0.0000	NA	0.0721	0.2387	0.1957	NA

Table S 35: sensitivity obtained by all the algorithms under the haplotype-based approach with 2-SNP haplotype length, recessive genetic model and holdout sampling. Results for each p-value threshold (column 1) are shown. The maximum p-value threshold used was 0.15.

p-value threshold	BD	CAD	HT	IBD	RA	T1D	T2D
				NBC			
0.15	0.8555	0.0000	0.0000	0.0343	0.8613	0.8216	0.7484
0.1	0.8405	0.0000	0.0000	0.0801	0.8452	0.8002	0.7277
0.05	0.8233	0.0000	0.0000	0.0881	0.8086	0.7635	0.7048
0.01	0.7752	0.0000	0.0000	0.0904	0.7581	0.6718	0.6819
0.0010	0.7505	0.0000	0.0809	0.5973	0.7140	0.6106	0.6383
1.0e-4	0.7827	0.0779	0.0000	0.6499	0.6828	0.6310	0.6663
1.0e-5	0.8672	0.9585	0.0277	0.7609	0.6677	0.6381	0.7006
1.0e-6	0.8951	0.9834	1,0000	0.7689	0.6634	0.6514	0.8503
1.0e-7	0.9454	0.9782	NA	1,0000	0.6581	0.6779	NA
				sSVM			
0.15	0.9741	1,0000	1,0000	0.5188	0.9743	0.9799	0.9063
0.1	0.9515	1,0000	1,0000	0.8585	0.9592	0.9551	0.8698
0.05	0.9085	1,0000	1,0000	0.6826	0.9340	0.9180	0.8385
0.01	0.8559	0.3730	0.4442	0.6236	0.8746	0.8989	0.7763
0.0010	0.7639	0.1230	0.2888	0.5258	0.8534	0.8740	0.7002
1.0e-4	0.8606	1,0000	0.8480	0.7661	0.8285	0.9123	0.8019
1.0e-5	0.9879	0.0508	0.0000	0.9476	0.8018	0.8996	0.9480
1.0e-6	0.9870	0.2175	1,0000	0.9792	0.8272	0.8954	0.9744
1.0e-7	0.9989	1,0000	NA	1,0000	0.8208	0.8823	NA
				AdaBoostM1			
0.15	0.7259	0.0530	0.8432	0.2334	0.7645	0.7666	0.6486
0.1	0.7430	0.5275	0.2848	0.5103	0.7495	0.7819	0.6601
0.05	0.7355	0.0800	0.1629	0.3375	0.7591	0.7839	0.6611
0.01	0.7527	0.1402	0.0072	0.6556	0.7527	0.7666	0.6320
0.0010	0.7291	0.5919	0.6455	0.2162	0.7366	0.7747	0.6372
1.0e-4	0.7473	0.0000	0.0000	0.6659	0.7710	0.8216	0.7017
1.0e-5	0.8094	0.6771	0.0266	0.7094	0.7688	0.8338	0.7869
1.0e-6	0.9015	0.6750	1,0000	0.7700	0.7806	0.8338	0.8503
1.0e-7	0.9454	0.9782	NA	1,0000	0.7688	0.8247	NA
				C4.5			
0.15	0.6763	0.0000	0.0000	0.0000	0.6710	0.6507	0.6652
0.1	0.7215	0.0000	0.0000	0.0000	0.6705	0.6678	0.6686
0.05	0.7401	0.0000	0.0000	0.0286	0.7225	0.6582	0.6392
0.01	0.7325	0.0000	0.0000	0.3000	0.6891	0.6197	0.6505
0.0010	0.6440	0.1055	0.7599	0.6259	0.7593	0.6328	0.6297
1.0e-4	0.6580	0.0000	0.5873	0.6002	0.7304	0.6810	0.6366
1.0e-5	0.8542	0.1701	0.0000	0.7414	0.7202	0.6731	0.8191
1.0e-6	0.8972	0.5545	1,0000	0.7700	0.7572	0.7108	0.7609
1.0e-7	0.9433	1,0000	NA	1,0000	0.7318	0.7308	NA
				20RF			
0.15	0.9893	0.8775	0.8627	0.9165	0.9860	0.9888	0.9595
0.1	0.9914	0.5670	0.4109	0.9451	0.9946	0.9918	0.9688
0.05	0.9850	0.8474	0.8709	0.8227	0.9914	0.9939	0.9699
0.01	0.9914	0.8837	0.9631	0.8181	0.9828	0.9939	0.9802
0.0010	0.9829	0.5691	0.7449	0.8101	0.9258	0.9490	0.9470
1.0e-4	0.9550	0.2150	0.7254	0.8330	0.8731	0.9144	0.8992
1.0e-5	0.9336	0.1952	0.0717	0.7941	0.8538	0.8930	0.8056
1.0e-6	0.9004	0.1693	0.9887	0.7689	0.8376	0.8981	0.8503
1.0e-7	0.9454	0.9990	NA	1,0000	0.8344	0.8940	NA

Table S 36: Sensitivity obtained by all the algorithms under the haplotype-based approach with 3-SNP haplotype length, additive genetic model and holdout sampling. Results for each p-value threshold (column 1) are shown. The maximum p-value threshold used was 0.15.

p-value threshold	BD	CAD	HT	IBD	RA	T1D	T2D
				NBC			
0.15	0.9486	0.0000	0.0000	0.1499	0.9398	0.9205	0.8929
0.1	0.9443	0.0000	0.0000	0.2941	0.9387	0.9174	0.8888
0.05	0.9293	0.0000	0.0000	0.3066	0.9247	0.8899	0.8763
0.01	0.9026	0.0000	0.0000	0.3158	0.8806	0.8012	0.8493
0.0010	0.9111	0.0031	0.3361	0.8295	0.8409	0.6993	0.8711
1.0e-4	0.9400	0.5846	0.0061	0.9279	0.8215	0.7074	0.9491
1.0e-5	0.9775	1,0000	1,0000	0.9783	0.8086	0.7176	0.9740
1.0e-6	0.9882	1,0000	1,0000	0.9989	0.8043	0.7380	1,0000
1.0e-7	0.9989	1,0000	NA	1,0000	0.7978	0.7686	NA
				sSVM			
0.15	0.9797	1,0000	1,0000	0.7517	0.9796	0.9847	0.9387
0.1	0.9657	1,0000	1,0000	0.9142	0.9699	0.9694	0.9220
0.05	0.9390	1,0000	1,0000	0.8330	0.9570	0.9460	0.9023
0.01	0.9101	0.6771	0.7193	0.8135	0.9226	0.9348	0.8805
0.0010	0.8683	0.4964	0.6240	0.7586	0.9108	0.9164	0.8420
1.0e-4	0.9208	1,0000	0.9098	0.8753	0.8860	0.9348	0.8898
1.0e-5	0.9893	0.3209	0.0379	0.9645	0.8559	0.9235	0.9647
1.0e-6	0.9882	0.5815	1,0000	0.9840	0.8742	0.9205	0.9802
1.0e-7	0.9989	1,0000	NA	1,0000	0.8720	0.9113	NA
				AdaBoostM1			
0.15	0.8865	0.2658	0.9447	0.4954	0.9022	0.8919	0.8285
0.1	0.8972	0.7819	0.5779	0.7654	0.8989	0.9042	0.8482
0.05	0.8951	0.3022	0.4344	0.6224	0.9054	0.9113	0.8482
0.01	0.8983	0.3956	0.0943	0.8478	0.8925	0.9062	0.8295
0.0010	0.8919	0.8463	0.8760	0.4817	0.9172	0.9246	0.8711
1.0e-4	0.9636	0.0021	0.0031	0.9703	0.9484	0.9592	0.9813
1.0e-5	0.9893	0.9958	1,0000	0.9931	0.9763	0.9694	0.9979
1.0e-6	0.9936	1,0000	1,0000	0.9989	0.9935	0.9715	1,0000
1.0e-7	0.9989	1,0000	NA	1,0000	0.9925	0.9755	NA
				C4.5			
0.15	0.8533	0.0000	0.0000	0.0721	0.8430	0.8145	0.8264
0.1	0.8565	0.0000	0.0000	0.0721	0.8204	0.8675	0.7807
0.05	0.8801	0.0000	0.0000	0.2620	0.8624	0.8532	0.8191
0.01	0.8555	0.0000	0.0000	0.5995	0.8602	0.8624	0.8087
0.0010	0.8287	0.4631	0.7961	0.7082	0.8989	0.9062	0.8295
1.0e-4	0.8972	0.0000	0.7633	0.8410	0.8559	0.8981	0.8732
1.0e-5	0.9839	0.4559	0.0758	0.9908	0.8946	0.9052	0.9958
1.0e-6	0.9904	1,0000	1,0000	0.9989	0.9097	0.9195	1,0000
1.0e-7	0.9989	1,0000	NA	1,0000	0.9075	0.9185	NA
				20RF			
0.15	1,0000	1,0000	1,0000	1,0000	1,0000	1,0000	1,0000
0.1	1,0000	1,0000	0.9990	1,0000	1,0000	1,0000	1,0000
0.05	1,0000	1,0000	1,0000	1,0000	1,0000	1,0000	1,0000
0.01	1,0000	1,0000	1,0000	1,0000	1,0000	1,0000	1,0000
0.0010	1,0000	1,0000	1,0000	1,0000	1,0000	1,0000	1,0000
1.0e-4	0.9989	1,0000	1,0000	1,0000	1,0000	1,0000	1,0000
1.0e-5	0.9979	1,0000	0.9990	0.9977	1,0000	1,0000	0.9990
1.0e-6	0.9957	0.7404	1,0000	0.9989	1,0000	1,0000	1,0000
1.0e-7	0.9989	1,0000	NA	1,0000	1,0000	0.9990	NA

Table S 37: Sensitivity obtained by all the algorithms under the haplotype-based approach with 3-SNP haplotype length, dominant genetic model and holdout sampling. Results for each p-value threshold (column 1) are shown. The maximum p-value threshold used was 0.15.

p-value threshold	BD	CAD	HT	IBD	RA	T1D	T2D
**NBC**							
0.15	0.6274	0.0000	0.0000	0.0080	0.6505	0.5719	0.4906
0.1	0.6242	0.0000	0.0000	0.0240	0.6344	0.5392	0.4813
0.05	0.5846	0.0000	0.0000	0.0275	0.6000	0.5025	0.4636
0.01	0.5343	0.0000	0.0000	0.0320	0.5570	0.4393	0.4096
0.0010	0.4893	0.0000	0.0102	0.3169	0.5538	0.3894	0.3389
1.0e-4	0.4272	0.0021	0.0000	0.2506	0.5462	0.4383	0.2464
1.0e-5	0.3201	0.1983	0.0000	0.0503	0.5344	0.4383	0.1778
1.0e-6	0.0921	0.1859	0.0000	0.0549	0.5269	0.4495	0.0166
1.0e-7	0.0931	0.0000	NA	0.0721	0.5151	0.4709	NA
**sSVM**							
0.15	0.7655	1,0000	1,0000	0.2677	0.7731	0.7472	0.5936
0.1	0.6724	1,0000	1,0000	0.5206	0.7075	0.6504	0.5208
0.05	0.6060	1,0000	1,0000	0.3593	0.6086	0.6045	0.5073
0.01	0.5343	0.1921	0.2244	0.3089	0.5398	0.5800	0.4148
0.0010	0.4261	0.0706	0.1527	0.2677	0.5194	0.5800	0.3690
1.0e-4	0.4893	0.9605	0.5031	0.4085	0.5505	0.6789	0.4459
1.0e-5	0.8737	0.0363	0.0000	0.6419	0.5828	0.6850	0.6445
1.0e-6	0.8972	0.1163	0.9887	0.7529	0.6022	0.6809	0.7516
1.0e-7	0.9433	1,0000	NA	1,0000	0.5860	0.6646	NA
**AdaBoostM1**							
0.15	0.4454	0.0125	0.5758	0.0881	0.5075	0.5127	0.3659
0.1	0.4797	0.2565	0.1158	0.2540	0.4989	0.5382	0.3753
0.05	0.4304	0.0280	0.0594	0.1293	0.5247	0.5403	0.3898
0.01	0.4572	0.0550	0.0041	0.3673	0.5151	0.5199	0.3732
0.0010	0.4133	0.2368	0.3094	0.0641	0.4968	0.4903	0.2963
1.0e-4	0.3030	0.0000	0.0000	0.1568	0.4462	0.5199	0.1715
1.0e-5	0.1660	0.0291	0.0000	0.0400	0.3839	0.5005	0.0852
1.0e-6	0.0921	0.0571	0.0000	0.0561	0.3419	0.5127	0.0301
1.0e-7	0.0931	0.0000	NA	0.0721	0.2925	0.4709	NA
**C4.5**							
0.15	0.4026	0.0000	0.0000	0.0000	0.4129	0.3456	0.3732
0.1	0.4304	0.0000	0.0000	0.0000	0.4398	0.4485	0.3316
0.05	0.4797	0.0000	0.0000	0.0217	0.4301	0.4220	0.3597
0.01	0.4368	0.0000	0.0000	0.1247	0.4419	0.4037	0.3742
0.0010	0.4026	0.0633	0.3043	0.2002	0.4914	0.4954	0.3368
1.0e-4	0.4133	0.0000	0.2592	0.3341	0.4882	0.4873	0.3285
1.0e-5	0.1959	0.0727	0.0000	0.0400	0.5419	0.5882	0.0832
1.0e-6	0.0000	0.0000	0.0000	0.0561	0.5452	0.5872	0.0260
1.0e-7	0.0000	0.0000	NA	0.0000	0.5258	0.5933	NA
**20RF**							
0.15	0.0000	0.0073	0.0020	0.0000	0.0000	0.0000	0.0000
0.1	0.0000	0.0000	0.0000	0.0000	0.0000	0.0000	0.0000
0.05	0.0000	0.0042	0.0092	0.0000	0.0000	0.0000	0.0000
0.01	0.0000	0.0062	0.0266	0.0000	0.0000	0.0000	0.0000
0.0010	0.0000	0.0000	0.0000	0.0000	0.0011	0.0071	0.0000
1.0e-4	0.0000	0.0000	0.0000	0.0011	0.0634	0.0642	0.0125
1.0e-5	0.0075	0.0000	0.0000	0.0389	0.1753	0.0948	0.0374
1.0e-6	0.0910	0.0093	0.0000	0.0561	0.1935	0.1315	0.0395
1.0e-7	0.0931	0.0000	NA	0.0721	0.2301	0.1427	NA

Table S 38: Sensitivity obtained by all the algorithms under the haplotype-based approach with 3-SNP haplotype length, recessive genetic model and holdout sampling. Results for each p-value threshold (column 1) are shown. The maximum p-value threshold used was 0.15.

p-value threshold	BD	CAD	HT	IBD	RA	T1D	T2D
				NBC			
0.15	0.8522	0.0000	0.0000	0.3673	0.8613	0.8236	0.7443
0.1	0.8405	0.0000	0.0000	0.3215	0.8376	0.8094	0.7360
0.05	0.8158	0.0000	0.0000	0.5812	0.8011	0.7778	0.7256
0.01	0.7666	0.0000	0.0020	0.0950	0.7312	0.6687	0.6736
0.0010	0.7034	0.0312	0.0031	0.2986	0.6957	0.6249	0.6538
1.0e-4	0.7527	0.9990	0.1445	0.3455	0.6710	0.6208	0.6549
1.0e-5	0.8769	1,0000	1,0000	0.5732	0.6624	0.6351	0.7214
1.0e-6	0.9347	1,0000	NA	0.7792	0.6527	0.6473	0.6975
1.0e-7	0.9743	1,0000	NA	1,0000	0.6645	0.6555	NA
				sSVM			
0.15	0.9694	1,0000	1,0000	0.8893	0.9823	0.9642	0.9298
0.1	0.9366	1,0000	1,0000	0.8962	0.9620	0.9527	0.8900
0.05	0.9161	1,0000	1,0000	0.9476	0.9260	0.9414	0.8422
0.01	0.8566	0.1023	0.9917	0.7515	0.8706	0.8995	0.7831
0.0010	0.7523	0.7160	0.7726	0.5772	0.8165	0.8812	0.7137
1.0e-4	0.8741	0.9977	0.2532	0.6049	0.8295	0.9045	0.7680
1.0e-5	0.9833	0.9706	0.9989	0.7474	0.8006	0.8890	0.7902
1.0e-6	0.9966	0.9691	NA	0.9824	0.7942	0.8868	0.7438
1.0e-7	1,0000	1,0000	NA	1,0000	0.7946	0.8822	NA
				AdaBoostM1			
0.15	0.7206	0.9616	0.3105	0.2895	0.7656	0.8002	0.6632
0.1	0.7163	0.0021	0.7377	0.6465	0.7710	0.7880	0.6570
0.05	0.7291	0.1215	0.8033	0.3776	0.7785	0.7900	0.6570
0.01	0.7259	0.0104	0.9057	0.3227	0.7516	0.7819	0.6736
0.0010	0.7313	0.7290	0.8545	0.0320	0.7387	0.7492	0.6362
1.0e-4	0.7602	1,0000	0.2746	0.3707	0.7720	0.8359	0.6694
1.0e-5	0.8694	1,0000	0.9980	0.7941	0.7505	0.8451	0.7921
1.0e-6	0.9347	1,0000	NA	0.7792	0.7849	0.8430	0.6975
1.0e-7	0.9743	1,0000	NA	1,0000	0.7817	0.8420	NA
				C4.5			
0.15	0.6778	0.0000	0.0000	0.0000	0.6935	0.6158	0.6199
0.1	0.7271	0.0000	0.0000	0.0000	0.7386	0.6476	0.5957
0.05	0.7281	0.0000	0.0000	0.0126	0.7288	0.6966	0.6542
0.01	0.6786	0.0000	0.0000	0.0738	0.7276	0.7339	0.6636
0.0010	0.6757	0.8099	0.8199	0.0467	0.6833	0.7076	0.6866
1.0e-4	0.6681	0.9964	0.1916	0.3937	0.6532	0.6710	0.6057
1.0e-5	0.8758	0.9678	0.9949	0.6888	0.6735	0.6842	0.7453
1.0e-6	0.9304	0.9065	NA	0.7792	0.7099	0.7146	0.8607
1.0e-7	0.9743	1,0000	NA	1,0000	0.7348	0.7137	NA
				20RF			
0.15	0.9936	0.8972	0.8402	0.9142	0.9882	0.9908	0.9595
0.1	0.9861	0.7736	0.6598	0.8146	0.9903	0.9969	0.9574
0.05	0.9839	0.8650	0.8842	0.9645	0.9892	0.9817	0.9532
0.01	0.9764	0.9709	0.9395	0.9565	0.9871	0.9878	0.9574
0.0010	0.9839	0.1495	0.5605	0.9119	0.9312	0.9511	0.9480
1.0e-4	0.9572	0.9772	0.6270	0.4542	0.8699	0.9062	0.8368
1.0e-5	0.9347	0.9990	1,0000	0.7961	0.8387	0.8858	0.8482
1.0e-6	0.9347	0.8494	NA	0.7792	0.8290	0.8807	0.7412
1.0e-7	0.9743	0.7290	NA	1,0000	0.8280	0.8695	NA

Table S 39: Sensitivity obtained by all the algorithms under the haplotype-based approach with 4-SNP haplotype length, additive genetic model and holdout sampling. Results for each p-value threshold (column 1) are shown. The maximum p-value threshold used was 0.15.

p-value threshold	BD	CAD	HT	IBD	RA	T1D	T2D
NBC							
0.15	0.9422	0.0000	0.0000	0.6270	0.9430	0.9185	0.8929
0.1	0.9347	0.0000	0.0000	0.6098	0.9312	0.9103	0.8857
0.05	0.9325	0.0000	0.0000	0.7986	0.9215	0.8940	0.8836
0.01	0.9165	0.0000	0.0205	0.3421	0.8785	0.7931	0.8565
0.0010	0.8737	0.2295	0.1260	0.6133	0.8161	0.6972	0.8545
1.0e-4	0.9358	1,0000	0.8504	0.7952	0.8022	0.7136	0.9491
1.0e-5	0.9904	1,0000	1,0000	0.9840	0.7839	0.7227	0.9948
1.0e-6	0.9968	1,0000	NA	1,0000	0.7946	0.7299	1,0000
1.0e-7	1,0000	1,0000	NA	1,0000	0.8108	0.7370	NA
sSVM							
0.15	0.9764	1,0000	1,0000	0.9314	0.9860	0.9725	0.9543
0.1	0.9561	1,0000	1,0000	0.9348	0.9731	0.9664	0.9324
0.05	0.9443	1,0000	1,0000	0.9657	0.9505	0.9613	0.9075
0.01	0.9122	0.4444	0.9928	0.8616	0.9204	0.9358	0.8773
0.0010	0.8587	0.8525	0.8740	0.7838	0.8849	0.9215	0.8503
1.0e-4	0.9261	0.9979	0.5861	0.8146	0.8871	0.9286	0.8690
1.0e-5	0.9861	0.9782	0.9990	0.8604	0.8613	0.9134	0.8846
1.0e-6	0.9968	0.9772	NA	0.9863	0.8559	0.9144	0.8607
1.0e-7	1,0000	1,0000	NA	1,0000	0.8538	0.9103	NA
AdaBoostM1							
0.15	0.8833	0.9896	0.6055	0.5721	0.9129	0.9103	0.8659
0.1	0.8887	0.0519	0.8832	0.8547	0.9065	0.9052	0.8482
0.05	0.8844	0.3728	0.9232	0.6602	0.9032	0.9256	0.8669
0.01	0.8801	0.0789	0.9703	0.6327	0.8935	0.9072	0.8701
0.0010	0.8940	0.9128	0.9652	0.1728	0.8978	0.9052	0.8732
1.0e-4	0.9647	1,0000	0.6947	0.8421	0.9484	0.9582	0.9802
1.0e-5	0.9946	1,0000	1,0000	0.9989	0.9645	0.9643	0.9990
1.0e-6	0.9968	1,0000	NA	1,0000	0.9871	0.9694	1,0000
1.0e-7	1,0000	1,0000	NA	1,0000	0.9903	0.9725	NA
C4.5							
0.15	0.8330	0.0000	0.0000	0.0732	0.8344	0.8624	0.8254
0.1	0.8458	0.0000	0.0000	0.0835	0.8699	0.8430	0.7869
0.05	0.8437	0.0000	0.0000	0.1945	0.8634	0.8644	0.8389
0.01	0.8608	0.0000	0.0020	0.3970	0.8667	0.8756	0.8274
0.0010	0.8704	0.8889	0.9037	0.2609	0.8548	0.8787	0.8129
1.0e-4	0.8747	0.9969	0.5328	0.6453	0.8570	0.9011	0.8805
1.0e-5	0.9957	1,0000	1,0000	1,0000	0.8935	0.9286	0.9958
1.0e-6	0.9968	0.9979	NA	1,0000	0.9032	0.9103	1,0000
1.0e-7	1,0000	1,0000	NA	1,0000	0.9161	0.9174	NA
20RF							
0.15	1,0000	1,0000	1,0000	1,0000	1,0000	1,0000	1,0000
0.1	1,0000	1,0000	1,0000	1,0000	1,0000	1,0000	1,0000
0.05	1,0000	1,0000	1,0000	1,0000	1,0000	1,0000	1,0000
0.01	1,0000	1,0000	1,0000	1,0000	1,0000	1,0000	1,0000
0.0010	1,0000	1,0000	1,0000	1,0000	1,0000	1,0000	1,0000
1.0e-4	1,0000	1,0000	1,0000	0.9966	1,0000	1,0000	1,0000
1.0e-5	0.9989	1,0000	1,0000	0.9954	1,0000	1,0000	1,0000
1.0e-6	0.9968	0.9958	NA	1,0000	1,0000	1,0000	1,0000
1.0e-7	1,0000	0.9782	NA	1,0000	1,0000	0.9990	NA

Table S 40: Sensitivity obtained by all the algorithms under the haplotype-based approach with 4-SNP haplotype length, dominant genetic model and holdout sampling. Results for each p-value threshold (column 1) are shown. The maximum p-value threshold used was 0.15.

p-value threshold	BD	CAD	HT	IBD	RA	T1D	T2D
				NBC			
0.15	0.6263	0.0000	0.0000	0.1499	0.6484	0.5780	0.4927
0.1	0.6017	0.0000	0.0000	0.1270	0.6183	0.5525	0.4927
0.05	0.5867	0.0000	0.0000	0.2929	0.5796	0.5301	0.4647
0.01	0.5193	0.0000	0.0000	0.0275	0.5430	0.4567	0.3971
0.0010	0.4251	0.0021	0.0000	0.1087	0.5462	0.4169	0.3451
1.0e-4	0.3726	0.9730	0.0000	0.0755	0.5366	0.4312	0.1913
1.0e-5	0.2623	0.8297	0.9795	0.0446	0.5226	0.4414	0.1507
1.0e-6	0.0931	0.9387	NA	0.0584	0.5172	0.4506	0.0437
1.0e-7	0.0953	0.9408	NA	0.0732	0.5054	0.4536	NA
				sSVM			
0.15	0.7473	1,0000	1,0000	0.5515	0.7763	0.7411	0.6060
0.1	0.6488	1,0000	1,0000	0.5629	0.6806	0.6769	0.5468
0.05	0.6081	1,0000	1,0000	0.6201	0.6194	0.6228	0.4938
0.01	0.5246	0.0633	0.8545	0.4188	0.5355	0.5749	0.4428
0.0010	0.4293	0.3718	0.4283	0.2952	0.5118	0.5821	0.3732
1.0e-4	0.5128	0.8899	0.1404	0.2838	0.5495	0.6758	0.4335
1.0e-5	0.8201	0.7196	0.9457	0.4130	0.5570	0.6942	0.4345
1.0e-6	0.9304	0.7175	NA	0.7643	0.5559	0.6707	0.4044
1.0e-7	0.9743	1,0000	NA	1,0000	0.5656	0.6718	NA
				AdaBoostM1			
0.15	0.4154	0.7944	0.1363	0.1133	0.5129	0.5525	0.3753
0.1	0.4197	0.0000	0.4324	0.3638	0.5280	0.5291	0.3784
0.05	0.4443	0.0415	0.5184	0.1648	0.5129	0.5260	0.3794
0.01	0.4411	0.0000	0.6814	0.1281	0.4828	0.5158	0.4168
0.0010	0.4261	0.4694	0.5523	0.0046	0.4925	0.4608	0.3233
1.0e-4	0.3009	0.9958	0.0676	0.0595	0.4581	0.5199	0.1705
1.0e-5	0.1831	0.9772	0.9826	0.0446	0.4108	0.5240	0.0977
1.0e-6	0.0942	0.9730	NA	0.0584	0.3269	0.5270	0.0437
1.0e-7	0.0953	0.9398	NA	0.0732	0.3075	0.4954	NA
				C4.5			
0.15	0.3983	0.0000	0.0000	0.0000	0.3806	0.3996	0.3482
0.1	0.4261	0.0000	0.0000	0.0000	0.4323	0.3925	0.3306
0.05	0.4133	0.0000	0.0000	0.0103	0.3903	0.4475	0.3815
0.01	0.4315	0.0000	0.0000	0.0481	0.4882	0.4750	0.3545
0.0010	0.4026	0.4735	0.4477	0.0229	0.4269	0.4618	0.3524
1.0e-4	0.4604	0.8619	0.0891	0.1281	0.4806	0.5076	0.3742
1.0e-5	0.1949	0.7092	0.0000	0.0446	0.5140	0.5647	0.0904
1.0e-6	0.0000	0.0125	NA	0.0584	0.4935	0.5678	0.0457
1.0e-7	0.0000	0.0000	NA	0.0000	0.5495	0.5545	NA
				20RF			
0.15	0.0000	0.0021	0.0020	0.0000	0.0000	0.0000	0.0000
0.1	0.0000	0.0021	0.0000	0.0000	0.0000	0.0000	0.0000
0.05	0.0000	0.0073	0.0082	0.0000	0.0000	0.0000	0.0000
0.01	0.0000	0.0042	0.0102	0.0000	0.0000	0.0000	0.0000
0.0010	0.0000	0.0000	0.0000	0.0000	0.0000	0.0194	0.0000
1.0e-4	0.0021	0.0093	0.0000	0.0011	0.0645	0.0673	0.0073
1.0e-5	0.0075	0.0291	0.9355	0.0458	0.1452	0.0917	0.0374
1.0e-6	0.0921	0.3458	NA	0.0584	0.1903	0.1488	0.0374
1.0e-7	0.0953	0.0010	NA	0.0732	0.2151	0.1855	NA

Table S 41: Sensitivity obtained by all the algorithms under the haplotype-based approach with 4-SNP haplotype length, recessive genetic model and holdout sampling. Results for each p-value threshold (column 1) are shown. The maximum p-value threshold used was 0.15.

p-value threshold	BD	CAD	HT	IBD	RA	T1D	T2D
			NBC				
0.15	0.8469	0.0000	0.0000	0.6144	0.8473	0.8175	0.7495
0.1	0.8448	0.0000	0.0000	0.8261	0.8344	0.8002	0.7380
0.05	0.7976	0.0000	0.0000	0.3902	0.7989	0.7594	0.7027
0.01	0.7441	0.0062	0.0000	0.1510	0.7183	0.6799	0.6788
0.0010	0.7045	0.0000	0.0000	0.5469	0.6677	0.6300	0.6549
1.0e-4	0.7591	0.0021	0.0666	0.7288	0.6602	0.6259	0.6507
1.0e-5	0.8994	0.9813	1,0000	0.5744	0.6538	0.6259	0.7141
1.0e-6	0.9325	0.9958	NA	0.7071	0.6441	0.6432	1,0000
1.0e-7	0.9582	1,0000	NA	1,0000	0.6656	0.6483	NA
			sSVM				
0.15	0.9648	1,0000	1,0000	0.9746	0.9747	0.9609	0.9035
0.1	0.9311	1,0000	1,0000	0.9946	0.9561	0.9550	0.8590
0.05	0.9015	1,0000	1,0000	0.9475	0.9237	0.9197	0.8304
0.01	0.8429	0.6611	0.7030	0.8507	0.8452	0.8961	0.7454
0.0010	0.8000	0.0296	0.9898	0.6875	0.8504	0.8447	0.7275
1.0e-4	0.8562	0.2848	1,0000	0.8558	0.8310	0.9053	0.7584
1.0e-5	0.9916	1,0000	1,0000	0.6798	0.8241	0.8936	0.8932
1.0e-6	0.9931	1,0000	NA	0.9478	0.8220	0.8911	1,0000
1.0e-7	0.9967	1,0000	NA	1,0000	0.8406	0.8762	NA
			AdaBoostM1				
0.15	0.7270	0.3624	0.1967	0.2735	0.7570	0.7971	0.6424
0.1	0.6991	0.6677	0.7111	0.6922	0.7452	0.7910	0.6726
0.05	0.7131	0.2409	0.4068	0.4314	0.7710	0.8002	0.6486
0.01	0.6916	0.0509	0.1240	0.4542	0.7140	0.7880	0.6310
0.0010	0.6959	0.1651	0.0133	0.3993	0.7409	0.7105	0.6778
1.0e-4	0.7570	0.7321	0.0205	0.7780	0.7634	0.8165	0.6746
1.0e-5	0.9090	0.7352	1,0000	0.7529	0.7731	0.8298	0.7131
1.0e-6	0.9315	0.8442	NA	0.7071	0.7860	0.8379	1,0000
1.0e-7	0.9582	1,0000	NA	1,0000	0.7989	0.8277	NA
			C4.5				
0.15	0.6684	0.0000	0.0000	0.0013	0.6558	0.5921	0.6767
0.1	0.6812	0.0000	0.0000	0.5671	0.6889	0.5802	0.6318
0.05	0.7152	0.0000	0.0000	0.2251	0.7610	0.5900	0.6392
0.01	0.6899	0.0000	0.0000	0.1597	0.6456	0.6883	0.5964
0.0010	0.5619	0.0000	0.0000	0.3648	0.7304	0.7185	0.5927
1.0e-4	0.6667	0.0000	0.6757	0.6553	0.6892	0.6942	0.6177
1.0e-5	0.8833	0.9990	1,0000	0.7963	0.6670	0.6849	0.6632
1.0e-6	0.9304	1,0000	NA	0.7071	0.6491	0.6840	1,0000
1.0e-7	0.9582	1,0000	NA	1,0000	0.6898	0.6964	NA
			20RF				
0.15	0.9818	0.9138	0.8105	0.8867	0.9946	0.9939	0.9605
0.1	0.9893	0.9325	0.9426	0.9222	0.9753	0.9898	0.9709
0.05	0.9904	0.8141	0.9355	0.6465	0.9849	0.9878	0.9595
0.01	0.9775	0.5036	0.7520	0.7540	0.9817	0.9786	0.9563
0.0010	0.9786	0.1246	0.4006	0.7025	0.9108	0.9368	0.9220
1.0e-4	0.9497	0.9948	0.7264	0.7803	0.8484	0.9011	0.8555
1.0e-5	0.9336	0.9979	1,0000	0.7540	0.8323	0.8807	0.8004
1.0e-6	0.9325	0.9927	NA	0.7071	0.8172	0.8777	1,0000
1.0e-7	0.9582	0.9782	NA	1,0000	0.8172	0.8593	NA

Table S 42: Sensitivity obtained by all the algorithms under the haplotype-based approach with 5-SNP haplotype length, additive genetic model and holdout sampling. Results for each p-value threshold (column 1) are shown. The maximum p-value threshold used was 0.15.

p-value threshold	BD	CAD	HT	IBD	RA	T1D	T2D
				NBC			
0.15	0.9400	0.0000	0.0000	0.8227	0.9452	0.9174	0.8867
0.1	0.9336	0.0000	0.0000	0.9325	0.9269	0.9052	0.8784
0.05	0.9143	0.0042	0.0000	0.6739	0.9151	0.8777	0.8659
0.01	0.8833	0.0322	0.0000	0.4279	0.8473	0.8094	0.8514
0.0010	0.8801	0.0021	0.0123	0.8169	0.8065	0.7156	0.8742
1.0e-4	0.9465	0.0384	0.9631	0.9577	0.7839	0.7115	0.9418
1.0e-5	0.9904	1,0000	1,0000	0.9680	0.7710	0.7125	0.9979
1.0e-6	0.9936	1,0000	NA	1,0000	0.7839	0.7238	1,0000
1.0e-7	0.9968	1,0000	NA	1,0000	0.7935	0.7401	NA
				sSVM			
0.15	0.9732	1,0000	1,0000	0.9805	0.9806	0.9704	0.9366
0.1	0.9518	1,0000	1,0000	0.9954	0.9688	0.9684	0.9116
0.05	0.9358	1,0000	1,0000	0.9634	0.9495	0.9480	0.9012
0.01	0.9058	0.8318	0.8494	0.9165	0.9043	0.9327	0.8576
0.0010	0.8844	0.2513	0.9918	0.8455	0.9043	0.8981	0.8555
1.0e-4	0.9111	0.6324	1,0000	0.9153	0.8839	0.9307	0.8732
1.0e-5	0.9925	1,0000	1,0000	0.8238	0.8731	0.9185	0.9345
1.0e-6	0.9936	1,0000	NA	0.9622	0.8731	0.9154	1,0000
1.0e-7	0.9968	1,0000	NA	1,0000	0.8828	0.9042	NA
				AdaBoostM1			
0.15	0.8854	0.6698	0.4918	0.5801	0.8989	0.9062	0.8326
0.1	0.8822	0.8660	0.8801	0.8799	0.9043	0.9103	0.8628
0.05	0.8822	0.5234	0.6783	0.7059	0.9097	0.9134	0.8534
0.01	0.8726	0.2212	0.3760	0.7483	0.8903	0.9164	0.8399
0.0010	0.8887	0.4683	0.1035	0.7048	0.9022	0.8889	0.8940
1.0e-4	0.9657	0.9772	0.0471	0.9794	0.9495	0.9521	0.9782
1.0e-5	0.9925	0.9782	1,0000	1,0000	0.9634	0.9674	0.9979
1.0e-6	0.9936	0.9782	NA	1,0000	0.9796	0.9725	1,0000
1.0e-7	0.9968	1,0000	NA	1,0000	0.9828	0.9684	NA
				C4.5			
0.15	0.8587	0.0000	0.0000	0.0870	0.8140	0.8879	0.8378
0.1	0.8555	0.0000	0.0000	0.7243	0.8581	0.8838	0.8233
0.05	0.8565	0.0000	0.0000	0.5549	0.8720	0.8624	0.8430
0.01	0.8480	0.0000	0.0031	0.4428	0.8484	0.8695	0.8004
0.0010	0.8298	0.0062	0.0031	0.5915	0.8570	0.8879	0.8347
1.0e-4	0.8544	0.0021	0.9109	0.8318	0.8538	0.8991	0.8503
1.0e-5	0.9904	1,0000	1,0000	0.9989	0.8624	0.8919	0.9896
1.0e-6	0.9936	1,0000	NA	1,0000	0.8763	0.9093	1,0000
1.0e-7	0.9968	1,0000	NA	1,0000	0.8742	0.8899	NA
				20RF			
0.15	1,0000	1,0000	1,0000	1,0000	1,0000	1,0000	1,0000
0.1	1,0000	1,0000	1,0000	1,0000	1,0000	1,0000	1,0000
0.05	1,0000	1,0000	1,0000	1,0000	1,0000	1,0000	1,0000
0.01	1,0000	0.9990	1,0000	1,0000	1,0000	1,0000	1,0000
0.0010	1,0000	0.9979	1,0000	1,0000	1,0000	1,0000	1,0000
1.0e-4	1,0000	1,0000	1,0000	1,0000	1,0000	1,0000	1,0000
1.0e-5	0.9979	1,0000	1,0000	0.9966	1,0000	1,0000	1,0000
1.0e-6	0.9936	1,0000	NA	1,0000	1,0000	0.9990	1,0000
1.0e-7	0.9968	1,0000	NA	1,0000	1,0000	0.9990	NA

Table S 43: Sensitivity obtained by all the algorithms under the haplotype-based approach with 5-SNP haplotype length, dominant genetic model and holdout sampling. Results for each p-value threshold (column 1) are shown. The maximum p-value threshold used was 0.15.

p-value threshold	BD	CAD	HT	IBD	RA	T1D	T2D
				NBC			
0.15	0.6028	0.0000	0.0000	0.3284	0.6151	0.5810	0.4906
0.1	0.5931	0.0000	0.0000	0.5744	0.6011	0.5627	0.4626
0.05	0.5503	0.0000	0.0000	0.1888	0.5613	0.5189	0.4543
0.01	0.4818	0.0010	0.0000	0.0526	0.5376	0.4536	0.3877
0.0010	0.4186	0.0000	0.0000	0.2449	0.5301	0.4098	0.3368
1.0e-4	0.3276	0.0000	0.0000	0.3101	0.5237	0.4251	0.1975
1.0e-5	0.1670	0.3551	0.0000	0.0572	0.5161	0.4281	0.0956
1.0e-6	0.0931	0.3406	NA	0.0503	0.5086	0.4424	0.0000
1.0e-7	0.0953	0.0000	NA	0.0721	0.5097	0.4485	NA
				sSVM			
0.15	0.7345	1,0000	1,0000	0.7460	0.7462	0.7258	0.5936
0.1	0.6510	1,0000	1,0000	0.8455	0.6796	0.6707	0.5385
0.05	0.5878	1,0000	1,0000	0.6602	0.6118	0.5953	0.4834
0.01	0.5054	0.3281	0.3566	0.4760	0.5226	0.5800	0.4168
0.0010	0.4625	0.0228	0.7920	0.3398	0.5441	0.5545	0.3857
1.0e-4	0.5289	0.1464	0.9713	0.5023	0.5710	0.6626	0.3981
1.0e-5	0.8822	1,0000	1,0000	0.3741	0.5946	0.6850	0.5478
1.0e-6	0.9304	1,0000	NA	0.6854	0.5860	0.6922	1,0000
1.0e-7	0.9582	1,0000	NA	1,0000	0.6183	0.6779	NA
				AdaBoostM1			
0.15	0.4272	0.1568	0.0707	0.0961	0.5097	0.5525	0.3815
0.1	0.4218	0.3790	0.4180	0.4039	0.4914	0.5331	0.3669
0.05	0.4411	0.1038	0.1834	0.1796	0.4860	0.5433	0.3815
0.01	0.4058	0.0187	0.0400	0.1991	0.4473	0.5015	0.3555
0.0010	0.3844	0.0633	0.0010	0.1831	0.4731	0.4383	0.3534
1.0e-4	0.2655	0.4933	0.0010	0.2803	0.4301	0.5189	0.1663
1.0e-5	0.1520	0.7207	0.9980	0.0389	0.3763	0.5270	0.0800
1.0e-6	0.0942	0.7217	NA	0.0503	0.3172	0.5076	0.0748
1.0e-7	0.0953	0.0000	NA	0.0721	0.3172	0.4873	NA
				C4.5			
0.15	0.3919	0.0000	0.0000	0.0011	0.3785	0.4332	0.3857
0.1	0.4036	0.0000	0.0000	0.2449	0.4011	0.4404	0.3805
0.05	0.3897	0.0000	0.0000	0.1053	0.4398	0.3976	0.3649
0.01	0.3801	0.0000	0.0000	0.0664	0.4269	0.4006	0.3368
0.0010	0.3694	0.0000	0.0000	0.1259	0.4742	0.4485	0.3659
1.0e-4	0.4208	0.0000	0.3965	0.3192	0.4720	0.4985	0.3316
1.0e-5	0.0021	0.8795	0.0000	0.0389	0.4591	0.4862	0.0956
1.0e-6	0.0000	0.0000	NA	0.0503	0.4237	0.4913	0.0000
1.0e-7	0.0000	0.0000	NA	0.0000	0.4688	0.5097	NA
				20RF			
0.15	0.0000	0.0010	0.0000	0.0000	0.0000	0.0000	0.0000
0.1	0.0000	0.0104	0.0051	0.0000	0.0000	0.0000	0.0000
0.05	0.0000	0.0021	0.0051	0.0000	0.0000	0.0000	0.0000
0.01	0.0000	0.0000	0.0000	0.0000	0.0000	0.0000	0.0000
0.0010	0.0000	0.0000	0.0000	0.0000	0.0022	0.0133	0.0000
1.0e-4	0.0000	0.1194	0.0000	0.0011	0.0882	0.0673	0.0010
1.0e-5	0.0310	0.0000	0.8217	0.0389	0.1495	0.1193	0.0374
1.0e-6	0.0931	0.1880	NA	0.0503	0.1957	0.1264	0.0000
1.0e-7	0.0953	0.0083	NA	0.0721	0.2118	0.1519	NA

Table S 44: Sensitivity obtained by all the algorithms under the haplotype-based approach with 5-SNP haplotype length, recessive genetic model and holdout sampling. Results for each p-value threshold (column 1) are shown. The maximum p-value threshold used was 0.15.

p-value threshold	BD	CAD	HT	IBD	RA	T1D	T2D
			NBC				
0.15	0.2297	1,0000	1,0000	0.6892	0.2419	0.3892	0.324
0.1	0.2486	1,0000	1,0000	0.9135	0.2811	0.4324	0.350
0.05	0.2703	1,0000	1,0000	0.9365	0.3365	0.5122	0.366
0.01	0.3608	1,0000	1,0000	0.9959	0.4716	0.6378	0.391
0.0010	0.3959	1,0000	1,0000	0.4581	0.5541	0.7135	0.427
1.0e-4	0.4000	1,0000	1,0000	0.4230	0.6068	0.7095	0.450
1.0e-5	0.2689	0.4108	1,0000	0.5054	0.6257	0.7095	0.409
1.0e-6	NA	0.0649	NA	NA	0.5811	0.7027	0.168
1.0e-7	NA	0.2595	NA	NA	0.5676	0.6797	NA
			sSVM				
0.15	0.0269	0.0000	0.0000	0.2141	0.0149	0.0627	0.102
0.1	0.0866	0.0000	0.0000	0.7071	0.0702	0.1797	0.182
0.05	0.1489	0.0000	0.0000	0.7150	0.1339	0.2550	0.209
0.01	0.2173	0.0081	0.0076	0.9768	0.2481	0.4268	0.237
0.0010	0.3034	0.5963	0.8460	0.2792	0.3753	0.6630	0.345
1.0e-4	0.1567	0.2302	0.2588	0.2976	0.5474	0.7271	0.264
1.0e-5	0.0759	0.0821	1,0000	0.3467	0.5747	0.7432	0.491
1.0e-6	NA	0.0174	NA	NA	0.5581	0.7373	0.055
1.0e-7	NA	0.0000	NA	NA	0.5580	0.7475	NA
			AdaBoostM1				
0.15	0.3486	0.9230	0.9649	0.9500	0.4514	0.6122	0.408
0.1	0.3608	0.1176	0.7730	0.9378	0.4541	0.5892	0.402
0.05	0.3662	0.1054	0.6054	0.7797	0.4486	0.6054	0.386
0.01	0.3730	0.8392	0.9243	0.6365	0.4270	0.5297	0.393
0.0010	0.3878	0.3230	0.7635	0.1932	0.5243	0.6216	0.437
1.0e-4	0.3203	0.6486	0.9797	0.4270	0.5662	0.6716	0.405
1.0e-5	0.2689	0.1757	1,0000	0.2568	0.6189	0.6581	0.395
1.0e-6	NA	0.2851	NA	NA	0.6027	0.6527	0.166
1.0e-7	NA	0.2595	NA	NA	0.5703	0.6595	NA
			C4.5				
0.15	0.4791	0.7056	0.0015	0.3544	0.4173	0.4985	0.435
0.1	0.4615	0.0430	0.1455	0.4704	0.4867	0.4410	0.427
0.05	0.4151	0.0043	0.9971	0.6328	0.4834	0.4636	0.524
0.01	0.4857	0.0079	0.7683	0.6124	0.5712	0.5943	0.456
0.0010	0.4938	0.3960	0.1717	0.8249	0.4759	0.5804	0.459
1.0e-4	0.4019	0.4947	1,0000	0.4742	0.5174	0.5994	0.429
1.0e-5	0.2014	0.1495	1,0000	0.1865	0.5535	0.6011	0.271
1.0e-6	NA	0.0108	NA	NA	0.4910	0.5961	0.429
1.0e-7	NA	0.0581	NA	NA	0.4836	0.6188	NA
			20RF				
0.15	0.0027	0.4041	0.5392	0.0865	0.0081	0.0095	0.032
0.1	0.0149	0.2784	0.1959	0.1108	0.0108	0.0054	0.025
0.05	0.0122	0.1730	0.2365	0.0419	0.0068	0.0095	0.032
0.01	0.0081	0.1203	0.3892	0.1730	0.0176	0.0189	0.044
0.0010	0.0135	0.2676	0.2041	0.0068	0.1203	0.2108	0.124
1.0e-4	0.1230	0.0784	0.6068	0.2459	0.3797	0.4892	0.314
1.0e-5	0.0986	0.2865	1,0000	0.2554	0.4649	0.5000	0.381
1.0e-6	NA	0.0000	NA	NA	0.4689	0.4973	0.168
1.0e-7	NA	0.0662	NA	NA	0.4757	0.5500	NA

Table S 45: Specificity obtained by all the algorithms under the haplotype-based approach with 1-SNP haplotype length, additive genetic model and holdout sampling. Results for each p-value threshold (column 1) are shown. The maximum p-value threshold used was 0.15.

p-value threshold	BD	CAD	HT	IBD	RA	T1D	T2D
			NBC				
0.15	0.1149	1,0000	1,0000	0.3973	0.1054	0.2270	0.154
0.1	0.1162	1,0000	1,0000	0.7176	0.1176	0.2486	0.163
0.05	0.1459	1,0000	1,0000	0.7324	0.1419	0.3095	0.173
0.01	0.1797	1,0000	1,0000	0.9500	0.2324	0.4716	0.174
0.0010	0.1905	1,0000	1,0000	0.2189	0.3608	0.6311	0.190
1.0e-4	0.0824	0.9473	0.9851	0.1216	0.4392	0.6432	0.071
1.0e-5	0.0000	0.0095	0.9446	0.0324	0.4257	0.6284	0.021
1.0e-6	NA	0.0000	NA	NA	0.4041	0.6135	0.000
1.0e-7	NA	0.0000	NA	NA	0.3919	0.5635	NA
			sSVM				
0.15	0.0216	0.0000	0.0000	0.1189	0.0122	0.0432	0.066
0.1	0.0568	0.0000	0.0000	0.3622	0.0459	0.1149	0.105
0.05	0.0905	0.0000	0.0000	0.3797	0.0811	0.1554	0.116
0.01	0.1189	0.0068	0.0068	0.7392	0.1338	0.2365	0.132
0.0010	0.1689	0.3054	0.4676	0.1486	0.1932	0.4095	0.173
1.0e-4	0.0851	0.1297	0.1392	0.1500	0.3122	0.4716	0.145
1.0e-5	0.0527	0.0581	0.9446	0.1865	0.3743	0.5203	0.225
1.0e-6	NA	0.0149	NA	NA	0.3635	0.5081	0.033
1.0e-7	NA	0.0000	NA	NA	0.3703	0.5000	NA
			AdaBoostM1				
0.15	0.1486	0.7162	0.7892	0.7568	0.2338	0.3635	0.186
0.1	0.1324	0.0365	0.4635	0.7392	0.2284	0.3473	0.171
0.05	0.1797	0.0351	0.2878	0.4946	0.2311	0.3635	0.160
0.01	0.1459	0.5541	0.7297	0.3432	0.2027	0.2892	0.156
0.0010	0.1514	0.1000	0.3770	0.0527	0.2459	0.3635	0.152
1.0e-4	0.0243	0.1784	0.7257	0.0527	0.1541	0.3689	0.016
1.0e-5	0.0000	0.0000	0.9446	0.0000	0.0473	0.3608	0.002
1.0e-6	NA	0.0000	NA	NA	0.0527	0.3500	0.000
1.0e-7	NA	0.0000	NA	NA	0.0135	0.3257	NA
			C4.5				
0.15	0.1946	0.6932	0.0014	0.1730	0.1986	0.2216	0.225
0.1	0.2095	0.0311	0.0865	0.2243	0.2459	0.2108	0.170
0.05	0.1851	0.0041	0.9176	0.3027	0.2459	0.2811	0.218
0.01	0.2365	0.0068	0.4257	0.3068	0.3216	0.2689	0.237
0.0010	0.2135	0.0878	0.0081	0.5770	0.2041	0.3527	0.179
1.0e-4	0.0892	0.1838	0.9473	0.1365	0.2770	0.3824	0.051
1.0e-5	0.0000	0.0162	0.9446	0.0000	0.3068	0.3432	0.000
1.0e-6	NA	0.0000	NA	NA	0.2108	0.3703	0.000
1.0e-7	NA	0.0000	NA	NA	0.1432	0.3824	NA
			20RF				
0.15	0.0000	0.0000	0.0000	0.0000	0.0000	0.0000	0.000
0.1	0.0000	0.0000	0.0000	0.0000	0.0000	0.0000	0.000
0.05	0.0000	0.0000	0.0000	0.0000	0.0000	0.0000	0.000
0.01	0.0000	0.0000	0.0000	0.0000	0.0000	0.0000	0.000
0.0010	0.0000	0.0000	0.0000	0.0000	0.0000	0.0000	0.000
1.0e-4	0.0000	0.0000	0.0000	0.0000	0.0000	0.0054	0.001
1.0e-5	0.0000	0.0027	0.9446	0.0000	0.0000	0.0176	0.006
1.0e-6	NA	0.0000	NA	NA	0.0000	0.0324	0.000
1.0e-7	NA	0.0000	NA	NA	0.0000	0.0554	NA

Table S 46: Specificity obtained by all the algorithms under the haplotype-based approach with 1-SNP haplotype length, dominant genetic model and holdout sampling. Results for each p-value threshold (column 1) are shown. The maximum p-value threshold used was 0.15.

p-value threshold	BD	CAD	HT	IBD	RA	T1D	T2D
NBC							
0.15	0.5500	1,0000	1,0000	0.8797	0.5392	0.6378	0.614
0.1	0.5527	1,0000	1,0000	0.9743	0.5595	0.6608	0.625
0.05	0.5649	1,0000	1,0000	0.9797	0.6068	0.7068	0.650
0.01	0.6405	1,0000	1,0000	1,0000	0.6716	0.8041	0.671
0.0010	0.6865	1,0000	1,0000	0.7378	0.7122	0.8730	0.740
1.0e-4	0.8068	1,0000	1,0000	0.7635	0.7311	0.8797	0.859
1.0e-5	1,0000	1,0000	1,0000	1,0000	0.7351	0.8770	0.944
1.0e-6	NA	1,0000	NA	NA	0.7432	0.8649	1,000
1.0e-7	NA	1,0000	NA	NA	0.7270	0.8541	NA
sSVM							
0.15	0.2189	0.0000	0.0000	0.5635	0.1932	0.3541	0.418
0.1	0.4014	0.0000	0.0000	0.8500	0.3919	0.4757	0.527
0.05	0.4824	0.0000	0.0000	0.8486	0.4757	0.5459	0.562
0.01	0.5716	0.1716	0.1162	0.9824	0.5946	0.6824	0.575
0.0010	0.6122	0.7932	0.9149	0.6162	0.6784	0.7919	0.673
1.0e-4	0.5419	0.5662	0.6014	0.6459	0.7419	0.8230	0.593
1.0e-5	0.3581	0.3500	1,0000	0.6486	0.7230	0.8203	0.766
1.0e-6	NA	0.1608	NA	NA	0.7122	0.8189	0.429
1.0e-7	NA	0.0676	NA	NA	0.7068	0.8311	NA
AdaBoostM1							
0.15	0.6500	0.9730	0.9932	0.9865	0.7135	0.8081	0.674
0.1	0.6581	0.3676	0.9203	0.9743	0.7297	0.7905	0.689
0.05	0.6595	0.3581	0.8135	0.9189	0.6986	0.7757	0.671
0.01	0.6608	0.9392	0.9784	0.8514	0.7135	0.7649	0.677
0.0010	0.6838	0.6541	0.9514	0.4486	0.7892	0.8365	0.768
1.0e-4	0.8635	0.9662	1,0000	0.9014	0.9378	0.8865	0.918
1.0e-5	1,0000	0.9554	1,0000	1,0000	0.9703	0.9027	0.977
1.0e-6	NA	1,0000	NA	NA	0.9784	0.9216	1,000
1.0e-7	NA	1,0000	NA	NA	0.9811	0.9203	NA
C4.5							
0.15	0.7095	0.9608	0.0878	0.6824	0.7392	0.7189	0.698
0.1	0.7122	0.3108	0.4919	0.7338	0.7473	0.7135	0.698
0.05	0.7027	0.0662	0.9973	0.8243	0.7230	0.7568	0.725
0.01	0.7162	0.1514	0.8716	0.7689	0.7892	0.7757	0.706
0.0010	0.7459	0.4743	0.6162	0.9514	0.7419	0.8054	0.685
1.0e-4	0.6662	0.6541	1,0000	0.7041	0.7919	0.8270	0.870
1.0e-5	1,0000	0.8770	1,0000	1,0000	0.7824	0.7757	0.993
1.0e-6	NA	0.7622	NA	NA	0.7851	0.7973	1,000
1.0e-7	NA	1,0000	NA	NA	0.7946	0.7959	NA
20RF							
0.15	1,0000	1,0000	1,0000	1,0000	1,0000	1,0000	1,000
0.1	1,0000	0.9986	0.9959	1,0000	1,0000	1,0000	1,000
0.05	1,0000	1,0000	1,0000	1,0000	1,0000	1,0000	1,000
0.01	1,0000	1,0000	1,0000	1,0000	1,0000	1,0000	1,000
0.0010	1,0000	0.9973	1,0000	0.9959	1,0000	0.9973	1,000
1.0e-4	0.9878	0.9973	1,0000	1,0000	0.9959	0.9865	0.979
1.0e-5	0.9689	0.9595	1,0000	1,0000	0.9716	0.9770	0.963
1.0e-6	NA	0.5892	NA	NA	0.9757	0.9770	1,000
1.0e-7	NA	1,0000	NA	NA	0.9649	0.9730	NA

Table S 47: Specificity obtained by all the algorithms under the haplotype-based approach with 1-SNP haplotype length, recessive genetic model and holdout sampling. Results for each p-value threshold (column 1) are shown. The maximum p-value threshold used was 0.15.

p-value threshold	BD	CAD	HT	IBD	RA	T1D	T2D
				NBC			
0.15	0.2730	1,0000	1,0000	0.5230	0.2919	0.3905	0.356
0.1	0.3081	1,0000	1,0000	0.7473	0.3257	0.4459	0.374
0.05	0.3378	1,0000	1,0000	0.9716	0.3905	0.5216	0.387
0.01	0.4095	1,0000	1,0000	1,0000	0.5203	0.6662	0.435
0.0010	0.4351	1,0000	1,0000	0.3757	0.5919	0.7284	0.479
1.0e-4	0.4351	0.9959	0.9662	0.4581	0.6284	0.7257	0.491
1.0e-5	0.3108	0.6527	0.0878	0.3608	0.6243	0.7297	0.368
1.0e-6	0.2662	0.7905	0.0946	0.0527	0.6041	0.7189	0.364
1.0e-7	0.1878	0.9892	NA	0.0000	0.5824	0.6986	NA
				sSVM			
0.15	0.0392	0.0000	0.0000	0.0459	0.0306	0.0738	0.121
0.1	0.0989	0.0000	0.0000	0.2720	0.0892	0.1607	0.174
0.05	0.1674	0.0000	0.0000	0.7003	0.1419	0.2440	0.217
0.01	0.2551	0.0191	0.0728	0.9833	0.2401	0.4271	0.266
0.0010	0.3437	0.1608	1,0000	0.2396	0.4211	0.6643	0.337
1.0e-4	0.2885	0.0000	0.8444	0.3185	0.5583	0.7405	0.309
1.0e-5	0.1215	0.9855	0.0000	0.4697	0.5921	0.7451	0.329
1.0e-6	0.0920	0.9554	0.0000	0.0014	0.5919	0.7613	0.000
1.0e-7	0.0115	0.9855	NA	0.0000	0.5799	0.7573	NA
				AdaBoostM1			
0.15	0.4284	0.1986	0.4797	0.8811	0.4865	0.6135	0.454
0.1	0.4203	0.8662	0.0419	0.8635	0.5068	0.5851	0.425
0.05	0.4122	0.9216	0.8122	0.9230	0.4838	0.6054	0.425
0.01	0.4595	0.9919	0.8554	0.9135	0.4757	0.5946	0.445
0.0010	0.4757	0.9946	0.9986	0.5216	0.5486	0.6851	0.494
1.0e-4	0.4527	0.9527	0.4851	0.4919	0.5865	0.7189	0.467
1.0e-5	0.3122	1,0000	0.0838	0.4135	0.6257	0.7108	0.281
1.0e-6	0.2662	0.7041	0.0946	0.0527	0.6095	0.6986	0.041
1.0e-7	0.1878	0.9892	NA	0.0000	0.5946	0.6973	NA
				C4.5			
0.15	0.6056	1,0000	1,0000	1,0000	0.6118	0.6096	0.518
0.1	0.5206	1,0000	1,0000	1,0000	0.6316	0.6642	0.564
0.05	0.5595	1,0000	1,0000	1,0000	0.6128	0.6212	0.490
0.01	0.5433	1,0000	1,0000	0.0071	0.5942	0.5531	0.463
0.0010	0.5496	0.6852	0.9816	0.3920	0.5543	0.6606	0.507
1.0e-4	0.4891	1,0000	0.1730	0.5467	0.5566	0.6280	0.467
1.0e-5	0.3410	0.2507	0.0946	0.3473	0.5866	0.6662	0.239
1.0e-6	0.2662	0.1986	0.0946	0.0541	0.5398	0.6347	0.041
1.0e-7	0.1878	0.9635	NA	0.0000	0.5339	0.6559	NA
				20RF			
0.15	0.0081	0.0554	0.0446	0.3095	0.0122	0.0054	0.029
0.1	0.0162	0.4162	0.3757	0.1054	0.0095	0.0176	0.028
0.05	0.0135	0.0676	0.2000	0.5378	0.0095	0.0108	0.040
0.01	0.0108	0.4216	0.0243	0.2446	0.0311	0.0216	0.036
0.0010	0.0473	0.5027	0.5203	0.0514	0.1405	0.2770	0.082
1.0e-4	0.1351	0.2527	0.7662	0.2892	0.3703	0.4568	0.233
1.0e-5	0.2014	0.6959	0.0784	0.3162	0.4432	0.4770	0.301
1.0e-6	0.2662	0.7770	0.0946	0.0527	0.4541	0.5054	0.041
1.0e-7	0.1878	0.9635	NA	0.0000	0.4770	0.5581	NA

Table S 48: Specificity obtained by all the algorithms under the haplotype-based approach with 2-SNP haplotype length, additive genetic model and holdout sampling. Results for each p-value threshold (column 1) are shown. The maximum p-value threshold used was 0.15.

p-value threshold	BD	CAD	HT	IBD	RA	T1D	T2D
				NBC			
0.15	0.1392	1,0000	1,0000	0.2649	0.1446	0.2189	0.185
0.1	0.1500	1,0000	1,0000	0.4378	0.1608	0.2608	0.190
0.05	0.1757	1,0000	1,0000	0.8203	0.1946	0.3068	0.198
0.01	0.2243	1,0000	1,0000	0.9743	0.2932	0.4824	0.223
0.0010	0.2473	1,0000	1,0000	0.1500	0.4149	0.6527	0.235
1.0e-4	0.2095	0.9135	0.7257	0.1378	0.4500	0.6703	0.136
1.0e-5	0.1203	0.1027	0.0000	0.0797	0.4405	0.6486	0.027
1.0e-6	0.0743	0.1000	0.0000	0.0014	0.4284	0.6216	0.012
1.0e-7	0.0095	0.0000	NA	0.0000	0.4230	0.5878	NA
				sSVM			
0.15	0.0284	0.0000	0.0000	0.0324	0.0230	0.0514	0.077
0.1	0.0635	0.0000	0.0000	0.1378	0.0581	0.0973	0.100
0.05	0.1041	0.0000	0.0000	0.3568	0.0878	0.1378	0.121
0.01	0.1365	0.0149	0.0473	0.7176	0.1311	0.2216	0.145
0.0010	0.1797	0.0932	0.8770	0.1324	0.2270	0.3824	0.175
1.0e-4	0.1622	0.0000	0.4986	0.1649	0.3108	0.4743	0.164
1.0e-5	0.0946	0.7324	0.0000	0.2405	0.3865	0.5135	0.168
1.0e-6	0.0743	0.7527	0.0000	0.0014	0.3743	0.5216	0.000
1.0e-7	0.0095	0.7324	NA	0.0000	0.3824	0.5230	NA
				AdaBoostM1			
0.15	0.2027	0.0757	0.2162	0.6459	0.2527	0.3784	0.212
0.1	0.2149	0.6014	0.0095	0.6203	0.2635	0.3459	0.217
0.05	0.1986	0.7162	0.5081	0.7108	0.2473	0.3500	0.187
0.01	0.2189	0.9189	0.5743	0.6797	0.2432	0.3527	0.204
0.0010	0.2365	0.9135	0.9770	0.2554	0.2824	0.3919	0.208
1.0e-4	0.1473	0.6473	0.0189	0.0730	0.2459	0.4257	0.094
1.0e-5	0.0892	0.9527	0.0000	0.0284	0.1676	0.4243	0.021
1.0e-6	0.0730	0.0797	0.0000	0.0014	0.1216	0.3946	0.000
1.0e-7	0.0095	0.0000	NA	0.0000	0.0905	0.3689	NA
				C4.5			
0.15	0.2554	1,0000	1,0000	0.9973	0.2716	0.2500	0.310
0.1	0.2838	1,0000	1,0000	0.9973	0.3122	0.3405	0.336
0.05	0.3257	1,0000	1,0000	0.9905	0.3027	0.3243	0.317
0.01	0.3162	1,0000	1,0000	0.0068	0.3351	0.3932	0.306
0.0010	0.3108	0.3811	0.8068	0.2108	0.3230	0.3959	0.260
1.0e-4	0.2446	1,0000	0.0122	0.2149	0.2973	0.4095	0.166
1.0e-5	0.0973	0.0095	0.0000	0.0270	0.2622	0.4243	0.027
1.0e-6	0.0743	0.0000	0.0000	0.0014	0.2905	0.3824	0.000
1.0e-7	0.0095	0.0000	NA	0.0000	0.2703	0.3405	NA
				20RF			
0.15	0.0000	0.0000	0.0000	0.0000	0.0000	0.0000	0.000
0.1	0.0000	0.0000	0.0000	0.0000	0.0000	0.0000	0.000
0.05	0.0000	0.0000	0.0000	0.0000	0.0000	0.0000	0.000
0.01	0.0000	0.0000	0.0000	0.0000	0.0000	0.0000	0.000
0.0010	0.0000	0.0000	0.0000	0.0000	0.0000	0.0000	0.000
1.0e-4	0.0041	0.0000	0.0000	0.0000	0.0000	0.0000	0.000
1.0e-5	0.0338	0.0000	0.0014	0.0257	0.0000	0.0122	0.008
1.0e-6	0.0703	0.0027	0.0000	0.0014	0.0000	0.0351	0.000
1.0e-7	0.0095	0.0716	NA	0.0000	0.0000	0.0419	NA

Table S 49: Specificity obtained by all the algorithms under the haplotype-based approach with 2-SNP haplotype length, dominant genetic model and holdout sampling. Results for each p-value threshold (column 1) are shown. The maximum p-value threshold used was 0.15.

p-value threshold	BD	CAD	HT	IBD	RA	T1D	T2D
				NBC			
0.15	0.5716	1,0000	1,0000	0.7797	0.5622	0.6459	0.632
0.1	0.5676	1,0000	1,0000	0.8959	0.5851	0.6851	0.648
0.05	0.5878	1,0000	1,0000	0.9932	0.6311	0.7419	0.656
0.01	0.6676	1,0000	1,0000	1,0000	0.7041	0.8514	0.697
0.0010	0.7122	1,0000	1,0000	0.6824	0.7392	0.8865	0.740
1.0e-4	0.7635	1,0000	1,0000	0.7973	0.7446	0.8865	0.864
1.0e-5	0.9014	0.9946	0.9959	0.9986	0.7459	0.8851	0.923
1.0e-6	0.9986	1,0000	1,0000	0.9986	0.7419	0.8757	1,000
1.0e-7	0.9986	1,0000	NA	0.9973	0.7405	0.8622	NA
				sSVM			
0.15	0.3041	0.0000	0.0000	0.3257	0.2716	0.3554	0.441
0.1	0.4216	0.0000	0.0000	0.6311	0.4068	0.4919	0.525
0.05	0.4824	0.0000	0.0000	0.8473	0.4689	0.5730	0.562
0.01	0.6014	0.2351	0.3973	0.9878	0.5851	0.7027	0.598
0.0010	0.6568	0.5135	1,0000	0.5797	0.6878	0.8068	0.655
1.0e-4	0.6000	0.0716	0.9081	0.6473	0.7541	0.8338	0.632
1.0e-5	0.3162	0.9892	0.0946	0.7284	0.7338	0.8243	0.656
1.0e-6	0.2662	0.9649	0.0946	0.0541	0.7419	0.8365	0.041
1.0e-7	0.1878	0.9892	NA	0.0000	0.7230	0.8324	NA
				AdaBoostM1			
0.15	0.6973	0.4986	0.7257	0.9541	0.7365	0.8041	0.732
0.1	0.6797	0.9541	0.2176	0.9608	0.7392	0.8081	0.712
0.05	0.6703	0.9851	0.9162	0.9770	0.7149	0.7959	0.697
0.01	0.7027	0.9986	0.9595	0.9757	0.7270	0.8041	0.721
0.0010	0.7595	1,0000	1,0000	0.7716	0.7986	0.8865	0.797
1.0e-4	0.8554	0.9986	0.9311	0.9162	0.9068	0.9257	0.916
1.0e-5	0.9365	1,0000	0.8419	0.9986	0.9514	0.9135	0.987
1.0e-6	0.9986	0.9797	1,0000	0.9986	0.9486	0.9338	0.997
1.0e-7	0.9986	1,0000	NA	0.9973	0.9649	0.9230	NA
				C4.5			
0.15	0.7270	1,0000	1,0000	1,0000	0.7230	0.7203	0.778
0.1	0.7486	1,0000	1,0000	1,0000	0.7608	0.7743	0.786
0.05	0.7932	1,0000	1,0000	1,0000	0.7676	0.7919	0.812
0.01	0.7770	1,0000	1,0000	0.1392	0.8081	0.8459	0.795
0.0010	0.7784	0.8514	0.9838	0.7149	0.7797	0.8541	0.768
1.0e-4	0.7203	1,0000	0.2081	0.7419	0.7757	0.8351	0.762
1.0e-5	0.9608	0.7595	1,0000	0.9986	0.7959	0.8392	1,000
1.0e-6	1,0000	0.6811	1,0000	1,0000	0.7824	0.7986	1,000
1.0e-7	1,0000	1,0000	NA	1,0000	0.8243	0.7986	NA
				20RF			
0.15	1,0000	0.9851	0.9865	1,0000	1,0000	1,0000	1,000
0.1	1,0000	1,0000	1,0000	1,0000	1,0000	1,0000	1,000
0.05	1,0000	0.9905	0.9986	1,0000	1,0000	1,0000	1,000
0.01	1,0000	1,0000	0.9635	1,0000	1,0000	1,0000	1,000
0.0010	1,0000	1,0000	1,0000	0.9973	1,0000	0.9932	1,000
1.0e-4	1,0000	1,0000	1,0000	1,0000	0.9946	0.9892	1,000
1.0e-5	1,0000	0.9959	0.9716	0.9986	0.9865	0.9797	0.995
1.0e-6	0.9986	1,0000	1,0000	0.9986	0.9770	0.9838	0.997
1.0e-7	0.9986	1,0000	NA	0.9973	0.9635	0.9770	NA

Table S 50: Specificity obtained by all the algorithms under the haplotype-based approach with 2-SNP haplotype length, recessive genetic model and holdout sampling. Results for each p-value threshold (column 1) are shown. The maximum p-value threshold used was 0.15.

p-value threshold	BD	CAD	HT	IBD	RA	T1D	T2D
NBC							
0.15	0.2824	1,0000	1,0000	0.9784	0.3054	0.4392	0.351
0.1	0.3095	1,0000	1,0000	0.9392	0.3486	0.4716	0.379
0.05	0.3541	1,0000	1,0000	0.9230	0.4149	0.5527	0.405
0.01	0.4108	1,0000	1,0000	0.9324	0.5351	0.6838	0.437
0.0010	0.4703	1,0000	0.9311	0.4541	0.6000	0.7311	0.510
1.0e-4	0.4203	0.9014	1,0000	0.4716	0.6149	0.7230	0.474
1.0e-5	0.3041	0.0541	0.9081	0.3865	0.6230	0.7243	0.428
1.0e-6	0.2743	0.0784	0.0000	0.2973	0.6095	0.7203	0.183
1.0e-7	0.1730	0.0973	NA	0.0000	0.5878	0.7122	NA
sSVM							
0.15	0.0596	0.0000	0.0000	0.5777	0.0600	0.1127	0.121
0.1	0.1102	0.0000	0.0000	0.1840	0.1062	0.2032	0.194
0.05	0.1843	0.0000	0.0000	0.3869	0.1719	0.2813	0.219
0.01	0.2247	0.6807	0.5268	0.4548	0.2782	0.3504	0.245
0.0010	0.3922	0.9070	0.7181	0.5143	0.4251	0.6513	0.413
1.0e-4	0.3406	0.0015	0.1011	0.3821	0.5519	0.7182	0.335
1.0e-5	0.1203	0.9360	1,0000	0.1429	0.5992	0.7556	0.139
1.0e-6	0.1045	0.7500	0.0000	0.0477	0.5652	0.7435	0.027
1.0e-7	0.0113	0.0000	NA	0.0000	0.5847	0.7544	NA
AdaBoostM1							
0.15	0.4500	0.9405	0.1378	0.8459	0.5095	0.6392	0.424
0.1	0.4405	0.4432	0.7081	0.5514	0.4973	0.6230	0.390
0.05	0.4486	0.9068	0.8216	0.7459	0.5135	0.6095	0.421
0.01	0.4027	0.8676	0.9865	0.4378	0.4459	0.5473	0.418
0.0010	0.5054	0.5162	0.3676	0.8730	0.5446	0.6730	0.493
1.0e-4	0.4662	1,0000	1,0000	0.4676	0.6014	0.7162	0.441
1.0e-5	0.3919	0.3581	0.9203	0.4649	0.6108	0.7041	0.374
1.0e-6	0.2703	0.3919	0.0000	0.2973	0.5824	0.7149	0.183
1.0e-7	0.1730	0.0973	NA	0.0000	0.5622	0.7081	NA
C4.5							
0.15	0.5151	1,0000	1,0000	1,0000	0.5579	0.5767	0.449
0.1	0.5243	1,0000	1,0000	1,0000	0.5919	0.5489	0.427
0.05	0.5083	1,0000	1,0000	0.9832	0.5353	0.6104	0.490
0.01	0.4591	1,0000	1,0000	0.7533	0.5789	0.5823	0.409
0.0010	0.5596	0.9041	0.2362	0.4529	0.5160	0.6541	0.502
1.0e-4	0.4847	1,0000	0.4189	0.4966	0.5477	0.6861	0.483
1.0e-5	0.3194	0.8062	0.9973	0.4135	0.5784	0.6942	0.308
1.0e-6	0.2703	0.4365	0.0000	0.2973	0.5429	0.6448	0.270
1.0e-7	0.1730	0.0000	NA	0.0000	0.5458	0.6283	NA
20RF							
0.15	0.0081	0.1243	0.1527	0.0986	0.0054	0.0135	0.044
0.1	0.0081	0.4000	0.5851	0.0446	0.0081	0.0108	0.033
0.05	0.0162	0.1568	0.1243	0.2122	0.0108	0.0027	0.040
0.01	0.0176	0.1216	0.0230	0.1824	0.0216	0.0365	0.040
0.0010	0.0811	0.4527	0.2743	0.2243	0.1838	0.2797	0.106
1.0e-4	0.1459	0.7459	0.2527	0.2797	0.3486	0.4608	0.236
1.0e-5	0.2095	0.7635	0.8514	0.3392	0.4405	0.5027	0.316
1.0e-6	0.2703	0.7770	0.0554	0.2973	0.4595	0.4797	0.183
1.0e-7	0.1730	0.0081	NA	0.0000	0.4662	0.5068	NA

Table S 51: Specificity obtained by all the algorithms under the haplotype-based approach with 3-SNP haplotype length, additive genetic model and holdout sampling. Results for each p-value threshold (column 1) are shown. The maximum p-value threshold used was 0.15.

p-value threshold	BD	CAD	HT	IBD	RA	T1D	T2D
			NBC				
0.15	0.1243	1,0000	1,0000	0.8622	0.1581	0.2608	0.194
0.1	0.1595	1,0000	1,0000	0.7419	0.1757	0.2838	0.191
0.05	0.1946	1,0000	1,0000	0.7297	0.2297	0.3608	0.201
0.01	0.2257	1,0000	1,0000	0.7378	0.3311	0.5311	0.237
0.0010	0.2365	1,0000	0.6676	0.1973	0.4257	0.6662	0.241
1.0e-4	0.2014	0.4041	0.9878	0.1365	0.4649	0.6649	0.140
1.0e-5	0.1378	0.0000	0.0000	0.0784	0.4527	0.6568	0.066
1.0e-6	0.0905	0.0000	0.0000	0.0108	0.4419	0.6378	0.000
1.0e-7	0.0095	0.0000	NA	0.0000	0.4311	0.5986	NA
			sSVM				
0.15	0.0419	0.0000	0.0000	0.3014	0.0419	0.0730	0.077
0.1	0.0703	0.0000	0.0000	0.1054	0.0676	0.1216	0.112
0.05	0.1108	0.0000	0.0000	0.1919	0.1027	0.1581	0.118
0.01	0.1203	0.3486	0.2527	0.2311	0.1500	0.1757	0.128
0.0010	0.2041	0.5405	0.3959	0.2676	0.2338	0.3811	0.212
1.0e-4	0.1905	0.0014	0.0608	0.1905	0.3378	0.4581	0.175
1.0e-5	0.0959	0.6919	0.8932	0.0878	0.3919	0.5054	0.082
1.0e-6	0.0851	0.4216	0.0000	0.0351	0.3689	0.5054	0.020
1.0e-7	0.0095	0.0000	NA	0.0000	0.3824	0.5230	NA
			AdaBoostM1				
0.15	0.2189	0.7405	0.0486	0.5905	0.2541	0.3959	0.200
0.1	0.2203	0.2149	0.4378	0.2865	0.2635	0.3676	0.185
0.05	0.2041	0.6743	0.5662	0.4635	0.2784	0.3676	0.189
0.01	0.2041	0.6135	0.9108	0.1946	0.2068	0.3122	0.191
0.0010	0.2446	0.2068	0.1324	0.5892	0.2608	0.3703	0.206
1.0e-4	0.1541	0.9851	0.9757	0.0892	0.2473	0.4054	0.066
1.0e-5	0.1068	0.0162	0.0000	0.0297	0.1257	0.3959	0.014
1.0e-6	0.0770	0.0000	0.0000	0.0108	0.0716	0.3905	0.000
1.0e-7	0.0095	0.0000	NA	0.0000	0.0297	0.3662	NA
			C4.5				
0.15	0.2811	1,0000	1,0000	0.9973	0.3257	0.3311	0.239
0.1	0.3054	1,0000	1,0000	0.9973	0.3068	0.2892	0.267
0.05	0.2838	1,0000	1,0000	0.7905	0.3419	0.3432	0.297
0.01	0.2676	1,0000	1,0000	0.4554	0.3203	0.3608	0.237
0.0010	0.2973	0.5608	0.1946	0.3432	0.2635	0.3230	0.298
1.0e-4	0.2392	0.9986	0.2446	0.2014	0.3270	0.4095	0.208
1.0e-5	0.1081	0.5162	0.8149	0.0338	0.2851	0.4014	0.035
1.0e-6	0.0770	0.0041	0.0000	0.0108	0.2662	0.3676	0.000
1.0e-7	0.0095	0.0000	NA	0.0000	0.2257	0.3595	NA
			20RF				
0.15	0.0000	0.0000	0.0000	0.0000	0.0000	0.0000	0.000
0.1	0.0000	0.0000	0.0000	0.0000	0.0000	0.0000	0.000
0.05	0.0000	0.0000	0.0000	0.0000	0.0000	0.0000	0.000
0.01	0.0000	0.0000	0.0000	0.0000	0.0000	0.0000	0.000
0.0010	0.0000	0.0000	0.0000	0.0000	0.0000	0.0000	0.000
1.0e-4	0.0095	0.0000	0.0000	0.0014	0.0000	0.0054	0.000
1.0e-5	0.0473	0.0000	0.0054	0.0149	0.0000	0.0095	0.004
1.0e-6	0.0649	0.2216	0.0000	0.0108	0.0000	0.0311	0.000
1.0e-7	0.0095	0.0000	NA	0.0000	0.0000	0.0378	NA

Table S 52: Specificity obtained by all the algorithms under the haplotype-based approach with 3-SNP haplotype length, dominant genetic model and holdout sampling. Results for each p-value threshold (column 1) are shown. The maximum p-value threshold used was 0.15.

p-value threshold	BD	CAD	HT	IBD	RA	T1D	T2D
NBC							
0.15	0.5608	1,0000	1,0000	0.9973	0.5878	0.6878	0.620
0.1	0.5784	1,0000	1,0000	0.9865	0.6162	0.7135	0.629
0.05	0.5986	1,0000	1,0000	0.9757	0.6649	0.7716	0.660
0.01	0.6689	1,0000	1,0000	0.9797	0.7149	0.8568	0.700
0.0010	0.7338	1,0000	0.9851	0.7297	0.7243	0.8959	0.783
1.0e-4	0.7568	0.9986	1,0000	0.8446	0.7486	0.8892	0.866
1.0e-5	0.8297	0.8230	1,0000	0.9986	0.7378	0.8878	0.902
1.0e-6	0.9986	0.7865	1,0000	0.9986	0.7432	0.8784	1,000
1.0e-7	0.9986	1,0000	NA	0.9973	0.7554	0.8689	NA
sSVM							
0.15	0.3392	0.0000	0.0000	0.7797	0.3432	0.4257	0.443
0.1	0.4324	0.0000	0.0000	0.5324	0.4311	0.5230	0.536
0.05	0.5095	0.0000	0.0000	0.6959	0.5054	0.5959	0.577
0.01	0.5851	0.8365	0.7730	0.7230	0.6108	0.6743	0.605
0.0010	0.6838	0.9446	0.8446	0.7473	0.6838	0.7959	0.698
1.0e-4	0.6311	0.0757	0.4595	0.6919	0.7257	0.8203	0.651
1.0e-5	0.2986	0.9527	1,0000	0.4730	0.7378	0.8365	0.489
1.0e-6	0.2703	0.8595	0.0635	0.2986	0.7162	0.8257	0.270
1.0e-7	0.1730	0.0000	NA	0.0000	0.7284	0.8297	NA
AdaBoostM1							
0.15	0.7027	0.9784	0.4284	0.9541	0.7351	0.8230	0.691
0.1	0.7041	0.7176	0.8986	0.7865	0.7351	0.8189	0.712
0.05	0.6986	0.9784	0.9392	0.8946	0.7608	0.8203	0.740
0.01	0.6649	0.9581	0.9946	0.6811	0.7257	0.7784	0.689
0.0010	0.7892	0.7905	0.7014	0.9581	0.7905	0.8568	0.804
1.0e-4	0.8568	1,0000	1,0000	0.9068	0.8946	0.9297	0.928
1.0e-5	0.9446	0.9649	1,0000	0.9986	0.9446	0.9338	0.981
1.0e-6	0.9986	0.9432	1,0000	0.9986	0.9635	0.9419	0.998
1.0e-7	0.9986	1,0000	NA	0.9973	0.9716	0.9365	NA
C4.5							
0.15	0.7284	1,0000	1,0000	1,0000	0.7865	0.8108	0.733
0.1	0.7959	1,0000	1,0000	1,0000	0.8149	0.7635	0.790
0.05	0.7270	1,0000	1,0000	0.9865	0.8068	0.8027	0.762
0.01	0.7365	1,0000	1,0000	0.8865	0.7905	0.8230	0.733
0.0010	0.7716	0.9405	0.6946	0.8419	0.7568	0.8081	0.764
1.0e-4	0.7000	1,0000	0.7405	0.7473	0.8270	0.8351	0.759
1.0e-5	0.9635	0.9095	1,0000	0.9986	0.7824	0.8338	0.981
1.0e-6	1,0000	1,0000	1,0000	0.9986	0.7865	0.8135	0.997
1.0e-7	1,0000	1,0000	NA	1,0000	0.7865	0.7838	NA
20RF							
0.15	1,0000	0.9878	0.9959	1,0000	1,0000	1,0000	1,000
0.1	1,0000	1,0000	1,0000	1,0000	1,0000	1,0000	1,000
0.05	1,0000	0.9986	0.9932	1,0000	1,0000	1,0000	1,000
0.01	1,0000	0.9973	0.9743	1,0000	1,0000	1,0000	1,000
0.0010	1,0000	1,0000	1,0000	1,0000	1,0000	0.9946	1,000
1.0e-4	1,0000	1,0000	0.9959	1,0000	0.9986	0.9919	1,000
1.0e-5	1,0000	1,0000	1,0000	0.9986	0.9770	0.9811	0.994
1.0e-6	0.9986	0.9892	1,0000	0.9986	0.9730	0.9851	0.994
1.0e-7	0.9986	1,0000	NA	0.9973	0.9635	0.9730	NA

Table S 53: Specificity obtained by all the algorithms under the haplotype-based approach with 3-SNP haplotype length, recessive genetic model and holdout sampling. Results for each p-value threshold (column 1) are shown. The maximum p-value threshold used was 0.15.

p-value threshold	BD	CAD	HT	IBD	RA	T1D	T2D
				NBC			
0.15	0.2851	1,0000	1,0000	0.7081	0.3203	0.4419	0.379
0.1	0.3054	1,0000	1,0000	0.7419	0.3459	0.4838	0.391
0.05	0.3378	1,0000	1,0000	0.4946	0.4216	0.5622	0.402
0.01	0.3973	1,0000	1,0000	0.9338	0.5459	0.6878	0.440
0.0010	0.4689	0.9676	0.9865	0.7622	0.6000	0.7203	0.500
1.0e-4	0.4419	0.0014	0.8622	0.7405	0.6162	0.7216	0.451
1.0e-5	0.2676	0.0027	0.0000	0.5365	0.6068	0.7311	0.386
1.0e-6	0.2014	0.0000	NA	0.2608	0.6135	0.7122	0.354
1.0e-7	0.0730	0.0000	NA	0.0000	0.6162	0.7054	NA
				sSVM			
0.15	0.0671	0.0000	0.0000	0.1671	0.0643	0.1368	0.145
0.1	0.1198	0.0000	0.0000	0.1350	0.1226	0.2121	0.216
0.05	0.1697	0.0000	0.0000	0.0671	0.1709	0.2734	0.250
0.01	0.2370	0.8864	0.0047	0.3003	0.2531	0.4130	0.273
0.0010	0.3652	0.3081	0.2555	0.4448	0.4175	0.6486	0.352
1.0e-4	0.3122	0.0078	0.7018	0.4926	0.5596	0.7106	0.318
1.0e-5	0.1155	0.0466	0.0031	0.3780	0.6039	0.7414	0.303
1.0e-6	0.0483	0.0484	NA	0.0353	0.6325	0.7426	0.305
1.0e-7	0.0000	0.0000	NA	0.0000	0.6280	0.7403	NA
				AdaBoostM1			
0.15	0.4297	0.0486	0.6568	0.7581	0.5000	0.6554	0.431
0.1	0.4662	1,0000	0.2986	0.4270	0.4878	0.6338	0.428
0.05	0.4122	0.8757	0.1892	0.6919	0.4824	0.6270	0.421
0.01	0.4203	0.9932	0.1068	0.7122	0.4743	0.5689	0.397
0.0010	0.4838	0.2878	0.1595	0.9892	0.5068	0.6649	0.491
1.0e-4	0.4338	0.0000	0.7135	0.7000	0.5932	0.6662	0.428
1.0e-5	0.2851	0.0000	0.0000	0.3324	0.5986	0.6878	0.316
1.0e-6	0.2014	0.0000	NA	0.2608	0.5838	0.6919	0.354
1.0e-7	0.0730	0.0000	NA	0.0000	0.5689	0.6703	NA
				C4.5			
0.15	0.5357	1,0000	1,0000	1,0000	0.5571	0.5816	0.509
0.1	0.4707	1,0000	1,0000	1,0000	0.5248	0.5093	0.537
0.05	0.4870	1,0000	1,0000	0.9953	0.4729	0.4855	0.449
0.01	0.5409	1,0000	1,0000	0.9556	0.5047	0.5762	0.451
0.0010	0.5111	0.1902	0.2167	0.9784	0.5462	0.5801	0.421
1.0e-4	0.5042	0.0124	0.7866	0.6977	0.5803	0.6226	0.451
1.0e-5	0.2851	0.0392	0.0189	0.4095	0.5731	0.6394	0.363
1.0e-6	0.2014	0.0851	NA	0.2608	0.5166	0.6220	0.156
1.0e-7	0.0730	0.0068	NA	0.0000	0.4894	0.6068	NA
				20RF			
0.15	0.0122	0.1108	0.1905	0.0973	0.0176	0.0095	0.035
0.1	0.0149	0.2554	0.3297	0.1622	0.0149	0.0108	0.050
0.05	0.0257	0.1419	0.1176	0.0581	0.0270	0.0176	0.036
0.01	0.0189	0.0365	0.0405	0.0730	0.0270	0.0527	0.055
0.0010	0.0568	0.8122	0.4446	0.1230	0.1514	0.2730	0.089
1.0e-4	0.1568	0.0378	0.3676	0.5973	0.3689	0.4635	0.237
1.0e-5	0.2203	0.0041	0.0000	0.3311	0.4203	0.5000	0.245
1.0e-6	0.2014	0.1419	NA	0.2608	0.4608	0.5324	0.295
1.0e-7	0.0730	0.2324	NA	0.0000	0.4770	0.5351	NA

Table S 54: Specificity obtained by all the algorithms under the haplotype-based approach with 4-SNP haplotype length, additive genetic model and holdout sampling. Results for each p-value threshold (column 1) are shown. The maximum p-value threshold used was 0.15.

p-value threshold	BD	CAD	HT	IBD	RA	T1D	T2D
			NBC				
0.15	0.1446	1,0000	1,0000	0.4432	0.1622	0.2689	0.185
0.1	0.1405	1,0000	1,0000	0.4595	0.1878	0.2892	0.187
0.05	0.1865	1,0000	1,0000	0.2378	0.2338	0.3743	0.201
0.01	0.2095	1,0000	0.9689	0.6878	0.3419	0.5581	0.227
0.0010	0.2432	0.8149	0.8514	0.4351	0.4581	0.6500	0.247
1.0e-4	0.1811	0.0000	0.1486	0.2689	0.4622	0.6608	0.101
1.0e-5	0.0838	0.0000	0.0000	0.0419	0.4608	0.6419	0.037
1.0e-6	0.0405	0.0000	NA	0.0000	0.4486	0.6270	0.000
1.0e-7	0.0000	0.0000	NA	0.0000	0.4230	0.6176	NA
			sSVM				
0.15	0.0446	0.0000	0.0000	0.0946	0.0446	0.0865	0.089
0.1	0.0703	0.0000	0.0000	0.0797	0.0784	0.1230	0.120
0.05	0.1014	0.0000	0.0000	0.0432	0.1000	0.1500	0.135
0.01	0.1351	0.5378	0.0041	0.1554	0.1392	0.2149	0.144
0.0010	0.1959	0.1649	0.1405	0.2122	0.2257	0.3568	0.174
1.0e-4	0.1662	0.0068	0.3784	0.2243	0.3297	0.4446	0.168
1.0e-5	0.0865	0.0365	0.0027	0.1946	0.3811	0.4959	0.158
1.0e-6	0.0405	0.0378	NA	0.0270	0.4257	0.5108	0.156
1.0e-7	0.0000	0.0000	NA	0.0000	0.4311	0.5162	NA
			AdaBoostM1				
0.15	0.2081	0.0176	0.3905	0.4676	0.2230	0.3946	0.201
0.1	0.2419	0.9568	0.1419	0.2081	0.2446	0.3770	0.200
0.05	0.2162	0.6189	0.0797	0.4041	0.2351	0.3689	0.202
0.01	0.1959	0.9203	0.0311	0.4311	0.2365	0.3203	0.208
0.0010	0.2203	0.0959	0.0568	0.8757	0.2500	0.3757	0.208
1.0e-4	0.1392	0.0000	0.3041	0.2081	0.2054	0.3851	0.059
1.0e-5	0.0743	0.0000	0.0000	0.0027	0.1635	0.3459	0.008
1.0e-6	0.0405	0.0000	NA	0.0000	0.0689	0.3419	0.000
1.0e-7	0.0000	0.0000	NA	0.0000	0.0338	0.3459	NA
			C4.5				
0.15	0.3216	1,0000	1,0000	1,0000	0.3446	0.2838	0.217
0.1	0.3284	1,0000	1,0000	0.9932	0.3378	0.2838	0.320
0.05	0.3351	1,0000	1,0000	0.8649	0.2919	0.2973	0.239
0.01	0.2878	1,0000	0.9973	0.6689	0.2919	0.3135	0.241
0.0010	0.2595	0.1054	0.1176	0.7743	0.3068	0.3446	0.252
1.0e-4	0.2176	0.0108	0.4676	0.4122	0.3324	0.3405	0.156
1.0e-5	0.0703	0.0014	0.0000	0.0000	0.2919	0.3459	0.025
1.0e-6	0.0405	0.0054	NA	0.0000	0.3054	0.3919	0.000
1.0e-7	0.0000	0.0000	NA	0.0000	0.2189	0.3878	NA
			20RF				
0.15	0.0000	0.0000	0.0000	0.0000	0.0000	0.0000	0.000
0.1	0.0000	0.0000	0.0000	0.0000	0.0000	0.0000	0.000
0.05	0.0000	0.0000	0.0000	0.0000	0.0000	0.0000	0.000
0.01	0.0000	0.0000	0.0000	0.0000	0.0000	0.0000	0.000
0.0010	0.0000	0.0000	0.0000	0.0000	0.0000	0.0000	0.000
1.0e-4	0.0000	0.0000	0.0000	0.0014	0.0000	0.0014	0.000
1.0e-5	0.0378	0.0000	0.0000	0.0068	0.0000	0.0081	0.001
1.0e-6	0.0405	0.0041	NA	0.0000	0.0000	0.0297	0.000
1.0e-7	0.0000	0.0338	NA	0.0000	0.0000	0.0351	NA

Table S 55: Specificity obtained by all the algorithms under the haplotype-based approach with 4-SNP haplotype length, dominant genetic model and holdout sampling. Results for each p-value threshold (column 1) are shown. The maximum p-value threshold used was 0.15.

p-value threshold	BD	CAD	HT	IBD	RA	T1D	T2D
			NBC				
0.15	0.5770	1,0000	1,0000	0.8811	0.5986	0.6757	0.623
0.1	0.5919	1,0000	1,0000	0.8959	0.6095	0.7054	0.655
0.05	0.5973	1,0000	1,0000	0.7541	0.6595	0.7757	0.656
0.01	0.6405	1,0000	1,0000	0.9676	0.7135	0.8486	0.710
0.0010	0.7324	0.9973	0.9986	0.9230	0.7203	0.8878	0.791
1.0e-4	0.7932	0.0311	0.9986	0.9581	0.7311	0.8797	0.878
1.0e-5	0.8824	0.1824	0.0649	1,0000	0.7392	0.8716	0.948
1.0e-6	0.9973	0.0770	NA	1,0000	0.7554	0.8743	0.989
1.0e-7	0.9973	0.1378	NA	1,0000	0.7568	0.8703	NA
			sSVM				
0.15	0.3797	0.0000	0.0000	0.5284	0.3514	0.4541	0.477
0.1	0.4838	0.0000	0.0000	0.4892	0.4392	0.5432	0.564
0.05	0.5041	0.0027	0.0000	0.3986	0.5149	0.6014	0.595
0.01	0.5649	0.9311	0.1365	0.6378	0.5892	0.6946	0.614
0.0010	0.6595	0.6297	0.5905	0.7351	0.6851	0.8068	0.679
1.0e-4	0.6338	0.1392	0.8392	0.7689	0.7405	0.8189	0.637
1.0e-5	0.3378	0.2527	0.1419	0.6797	0.7500	0.8270	0.636
1.0e-6	0.2014	0.2554	NA	0.2608	0.7527	0.8230	0.643
1.0e-7	0.0730	0.0176	NA	0.0000	0.7446	0.8189	NA
			AdaBoostM1				
0.15	0.6932	0.2392	0.8770	0.9095	0.7581	0.8419	0.710
0.1	0.6919	1,0000	0.5554	0.6770	0.7324	0.8378	0.717
0.05	0.6838	0.9595	0.4838	0.8703	0.7459	0.8270	0.714
0.01	0.6770	0.9973	0.3257	0.8784	0.7243	0.7797	0.704
0.0010	0.7554	0.5351	0.4378	0.9986	0.7486	0.8554	0.801
1.0e-4	0.8486	0.0162	0.9297	0.9662	0.8811	0.9041	0.923
1.0e-5	0.9554	0.0378	0.0459	1,0000	0.9230	0.9270	0.973
1.0e-6	0.9973	0.0486	NA	1,0000	0.9581	0.9270	0.989
1.0e-7	0.9973	0.1689	NA	1,0000	0.9676	0.9297	NA
			C4.5				
0.15	0.7865	1,0000	1,0000	1,0000	0.8108	0.7622	0.724
0.1	0.7865	1,0000	1,0000	1,0000	0.7878	0.7595	0.787
0.05	0.7622	1,0000	1,0000	0.9959	0.7838	0.7892	0.758
0.01	0.7703	1,0000	1,0000	0.9689	0.7635	0.8284	0.755
0.0010	0.7486	0.5514	0.5703	0.9811	0.7865	0.8014	0.717
1.0e-4	0.7068	0.1378	0.9095	0.9027	0.7946	0.8108	0.683
1.0e-5	0.9459	0.3068	1,0000	1,0000	0.7541	0.7784	0.985
1.0e-6	1,0000	0.9824	NA	1,0000	0.7676	0.8027	0.994
1.0e-7	1,0000	1,0000	NA	1,0000	0.7230	0.7973	NA
			20RF				
0.15	1,0000	0.9973	1,0000	1,0000	1,0000	1,0000	1,000
0.1	1,0000	1,0000	0.9986	1,0000	1,0000	1,0000	1,000
0.05	1,0000	0.9946	0.9905	1,0000	1,0000	1,0000	1,000
0.01	1,0000	0.9946	0.9959	1,0000	1,0000	1,0000	1,000
0.0010	1,0000	1,0000	1,0000	1,0000	1,0000	0.9932	1,000
1.0e-4	1,0000	0.9932	1,0000	1,0000	0.9946	0.9919	0.998
1.0e-5	1,0000	0.9635	0.1608	0.9946	0.9851	0.9878	0.997
1.0e-6	0.9973	0.6230	NA	1,0000	0.9676	0.9797	0.995
1.0e-7	0.9973	0.9838	NA	1,0000	0.9622	0.9743	NA

Table S 56: Specificity obtained by all the algorithms under the haplotype-based approach with 4-SNP haplotype length, recessive genetic model and holdout sampling. Results for each p-value threshold (column 1) are shown. The maximum p-value threshold used was 0.15.

p-value threshold	BD	CAD	HT	IBD	RA	T1D	T2D
			NBC				
0.15	0.2730	1,0000	1,0000	0.4662	0.3514	0.4608	0.386
0.1	0.3176	1,0000	1,0000	0.2419	0.3919	0.5014	0.406
0.05	0.3378	1,0000	1,0000	0.6500	0.4527	0.5716	0.412
0.01	0.4176	0.9973	1,0000	0.8784	0.5811	0.6865	0.448
0.0010	0.4676	1,0000	1,0000	0.5284	0.6095	0.7270	0.483
1.0e-4	0.4378	0.9959	0.9351	0.3419	0.6189	0.7257	0.447
1.0e-5	0.3000	0.0189	0.0000	0.5297	0.6162	0.7176	0.394
1.0e-6	0.2257	0.0068	NA	0.3473	0.6054	0.7216	0.000
1.0e-7	0.1257	0.0000	NA	0.0000	0.6081	0.7243	NA
			sSVM				
0.15	0.0747	0.0000	0.0000	0.0460	0.0896	0.1645	0.170
0.1	0.1343	0.0000	0.0000	0.0134	0.1460	0.2199	0.237
0.05	0.1719	0.0000	0.0000	0.0769	0.1936	0.2768	0.228
0.01	0.2554	0.3252	0.3504	0.1931	0.3085	0.3962	0.326
0.0010	0.2938	0.9839	0.0148	0.3936	0.3846	0.6842	0.387
1.0e-4	0.2747	0.7229	0.0014	0.1990	0.4864	0.6866	0.321
1.0e-5	0.1022	0.0000	0.0000	0.4462	0.5394	0.7174	0.182
1.0e-6	0.0607	0.0000	NA	0.0658	0.5388	0.7171	0.000
1.0e-7	0.0167	0.0000	NA	0.0000	0.4937	0.7165	NA
			AdaBoostM1				
0.15	0.4297	0.6581	0.7851	0.7959	0.4743	0.6122	0.425
0.1	0.4324	0.3676	0.2608	0.4216	0.4865	0.6311	0.433
0.05	0.4203	0.7568	0.5986	0.6284	0.4595	0.6054	0.413
0.01	0.4378	0.9608	0.8946	0.5622	0.4622	0.5432	0.445
0.0010	0.5054	0.8338	0.9851	0.6473	0.4770	0.6541	0.439
1.0e-4	0.4459	0.2676	0.9770	0.2703	0.5527	0.6554	0.455
1.0e-5	0.2865	0.2311	0.0000	0.3730	0.5527	0.6595	0.397
1.0e-6	0.2257	0.1486	NA	0.3473	0.5500	0.6514	0.000
1.0e-7	0.1257	0.0000	NA	0.0000	0.5243	0.6635	NA
			C4.5				
0.15	0.5439	1,0000	1,0000	1,0000	0.4775	0.6329	0.375
0.1	0.4851	1,0000	1,0000	0.4506	0.5441	0.6703	0.435
0.05	0.5172	1,0000	1,0000	0.8165	0.4709	0.6517	0.451
0.01	0.5409	1,0000	1,0000	0.8563	0.5533	0.5840	0.448
0.0010	0.5705	1,0000	1,0000	0.6952	0.4705	0.5930	0.495
1.0e-4	0.4501	1,0000	0.3218	0.3598	0.5016	0.6435	0.463
1.0e-5	0.2905	0.0122	0.0000	0.2986	0.5047	0.6965	0.425
1.0e-6	0.2257	0.0000	NA	0.3473	0.5265	0.6046	0.000
1.0e-7	0.1257	0.0000	NA	0.0000	0.5028	0.6000	NA
			20RF				
0.15	0.0149	0.0757	0.2176	0.1041	0.0014	0.0135	0.037
0.1	0.0081	0.0649	0.0432	0.0878	0.0203	0.0135	0.041
0.05	0.0149	0.2081	0.0649	0.4068	0.0149	0.0189	0.051
0.01	0.0311	0.4838	0.2203	0.2527	0.0297	0.0662	0.037
0.0010	0.0500	0.8824	0.6662	0.3243	0.2014	0.3473	0.101
1.0e-4	0.1743	0.0054	0.2824	0.2865	0.3622	0.4541	0.193
1.0e-5	0.2257	0.0419	0.0000	0.3730	0.4243	0.4986	0.293
1.0e-6	0.2257	0.0189	NA	0.3473	0.4419	0.5068	0.000
1.0e-7	0.1257	0.0324	NA	0.0000	0.4730	0.5284	NA

Table S 57: Specificity obtained by all the algorithms under the haplotype-based approach with 5-SNP haplotype length, additive genetic model and holdout sampling. Results for each p-value threshold (column 1) are shown. The maximum p-value threshold used was 0.15.

p-value threshold	BD	CAD	HT	IBD	RA	T1D	T2D
			NBC				
0.15	0.1311	1,0000	1,0000	0.2459	0.1892	0.2811	0.202
0.1	0.1500	1,0000	1,0000	0.1014	0.2203	0.3081	0.209
0.05	0.1716	0.9892	1,0000	0.3622	0.2500	0.3878	0.186
0.01	0.2392	0.9568	1,0000	0.6419	0.3824	0.5622	0.205
0.0010	0.2338	0.9973	0.9892	0.2486	0.4635	0.6378	0.214
1.0e-4	0.1649	0.9541	0.0649	0.0541	0.4662	0.6527	0.086
1.0e-5	0.0946	0.0000	0.0000	0.0473	0.4635	0.6500	0.025
1.0e-6	0.0500	0.0000	NA	0.0000	0.4514	0.6338	0.000
1.0e-7	0.0149	0.0000	NA	0.0000	0.4284	0.6311	NA
			sSVM				
0.15	0.0500	0.0000	0.0000	0.0338	0.0608	0.1014	0.102
0.1	0.0784	0.0000	0.0000	0.0108	0.0892	0.1284	0.139
0.05	0.1041	0.0000	0.0000	0.0500	0.1068	0.1568	0.129
0.01	0.1446	0.1622	0.1757	0.1054	0.1676	0.1986	0.175
0.0010	0.1608	0.7432	0.0122	0.2000	0.2027	0.3865	0.200
1.0e-4	0.1541	0.4054	0.0014	0.1108	0.2662	0.4351	0.164
1.0e-5	0.0811	0.0000	0.0000	0.2243	0.3419	0.4838	0.106
1.0e-6	0.0500	0.0000	NA	0.0459	0.3378	0.4865	0.000
1.0e-7	0.0149	0.0000	NA	0.0000	0.3189	0.4986	NA
			AdaBoostM1				
0.15	0.2095	0.3743	0.5108	0.4865	0.2189	0.3716	0.208
0.1	0.1986	0.1676	0.1122	0.1784	0.2270	0.3730	0.205
0.05	0.2257	0.4770	0.3203	0.3622	0.2324	0.3500	0.175
0.01	0.1959	0.7824	0.6311	0.3054	0.2054	0.2770	0.214
0.0010	0.2365	0.5324	0.8851	0.3527	0.2351	0.3878	0.178
1.0e-4	0.1459	0.0608	0.9405	0.0176	0.1581	0.3662	0.068
1.0e-5	0.0811	0.0378	0.0000	0.0014	0.1311	0.3351	0.021
1.0e-6	0.0500	0.0378	NA	0.0000	0.0689	0.3351	0.000
1.0e-7	0.0149	0.0000	NA	0.0000	0.0392	0.3324	NA
			C4.5				
0.15	0.2797	1,0000	1,0000	0.9851	0.2824	0.2973	0.216
0.1	0.2770	1,0000	1,0000	0.2784	0.3000	0.2946	0.225
0.05	0.3189	1,0000	1,0000	0.4959	0.2743	0.3419	0.231
0.01	0.3230	1,0000	1,0000	0.5554	0.3081	0.3297	0.229
0.0010	0.2743	0.9919	0.9973	0.4608	0.2324	0.3473	0.206
1.0e-4	0.2378	0.9946	0.0986	0.1892	0.2730	0.3703	0.204
1.0e-5	0.0824	0.0000	0.0000	0.0000	0.2595	0.3959	0.043
1.0e-6	0.0500	0.0000	NA	0.0000	0.2595	0.3662	0.000
1.0e-7	0.0149	0.0000	NA	0.0000	0.2689	0.3878	NA
			20RF				
0.15	0.0000	0.0000	0.0000	0.0000	0.0000	0.0000	0.000
0.1	0.0000	0.0000	0.0000	0.0000	0.0000	0.0000	0.000
0.05	0.0000	0.0000	0.0000	0.0000	0.0000	0.0000	0.000
0.01	0.0000	0.0000	0.0000	0.0000	0.0000	0.0000	0.000
0.0010	0.0000	0.0027	0.0000	0.0000	0.0000	0.0000	0.000
1.0e-4	0.0000	0.0000	0.0000	0.0000	0.0000	0.0000	0.000
1.0e-5	0.0365	0.0000	0.0000	0.0000	0.0000	0.0068	0.002
1.0e-6	0.0500	0.0000	NA	0.0000	0.0000	0.0216	0.000
1.0e-7	0.0149	0.0000	NA	0.0000	0.0000	0.0324	NA

Table S 58: Specificity obtained by all the algorithms under the haplotype-based approach with 5-SNP haplotype length, dominant genetic model and holdout sampling. Results for each p-value threshold (column 1) are shown. The maximum p-value threshold used was 0.15.

p-value threshold	BD	CAD	HT	IBD	RA	T1D	T2D
				NBC			
0.15	0.5730	1,0000	1,0000	0.7486	0.6149	0.7068	0.628
0.1	0.5959	1,0000	1,0000	0.5108	0.6297	0.7311	0.645
0.05	0.6068	1,0000	1,0000	0.8378	0.6811	0.7851	0.659
0.01	0.6459	1,0000	1,0000	0.9568	0.7311	0.8514	0.704
0.0010	0.7270	1,0000	1,0000	0.7905	0.7392	0.8838	0.763
1.0e-4	0.7919	1,0000	0.9986	0.7757	0.7405	0.8865	0.863
1.0e-5	0.9568	0.6108	1,0000	0.9986	0.7473	0.8797	0.967
1.0e-6	0.9959	0.6378	NA	0.9973	0.7500	0.8824	1,000
1.0e-7	0.9959	1,0000	NA	0.9973	0.7622	0.8743	NA
				sSVM			
0.15	0.3811	0.0000	0.0000	0.3000	0.3824	0.4851	0.500
0.1	0.4946	0.0000	0.0000	0.2027	0.4784	0.5446	0.554
0.05	0.4986	0.0000	0.0000	0.4000	0.5554	0.5905	0.560
0.01	0.5784	0.6635	0.6743	0.5595	0.6243	0.6973	0.637
0.0010	0.6135	0.9878	0.1892	0.6919	0.6757	0.8216	0.683
1.0e-4	0.5932	0.8446	0.0635	0.5541	0.7189	0.8014	0.651
1.0e-5	0.2878	0.0000	0.0000	0.7216	0.7081	0.8095	0.523
1.0e-6	0.2257	0.0000	NA	0.3473	0.7108	0.8081	0.000
1.0e-7	0.1257	0.0000	NA	0.0000	0.6730	0.8027	NA
				AdaBoostM1			
0.15	0.7000	0.8324	0.9149	0.9149	0.7095	0.8203	0.706
0.1	0.7081	0.6365	0.5297	0.6797	0.7365	0.8257	0.705
0.05	0.6797	0.8824	0.8351	0.8338	0.7068	0.7878	0.681
0.01	0.6730	0.9851	0.9676	0.7892	0.7257	0.7784	0.735
0.0010	0.7838	0.9541	0.9986	0.8541	0.7595	0.8419	0.751
1.0e-4	0.8541	0.4743	0.9973	0.7784	0.8757	0.8797	0.895
1.0e-5	0.9608	0.2514	0.0014	0.9986	0.9122	0.9000	0.974
1.0e-6	0.9959	0.2500	NA	0.9973	0.9365	0.9176	0.993
1.0e-7	0.9959	1,0000	NA	0.9973	0.9486	0.9108	NA
				C4.5			
0.15	0.7541	1,0000	1,0000	1,0000	0.7743	0.7797	0.709
0.1	0.7270	1,0000	1,0000	0.8068	0.8108	0.7838	0.723
0.05	0.7689	1,0000	1,0000	0.9108	0.7838	0.8027	0.733
0.01	0.8068	1,0000	1,0000	0.9405	0.8081	0.7932	0.728
0.0010	0.7446	1,0000	1,0000	0.8959	0.7189	0.8014	0.717
1.0e-4	0.7405	1,0000	0.6216	0.6851	0.7432	0.8068	0.700
1.0e-5	0.9973	0.1851	1,0000	0.9986	0.7527	0.8527	0.956
1.0e-6	1,0000	1,0000	NA	0.9973	0.7500	0.7973	1,000
1.0e-7	1,0000	1,0000	NA	1,0000	0.7662	0.8189	NA
				20RF			
0.15	1,0000	0.9973	0.9973	1,0000	1,0000	1,0000	1,000
0.1	1,0000	0.9919	0.9959	1,0000	1,0000	1,0000	1,000
0.05	1,0000	0.9986	0.9905	1,0000	1,0000	1,0000	1,000
0.01	1,0000	1,0000	1,0000	1,0000	1,0000	1,0000	1,000
0.0010	1,0000	1,0000	1,0000	1,0000	1,0000	0.9973	1,000
1.0e-4	1,0000	0.8797	1,0000	1,0000	0.9797	0.9905	1,000
1.0e-5	0.9973	1,0000	0.2419	0.9959	0.9770	0.9811	0.994
1.0e-6	0.9959	0.8405	NA	0.9973	0.9581	0.9730	1,000
1.0e-7	0.9959	0.9851	NA	0.9973	0.9595	0.9716	NA

Table S 59: Specificity obtained by all the algorithms under the haplotype-based approach with 5-SNP haplotype length, recessive genetic model and holdout sampling. Results for each p-value threshold (column 1) are shown. The maximum p-value threshold used was 0.15.

p-value threshold	BD	CAD	HT	IBD	RA	T1D	T2D
				NBC			
0.15	0.5653	0.1888	0.1860	0.5617	0.5860	0.6080	0.546
0.1	0.5620	0.1888	0.1860	0.5468	0.5939	0.6266	0.549
0.05	0.5578	0.1888	0.1860	0.5459	0.6008	0.6395	0.534
0.01	0.5569	0.1888	0.1860	0.5715	0.6283	0.6544	0.525
0.0010	0.5241	0.1888	0.1860	0.5208	0.6384	0.6636	0.509
1.0e-4	0.5284	0.1888	0.1860	0.5266	0.6460	0.6615	0.539
1.0e-5	0.5185	0.5038	0.1860	0.5311	0.6530	0.6776	0.560
1.0e-6	NA	0.5345	NA	NA	0.6326	0.6801	0.536
1.0e-7	NA	0.5036	NA	NA	0.6234	0.6922	NA
				sSVM			
0.15	0.6585	0.3198	0.3235	0.5866	0.5847	0.6976	0.570
0.1	0.6215	0.3198	0.3235	0.5687	0.6004	0.6728	0.566
0.05	0.6049	0.3198	0.3235	0.5459	0.5884	0.6673	0.545
0.01	0.5815	0.5619	0.5933	0.5604	0.6109	0.7069	0.533
0.0010	0.5360	0.5347	0.5485	0.5317	0.6553	0.7944	0.539
1.0e-4	0.5157	0.5137	0.5030	0.5157	0.7184	0.8281	0.564
1.0e-5	0.5239	0.5539	0.1817	0.5488	0.7199	0.8180	0.565
1.0e-6	NA	0.5949	NA	NA	0.7179	0.8199	0.529
1.0e-7	NA	0.3303	NA	NA	0.7100	0.8218	NA
				AdaBoostM1			
0.15	0.5362	0.5463	0.5278	0.5137	0.6254	0.7121	0.528
0.1	0.5475	0.5291	0.5105	0.5610	0.6234	0.7039	0.536
0.05	0.5423	0.5068	0.5174	0.5298	0.6182	0.7074	0.523
0.01	0.5507	0.5066	0.4927	0.5420	0.5981	0.6684	0.523
0.0010	0.5278	0.5188	0.5140	0.5234	0.6488	0.6902	0.521
1.0e-4	0.5431	0.4971	0.4791	0.5304	0.6829	0.7578	0.553
1.0e-5	0.5185	0.5032	0.1860	0.5468	0.6997	0.7538	0.559
1.0e-6	NA	0.5155	NA	NA	0.6827	0.7540	0.541
1.0e-7	NA	0.5036	NA	NA	0.6716	0.7618	NA
				C4.5			
0.15	0.5172	0.5157	0.7554	0.5089	0.5453	0.5522	0.496
0.1	0.5151	0.5620	0.5079	0.4646	0.5961	0.5714	0.532
0.05	0.5103	0.6471	0.5664	0.5138	0.5708	0.5834	0.527
0.01	0.5370	0.5218	0.4940	0.5347	0.6200	0.6037	0.518
0.0010	0.5507	0.5121	0.5147	0.5138	0.6009	0.6202	0.538
1.0e-4	0.5293	0.5010	0.1860	0.5169	0.6485	0.6642	0.544
1.0e-5	0.5393	0.4888	0.1860	0.5354	0.6516	0.6574	0.528
1.0e-6	NA	0.6369	NA	NA	0.6279	0.6673	0.548
1.0e-7	NA	0.5502	NA	NA	0.6231	0.6835	NA
				20RF			
0.15	0.3839	0.4958	0.5137	0.5206	0.5000	0.5019	0.589
0.1	0.5681	0.5398	0.5018	0.5046	0.5320	0.6695	0.571
0.05	0.5458	0.5209	0.5166	0.4789	0.4946	0.5569	0.533
0.01	0.4668	0.4810	0.4876	0.4883	0.6149	0.6001	0.578
0.0010	0.4286	0.5248	0.4985	0.5016	0.6060	0.6708	0.556
1.0e-4	0.5419	0.4959	0.4820	0.5101	0.6595	0.7393	0.530
1.0e-5	0.5167	0.4902	0.1860	0.5477	0.6717	0.7210	0.554
1.0e-6	NA	0.3198	NA	NA	0.6615	0.7207	0.536
1.0e-7	NA	0.5944	NA	NA	0.6865	0.7326	NA

Table S60: Precision obtained by all the algorithms under the haplotype-based approach with 1-SNP haplotype length, additive genetic model and holdout sampling. Results for each p-value threshold (column 1) are shown. The maximum p-value threshold used was 0.15.

p-value threshold	BD	CAD	HT	IBD	RA	T1D	T2D
				NBC			
0.15	0.5742	0.1888	0.1860	0.5354	0.5856	0.6223	0.538
0.1	0.5720	0.1888	0.1860	0.5142	0.5904	0.6236	0.536
0.05	0.5872	0.1888	0.1860	0.5167	0.5845	0.6418	0.538
0.01	0.5632	0.1888	0.1860	0.5330	0.6061	0.6585	0.524
0.0010	0.5389	0.1888	0.1860	0.5327	0.6364	0.6672	0.512
1.0e-4	0.5254	0.5035	0.4700	0.5282	0.6489	0.6695	0.539
1.0e-5	0.3112	0.4794	0.4457	0.5703	0.6374	0.6794	0.669
1.0e-6	NA	0.3198	NA	NA	0.6273	0.6892	0.319
1.0e-7	NA	0.3198	NA	NA	0.6276	0.7001	NA
				sSVM			
0.15	0.6504	0.3198	0.3235	0.5753	0.5768	0.6829	0.559
0.1	0.6015	0.3198	0.3235	0.5363	0.5823	0.6525	0.550
0.05	0.5871	0.3198	0.3235	0.5208	0.5707	0.6447	0.534
0.01	0.5659	0.5615	0.5935	0.5268	0.5856	0.6548	0.531
0.0010	0.5370	0.5150	0.5176	0.5154	0.6015	0.7250	0.514
1.0e-4	0.4986	0.5247	0.5084	0.4974	0.6448	0.7511	0.548
1.0e-5	0.5172	0.5502	0.4457	0.5365	0.6654	0.7510	0.530
1.0e-6	NA	0.5864	NA	NA	0.6682	0.7499	0.506
1.0e-7	NA	0.3196	NA	NA	0.6655	0.7460	NA
				AdaBoostM1			
0.15	0.5270	0.5232	0.5137	0.5085	0.6344	0.6930	0.524
0.1	0.5278	0.5264	0.4926	0.5296	0.6095	0.6968	0.509
0.05	0.5520	0.5048	0.4893	0.5377	0.6100	0.7004	0.525
0.01	0.5432	0.4967	0.5202	0.5301	0.5926	0.6478	0.511
0.0010	0.5262	0.5238	0.5094	0.4960	0.6508	0.6961	0.512
1.0e-4	0.5053	0.5278	0.5110	0.5206	0.6818	0.7456	0.557
1.0e-5	0.3112	0.3195	0.4457	0.2932	0.7138	0.7588	0.609
1.0e-6	NA	0.3198	NA	NA	0.6480	0.7654	0.319
1.0e-7	NA	0.3198	NA	NA	0.6279	0.7641	NA
				C4.5			
0.15	0.5034	0.5164	0.7549	0.4972	0.5303	0.5798	0.528
0.1	0.5353	0.5593	0.5158	0.4813	0.5749	0.5736	0.506
0.05	0.5319	0.6461	0.5445	0.4986	0.5836	0.6071	0.523
0.01	0.5441	0.5173	0.5022	0.5341	0.6012	0.5913	0.526
0.0010	0.5138	0.5365	0.4849	0.5030	0.5748	0.6405	0.518
1.0e-4	0.5426	0.5157	0.4531	0.5119	0.6232	0.6672	0.521
1.0e-5	0.3113	0.4722	0.4457	0.2932	0.6665	0.6928	0.319
1.0e-6	NA	0.3198	NA	NA	0.6082	0.6898	0.319
1.0e-7	NA	0.3198	NA	NA	0.5943	0.7199	NA
				20RF			
0.15	0.3113	0.3198	0.3235	0.2932	0.3101	0.3249	0.319
0.1	0.3113	0.3198	0.3235	0.2932	0.3101	0.3249	0.319
0.05	0.3113	0.3198	0.3235	0.2932	0.3101	0.3249	0.319
0.01	0.3113	0.3198	0.3235	0.2932	0.3101	0.3249	0.319
0.0010	0.3113	0.3198	0.3235	0.2932	0.3101	0.3249	0.319
1.0e-4	0.3113	0.3198	0.3235	0.2932	0.3101	0.7557	0.754
1.0e-5	0.3112	0.4059	0.4457	0.2932	0.3101	0.7574	0.630
1.0e-6	NA	0.3198	NA	NA	0.3101	0.7595	0.319
1.0e-7	NA	0.3198	NA	NA	0.3101	0.7525	NA

Table S61: Precision obtained by all the algorithms under the haplotype-based approach with 1-SNP haplotype length, dominant genetic model and holdout sampling. Results for each p-value threshold (column 1) are shown. The maximum p-value threshold used was 0.15.

p-value threshold	BD	CAD	HT	IBD	RA	T1D	T2D
				NBC			
0.15	0.5855	0.1888	0.1860	0.5730	0.5750	0.5937	0.536
0.1	0.5763	0.1888	0.1860	0.5383	0.5823	0.5891	0.541
0.05	0.5443	0.1888	0.1860	0.5387	0.5954	0.5955	0.543
0.01	0.5473	0.1888	0.1860	0.7522	0.6093	0.6341	0.527
0.0010	0.5081	0.1888	0.1860	0.5094	0.6428	0.6712	0.539
1.0e-4	0.5261	0.1888	0.1860	0.5336	0.6500	0.6927	0.520
1.0e-5	0.1954	0.1888	0.1860	0.2102	0.6423	0.7018	0.545
1.0e-6	NA	0.1888	NA	NA	0.6465	0.6963	0.189
1.0e-7	NA	0.1888	NA	NA	0.6337	0.7028	NA
				sSVM			
0.15	0.5739	0.3198	0.3235	0.5369	0.5531	0.5899	0.538
0.1	0.5742	0.3198	0.3235	0.5564	0.5624	0.5876	0.544
0.05	0.5575	0.3198	0.3235	0.5407	0.5526	0.5862	0.530
0.01	0.5440	0.5121	0.5110	0.5572	0.5654	0.6402	0.516
0.0010	0.5145	0.5335	0.5468	0.5300	0.6155	0.7133	0.540
1.0e-4	0.5291	0.4975	0.5023	0.5254	0.6766	0.7538	0.543
1.0e-5	0.5228	0.5240	0.1860	0.5311	0.6711	0.7514	0.556
1.0e-6	NA	0.5726	NA	NA	0.6628	0.7545	0.548
1.0e-7	NA	0.5931	NA	NA	0.6540	0.7543	NA
				AdaBoostM1			
0.15	0.5476	0.5331	0.5178	0.5519	0.6309	0.6914	0.524
0.1	0.5521	0.5370	0.5040	0.5287	0.6280	0.6872	0.534
0.05	0.5452	0.5210	0.5121	0.5173	0.6102	0.6667	0.530
0.01	0.5422	0.4883	0.5153	0.5425	0.6097	0.6645	0.533
0.0010	0.5238	0.5189	0.5110	0.5172	0.6539	0.6794	0.545
1.0e-4	0.5256	0.5106	0.1860	0.5418	0.7371	0.7343	0.540
1.0e-5	0.1954	0.4659	0.1860	0.7519	0.7489	0.7438	0.567
1.0e-6	NA	0.1888	NA	NA	0.7533	0.7565	0.189
1.0e-7	NA	0.1888	NA	NA	0.7523	0.7570	NA
				C4.5			
0.15	0.5196	0.4936	0.5286	0.5150	0.5701	0.5634	0.504
0.1	0.5200	0.5209	0.4989	0.4714	0.5927	0.5649	0.541
0.05	0.5357	0.5346	0.5653	0.5178	0.5705	0.6109	0.513
0.01	0.5440	0.5307	0.4962	0.5180	0.6205	0.6122	0.508
0.0010	0.5596	0.5166	0.5108	0.4983	0.6110	0.6401	0.525
1.0e-4	0.5271	0.4965	0.1860	0.5254	0.6534	0.6840	0.538
1.0e-5	0.1954	0.4370	0.1860	0.2102	0.6714	0.7023	0.471
1.0e-6	NA	0.5217	NA	NA	0.6541	0.6963	0.189
1.0e-7	NA	0.1888	NA	NA	0.6570	0.7102	NA
				20RF			
0.15	0.1954	0.1888	0.1860	0.2102	0.1963	0.1849	0.189
0.1	0.1954	0.1887	0.3278	0.2102	0.1963	0.1849	0.189
0.05	0.1954	0.7544	0.1860	0.2102	0.1963	0.1849	0.189
0.01	0.1954	0.7544	0.1860	0.2102	0.1963	0.1849	0.189
0.0010	0.1954	0.4715	0.7548	0.2097	0.7534	0.6287	0.189
1.0e-4	0.4576	0.5930	0.1860	0.2102	0.7410	0.6966	0.512
1.0e-5	0.4911	0.4753	0.1860	0.2102	0.7169	0.7065	0.555
1.0e-6	NA	0.5044	NA	NA	0.7252	0.6949	0.189
1.0e-7	NA	0.1888	NA	NA	0.7245	0.7186	NA

Table S62: Precision obtained by all the algorithms under the haplotype-based approach with 1-SNP haplotype length, recessive genetic model and holdout sampling. Results for each p-value threshold (column 1) are shown. The maximum p-value threshold used was 0.15.

p-value threshold	BD	CAD	HT	IBD	RA	T1D	T2D
				NBC			
0.15	0.6092	0.1888	0.1860	0.5591	0.6190	0.6454	0.567
0.1	0.6104	0.1888	0.1860	0.5774	0.6280	0.6524	0.582
0.05	0.6104	0.1888	0.1860	0.5926	0.6332	0.6666	0.581
0.01	0.6118	0.1888	0.1860	0.7519	0.6539	0.6821	0.568
0.0010	0.6079	0.1888	0.1860	0.5427	0.6596	0.6762	0.576
1.0e-4	0.6106	0.4148	0.5368	0.5651	0.6597	0.6806	0.596
1.0e-5	0.6229	0.4994	0.6631	0.5919	0.6567	0.6933	0.542
1.0e-6	0.6466	0.4915	0.6698	0.6716	0.6442	0.6927	0.515
1.0e-7	0.6463	0.5491	NA	0.2932	0.6354	0.6937	NA
				sSVM			
0.15	0.6049	0.3198	0.3235	0.5495	0.6123	0.6875	0.596
0.1	0.6229	0.3198	0.3235	0.5707	0.5901	0.6800	0.572
0.05	0.5887	0.3198	0.3235	0.5453	0.5829	0.6731	0.577
0.01	0.5785	0.5414	0.5039	0.5706	0.6199	0.7145	0.540
0.0010	0.5771	0.5347	0.7559	0.5620	0.6757	0.7871	0.553
1.0e-4	0.6141	0.3222	0.5223	0.5376	0.7288	0.8361	0.604
1.0e-5	0.7169	0.5436	0.3457	0.5952	0.7252	0.8389	0.581
1.0e-6	0.7296	0.4384	0.3457	0.5290	0.7259	0.8368	0.330
1.0e-7	0.7598	0.5436	NA	0.2932	0.7166	0.8297	NA
				AdaBoostM1			
0.15	0.5919	0.5179	0.5086	0.5773	0.6424	0.7248	0.566
0.1	0.6081	0.4962	0.4808	0.5355	0.6536	0.7120	0.543
0.05	0.6002	0.5036	0.5262	0.5496	0.6492	0.7189	0.549
0.01	0.6000	0.3768	0.5180	0.5509	0.6237	0.6996	0.566
0.0010	0.6220	0.3014	0.4703	0.5642	0.6757	0.7314	0.587
1.0e-4	0.6105	0.4823	0.5147	0.5767	0.7061	0.7761	0.600
1.0e-5	0.6107	0.1888	0.6673	0.6015	0.7175	0.7719	0.563
1.0e-6	0.6479	0.5040	0.6698	0.6797	0.7034	0.7701	0.688
1.0e-7	0.6463	0.5491	NA	0.2932	0.6863	0.7754	NA
				C4.5			
0.15	0.5854	0.1888	0.1860	0.2270	0.6129	0.5816	0.587
0.1	0.6149	0.1888	0.1860	0.2270	0.6219	0.6415	0.611
0.05	0.6195	0.1888	0.1860	0.2268	0.5969	0.6364	0.592
0.01	0.6161	0.1888	0.1860	0.5521	0.6360	0.6436	0.568
0.0010	0.6225	0.5014	0.4822	0.5090	0.6687	0.6633	0.561
1.0e-4	0.6022	0.1888	0.5271	0.5542	0.6310	0.6628	0.588
1.0e-5	0.6162	0.4688	0.6698	0.5980	0.6628	0.6786	0.548
1.0e-6	0.6429	0.5359	0.6698	0.6659	0.6507	0.6489	0.699
1.0e-7	0.6428	0.4131	NA	0.2932	0.6439	0.6846	NA
				20RF			
0.15	0.5007	0.5093	0.5108	0.5129	0.5959	0.4810	0.486
0.1	0.5532	0.4861	0.5203	0.5008	0.5170	0.5405	0.501
0.05	0.5327	0.5325	0.5089	0.4948	0.5319	0.6701	0.520
0.01	0.4973	0.5040	0.5229	0.5070	0.6039	0.5553	0.551
0.0010	0.6316	0.4937	0.5088	0.5641	0.6472	0.6823	0.565
1.0e-4	0.6315	0.5019	0.5361	0.5621	0.6542	0.7467	0.600
1.0e-5	0.6618	0.4191	0.6705	0.5954	0.6766	0.7259	0.557
1.0e-6	0.6479	0.4337	0.6698	0.6797	0.6756	0.7386	0.688
1.0e-7	0.6463	0.4131	NA	0.2932	0.6801	0.7540	NA

Table S63: Precision obtained by all the algorithms under the haplotype-based approach with 2-SNP haplotype length, additive genetic model and holdout sampling. Results for each p-value threshold (column 1) are shown. The maximum p-value threshold used was 0.15.

p-value threshold	BD	CAD	HT	IBD	RA	T1D	T2D
				NBC			
0.15	0.6117	0.1888	0.1860	0.5320	0.6178	0.6476	0.587
0.1	0.6287	0.1888	0.1860	0.5322	0.6467	0.6525	0.591
0.05	0.6242	0.1888	0.1860	0.5280	0.6433	0.6525	0.580
0.01	0.6200	0.1888	0.1860	0.5513	0.6495	0.6813	0.570
0.0010	0.6300	0.1888	0.1860	0.5399	0.6641	0.6883	0.593
1.0e-4	0.6357	0.4949	0.5283	0.5721	0.6606	0.6901	0.621
1.0e-5	0.7049	0.5161	0.3235	0.6392	0.6473	0.6894	0.656
1.0e-6	0.7264	0.5422	0.3235	0.7519	0.6402	0.6875	0.646
1.0e-7	0.7547	0.3198	NA	0.2932	0.6422	0.6913	NA
				sSVM			
0.15	0.5864	0.3198	0.3235	0.5389	0.6017	0.6712	0.588
0.1	0.6052	0.3198	0.3235	0.5351	0.5712	0.6582	0.554
0.05	0.5718	0.3198	0.3235	0.5162	0.5680	0.6440	0.563
0.01	0.5541	0.5375	0.5005	0.4939	0.5940	0.6564	0.534
0.0010	0.5498	0.5256	0.5094	0.5487	0.6290	0.7061	0.541
1.0e-4	0.5945	0.3198	0.5188	0.5221	0.6571	0.7550	0.579
1.0e-5	0.7053	0.4479	0.3235	0.5683	0.6672	0.7698	0.563
1.0e-6	0.7196	0.5253	0.3235	0.5225	0.6626	0.7645	0.319
1.0e-7	0.7547	0.4479	NA	0.2932	0.6653	0.7597	NA
				AdaBoostM1			
0.15	0.6086	0.5355	0.4982	0.5697	0.6374	0.7139	0.569
0.1	0.6127	0.5059	0.4739	0.5350	0.6337	0.6962	0.558
0.05	0.6006	0.5181	0.4962	0.5321	0.6294	0.6893	0.537
0.01	0.5813	0.4986	0.5097	0.5311	0.6283	0.6852	0.559
0.0010	0.6312	0.4596	0.4610	0.5585	0.6803	0.7140	0.593
1.0e-4	0.6307	0.4951	0.5661	0.6214	0.6985	0.7706	0.672
1.0e-5	0.7118	0.2144	0.3235	0.7154	0.7193	0.7862	0.757
1.0e-6	0.7327	0.5480	0.3235	0.7519	0.6647	0.7842	0.319
1.0e-7	0.7547	0.3198	NA	0.2932	0.6614	0.7740	NA
				C4.5			
0.15	0.5980	0.1888	0.1860	0.7433	0.6279	0.6004	0.598
0.1	0.5971	0.1888	0.1860	0.7433	0.6417	0.6514	0.595
0.05	0.6286	0.1888	0.1860	0.7117	0.6411	0.6530	0.580
0.01	0.6067	0.1888	0.1860	0.5482	0.6384	0.6562	0.567
0.0010	0.6163	0.5122	0.5166	0.4996	0.6522	0.7065	0.565
1.0e-4	0.6165	0.1888	0.5083	0.5638	0.6549	0.7015	0.570
1.0e-5	0.7024	0.6588	0.3235	0.6784	0.6427	0.7160	0.737
1.0e-6	0.7264	0.3195	0.3235	0.5225	0.6402	0.6970	0.319
1.0e-7	0.7547	0.3198	NA	0.2932	0.6238	0.7053	NA
				20RF			
0.15	0.3113	0.3198	0.3235	0.2932	0.3101	0.3249	0.319
0.1	0.3113	0.3196	0.3235	0.2932	0.3101	0.3249	0.319
0.05	0.3113	0.3198	0.3235	0.2932	0.3101	0.3249	0.319
0.01	0.3113	0.3198	0.3235	0.2932	0.3101	0.3249	0.319
0.0010	0.3113	0.3198	0.3235	0.2932	0.3101	0.3249	0.319
1.0e-4	0.7539	0.3198	0.3235	0.2932	0.3101	0.3249	0.319
1.0e-5	0.7250	0.3198	0.7549	0.6922	0.3101	0.7566	0.755
1.0e-6	0.7312	0.7547	0.3235	0.7519	0.3100	0.7599	0.319
1.0e-7	0.7547	0.6566	NA	0.2932	0.3101	0.7345	NA

Table S64: Precision obtained by all the algorithms under the haplotype-based approach with 2-SNP haplotype length, dominant genetic model and holdout sampling. Results for each p-value threshold (column 1) are shown. The maximum p-value threshold used was 0.15.

p-value threshold	BD	CAD	HT	IBD	RA	T1D	T2D
				NBC			
0.15	0.6132	0.1888	0.1860	0.5688	0.6017	0.6203	0.573
0.1	0.6000	0.1888	0.1860	0.5780	0.6118	0.6249	0.580
0.05	0.5989	0.1888	0.1860	0.6241	0.6237	0.6380	0.585
0.01	0.5987	0.1888	0.1860	0.7519	0.6433	0.6857	0.579
0.0010	0.5996	0.1888	0.1860	0.5460	0.6680	0.6968	0.573
1.0e-4	0.5942	0.1888	0.1860	0.5613	0.6616	0.7037	0.589
1.0e-5	0.6550	0.5661	0.5654	0.7470	0.6547	0.7110	0.552
1.0e-6	0.7574	0.1888	0.1860	0.7511	0.6457	0.7081	0.189
1.0e-7	0.7576	0.1888	NA	0.7433	0.6444	0.7044	NA
				sSVM			
0.15	0.5819	0.3198	0.3235	0.5376	0.5578	0.5947	0.536
0.1	0.5661	0.3198	0.3235	0.5635	0.5614	0.5863	0.548
0.05	0.5537	0.3198	0.3235	0.5422	0.5476	0.5929	0.543
0.01	0.5550	0.5232	0.5139	0.5722	0.5694	0.6436	0.524
0.0010	0.5572	0.5262	0.7555	0.5356	0.6166	0.7132	0.534
1.0e-4	0.5606	0.5230	0.5197	0.5303	0.6740	0.7604	0.563
1.0e-5	0.6210	0.5491	0.6698	0.5611	0.6789	0.7626	0.548
1.0e-6	0.6429	0.4331	0.6698	0.6659	0.6809	0.7624	0.699
1.0e-7	0.6428	0.5491	NA	0.2932	0.6662	0.7567	NA
				AdaBoostM1			
0.15	0.5827	0.5285	0.5028	0.5469	0.6450	0.7087	0.576
0.1	0.5800	0.5011	0.4878	0.5652	0.6527	0.7046	0.565
0.05	0.5794	0.5475	0.5043	0.5384	0.6370	0.7018	0.561
0.01	0.5890	0.4715	0.5454	0.5582	0.6274	0.6813	0.567
0.0010	0.6158	0.1888	0.1860	0.5572	0.6833	0.7350	0.599
1.0e-4	0.6200	0.4715	0.5452	0.5741	0.7397	0.7712	0.620
1.0e-5	0.6780	0.1888	0.5277	0.7417	0.7556	0.7592	0.632
1.0e-6	0.7574	0.4506	0.1860	0.7514	0.7450	0.7748	0.613
1.0e-7	0.7576	0.1888	NA	0.7433	0.7522	0.7650	NA
				C4.5			
0.15	0.5826	0.1888	0.1860	0.2102	0.6191	0.6091	0.612
0.1	0.5871	0.1888	0.1860	0.2102	0.6153	0.6232	0.609
0.05	0.6191	0.1888	0.1860	0.2102	0.6156	0.6386	0.610
0.01	0.6202	0.1888	0.1860	0.5258	0.6521	0.6627	0.581
0.0010	0.6089	0.5028	0.4814	0.5241	0.6600	0.7094	0.582
1.0e-4	0.5890	0.1888	0.5335	0.5618	0.6557	0.6883	0.574
1.0e-5	0.7048	0.4731	0.1860	0.7417	0.6942	0.7138	0.755
1.0e-6	0.1954	0.5211	0.1860	0.2102	0.6732	0.6926	0.189
1.0e-7	0.1954	0.1888	NA	0.2102	0.6858	0.7063	NA
				20RF			
0.15	0.1954	0.4262	0.5721	0.2102	0.1963	0.1849	0.189
0.1	0.1954	0.1888	0.7548	0.2102	0.1963	0.1849	0.189
0.05	0.1954	0.4713	0.4703	0.2102	0.1963	0.1849	0.189
0.01	0.1954	0.1888	0.4398	0.2102	0.1963	0.1849	0.189
0.0010	0.1954	0.1888	0.1860	0.3905	0.1963	0.5878	0.189
1.0e-4	0.1954	0.1888	0.1860	0.7519	0.7342	0.6652	0.754
1.0e-5	0.7575	0.6005	0.5498	0.7412	0.7341	0.6963	0.678
1.0e-6	0.7572	0.1888	0.1860	0.7514	0.7275	0.7152	0.613
1.0e-7	0.7576	0.1888	NA	0.7433	0.7188	0.7293	NA

Table S65: Precision obtained by all the algorithms under the haplotype-based approach with 2-SNP haplotype length, recessive genetic model and holdout sampling. Results for each p-value threshold (column 1) are shown. The maximum p-value threshold used was 0.15.

p-value threshold	BD	CAD	HT	IBD	RA	T1D	T2D
NBC							
0.15	0.6038	0.1888	0.1860	0.5649	0.6213	0.6558	0.564
0.1	0.6058	0.1888	0.1860	0.5422	0.6296	0.6558	0.566
0.05	0.6153	0.1888	0.1860	0.5228	0.6339	0.6697	0.566
0.01	0.6097	0.1888	0.1860	0.5447	0.6568	0.6834	0.569
0.0010	0.6226	0.1888	0.5330	0.5292	0.6623	0.6799	0.582
1.0e-4	0.6191	0.4729	0.1860	0.5650	0.6532	0.6847	0.578
1.0e-5	0.6261	0.5388	0.3404	0.5867	0.6495	0.6886	0.575
1.0e-6	0.6379	0.6693	0.3235	0.5443	0.6408	0.6925	0.536
1.0e-7	0.6456	0.6673	NA	0.2932	0.6276	0.7005	NA
sSVM							
0.15	0.6047	0.3198	0.3235	0.5513	0.6063	0.6914	0.542
0.1	0.6075	0.3198	0.3235	0.5422	0.6225	0.6861	0.564
0.05	0.6012	0.3198	0.3235	0.5428	0.6275	0.6740	0.558
0.01	0.5768	0.5391	0.4988	0.5422	0.6236	0.6905	0.523
0.0010	0.5963	0.5476	0.5112	0.5226	0.6745	0.7841	0.568
1.0e-4	0.6391	0.7558	0.4586	0.5898	0.7102	0.8365	0.593
1.0e-5	0.7218	0.4731	0.1707	0.6278	0.7145	0.8417	0.629
1.0e-6	0.7091	0.4818	0.3388	0.5998	0.7159	0.8338	0.516
1.0e-7	0.7076	0.3198	NA	0.2932	0.7194	0.8292	NA
AdaBoostM1							
0.15	0.5986	0.4916	0.4929	0.5688	0.6490	0.7104	0.545
0.1	0.6041	0.4943	0.5050	0.5342	0.6344	0.7116	0.534
0.05	0.6035	0.4855	0.4948	0.5545	0.6476	0.7068	0.550
0.01	0.5916	0.5174	0.4195	0.5514	0.6123	0.6690	0.534
0.0010	0.6266	0.5615	0.5161	0.5842	0.6488	0.7302	0.572
1.0e-4	0.6188	0.1888	0.1860	0.5716	0.6942	0.7754	0.582
1.0e-5	0.6237	0.5271	0.3540	0.5942	0.6973	0.7772	0.600
1.0e-6	0.6427	0.5431	0.3235	0.5451	0.6914	0.7818	0.536
1.0e-7	0.6456	0.6673	NA	0.2932	0.6754	0.7736	NA
C4.5							
0.15	0.6008	0.1888	0.1860	0.2270	0.6204	0.6232	0.566
0.1	0.6308	0.1888	0.1860	0.2270	0.6373	0.6200	0.557
0.05	0.6332	0.1888	0.1860	0.5703	0.6360	0.6411	0.573
0.01	0.6066	0.1888	0.1860	0.5355	0.6382	0.6076	0.538
0.0010	0.6082	0.5209	0.5073	0.5432	0.6504	0.6523	0.573
1.0e-4	0.5786	0.1885	0.5090	0.5518	0.6472	0.6878	0.568
1.0e-5	0.6225	0.4862	0.1857	0.5880	0.6564	0.6902	0.589
1.0e-6	0.6380	0.5042	0.3235	0.5451	0.6596	0.6845	0.527
1.0e-7	0.6418	0.3198	NA	0.2932	0.6468	0.6864	NA
20RF							
0.15	0.4767	0.5104	0.5233	0.5247	0.4132	0.5300	0.519
0.1	0.5007	0.4923	0.5074	0.4786	0.5522	0.5403	0.517
0.05	0.5155	0.5121	0.5047	0.5298	0.5320	0.4319	0.542
0.01	0.5862	0.5142	0.4598	0.5038	0.5323	0.6810	0.588
0.0010	0.6695	0.5193	0.5208	0.5283	0.6213	0.7088	0.591
1.0e-4	0.6448	0.4797	0.4966	0.5815	0.6535	0.7395	0.621
1.0e-5	0.6497	0.4761	0.3981	0.5847	0.6787	0.7367	0.583
1.0e-6	0.6415	0.4627	0.6699	0.5443	0.6748	0.7322	0.536
1.0e-7	0.6456	0.6932	NA	0.2932	0.6754	0.7391	NA

Table S66: Precision obtained by all the algorithms under the haplotype-based approach with 3-SNP haplotype length, additive genetic model and holdout sampling. Results for each p-value threshold (column 1) are shown. The maximum p-value threshold used was 0.15.

p-value threshold	BD	CAD	HT	IBD	RA	T1D	T2D
				NBC			
0.15	0.6127	0.1888	0.1860	0.5163	0.6248	0.6612	0.587
0.1	0.6340	0.1888	0.1860	0.5265	0.6359	0.6690	0.580
0.05	0.6339	0.1888	0.1860	0.5261	0.6488	0.6759	0.574
0.01	0.6183	0.1888	0.1860	0.5368	0.6521	0.6828	0.572
0.0010	0.6352	0.7546	0.5115	0.5246	0.6623	0.6881	0.595
1.0e-4	0.6548	0.5030	0.4128	0.5852	0.6657	0.6915	0.628
1.0e-5	0.6950	0.3198	0.3235	0.6466	0.6513	0.6928	0.613
1.0e-6	0.7024	0.3198	0.3235	0.7021	0.6435	0.6944	0.319
1.0e-7	0.6993	0.3198	NA	0.2932	0.6341	0.6932	NA
				sSVM			
0.15	0.5884	0.3198	0.3235	0.5354	0.5879	0.6698	0.535
0.1	0.5902	0.3198	0.3235	0.5299	0.5996	0.6611	0.553
0.05	0.5796	0.3198	0.3235	0.5234	0.6093	0.6370	0.533
0.01	0.5434	0.5221	0.4931	0.5355	0.5901	0.6304	0.517
0.0010	0.5668	0.5268	0.5195	0.5199	0.6330	0.7107	0.549
1.0e-4	0.6188	0.7545	0.4650	0.5623	0.6604	0.7583	0.569
1.0e-5	0.7111	0.5162	0.3596	0.6112	0.6588	0.7642	0.605
1.0e-6	0.6982	0.5101	0.3235	0.5939	0.6639	0.7614	0.511
1.0e-7	0.6993	0.3198	NA	0.2932	0.6681	0.7597	NA
				AdaBoostM1			
0.15	0.5958	0.5129	0.4950	0.5468	0.6345	0.6930	0.529
0.1	0.6086	0.5064	0.5172	0.5357	0.6361	0.6927	0.535
0.05	0.5954	0.4945	0.5098	0.5467	0.6513	0.6999	0.538
0.01	0.5992	0.5134	0.5177	0.5385	0.5942	0.6700	0.524
0.0010	0.6176	0.5496	0.5178	0.5393	0.6560	0.7151	0.572
1.0e-4	0.6696	0.2744	0.2649	0.6306	0.6924	0.7678	0.644
1.0e-5	0.7176	0.6473	0.3235	0.6566	0.6835	0.7778	0.689
1.0e-6	0.7214	0.3198	0.3235	0.7021	0.7175	0.7789	0.319
1.0e-7	0.6993	0.3198	NA	0.2932	0.6494	0.7775	NA
				C4.5			
0.15	0.6011	0.1888	0.1860	0.7433	0.6162	0.5987	0.554
0.1	0.6172	0.1888	0.1860	0.7433	0.5883	0.6198	0.538
0.05	0.6275	0.1888	0.1860	0.5410	0.6407	0.6350	0.583
0.01	0.5953	0.1888	0.1860	0.5310	0.6281	0.6512	0.540
0.0010	0.5897	0.5206	0.5029	0.5321	0.6361	0.6750	0.592
1.0e-4	0.6203	0.1887	0.5144	0.5374	0.6278	0.7043	0.575
1.0e-5	0.6970	0.4947	0.3723	0.6440	0.6430	0.7077	0.700
1.0e-6	0.7027	0.7549	0.3235	0.7021	0.6498	0.7085	0.319
1.0e-7	0.6993	0.3198	NA	0.2932	0.6242	0.7041	NA
				20RF			
0.15	0.3113	0.3198	0.3235	0.2932	0.3101	0.3249	0.319
0.1	0.3113	0.3198	0.3234	0.2932	0.3101	0.3249	0.319
0.05	0.3113	0.3198	0.3235	0.2932	0.3101	0.3249	0.319
0.01	0.3113	0.3198	0.3235	0.2932	0.3101	0.3249	0.319
0.0010	0.3113	0.3198	0.3235	0.2932	0.3101	0.3249	0.319
1.0e-4	0.6993	0.3198	0.3235	0.7519	0.3101	0.7557	0.319
1.0e-5	0.7358	0.3198	0.6691	0.6829	0.3101	0.7562	0.646
1.0e-6	0.7280	0.4849	0.3235	0.7021	0.3101	0.7593	0.319
1.0e-7	0.6993	0.3198	NA	0.2932	0.3101	0.7453	NA

Table S67: Precision obtained by all the algorithms under the haplotype-based approach with 3-SNP haplotype length, dominant genetic model and holdout sampling. Results for each p-value threshold (column 1) are shown. The maximum p-value threshold used was 0.15.

p-value threshold	BD	CAD	HT	IBD	RA	T1D	T2D
				NBC			
0.15	0.5993	0.1888	0.1860	0.6320	0.6239	0.6394	0.564
0.1	0.6063	0.1888	0.1860	0.5783	0.6298	0.6386	0.565
0.05	0.5970	0.1888	0.1860	0.5200	0.6379	0.6563	0.573
0.01	0.6095	0.1888	0.1860	0.5642	0.6448	0.6877	0.568
0.0010	0.6252	0.1888	0.4563	0.5322	0.6486	0.7003	0.586
1.0e-4	0.6106	0.5659	0.1860	0.5789	0.6592	0.7127	0.602
1.0e-5	0.6098	0.5270	0.1860	0.7454	0.6477	0.7116	0.596
1.0e-6	0.7574	0.4855	0.1860	0.7470	0.6475	0.7083	0.756
1.0e-7	0.7576	0.1888	NA	0.7433	0.6494	0.7090	NA
				sSVM			
0.15	0.5674	0.3198	0.3235	0.5366	0.5743	0.6014	0.526
0.1	0.5603	0.3198	0.3235	0.5298	0.5788	0.5949	0.536
0.05	0.5636	0.3198	0.3235	0.5351	0.5628	0.6077	0.550
0.01	0.5659	0.5344	0.5075	0.5232	0.5815	0.6359	0.519
0.0010	0.5660	0.5433	0.5066	0.5135	0.6104	0.7035	0.547
1.0e-4	0.5680	0.5840	0.4911	0.5586	0.6479	0.7582	0.559
1.0e-5	0.6292	0.4703	0.1860	0.5615	0.6691	0.7699	0.574
1.0e-6	0.6380	0.4791	0.6805	0.5346	0.6660	0.7621	0.521
1.0e-7	0.6418	0.3198	NA	0.2932	0.6653	0.7577	NA
				AdaBoostM1			
0.15	0.5864	0.4302	0.5115	0.5910	0.6341	0.6931	0.541
0.1	0.6035	0.4913	0.5295	0.5326	0.6302	0.7007	0.558
0.05	0.5767	0.5446	0.5061	0.5337	0.6569	0.7024	0.583
0.01	0.5707	0.5471	0.4702	0.5307	0.6320	0.6679	0.543
0.0010	0.6254	0.5292	0.5160	0.5614	0.6632	0.7075	0.578
1.0e-4	0.6240	0.1888	0.1860	0.5786	0.7180	0.7725	0.629
1.0e-5	0.6503	0.4814	0.1860	0.7412	0.7430	0.7699	0.679
1.0e-6	0.7574	0.5095	0.1860	0.7473	0.7517	0.7802	0.738
1.0e-7	0.7576	0.1888	NA	0.7433	0.7483	0.7632	NA
				C4.5			
0.15	0.5808	0.1888	0.1860	0.2102	0.6232	0.6111	0.571
0.1	0.6379	0.1888	0.1860	0.2102	0.6549	0.6275	0.587
0.05	0.6168	0.1888	0.1860	0.5659	0.6450	0.6414	0.582
0.01	0.6025	0.1888	0.1860	0.5175	0.6392	0.6476	0.571
0.0010	0.6085	0.5178	0.5088	0.5406	0.6397	0.6764	0.572
1.0e-4	0.5690	0.1888	0.5093	0.5535	0.6837	0.6912	0.563
1.0e-5	0.7015	0.4757	0.1860	0.7412	0.6773	0.7297	0.677
1.0e-6	0.1954	0.1888	0.1860	0.7473	0.6812	0.7169	0.714
1.0e-7	0.1954	0.1888	NA	0.2102	0.6730	0.7018	NA
				20RF			
0.15	0.1954	0.4357	0.4133	0.2102	0.1963	0.1849	0.189
0.1	0.1954	0.1888	0.1860	0.2102	0.1963	0.1849	0.189
0.05	0.1954	0.6415	0.5519	0.2102	0.1963	0.1849	0.189
0.01	0.1954	0.6133	0.5147	0.2102	0.1963	0.1849	0.189
0.0010	0.1954	0.1888	0.1860	0.2102	0.7534	0.5478	0.189
1.0e-4	0.1954	0.1888	0.1855	0.7519	0.7510	0.7115	0.755
1.0e-5	0.7542	0.1888	0.1860	0.7406	0.7193	0.6888	0.701
1.0e-6	0.7572	0.4880	0.1860	0.7473	0.7182	0.7235	0.704
1.0e-7	0.7576	0.1888	NA	0.7433	0.7156	0.6971	NA

Table S68: Precision obtained by all the algorithms under the haplotype-based approach with 3-SNP haplotype length, recessive genetic model and holdout sampling. Results for each p-value threshold (column 1) are shown. The maximum p-value threshold used was 0.15.

p-value threshold	BD	CAD	HT	IBD	RA	T1D	T2D
				NBC			
0.15	0.6024	0.1888	0.1860	0.5468	0.6290	0.6584	0.576
0.1	0.6036	0.1888	0.1860	0.5428	0.6222	0.6673	0.577
0.05	0.6015	0.1888	0.1860	0.5411	0.6319	0.6822	0.576
0.01	0.5976	0.1888	0.7549	0.5543	0.6464	0.6840	0.566
0.0010	0.5949	0.5028	0.3161	0.5431	0.6527	0.6805	0.584
1.0e-4	0.6105	0.5371	0.5168	0.5554	0.6479	0.6794	0.561
1.0e-5	0.6154	0.7547	0.3235	0.5578	0.6390	0.6908	0.566
1.0e-6	0.6464	0.3198	NA	0.5295	0.6374	0.6864	0.536
1.0e-7	0.6242	0.3198	NA	0.2932	0.6446	0.6867	NA
				sSVM			
0.15	0.6020	0.3198	0.3235	0.5619	0.6486	0.6660	0.600
0.1	0.5916	0.3198	0.3235	0.5403	0.6436	0.6898	0.602
0.05	0.6004	0.3205	0.3235	0.5341	0.6163	0.7016	0.578
0.01	0.5805	0.4939	0.4510	0.5377	0.6047	0.7148	0.547
0.0010	0.5754	0.5203	0.5275	0.5177	0.6462	0.7899	0.547
1.0e-4	0.6395	0.6338	0.4818	0.5532	0.7154	0.8290	0.562
1.0e-5	0.7018	0.5590	0.6227	0.5762	0.7156	0.8301	0.568
1.0e-6	0.7258	0.5580	NA	0.5840	0.7225	0.8275	0.540
1.0e-7	0.3251	0.3247	NA	0.2932	0.7207	0.8236	NA
				AdaBoostM1			
0.15	0.5857	0.5355	0.4902	0.5347	0.6453	0.7365	0.556
0.1	0.6008	0.7545	0.5303	0.5412	0.6430	0.7199	0.551
0.05	0.5821	0.5050	0.5040	0.5425	0.6452	0.7181	0.548
0.01	0.5842	0.5662	0.5247	0.5244	0.6250	0.6877	0.545
0.0010	0.6177	0.5183	0.5215	0.6339	0.6325	0.7129	0.571
1.0e-4	0.6112	0.3198	0.5018	0.5437	0.6912	0.7622	0.558
1.0e-5	0.6179	0.3198	0.3232	0.5811	0.6815	0.7769	0.574
1.0e-6	0.6464	0.3198	NA	0.5295	0.6945	0.7774	0.536
1.0e-7	0.6242	0.3198	NA	0.2932	0.6860	0.7676	NA
				C4.5			
0.15	0.6129	0.1888	0.1860	0.2279	0.6304	0.6067	0.572
0.1	0.6086	0.1888	0.1860	0.2279	0.6409	0.5883	0.573
0.05	0.6167	0.1888	0.1860	0.6202	0.6110	0.6007	0.560
0.01	0.6172	0.1888	0.1858	0.5690	0.6247	0.6632	0.567
0.0010	0.6004	0.5125	0.5353	0.5997	0.6237	0.6525	0.563
1.0e-4	0.5929	0.6361	0.4916	0.5541	0.6233	0.6545	0.536
1.0e-5	0.6241	0.5308	0.6432	0.5554	0.6294	0.6695	0.568
1.0e-6	0.6399	0.4974	NA	0.5295	0.6216	0.6758	0.524
1.0e-7	0.6242	0.7552	NA	0.2932	0.6228	0.6688	NA
				20RF			
0.15	0.5773	0.5179	0.5334	0.5195	0.5509	0.5131	0.492
0.1	0.5140	0.5268	0.5040	0.4846	0.5546	0.6387	0.526
0.05	0.5597	0.5150	0.5114	0.5628	0.6078	0.5051	0.481
0.01	0.4826	0.5341	0.4659	0.5666	0.5890	0.6596	0.538
0.0010	0.6428	0.4716	0.5119	0.5468	0.6048	0.7090	0.572
1.0e-4	0.6573	0.5652	0.5067	0.5298	0.6601	0.7331	0.562
1.0e-5	0.6576	0.6461	0.3235	0.5818	0.6582	0.7299	0.576
1.0e-6	0.6464	0.5008	NA	0.5295	0.6692	0.7386	0.530
1.0e-7	0.6242	0.4852	NA	0.2932	0.6755	0.7312	NA

Table S69: Precision obtained by all the algorithms under the haplotype-based approach with 4-SNP haplotype length, additive genetic model and holdout sampling. Results for each p-value threshold (column 1) are shown. The maximum p-value threshold used was 0.15.

p-value threshold	BD	CAD	HT	IBD	RA	T1D	T2D
				NBC			
0.15	0.6183	0.1888	0.1860	0.5391	0.6336	0.6629	0.580
0.1	0.6014	0.1888	0.1860	0.5383	0.6322	0.6634	0.574
0.05	0.6334	0.1888	0.1860	0.5287	0.6468	0.6857	0.581
0.01	0.6255	0.1888	0.4494	0.5208	0.6552	0.6896	0.572
0.0010	0.5979	0.5439	0.4834	0.5279	0.6589	0.6793	0.583
1.0e-4	0.6348	0.3198	0.5086	0.5459	0.6513	0.6926	0.590
1.0e-5	0.7080	0.3198	0.3235	0.6127	0.6387	0.6883	0.693
1.0e-6	0.7184	0.3198	NA	0.2932	0.6401	0.6851	0.319
1.0e-7	0.3113	0.3198	NA	0.2932	0.6392	0.6845	NA
				sSVM			
0.15	0.5795	0.3198	0.3235	0.5439	0.6323	0.6360	0.586
0.1	0.5623	0.3198	0.3235	0.5285	0.6272	0.6539	0.578
0.05	0.5792	0.3198	0.3235	0.5311	0.5909	0.6623	0.556
0.01	0.5615	0.4997	0.4524	0.5193	0.5771	0.6570	0.529
0.0010	0.5517	0.5234	0.5234	0.5006	0.5984	0.7063	0.529
1.0e-4	0.6088	0.6308	0.4918	0.5319	0.6576	0.7474	0.542
1.0e-5	0.6892	0.5663	0.6112	0.5503	0.6583	0.7516	0.549
1.0e-6	0.7184	0.5652	NA	0.5816	0.6739	0.7579	0.524
1.0e-7	0.3113	0.3198	NA	0.2932	0.6746	0.7564	NA
				AdaBoostM1			
0.15	0.5851	0.5664	0.5075	0.5232	0.6292	0.7100	0.563
0.1	0.6125	0.5346	0.5343	0.5547	0.6341	0.6977	0.546
0.05	0.5917	0.5041	0.5138	0.5369	0.6248	0.7157	0.565
0.01	0.5731	0.5071	0.5144	0.5360	0.6145	0.6749	0.572
0.0010	0.6050	0.5201	0.5650	0.5532	0.6273	0.6971	0.575
1.0e-4	0.6617	0.3198	0.5088	0.5433	0.6709	0.7600	0.628
1.0e-5	0.7264	0.3198	0.3235	0.5991	0.6777	0.7554	0.693
1.0e-6	0.7184	0.3198	NA	0.2932	0.6768	0.7614	0.319
1.0e-7	0.3113	0.3198	NA	0.2932	0.6393	0.7671	NA
				C4.5			
0.15	0.6062	0.1888	0.1860	0.7604	0.6190	0.6122	0.539
0.1	0.6201	0.1888	0.1860	0.7262	0.6454	0.5955	0.572
0.05	0.6217	0.1888	0.1860	0.5593	0.6160	0.6213	0.564
0.01	0.6115	0.1888	0.4703	0.5395	0.6190	0.6400	0.557
0.0010	0.6045	0.5021	0.5341	0.5281	0.6163	0.6579	0.552
1.0e-4	0.5825	0.6369	0.5096	0.5331	0.6314	0.6777	0.543
1.0e-5	0.7312	0.7545	0.3235	0.2932	0.6454	0.7099	0.681
1.0e-6	0.7184	0.6099	NA	0.2932	0.6624	0.7089	0.319
1.0e-7	0.3113	0.3198	NA	0.2932	0.6309	0.7145	NA
				20RF			
0.15	0.3113	0.3198	0.3235	0.2932	0.3101	0.3249	0.319
0.1	0.3113	0.3198	0.3235	0.2932	0.3101	0.3249	0.319
0.05	0.3113	0.3198	0.3235	0.2932	0.3101	0.3249	0.319
0.01	0.3113	0.3198	0.3235	0.2932	0.3101	0.3249	0.319
0.0010	0.3113	0.3198	0.3235	0.2932	0.3101	0.3249	0.319
1.0e-4	0.3113	0.3198	0.3235	0.4076	0.3101	0.7551	0.319
1.0e-5	0.7433	0.3198	0.3235	0.5482	0.3101	0.7560	0.754
1.0e-6	0.7184	0.5060	NA	0.2932	0.3101	0.7591	0.319
1.0e-7	0.3113	0.5576	NA	0.2932	0.3101	0.7438	NA

Table S70: Precision obtained by all the algorithms under the haplotype-based approach with 4-SNP haplotype length, dominant genetic model and holdout sampling. Results for each p-value threshold (column 1) are shown. The maximum p-value threshold used was 0.15.

p-value threshold	BD	CAD	HT	IBD	RA	T1D	T2D
NBC							
0.15	0.6067	0.1888	0.1860	0.5382	0.6280	0.6357	0.567
0.1	0.6020	0.1888	0.1860	0.5329	0.6186	0.6401	0.584
0.05	0.5974	0.1888	0.1860	0.5340	0.6255	0.6705	0.571
0.01	0.5873	0.1888	0.1860	0.4804	0.6377	0.6886	0.569
0.0010	0.5943	0.4715	0.1858	0.5526	0.6430	0.7037	0.595
1.0e-4	0.6088	0.5243	0.1858	0.5827	0.6447	0.7025	0.577
1.0e-5	0.6268	0.5180	0.6343	0.7569	0.6432	0.6999	0.648
1.0e-6	0.7512	0.5356	NA	0.7586	0.6504	0.7055	0.667
1.0e-7	0.7518	0.6106	NA	0.7604	0.6459	0.7035	NA
sSVM							
0.15	0.5769	0.3198	0.3235	0.5430	0.5807	0.6113	0.549
0.1	0.5729	0.3198	0.3235	0.5293	0.5679	0.6181	0.563
0.05	0.5621	0.7547	0.3235	0.5131	0.5728	0.6192	0.553
0.01	0.5511	0.4962	0.5013	0.5334	0.5684	0.6443	0.538
0.0010	0.5539	0.5094	0.5191	0.5227	0.6077	0.7108	0.538
1.0e-4	0.5807	0.5385	0.4878	0.5388	0.6559	0.7562	0.545
1.0e-5	0.6047	0.4923	0.6235	0.5539	0.6647	0.7684	0.545
1.0e-6	0.6399	0.4928	NA	0.5195	0.6657	0.7563	0.534
1.0e-7	0.6242	0.7567	NA	0.2932	0.6653	0.7545	NA
AdaBoostM1							
0.15	0.5661	0.5308	0.5253	0.5360	0.6501	0.7211	0.557
0.1	0.5675	0.1888	0.5033	0.5265	0.6417	0.7094	0.563
0.05	0.5750	0.5121	0.5105	0.5398	0.6428	0.7011	0.562
0.01	0.5696	0.1885	0.5132	0.5113	0.6165	0.6670	0.574
0.0010	0.6092	0.5108	0.5046	0.6438	0.6353	0.6951	0.591
1.0e-4	0.6157	0.6473	0.5038	0.5790	0.7115	0.7527	0.619
1.0e-5	0.6803	0.5652	0.6151	0.7569	0.7304	0.7717	0.663
1.0e-6	0.7515	0.5752	NA	0.7586	0.7407	0.7726	0.667
1.0e-7	0.7518	0.6335	NA	0.7604	0.7471	0.7650	NA
C4.5							
0.15	0.6165	0.1888	0.1860	0.2102	0.6251	0.6037	0.551
0.1	0.6295	0.1888	0.1860	0.2102	0.6330	0.5985	0.585
0.05	0.6074	0.1888	0.1860	0.6171	0.6106	0.6436	0.590
0.01	0.6213	0.1888	0.1860	0.5621	0.6424	0.6816	0.575
0.0010	0.5935	0.5210	0.5186	0.5292	0.6296	0.6578	0.549
1.0e-4	0.5959	0.5083	0.5071	0.5438	0.6589	0.6832	0.541
1.0e-5	0.6705	0.5175	0.1860	0.7569	0.6481	0.6867	0.699
1.0e-6	0.1954	0.4597	NA	0.7586	0.6473	0.7025	0.711
1.0e-7	0.1954	0.1888	NA	0.2102	0.6459	0.6938	NA
20RF							
0.15	0.1954	0.4715	0.7549	0.2102	0.1963	0.1849	0.189
0.1	0.1954	0.7545	0.1858	0.2102	0.1963	0.1849	0.189
0.05	0.1954	0.5489	0.4892	0.2102	0.1963	0.1849	0.189
0.01	0.1954	0.4714	0.6241	0.2102	0.1963	0.1849	0.189
0.0010	0.1954	0.1888	0.1860	0.2102	0.1963	0.6375	0.189
1.0e-4	0.7536	0.5526	0.1860	0.7519	0.7252	0.7139	0.684
1.0e-5	0.7542	0.4759	0.6205	0.7072	0.7269	0.7120	0.728
1.0e-6	0.7510	0.4913	NA	0.7586	0.7064	0.7167	0.714
1.0e-7	0.7518	0.2307	NA	0.7604	0.7073	0.7201	NA

Table S71: Precision obtained by all the algorithms under the haplotype-based approach with 4-SNP haplotype length, recessive genetic model and holdout sampling. Results for each p-value threshold (column 1) are shown. The maximum p-value threshold used was 0.15.

p-value threshold	BD	CAD	HT	IBD	RA	T1D	T2D
				NBC			
0.15	0.5909	0.1888	0.1860	0.5439	0.6327	0.6626	0.582
0.1	0.6136	0.1888	0.1860	0.5527	0.6419	0.6691	0.585
0.05	0.5883	0.1888	0.1860	0.5252	0.6448	0.6759	0.568
0.01	0.5936	0.6133	0.1860	0.5361	0.6557	0.6886	0.573
0.0010	0.5949	0.1888	0.1860	0.5408	0.6430	0.6863	0.577
1.0e-4	0.6125	0.4148	0.5133	0.5436	0.6438	0.6838	0.557
1.0e-5	0.6557	0.5099	0.3235	0.5551	0.6393	0.6795	0.566
1.0e-6	0.6575	0.5615	NA	0.5336	0.6292	0.6895	0.319
1.0e-7	0.6353	0.3198	NA	0.2932	0.6412	0.6932	NA
				sSVM			
0.15	0.5985	0.3198	0.3235	0.5743	0.6490	0.6780	0.585
0.1	0.5968	0.3198	0.3235	0.6054	0.6508	0.6970	0.584
0.05	0.5820	0.3198	0.3235	0.5524	0.6310	0.6728	0.551
0.01	0.5792	0.5010	0.5388	0.5407	0.6139	0.7074	0.551
0.0010	0.5685	0.5908	0.5483	0.5458	0.6582	0.7796	0.571
1.0e-4	0.6035	0.5088	0.7562	0.5507	0.6904	0.8195	0.555
1.0e-5	0.7281	0.3198	0.3235	0.5722	0.7038	0.8229	0.580
1.0e-6	0.7085	0.3198	NA	0.5328	0.7021	0.8213	0.319
1.0e-7	0.6672	0.3198	NA	0.2932	0.6957	0.8107	NA
				AdaBoostM1			
0.15	0.5894	0.5200	0.4947	0.5525	0.6282	0.7157	0.542
0.1	0.5749	0.5269	0.4933	0.5634	0.6269	0.7205	0.562
0.05	0.5768	0.5069	0.5123	0.5347	0.6299	0.7146	0.539
0.01	0.5734	0.5454	0.5340	0.5118	0.5974	0.6800	0.546
0.0010	0.6082	0.5075	0.4939	0.5286	0.6199	0.6879	0.567
1.0e-4	0.6150	0.5084	0.4931	0.5346	0.6679	0.7460	0.574
1.0e-5	0.6595	0.4879	0.3235	0.5748	0.6736	0.7556	0.566
1.0e-6	0.6560	0.5024	NA	0.5336	0.6802	0.7572	0.319
1.0e-7	0.6353	0.3198	NA	0.2932	0.6769	0.7561	NA
				C4.5			
0.15	0.6137	0.1888	0.1860	0.7507	0.5725	0.6216	0.535
0.1	0.5914	0.1888	0.1860	0.5134	0.6232	0.6343	0.543
0.05	0.6238	0.1888	0.1860	0.5353	0.6299	0.6288	0.556
0.01	0.6245	0.1888	0.1866	0.5185	0.6058	0.6455	0.532
0.0010	0.5717	0.1884	0.1860	0.5383	0.6119	0.6623	0.553
1.0e-4	0.5663	0.1881	0.5085	0.5110	0.6032	0.6762	0.549
1.0e-5	0.6343	0.7124	0.3235	0.5641	0.5963	0.6968	0.553
1.0e-6	0.6544	0.3198	NA	0.5336	0.5962	0.6503	0.319
1.0e-7	0.6353	0.3198	NA	0.2932	0.6036	0.6553	NA
				20RF			
0.15	0.4845	0.4932	0.5291	0.4924	0.3834	0.5947	0.503
0.1	0.4767	0.5039	0.4782	0.5188	0.4844	0.5404	0.549
0.05	0.5552	0.5246	0.5100	0.5310	0.5051	0.5574	0.535
0.01	0.5436	0.5024	0.4920	0.5077	0.5617	0.6324	0.492
0.0010	0.6023	0.5175	0.5458	0.5185	0.6126	0.7201	0.540
1.0e-4	0.6544	0.5129	0.5145	0.5458	0.6388	0.7249	0.548
1.0e-5	0.6591	0.7336	0.3235	0.5755	0.6552	0.7252	0.567
1.0e-6	0.6575	0.6111	NA	0.5336	0.6524	0.7261	0.319
1.0e-7	0.6353	0.5530	NA	0.2932	0.6663	0.7209	NA

Table S72: Precision obtained by all the algorithms under the haplotype-based approach with 5-SNP haplotype length, additive genetic model and holdout sampling. Results for each p-value threshold (column 1) are shown. The maximum p-value threshold used was 0.15.

p-value threshold	BD	CAD	HT	IBD	RA	T1D	T2D
			NBC				
0.15	0.6023	0.1888	0.1860	0.5525	0.6558	0.6677	0.585
0.1	0.6078	0.1888	0.1860	0.5548	0.6463	0.6670	0.581
0.05	0.5960	0.3766	0.1860	0.5228	0.6476	0.6767	0.552
0.01	0.6052	0.4658	0.1860	0.5403	0.6475	0.7015	0.553
0.0010	0.5985	0.4715	0.5274	0.5497	0.6547	0.6828	0.581
1.0e-4	0.6419	0.4827	0.5740	0.5331	0.6412	0.6877	0.555
1.0e-5	0.7153	0.3198	0.3235	0.5501	0.6318	0.6870	0.716
1.0e-6	0.6978	0.3198	NA	0.2932	0.6343	0.6851	0.319
1.0e-7	0.6602	0.3198	NA	0.2932	0.6300	0.6924	NA
			sSVM				
0.15	0.5784	0.3198	0.3235	0.5681	0.6326	0.6457	0.566
0.1	0.5647	0.3198	0.3235	0.5997	0.6264	0.6637	0.565
0.05	0.5657	0.3198	0.3235	0.5410	0.5963	0.6398	0.542
0.01	0.5617	0.5037	0.5300	0.5333	0.5794	0.6426	0.536
0.0010	0.5502	0.5048	0.5524	0.5404	0.6054	0.6948	0.553
1.0e-4	0.5773	0.5276	0.7549	0.5381	0.6216	0.7460	0.543
1.0e-5	0.7177	0.3198	0.3235	0.5391	0.6503	0.7517	0.567
1.0e-6	0.6978	0.3198	NA	0.5270	0.6483	0.7500	0.319
1.0e-7	0.6602	0.3198	NA	0.2932	0.6482	0.7446	NA
			AdaBoostM1				
0.15	0.5883	0.5315	0.5107	0.5366	0.6097	0.6965	0.539
0.1	0.5773	0.5382	0.5012	0.5578	0.6211	0.7011	0.563
0.05	0.5956	0.5088	0.5087	0.5409	0.6309	0.6946	0.532
0.01	0.5653	0.5114	0.5133	0.5355	0.5908	0.6645	0.549
0.0010	0.6092	0.5090	0.4935	0.5352	0.6235	0.6868	0.576
1.0e-4	0.6691	0.6171	0.4753	0.4851	0.6426	0.7459	0.634
1.0e-5	0.7177	0.5704	0.3235	0.7519	0.6523	0.7562	0.708
1.0e-6	0.6978	0.5704	NA	0.2932	0.6399	0.7638	0.319
1.0e-7	0.6602	0.3198	NA	0.2932	0.5988	0.7569	NA
			C4.5				
0.15	0.6051	0.1888	0.1860	0.6919	0.5697	0.6436	0.548
0.1	0.6007	0.1888	0.1860	0.5050	0.6156	0.6381	0.543
0.05	0.6243	0.1888	0.1860	0.5287	0.6144	0.6421	0.563
0.01	0.6191	0.1888	0.7551	0.5025	0.6115	0.6425	0.529
0.0010	0.5775	0.4713	0.5273	0.5296	0.5750	0.6678	0.539
1.0e-4	0.5763	0.3769	0.5217	0.5203	0.5968	0.6888	0.551
1.0e-5	0.7070	0.3198	0.3235	0.2931	0.5967	0.6930	0.655
1.0e-6	0.6978	0.3198	NA	0.2932	0.6101	0.6973	0.319
1.0e-7	0.6602	0.3198	NA	0.2932	0.6134	0.6877	NA
			20RF				
0.15	0.3113	0.3198	0.3235	0.2932	0.3101	0.3249	0.319
0.1	0.3113	0.3198	0.3235	0.2932	0.3101	0.3249	0.319
0.05	0.3113	0.3198	0.3235	0.2932	0.3101	0.3249	0.319
0.01	0.3113	0.3196	0.3235	0.2932	0.3101	0.3249	0.319
0.0010	0.3113	0.5371	0.3235	0.2932	0.3101	0.3249	0.319
1.0e-4	0.3113	0.3198	0.3235	0.2932	0.3101	0.3249	0.319
1.0e-5	0.7277	0.3198	0.3235	0.2928	0.3101	0.7558	0.754
1.0e-6	0.6978	0.3198	NA	0.2932	0.3101	0.7325	0.319
1.0e-7	0.6602	0.3198	NA	0.2932	0.3101	0.7422	NA

Table S73: Precision obtained by all the algorithms under the haplotype-based approach with 5-SNP haplotype length, dominant genetic model and holdout sampling. Results for each p-value threshold (column 1) are shown. The maximum p-value threshold used was 0.15.

p-value threshold	BD	CAD	HT	IBD	RA	T1D	T2D
			NBC				
0.15	0.5931	0.1888	0.1860	0.5512	0.6197	0.6536	0.568
0.1	0.5998	0.1888	0.1860	0.5457	0.6204	0.6588	0.564
0.05	0.5847	0.1888	0.1860	0.5274	0.6284	0.6716	0.567
0.01	0.5723	0.7544	0.1860	0.5307	0.6452	0.6894	0.560
0.0010	0.5879	0.1888	0.1860	0.5295	0.6465	0.6976	0.571
1.0e-4	0.5845	0.1888	0.1858	0.5594	0.6444	0.7056	0.565
1.0e-5	0.6736	0.4900	0.1860	0.7477	0.6451	0.7013	0.644
1.0e-6	0.7450	0.4965	NA	0.7338	0.6433	0.7088	0.189
1.0e-7	0.7457	0.1888	NA	0.7433	0.6511	0.7047	NA
			sSVM				
0.15	0.5698	0.3198	0.3235	0.5310	0.5774	0.6171	0.554
0.1	0.5793	0.3198	0.3235	0.5424	0.5863	0.6156	0.554
0.05	0.5493	0.3198	0.3235	0.5349	0.5888	0.6006	0.530
0.01	0.5485	0.5037	0.5270	0.5213	0.5802	0.6481	0.537
0.0010	0.5456	0.5913	0.4962	0.5219	0.6175	0.7089	0.547
1.0e-4	0.5675	0.4991	0.5988	0.5316	0.6534	0.7408	0.535
1.0e-5	0.6318	0.3198	0.3235	0.5587	0.6582	0.7548	0.543
1.0e-6	0.6544	0.3198	NA	0.5213	0.6557	0.7571	0.319
1.0e-7	0.6353	0.3198	NA	0.2932	0.6506	0.7481	NA
			AdaBoostM1				
0.15	0.5759	0.4979	0.4816	0.5210	0.6203	0.7072	0.558
0.1	0.5781	0.5170	0.4830	0.5492	0.6276	0.7030	0.550
0.05	0.5711	0.4896	0.5266	0.5157	0.6078	0.6835	0.543
0.01	0.5496	0.5402	0.5389	0.4941	0.6007	0.6601	0.562
0.0010	0.6079	0.5539	0.4703	0.5386	0.6332	0.6763	0.572
1.0e-4	0.6007	0.4927	0.3754	0.5435	0.6965	0.7344	0.578
1.0e-5	0.6724	0.4921	0.4671	0.7406	0.7080	0.7521	0.648
1.0e-6	0.7454	0.4919	NA	0.7338	0.7116	0.7591	0.725
1.0e-7	0.7457	0.1888	NA	0.7433	0.7260	0.7471	NA
			C4.5				
0.15	0.5917	0.1888	0.1860	0.7519	0.5983	0.6310	0.562
0.1	0.5805	0.1888	0.1860	0.5424	0.6347	0.6369	0.568
0.05	0.6004	0.1888	0.1860	0.5276	0.6337	0.6303	0.566
0.01	0.6221	0.1888	0.1860	0.5190	0.6445	0.6251	0.547
0.0010	0.5741	0.1888	0.1860	0.5316	0.6093	0.6521	0.557
1.0e-4	0.5973	0.1888	0.5191	0.5060	0.6228	0.6768	0.527
1.0e-5	0.4743	0.5656	0.1860	0.7406	0.6227	0.7030	0.614
1.0e-6	0.1954	0.1888	NA	0.7338	0.6044	0.6677	0.189
1.0e-7	0.1954	0.1888	NA	0.2102	0.6355	0.6893	NA
			20RF				
0.15	0.1954	0.3771	0.1857	0.2102	0.1963	0.1849	0.189
0.1	0.1954	0.5425	0.5416	0.2102	0.1963	0.1849	0.189
0.05	0.1954	0.5659	0.4225	0.2102	0.1963	0.1849	0.189
0.01	0.1954	0.1888	0.1860	0.2102	0.1963	0.1849	0.189
0.0010	0.1954	0.1888	0.1860	0.2102	0.7535	0.6800	0.189
1.0e-4	0.1954	0.5075	0.1860	0.7519	0.6750	0.7066	0.754
1.0e-5	0.7205	0.1888	0.5533	0.7119	0.7078	0.7054	0.701
1.0e-6	0.7450	0.5348	NA	0.7338	0.6915	0.6872	0.189
1.0e-7	0.7457	0.4262	NA	0.7433	0.7013	0.6989	NA

Table S74: Precision obtained by all the algorithms under the haplotype-based approach with 5-SNP haplotype length, recessive genetic model and holdout sampling. Results for each p-value threshold (column 1) are shown. The maximum p-value threshold used was 0.15.

p-value threshold	BD	CAD	HT	IBD	RA	T1D	T2D
				NBC			
0.15	0.5747	0.4345	0.4312	0.5421	0.5874	0.6153	0.560
0.1	0.5729	0.4345	0.4312	0.4820	0.5952	0.6322	0.562
0.05	0.5699	0.4345	0.4312	0.4758	0.6036	0.6438	0.545
0.01	0.5669	0.4345	0.4312	0.4603	0.6317	0.6496	0.532
0.0010	0.5311	0.4345	0.4312	0.5223	0.6407	0.6461	0.511
1.0e-4	0.5352	0.4345	0.4312	0.5310	0.6455	0.6444	0.541
1.0e-5	0.5376	0.5062	0.4312	0.5297	0.6515	0.6647	0.568
1.0e-6	NA	0.5643	NA	NA	0.6329	0.6694	0.561
1.0e-7	NA	0.5267	NA	NA	0.6240	0.6880	NA
				sSVM			
0.15	0.4922	0.5655	0.5688	0.3271	0.4856	0.4660	0.390
0.1	0.4295	0.5655	0.5688	0.2801	0.4174	0.4370	0.354
0.05	0.3859	0.5655	0.5688	0.2782	0.3796	0.4149	0.323
0.01	0.3333	0.4739	0.5064	0.3532	0.3509	0.4323	0.306
0.0010	0.2993	0.2684	0.2640	0.3036	0.3844	0.5235	0.293
1.0e-4	0.3184	0.2895	0.2791	0.2825	0.4695	0.5816	0.332
1.0e-5	0.3984	0.4093	0.4073	0.3036	0.5030	0.6014	0.271
1.0e-6	NA	0.5167	NA	NA	0.4952	0.6014	0.381
1.0e-7	NA	0.5473	NA	NA	0.4910	0.5880	NA
				AdaBoostM1			
0.15	0.5478	0.4574	0.4388	0.4647	0.6287	0.7141	0.534
0.1	0.5579	0.5602	0.4633	0.4808	0.6269	0.7060	0.544
0.05	0.5526	0.5508	0.4942	0.4969	0.6222	0.7095	0.531
0.01	0.5603	0.4545	0.4371	0.5285	0.6036	0.6717	0.531
0.0010	0.5358	0.5338	0.4674	0.5390	0.6515	0.6915	0.523
1.0e-4	0.5562	0.4692	0.4318	0.5347	0.6844	0.7589	0.561
1.0e-5	0.5376	0.5385	0.4312	0.5545	0.7012	0.7548	0.568
1.0e-6	NA	0.5349	NA	NA	0.6844	0.7548	0.564
1.0e-7	NA	0.5267	NA	NA	0.6737	0.7623	NA
				C4.5			
0.15	0.4146	0.4709	0.5268	0.2627	0.4551	0.4956	0.414
0.1	0.4104	0.4169	0.3141	0.2280	0.5084	0.4980	0.445
0.05	0.4534	0.5373	0.3980	0.2429	0.5054	0.5508	0.444
0.01	0.4301	0.4962	0.2506	0.3463	0.5311	0.5474	0.445
0.0010	0.4415	0.4962	0.5146	0.3947	0.5263	0.5660	0.442
1.0e-4	0.5329	0.4451	0.4312	0.5136	0.5719	0.6026	0.549
1.0e-5	0.5579	0.5291	0.4312	0.5465	0.6377	0.6310	0.548
1.0e-6	NA	0.5684	NA	NA	0.6180	0.6537	0.554
1.0e-7	NA	0.5678	NA	NA	0.6198	0.6595	NA
				20RF			
0.15	0.5532	0.4979	0.4994	0.5403	0.5557	0.5683	0.570
0.1	0.5597	0.5578	0.5355	0.5328	0.5569	0.5718	0.568
0.05	0.5585	0.5514	0.5425	0.5328	0.5557	0.5706	0.564
0.01	0.5550	0.5332	0.4901	0.5167	0.5611	0.5735	0.571
0.0010	0.5478	0.5455	0.5315	0.5409	0.5808	0.6299	0.571
1.0e-4	0.5603	0.5502	0.4569	0.5266	0.6491	0.7246	0.546
1.0e-5	0.5508	0.5091	0.4312	0.5551	0.6683	0.7135	0.564
1.0e-6	NA	0.5655	NA	NA	0.6605	0.7130	0.561
1.0e-7	NA	0.5766	NA	NA	0.6808	0.7286	NA

Table S75: Overall accuracy obtained by all the algorithms under the haplotype-based approach with 1-SNP haplotype length, additive genetic model and holdout sampling. Results for each p-value threshold (column 1) are shown. The maximum p-value threshold used was 0.15.

p-value threshold	BD	CAD	HT	IBD	RA	T1D	T2D
				NBC			
0.15	0.5717	0.4345	0.4312	0.5415	0.5731	0.6130	0.562
0.1	0.5711	0.4345	0.4312	0.4913	0.5760	0.6165	0.561
0.05	0.5795	0.4345	0.4312	0.4919	0.5772	0.6345	0.562
0.01	0.5717	0.4345	0.4312	0.4690	0.5976	0.6612	0.553
0.0010	0.5579	0.4345	0.4312	0.5446	0.6311	0.6653	0.543
1.0e-4	0.5550	0.4404	0.4312	0.5428	0.6479	0.6665	0.565
1.0e-5	0.5573	0.5625	0.4277	0.5465	0.6377	0.6793	0.572
1.0e-6	NA	0.5655	NA	NA	0.6281	0.6909	0.565
1.0e-7	NA	0.5655	NA	NA	0.6275	0.7019	NA
				sSVM			
0.15	0.5645	0.5655	0.5688	0.5595	0.5587	0.5845	0.569
0.1	0.5693	0.5655	0.5688	0.5440	0.5641	0.6014	0.568
0.05	0.5723	0.5655	0.5688	0.5279	0.5665	0.6089	0.562
0.01	0.5693	0.5661	0.5699	0.5000	0.5766	0.6293	0.560
0.0010	0.5573	0.5320	0.5140	0.5359	0.5904	0.7002	0.547
1.0e-4	0.5448	0.5573	0.5490	0.5254	0.6299	0.7286	0.568
1.0e-5	0.5544	0.5678	0.4277	0.5471	0.6521	0.7379	0.554
1.0e-6	NA	0.5678	NA	NA	0.6521	0.7350	0.560
1.0e-7	NA	0.5649	NA	NA	0.6515	0.7310	NA
				AdaBoostM1			
0.15	0.5526	0.4850	0.4633	0.4827	0.6114	0.6729	0.552
0.1	0.5538	0.5637	0.4854	0.5025	0.5988	0.6717	0.543
0.05	0.5657	0.5602	0.5082	0.5378	0.5994	0.6769	0.555
0.01	0.5609	0.4797	0.4779	0.5390	0.5874	0.6351	0.546
0.0010	0.5520	0.5590	0.5169	0.5353	0.6210	0.6746	0.547
1.0e-4	0.5550	0.5555	0.4703	0.5409	0.6078	0.7008	0.566
1.0e-5	0.5573	0.5643	0.4277	0.5415	0.5754	0.7042	0.565
1.0e-6	NA	0.5655	NA	NA	0.5725	0.7037	0.565
1.0e-7	NA	0.5655	NA	NA	0.5605	0.6955	NA
				C4.5			
0.15	0.5329	0.4821	0.5693	0.5229	0.5515	0.5898	0.552
0.1	0.5550	0.5678	0.5594	0.5043	0.5808	0.5857	0.541
0.05	0.5538	0.5666	0.4551	0.5118	0.5862	0.6101	0.548
0.01	0.5603	0.5649	0.5017	0.5440	0.6030	0.5991	0.549
0.0010	0.5388	0.5643	0.5664	0.4932	0.5784	0.6386	0.548
1.0e-4	0.5603	0.5467	0.4289	0.5347	0.6126	0.6601	0.561
1.0e-5	0.5579	0.5596	0.4277	0.5415	0.6407	0.6688	0.565
1.0e-6	NA	0.5655	NA	NA	0.5958	0.6723	0.565
1.0e-7	NA	0.5655	NA	NA	0.5808	0.6915	NA
				20RF			
0.15	0.5579	0.5655	0.5688	0.5415	0.5569	0.5700	0.565
0.1	0.5579	0.5655	0.5688	0.5415	0.5569	0.5700	0.565
0.05	0.5579	0.5655	0.5688	0.5415	0.5569	0.5700	0.565
0.01	0.5579	0.5655	0.5688	0.5415	0.5569	0.5700	0.565
0.0010	0.5579	0.5655	0.5688	0.5415	0.5569	0.5700	0.565
1.0e-4	0.5579	0.5655	0.5688	0.5415	0.5569	0.5723	0.565
1.0e-5	0.5573	0.5620	0.4277	0.5415	0.5569	0.5776	0.567
1.0e-6	NA	0.5655	NA	NA	0.5569	0.5840	0.565
1.0e-7	NA	0.5655	NA	NA	0.5569	0.5933	NA

Table S76: Overall accuracy obtained by all the algorithms under the haplotype-based approach with 1-SNP haplotype length, dominant genetic model and holdout sampling. Results for each p-value threshold (column 1) are shown. The maximum p-value threshold used was 0.15.

p-value threshold	BD	CAD	HT	IBD	RA	T1D	T2D
				NBC			
0.15	0.5836	0.4345	0.4312	0.5068	0.5731	0.5764	0.515
0.1	0.5729	0.4345	0.4312	0.4647	0.5790	0.5660	0.520
0.05	0.5346	0.4345	0.4312	0.4634	0.5880	0.5625	0.517
0.01	0.5269	0.4345	0.4312	0.4610	0.5934	0.5758	0.496
0.0010	0.4797	0.4345	0.4312	0.4851	0.6252	0.5845	0.495
1.0e-4	0.4779	0.4345	0.4312	0.5025	0.6293	0.6095	0.459
1.0e-5	0.4421	0.4345	0.4312	0.4585	0.6186	0.6252	0.451
1.0e-6	NA	0.4345	NA	NA	0.6216	0.6258	0.434
1.0e-7	NA	0.4345	NA	NA	0.6102	0.6427	NA
				sSVM			
0.15	0.5795	0.5655	0.5688	0.5310	0.5659	0.5997	0.544
0.1	0.5818	0.5655	0.5688	0.5037	0.5707	0.5921	0.537
0.05	0.5591	0.5655	0.5688	0.4932	0.5545	0.5828	0.517
0.01	0.5335	0.5455	0.5536	0.4647	0.5551	0.6241	0.498
0.0010	0.4952	0.4803	0.4569	0.5180	0.5994	0.6880	0.510
1.0e-4	0.5203	0.4792	0.4784	0.5099	0.6599	0.7327	0.527
1.0e-5	0.5335	0.5361	0.4312	0.5155	0.6575	0.7304	0.506
1.0e-6	NA	0.5802	NA	NA	0.6497	0.7350	0.554
1.0e-7	NA	0.5766	NA	NA	0.6401	0.7304	NA
				AdaBoostM1			
0.15	0.5257	0.4410	0.4324	0.4628	0.6102	0.6508	0.492
0.1	0.5293	0.5479	0.4406	0.4634	0.6024	0.6525	0.501
0.05	0.5215	0.5320	0.4586	0.4696	0.5886	0.6304	0.499
0.01	0.5179	0.4381	0.4347	0.4938	0.5844	0.6316	0.501
0.0010	0.4952	0.4903	0.4382	0.5192	0.6156	0.6194	0.495
1.0e-4	0.4677	0.4392	0.4312	0.4827	0.6311	0.6688	0.456
1.0e-5	0.4421	0.4339	0.4312	0.4591	0.5976	0.6711	0.444
1.0e-6	NA	0.4345	NA	NA	0.5862	0.6752	0.434
1.0e-7	NA	0.4345	NA	NA	0.5760	0.6775	NA
				C4.5			
0.15	0.4875	0.4375	0.5641	0.4957	0.5329	0.5224	0.470
0.1	0.4875	0.5373	0.4889	0.4560	0.5557	0.5253	0.505
0.05	0.5042	0.5643	0.4324	0.4820	0.5371	0.5660	0.475
0.01	0.5102	0.5590	0.4429	0.4888	0.5743	0.5607	0.473
0.0010	0.5197	0.5126	0.4854	0.4616	0.5784	0.5828	0.492
1.0e-4	0.5012	0.4680	0.4312	0.5031	0.6138	0.6310	0.467
1.0e-5	0.4421	0.4245	0.4312	0.4585	0.6407	0.6787	0.434
1.0e-6	NA	0.4762	NA	NA	0.6174	0.6624	0.434
1.0e-7	NA	0.4345	NA	NA	0.6174	0.6822	NA
				20RF			
0.15	0.4421	0.4345	0.4312	0.4585	0.4431	0.4300	0.434
0.1	0.4421	0.4339	0.4301	0.4585	0.4431	0.4300	0.434
0.05	0.4421	0.4351	0.4312	0.4585	0.4431	0.4300	0.434
0.01	0.4421	0.4351	0.4312	0.4585	0.4431	0.4300	0.434
0.0010	0.4421	0.4345	0.4318	0.4566	0.4437	0.4329	0.434
1.0e-4	0.4415	0.4363	0.4312	0.4585	0.4868	0.4689	0.437
1.0e-5	0.4438	0.4351	0.4312	0.4585	0.5401	0.5009	0.447
1.0e-6	NA	0.4839	NA	NA	0.5425	0.4892	0.434
1.0e-7	NA	0.4345	NA	NA	0.5659	0.5259	NA

Table S77: Overall accuracy obtained by all the algorithms under the haplotype-based approach with 1-SNP haplotype length, recessive genetic model and holdout sampling. Results for each p-value threshold (column 1) are shown. The maximum p-value threshold used was 0.15.

p-value threshold	BD	CAD	HT	IBD	RA	T1D	T2D
				NBC			
0.15	0.6045	0.4345	0.4312	0.5589	0.6120	0.6456	0.578
0.1	0.6087	0.4345	0.4312	0.5477	0.6216	0.6549	0.591
0.05	0.6111	0.4345	0.4312	0.4758	0.6317	0.6700	0.590
0.01	0.6159	0.4345	0.4312	0.4591	0.6563	0.6781	0.574
0.0010	0.6129	0.4345	0.4312	0.5496	0.6611	0.6583	0.579
1.0e-4	0.6153	0.4339	0.4400	0.5688	0.6587	0.6647	0.600
1.0e-5	0.6171	0.4709	0.5950	0.5923	0.6557	0.6798	0.552
1.0e-6	0.6237	0.4504	0.5979	0.5601	0.6437	0.6816	0.525
1.0e-7	0.6075	0.4381	NA	0.5415	0.6359	0.6868	NA
				sSVM			
0.15	0.4665	0.5655	0.5688	0.4120	0.4593	0.4730	0.381
0.1	0.4146	0.5655	0.5688	0.3265	0.4144	0.4195	0.357
0.05	0.3877	0.5655	0.5688	0.2695	0.3808	0.3998	0.340
0.01	0.3363	0.4516	0.3683	0.3401	0.3599	0.4166	0.307
0.0010	0.3244	0.3359	0.3823	0.3228	0.3946	0.4997	0.298
1.0e-4	0.3548	0.5297	0.2710	0.2937	0.4569	0.5834	0.340
1.0e-5	0.5185	0.3265	0.5571	0.3110	0.5108	0.6107	0.310
1.0e-6	0.5370	0.3388	0.5571	0.5359	0.5024	0.6043	0.562
1.0e-7	0.5275	0.3265	NA	0.5415	0.5006	0.6002	NA
				AdaBoostM1			
0.15	0.5980	0.5467	0.5017	0.5093	0.6449	0.7263	0.571
0.1	0.6129	0.4463	0.5559	0.4870	0.6557	0.7135	0.549
0.05	0.6057	0.4433	0.4685	0.4808	0.6509	0.7205	0.555
0.01	0.6051	0.4328	0.4557	0.4839	0.6275	0.7019	0.572
0.0010	0.6260	0.4328	0.4312	0.5644	0.6772	0.7316	0.589
1.0e-4	0.6153	0.4363	0.5082	0.5793	0.7066	0.7769	0.606
1.0e-5	0.6093	0.4345	0.5944	0.6029	0.7186	0.7728	0.577
1.0e-6	0.6243	0.4692	0.5979	0.5607	0.7048	0.7711	0.579
1.0e-7	0.6075	0.4381	NA	0.5415	0.6880	0.7763	NA
				C4.5			
0.15	0.4958	0.4345	0.4312	0.4572	0.5737	0.5322	0.520
0.1	0.5119	0.4345	0.4312	0.4572	0.5737	0.5654	0.545
0.05	0.5424	0.4345	0.4312	0.4542	0.5305	0.5660	0.521
0.01	0.5275	0.4345	0.4312	0.5285	0.5934	0.5962	0.454
0.0010	0.5161	0.2595	0.3800	0.2850	0.5503	0.5962	0.471
1.0e-4	0.5956	0.4345	0.5530	0.5397	0.5731	0.6049	0.590
1.0e-5	0.6153	0.4897	0.5979	0.5960	0.6407	0.6374	0.567
1.0e-6	0.6219	0.5596	0.5979	0.5601	0.6246	0.6119	0.580
1.0e-7	0.6063	0.4292	NA	0.5415	0.6377	0.6397	NA
				20RF			
0.15	0.5568	0.5584	0.5629	0.5248	0.5593	0.5683	0.557
0.1	0.5591	0.4850	0.5291	0.5316	0.5563	0.5700	0.560
0.05	0.5579	0.5637	0.5408	0.4876	0.5569	0.5735	0.562
0.01	0.5562	0.5050	0.5670	0.5242	0.5635	0.5712	0.567
0.0010	0.5705	0.4827	0.4965	0.5483	0.5958	0.6496	0.572
1.0e-4	0.5914	0.5261	0.4854	0.5663	0.6443	0.7228	0.599
1.0e-5	0.6159	0.4052	0.5932	0.5917	0.6695	0.7135	0.571
1.0e-6	0.6243	0.4175	0.5979	0.5607	0.6701	0.7269	0.579
1.0e-7	0.6075	0.4292	NA	0.5415	0.6760	0.7461	NA

Table S78: Overall accuracy obtained by all the algorithms under the haplotype-based approach with 2-SNP haplotype length, additive genetic model and holdout sampling. Results for each p-value threshold (column 1) are shown. The maximum p-value threshold used was 0.15.

p-value threshold	BD	CAD	HT	IBD	RA	T1D	T2D
				NBC			
0.15	0.5866	0.4345	0.4312	0.5434	0.5886	0.6229	0.588
0.1	0.5938	0.4345	0.4312	0.5359	0.6006	0.6328	0.591
0.05	0.5974	0.4345	0.4312	0.4895	0.6072	0.6403	0.586
0.01	0.6039	0.4345	0.4312	0.4665	0.6293	0.6810	0.581
0.0010	0.6123	0.4345	0.4312	0.5483	0.6569	0.6868	0.595
1.0e-4	0.6081	0.4416	0.4860	0.5607	0.6581	0.6868	0.594
1.0e-5	0.6022	0.5555	0.5688	0.5651	0.6467	0.6886	0.573
1.0e-6	0.5878	0.5661	0.5688	0.5421	0.6401	0.6886	0.568
1.0e-7	0.5621	0.5655	NA	0.5415	0.6413	0.6938	NA
				sSVM			
0.15	0.5627	0.5655	0.5688	0.5434	0.5617	0.5863	0.577
0.1	0.5711	0.5655	0.5688	0.5458	0.5641	0.5979	0.569
0.05	0.5699	0.5655	0.5688	0.5248	0.5665	0.6049	0.574
0.01	0.5657	0.5655	0.5600	0.4740	0.5790	0.6270	0.561
0.0010	0.5645	0.5602	0.4481	0.5514	0.6078	0.6839	0.564
1.0e-4	0.5842	0.5655	0.5111	0.5390	0.6365	0.7316	0.583
1.0e-5	0.5932	0.4245	0.5688	0.5682	0.6551	0.7490	0.575
1.0e-6	0.5872	0.4809	0.5688	0.5415	0.6503	0.7472	0.565
1.0e-7	0.5621	0.4245	NA	0.5415	0.6533	0.7443	NA
				AdaBoostM1			
0.15	0.5956	0.5643	0.5291	0.5576	0.6162	0.6874	0.580
0.1	0.5992	0.4839	0.5653	0.5229	0.6162	0.6711	0.574
0.05	0.5914	0.4803	0.4837	0.5087	0.6114	0.6682	0.561
0.01	0.5836	0.4422	0.4901	0.5118	0.6102	0.6665	0.574
0.0010	0.6111	0.4322	0.4307	0.5626	0.6419	0.6903	0.593
1.0e-4	0.5938	0.4674	0.5705	0.5607	0.6395	0.7281	0.595
1.0e-5	0.5920	0.4152	0.5688	0.5533	0.6204	0.7350	0.574
1.0e-6	0.5878	0.5678	0.5688	0.5421	0.5946	0.7252	0.565
1.0e-7	0.5621	0.5655	NA	0.5415	0.5850	0.7130	NA
				C4.5			
0.15	0.5962	0.4345	0.4312	0.4963	0.6144	0.6037	0.604
0.1	0.5980	0.4345	0.4312	0.4963	0.6281	0.6444	0.602
0.05	0.6225	0.4345	0.4312	0.4981	0.6263	0.6432	0.590
0.01	0.6069	0.4345	0.4312	0.5421	0.6293	0.6537	0.579
0.0010	0.6129	0.5197	0.4627	0.5211	0.6359	0.6868	0.578
1.0e-4	0.6051	0.4345	0.5670	0.5638	0.6329	0.6862	0.579
1.0e-5	0.5938	0.5684	0.5688	0.5514	0.6204	0.6979	0.576
1.0e-6	0.5878	0.5643	0.5688	0.5415	0.6240	0.6787	0.565
1.0e-7	0.5621	0.5655	NA	0.5415	0.6120	0.6746	NA
				20RF			
0.15	0.5579	0.5655	0.5688	0.5415	0.5569	0.5700	0.565
0.1	0.5579	0.5649	0.5688	0.5415	0.5569	0.5700	0.565
0.05	0.5579	0.5655	0.5688	0.5415	0.5569	0.5700	0.565
0.01	0.5579	0.5655	0.5688	0.5415	0.5569	0.5700	0.565
0.0010	0.5579	0.5655	0.5688	0.5415	0.5569	0.5700	0.565
1.0e-4	0.5597	0.5655	0.5688	0.5415	0.5569	0.5700	0.565
1.0e-5	0.5717	0.5655	0.5693	0.5514	0.5569	0.5752	0.568
1.0e-6	0.5866	0.5666	0.5688	0.5421	0.5563	0.5851	0.565
1.0e-7	0.5621	0.5866	NA	0.5415	0.5569	0.5869	NA

Table S79: Overall accuracy obtained by all the algorithms under the haplotype-based approach with 2-SNP haplotype length, dominant genetic model and holdout sampling. Results for each p-value threshold (column 1) are shown. The maximum p-value threshold used was 0.15.

p-value threshold	BD	CAD	HT	IBD	RA	T1D	T2D
			NBC				
0.15	0.6123	0.4345	0.4312	0.5316	0.6006	0.6072	0.555
0.1	0.5980	0.4345	0.4312	0.5043	0.6096	0.6043	0.559
0.05	0.5944	0.4345	0.4312	0.4653	0.6174	0.6049	0.564
0.01	0.5812	0.4345	0.4312	0.4591	0.6275	0.6194	0.548
0.0010	0.5723	0.4345	0.4312	0.5266	0.6497	0.6101	0.531
1.0e-4	0.5520	0.4345	0.4312	0.5204	0.6401	0.6206	0.504
1.0e-5	0.5532	0.4369	0.4330	0.4876	0.6311	0.6328	0.460
1.0e-6	0.4928	0.4345	0.4312	0.4957	0.6210	0.6357	0.434
1.0e-7	0.4934	0.4345	NA	0.4963	0.6198	0.6397	NA
			sSVM				
0.15	0.5884	0.5655	0.5688	0.5465	0.5695	0.6037	0.540
0.1	0.5729	0.5655	0.5688	0.5527	0.5689	0.5892	0.542
0.05	0.5550	0.5655	0.5688	0.4944	0.5497	0.5869	0.531
0.01	0.5418	0.5473	0.5192	0.4641	0.5611	0.6235	0.504
0.0010	0.5352	0.5185	0.4353	0.5279	0.5988	0.6822	0.507
1.0e-4	0.5484	0.5608	0.4476	0.5149	0.6533	0.7379	0.543
1.0e-5	0.6165	0.4381	0.5979	0.5347	0.6647	0.7443	0.522
1.0e-6	0.6219	0.4310	0.5979	0.5601	0.6653	0.7397	0.580
1.0e-7	0.6063	0.4381	NA	0.5415	0.6515	0.7333	NA
			AdaBoostM1				
0.15	0.5562	0.5232	0.4633	0.4715	0.6216	0.6769	0.535
0.1	0.5568	0.4392	0.5192	0.4740	0.6305	0.6694	0.529
0.05	0.5579	0.4392	0.4411	0.4641	0.6174	0.6705	0.527
0.01	0.5621	0.4345	0.4435	0.4672	0.6024	0.6386	0.528
0.0010	0.5783	0.4345	0.4312	0.5223	0.6503	0.6700	0.541
1.0e-4	0.5442	0.4345	0.4516	0.4944	0.6689	0.6979	0.502
1.0e-5	0.5460	0.4345	0.4639	0.4802	0.6455	0.6880	0.448
1.0e-6	0.4928	0.4334	0.4312	0.4963	0.6299	0.6961	0.437
1.0e-7	0.4934	0.4345	NA	0.4963	0.6156	0.6891	NA
			C4.5				
0.15	0.5490	0.4345	0.4312	0.4585	0.5934	0.5752	0.563
0.1	0.5484	0.4345	0.4312	0.4585	0.5778	0.5747	0.557
0.05	0.5705	0.4345	0.4312	0.4585	0.5760	0.5869	0.547
0.01	0.5777	0.4345	0.4312	0.5415	0.6054	0.5909	0.523
0.0010	0.5639	0.4510	0.4318	0.5006	0.6269	0.6525	0.532
1.0e-4	0.5579	0.4345	0.5589	0.5328	0.6228	0.6328	0.526
1.0e-5	0.5442	0.4404	0.4312	0.4802	0.6659	0.6676	0.443
1.0e-6	0.4421	0.4886	0.4312	0.4585	0.6431	0.6566	0.434
1.0e-7	0.4421	0.4345	NA	0.4585	0.6425	0.6758	NA
			20RF				
0.15	0.4421	0.4328	0.4376	0.4585	0.4431	0.4300	0.434
0.1	0.4421	0.4345	0.4318	0.4585	0.4431	0.4300	0.434
0.05	0.4421	0.4345	0.4312	0.4585	0.4431	0.4300	0.434
0.01	0.4421	0.4345	0.4283	0.4585	0.4431	0.4300	0.434
0.0010	0.4421	0.4345	0.4312	0.4579	0.4431	0.4341	0.434
1.0e-4	0.4421	0.4345	0.4312	0.4591	0.4862	0.4492	0.436
1.0e-5	0.4630	0.4375	0.4406	0.4796	0.5234	0.4846	0.444
1.0e-6	0.4922	0.4345	0.4312	0.4963	0.5425	0.4927	0.437
1.0e-7	0.4934	0.4345	NA	0.4963	0.5599	0.5317	NA

Table S80: Overall accuracy obtained by all the algorithms under the haplotype-based approach with 2-SNP haplotype length, recessive genetic model and holdout sampling. Results for each p-value threshold (column 1) are shown. The maximum p-value threshold used was 0.15.

p-value threshold	BD	CAD	HT	IBD	RA	T1D	T2D
NBC							
0.15	0.6022	0.4345	0.4312	0.4672	0.6150	0.6572	0.575
0.1	0.6057	0.4345	0.4312	0.4740	0.6252	0.6589	0.576
0.05	0.6159	0.4345	0.4312	0.4709	0.6341	0.6729	0.574
0.01	0.6141	0.4345	0.4312	0.4765	0.6593	0.6769	0.575
0.0010	0.6266	0.4345	0.4476	0.5316	0.6635	0.6624	0.582
1.0e-4	0.6225	0.4357	0.4312	0.5682	0.6527	0.6705	0.582
1.0e-5	0.6183	0.5655	0.4073	0.5892	0.6479	0.6752	0.582
1.0e-6	0.6207	0.5901	0.5688	0.5527	0.6395	0.6810	0.560
1.0e-7	0.6039	0.5954	NA	0.5415	0.6269	0.6926	NA
sSVM							
0.15	0.4456	0.5655	0.5688	0.2831	0.4491	0.4573	0.369
0.1	0.4062	0.5655	0.5688	0.3302	0.4240	0.4230	0.343
0.05	0.3871	0.5655	0.5688	0.2825	0.3844	0.4126	0.338
0.01	0.3513	0.2601	0.2366	0.2732	0.3671	0.4062	0.290
0.0010	0.3280	0.2748	0.2576	0.2677	0.3928	0.4945	0.300
1.0e-4	0.3572	0.5437	0.3124	0.3086	0.4563	0.5840	0.328
1.0e-5	0.5299	0.3212	0.3852	0.3879	0.4982	0.6078	0.400
1.0e-6	0.5382	0.2490	0.5624	0.4238	0.4988	0.6055	0.433
1.0e-7	0.5305	0.5655	NA	0.5415	0.4958	0.6037	NA
AdaBoostM1							
0.15	0.6039	0.4386	0.5390	0.5143	0.6515	0.7118	0.551
0.1	0.6093	0.4909	0.4674	0.5291	0.6377	0.7135	0.542
0.05	0.6087	0.4392	0.4470	0.5248	0.6503	0.7089	0.557
0.01	0.5980	0.4563	0.4295	0.5558	0.6168	0.6723	0.539
0.0010	0.6302	0.5590	0.5256	0.5173	0.6515	0.7310	0.574
1.0e-4	0.6231	0.4345	0.4312	0.5750	0.6958	0.7763	0.588
1.0e-5	0.6249	0.5385	0.4120	0.5973	0.6988	0.7780	0.607
1.0e-6	0.6225	0.5520	0.5688	0.5533	0.6928	0.7827	0.560
1.0e-7	0.6039	0.5954	NA	0.5415	0.6772	0.7746	NA
C4.5							
0.15	0.5024	0.4345	0.4312	0.4572	0.5629	0.5160	0.528
0.1	0.5430	0.4345	0.4312	0.4572	0.5826	0.5253	0.501
0.05	0.5585	0.4345	0.4312	0.3742	0.5796	0.5636	0.498
0.01	0.5143	0.4345	0.4312	0.3030	0.5383	0.5404	0.456
0.0010	0.4821	0.2795	0.4848	0.4548	0.5383	0.5683	0.473
1.0e-4	0.5693	0.4339	0.3054	0.5452	0.5850	0.6066	0.564
1.0e-5	0.6171	0.4063	0.4301	0.5911	0.6102	0.6444	0.596
1.0e-6	0.6201	0.5032	0.5688	0.5533	0.6419	0.6554	0.547
1.0e-7	0.6027	0.5655	NA	0.5415	0.6323	0.6641	NA
20RF							
0.15	0.5556	0.5502	0.5565	0.5415	0.5515	0.5694	0.561
0.1	0.5568	0.4944	0.4860	0.5322	0.5575	0.5700	0.562
0.05	0.5568	0.5473	0.5490	0.5428	0.5569	0.5677	0.565
0.01	0.5609	0.5526	0.5577	0.5266	0.5569	0.5822	0.571
0.0010	0.5842	0.5185	0.5420	0.5415	0.5970	0.6612	0.581
1.0e-4	0.5974	0.4457	0.5216	0.5793	0.6407	0.7193	0.611
1.0e-5	0.6135	0.4422	0.4079	0.5855	0.6707	0.7252	0.592
1.0e-6	0.6219	0.4334	0.5862	0.5527	0.6701	0.7182	0.560
1.0e-7	0.6039	0.5684	NA	0.5415	0.6713	0.7275	NA

Table S81: Overall accuracy obtained by all the algorithms under the haplotype-based approach with 3-SNP haplotype length, additive genetic model and holdout sampling. Results for each p-value threshold (column 1) are shown. The maximum p-value threshold used was 0.15.

p-value threshold	BD	CAD	HT	IBD	RA	T1D	T2D
			NBC				
0.15	0.5842	0.4345	0.4312	0.4765	0.5934	0.6368	0.589
0.1	0.5974	0.4345	0.4312	0.4994	0.6006	0.6450	0.585
0.05	0.6045	0.4345	0.4312	0.5006	0.6168	0.6624	0.582
0.01	0.6033	0.4345	0.4312	0.5093	0.6371	0.6851	0.583
0.0010	0.6129	0.4363	0.4790	0.5397	0.6569	0.6851	0.597
1.0e-4	0.6135	0.5062	0.4295	0.5651	0.6635	0.6891	0.597
1.0e-5	0.6063	0.5655	0.5688	0.5657	0.6509	0.6915	0.579
1.0e-6	0.5914	0.5655	0.5688	0.5458	0.6437	0.6949	0.565
1.0e-7	0.5615	0.5655	NA	0.5415	0.6353	0.6955	NA
			sSVM				
0.15	0.5651	0.5655	0.5688	0.5452	0.5641	0.5927	0.564
0.1	0.5699	0.5655	0.5688	0.5434	0.5701	0.6049	0.569
0.05	0.5729	0.5655	0.5688	0.5390	0.5784	0.6072	0.561
0.01	0.5609	0.5344	0.5181	0.5465	0.5802	0.6084	0.553
0.0010	0.5747	0.5156	0.5256	0.5335	0.6108	0.6862	0.568
1.0e-4	0.5980	0.5661	0.5437	0.5613	0.6431	0.7298	0.579
1.0e-5	0.5944	0.4821	0.4068	0.5626	0.6503	0.7438	0.581
1.0e-6	0.5890	0.5120	0.5688	0.5489	0.6503	0.7420	0.562
1.0e-7	0.5615	0.5655	NA	0.5415	0.6551	0.7443	NA
			AdaBoostM1				
0.15	0.5914	0.4721	0.5583	0.5390	0.6150	0.6787	0.555
0.1	0.5980	0.5355	0.5175	0.5458	0.6174	0.6734	0.559
0.05	0.5896	0.4639	0.4913	0.5496	0.6275	0.6775	0.561
0.01	0.5914	0.4903	0.4464	0.5483	0.5886	0.6508	0.552
0.0010	0.6057	0.5684	0.5554	0.5310	0.6263	0.6862	0.582
1.0e-4	0.6057	0.4292	0.4225	0.5663	0.6377	0.7211	0.583
1.0e-5	0.5992	0.5702	0.5688	0.5514	0.5994	0.7228	0.570
1.0e-6	0.5884	0.5655	0.5688	0.5458	0.5850	0.7217	0.565
1.0e-7	0.5615	0.5655	NA	0.5415	0.5659	0.7135	NA
			C4.5				
0.15	0.6004	0.4345	0.4312	0.4963	0.6138	0.6066	0.571
0.1	0.6129	0.4345	0.4312	0.4963	0.5928	0.6188	0.557
0.05	0.6165	0.4345	0.4312	0.5043	0.6317	0.6339	0.592
0.01	0.5956	0.4345	0.4312	0.5335	0.6210	0.6467	0.560
0.0010	0.5938	0.5056	0.5367	0.5409	0.6174	0.6554	0.598
1.0e-4	0.6063	0.4339	0.5396	0.5477	0.6216	0.6880	0.584
1.0e-5	0.5968	0.4821	0.3945	0.5520	0.6246	0.6886	0.578
1.0e-6	0.5866	0.5672	0.5688	0.5458	0.6246	0.6822	0.565
1.0e-7	0.5615	0.5655	NA	0.5415	0.6054	0.6781	NA
			20RF				
0.15	0.5579	0.5655	0.5688	0.5415	0.5569	0.5700	0.565
0.1	0.5579	0.5655	0.5682	0.5415	0.5569	0.5700	0.565
0.05	0.5579	0.5655	0.5688	0.5415	0.5569	0.5700	0.565
0.01	0.5579	0.5655	0.5688	0.5415	0.5569	0.5700	0.565
0.0010	0.5579	0.5655	0.5688	0.5415	0.5569	0.5700	0.565
1.0e-4	0.5615	0.5655	0.5688	0.5421	0.5569	0.5723	0.565
1.0e-5	0.5777	0.5655	0.5705	0.5471	0.5569	0.5741	0.566
1.0e-6	0.5842	0.5150	0.5688	0.5458	0.5569	0.5834	0.565
1.0e-7	0.5615	0.5655	NA	0.5415	0.5569	0.5857	NA

Table S82: Overall accuracy obtained by all the algorithms under the haplotype-based approach with 3-SNP haplotype length, dominant genetic model and holdout sampling. Results for each p-value threshold (column 1) are shown. The maximum p-value threshold used was 0.15.

p-value threshold	BD	CAD	HT	IBD	RA	T1D	T2D
NBC							
0.15	0.5980	0.4345	0.4312	0.4616	0.6228	0.6217	0.547
0.1	0.6039	0.4345	0.4312	0.4653	0.6263	0.6142	0.545
0.05	0.5908	0.4345	0.4312	0.4622	0.6287	0.6182	0.549
0.01	0.5938	0.4345	0.4312	0.4665	0.6269	0.6188	0.535
0.0010	0.5974	0.4345	0.4307	0.5062	0.6293	0.6072	0.532
1.0e-4	0.5729	0.4351	0.4312	0.5229	0.6359	0.6322	0.515
1.0e-5	0.5454	0.4698	0.4312	0.4851	0.6246	0.6316	0.492
1.0e-6	0.4928	0.4469	0.4312	0.4876	0.6228	0.6339	0.444
1.0e-7	0.4934	0.4345	NA	0.4963	0.6216	0.6421	NA
sSVM							
0.15	0.5771	0.5655	0.5688	0.5025	0.5826	0.6089	0.528
0.1	0.5663	0.5655	0.5688	0.5260	0.5850	0.5956	0.527
0.05	0.5633	0.5655	0.5688	0.5136	0.5629	0.6008	0.537
0.01	0.5568	0.4721	0.4610	0.4988	0.5713	0.6206	0.497
0.0010	0.5400	0.4504	0.4510	0.4876	0.5922	0.6729	0.512
1.0e-4	0.5520	0.5760	0.4843	0.5384	0.6281	0.7397	0.535
1.0e-5	0.6195	0.4345	0.4312	0.5644	0.6515	0.7501	0.577
1.0e-6	0.6201	0.4392	0.5897	0.5446	0.6527	0.7432	0.542
1.0e-7	0.6027	0.5655	NA	0.5415	0.6491	0.7356	NA
AdaBoostM1							
0.15	0.5591	0.4322	0.5122	0.4851	0.6084	0.6461	0.507
0.1	0.5789	0.4568	0.4534	0.4981	0.6036	0.6589	0.521
0.05	0.5490	0.4410	0.4388	0.4802	0.6293	0.6607	0.542
0.01	0.5490	0.4474	0.4312	0.5112	0.6084	0.6310	0.510
0.0010	0.5795	0.4774	0.4784	0.4740	0.6269	0.6479	0.517
1.0e-4	0.5478	0.4345	0.4312	0.5006	0.6449	0.6961	0.500
1.0e-5	0.5102	0.4357	0.4312	0.4796	0.6323	0.6868	0.474
1.0e-6	0.4928	0.4422	0.4312	0.4882	0.6174	0.6973	0.451
1.0e-7	0.4934	0.4345	NA	0.4963	0.5934	0.6711	NA
C4.5							
0.15	0.5466	0.4345	0.4312	0.4585	0.5784	0.5456	0.530
0.1	0.5920	0.4345	0.4312	0.4585	0.6060	0.5840	0.531
0.05	0.5890	0.4345	0.4312	0.4641	0.5970	0.5857	0.534
0.01	0.5693	0.4345	0.4312	0.4740	0.5964	0.5840	0.530
0.0010	0.5657	0.4445	0.4726	0.4944	0.6090	0.6299	0.522
1.0e-4	0.5400	0.4345	0.4668	0.5235	0.6383	0.6368	0.515
1.0e-5	0.5352	0.4363	0.4312	0.4796	0.6485	0.6938	0.473
1.0e-6	0.4421	0.4345	0.4312	0.4882	0.6521	0.6845	0.448
1.0e-7	0.4421	0.4345	NA	0.4585	0.6413	0.6752	NA
20RF							
0.15	0.4421	0.4334	0.4307	0.4585	0.4431	0.4300	0.434
0.1	0.4421	0.4345	0.4312	0.4585	0.4431	0.4300	0.434
0.05	0.4421	0.4363	0.4336	0.4585	0.4431	0.4300	0.434
0.01	0.4421	0.4369	0.4353	0.4585	0.4431	0.4300	0.434
0.0010	0.4421	0.4345	0.4312	0.4585	0.4437	0.4317	0.434
1.0e-4	0.4421	0.4345	0.4295	0.4591	0.4778	0.4631	0.441
1.0e-5	0.4462	0.4345	0.4312	0.4789	0.5305	0.4759	0.453
1.0e-6	0.4922	0.4351	0.4312	0.4882	0.5389	0.4985	0.454
1.0e-7	0.4934	0.4345	NA	0.4963	0.5551	0.4997	NA

Table S83: Overall accuracy obtained by all the algorithms under the haplotype-based approach with 3-SNP haplotype length, recessive genetic model and holdout sampling. Results for each p-value threshold (column 1) are shown. The maximum p-value threshold used was 0.15.

p-value threshold	BD	CAD	HT	IBD	RA	T1D	T2D
				NBC			
0.15	0.6016	0.4345	0.4312	0.5235	0.6216	0.6595	0.585
0.1	0.6039	0.4345	0.4312	0.5143	0.6198	0.6694	0.586
0.05	0.6045	0.4345	0.4312	0.5415	0.6329	0.6851	0.585
0.01	0.6033	0.4345	0.4324	0.4796	0.6491	0.6769	0.572
0.0010	0.5998	0.4381	0.4272	0.5112	0.6533	0.6659	0.587
1.0e-4	0.6153	0.5655	0.4540	0.5266	0.6467	0.6641	0.566
1.0e-5	0.6075	0.5666	0.5688	0.5564	0.6377	0.6764	0.575
1.0e-6	0.6105	0.5655	NA	0.5415	0.6353	0.6752	0.548
1.0e-7	0.5759	0.5655	NA	0.5415	0.6431	0.6769	NA
				sSVM			
0.15	0.4367	0.5655	0.5688	0.3420	0.4521	0.4596	0.381
0.1	0.3931	0.5655	0.5688	0.3414	0.4138	0.4387	0.361
0.05	0.3841	0.5655	0.5688	0.3556	0.3892	0.4195	0.337
0.01	0.3524	0.2695	0.4878	0.2980	0.3599	0.4201	0.313
0.0010	0.3262	0.2819	0.3042	0.2571	0.3850	0.4852	0.286
1.0e-4	0.3596	0.5062	0.2430	0.2565	0.4521	0.5764	0.318
1.0e-5	0.4958	0.4228	0.5390	0.3129	0.4790	0.6089	0.314
1.0e-6	0.5370	0.4222	NA	0.4263	0.4982	0.6020	0.296
1.0e-7	0.5436	0.5655	NA	0.5415	0.5060	0.6049	NA
				AdaBoostM1			
0.15	0.5920	0.5649	0.4598	0.5043	0.6479	0.7379	0.562
0.1	0.6057	0.4357	0.5484	0.5458	0.6455	0.7217	0.557
0.05	0.5890	0.4492	0.5385	0.5217	0.6473	0.7199	0.554
0.01	0.5908	0.4375	0.5612	0.5012	0.6287	0.6903	0.553
0.0010	0.6219	0.5373	0.5548	0.4709	0.6359	0.7130	0.573
1.0e-4	0.6159	0.5655	0.4639	0.5217	0.6928	0.7629	0.564
1.0e-5	0.6111	0.5655	0.5676	0.5824	0.6832	0.7775	0.585
1.0e-6	0.6105	0.5655	NA	0.5415	0.6958	0.7780	0.548
1.0e-7	0.5759	0.5655	NA	0.5415	0.6874	0.7682	NA
				C4.5			
0.15	0.5114	0.4345	0.4312	0.4585	0.5467	0.5160	0.468
0.1	0.5466	0.4345	0.4312	0.4585	0.5449	0.5195	0.464
0.05	0.5747	0.4345	0.4312	0.4021	0.5222	0.5311	0.487
0.01	0.5102	0.4345	0.4301	0.3327	0.5323	0.5619	0.456
0.0010	0.4767	0.3136	0.3059	0.4727	0.5228	0.5479	0.438
1.0e-4	0.5783	0.4921	0.3805	0.5248	0.5509	0.6026	0.533
1.0e-5	0.6147	0.5643	0.5740	0.5607	0.5844	0.6078	0.579
1.0e-6	0.6081	0.5496	NA	0.5415	0.5910	0.6490	0.554
1.0e-7	0.5759	0.5684	NA	0.5415	0.6060	0.6275	NA
				20RF			
0.15	0.5597	0.5555	0.5600	0.5397	0.5581	0.5689	0.557
0.1	0.5568	0.5484	0.5175	0.5155	0.5581	0.5729	0.562
0.05	0.5603	0.5508	0.5536	0.5489	0.5629	0.5671	0.554
0.01	0.5532	0.5649	0.5519	0.5514	0.5617	0.5857	0.565
0.0010	0.5741	0.4375	0.5105	0.5502	0.5856	0.6595	0.574
1.0e-4	0.6033	0.5690	0.5152	0.5198	0.6479	0.7159	0.576
1.0e-5	0.6189	0.5666	0.5688	0.5824	0.6533	0.7199	0.586
1.0e-6	0.6105	0.5420	NA	0.5415	0.6659	0.7310	0.547
1.0e-7	0.5759	0.5132	NA	0.5415	0.6725	0.7257	NA

Table S84: Overall accuracy obtained by all the algorithms under the haplotype-based approach with 4-SNP haplotype length, additive genetic model and holdout sampling. Results for each p-value threshold (column 1) are shown. The maximum p-value threshold used was 0.15.

p-value threshold	BD	CAD	HT	IBD	RA	T1D	T2D
				NBC			
0.15	0.5896	0.4345	0.4312	0.5428	0.5970	0.6392	0.585
0.1	0.5836	0.4345	0.4312	0.5409	0.6018	0.6432	0.582
0.05	0.6027	0.4345	0.4312	0.5415	0.6168	0.6705	0.587
0.01	0.6039	0.4345	0.4295	0.5006	0.6407	0.6920	0.582
0.0010	0.5950	0.4839	0.4388	0.5316	0.6575	0.6769	0.590
1.0e-4	0.6022	0.5655	0.5478	0.5539	0.6515	0.6909	0.580
1.0e-5	0.5896	0.5655	0.5688	0.5520	0.6407	0.6880	0.578
1.0e-6	0.5741	0.5655	NA	0.5415	0.6413	0.6856	0.565
1.0e-7	0.5579	0.5655	NA	0.5415	0.6389	0.6856	NA
				sSVM			
0.15	0.5645	0.5655	0.5688	0.5477	0.5689	0.5915	0.578
0.1	0.5645	0.5655	0.5688	0.5428	0.5766	0.6037	0.579
0.05	0.5717	0.5655	0.5688	0.5428	0.5737	0.6124	0.571
0.01	0.5687	0.4850	0.5664	0.5378	0.5743	0.6258	0.558
0.0010	0.5657	0.5537	0.5577	0.5217	0.5928	0.6787	0.556
1.0e-4	0.5902	0.5672	0.4965	0.5440	0.6401	0.7205	0.564
1.0e-5	0.5884	0.5690	0.5693	0.5551	0.6485	0.7339	0.568
1.0e-6	0.5741	0.5690	NA	0.5465	0.6653	0.7408	0.554
1.0e-7	0.5579	0.5655	NA	0.5415	0.6665	0.7408	NA
				AdaBoostM1			
0.15	0.5848	0.5672	0.5128	0.5242	0.6072	0.6886	0.577
0.1	0.6027	0.4451	0.5635	0.5582	0.6132	0.6781	0.566
0.05	0.5890	0.4797	0.5594	0.5428	0.6072	0.6862	0.578
0.01	0.5777	0.4445	0.5653	0.5403	0.6024	0.6549	0.582
0.0010	0.5962	0.5578	0.5734	0.4950	0.6108	0.6775	0.584
1.0e-4	0.5998	0.5655	0.5262	0.5514	0.6192	0.7118	0.579
1.0e-5	0.5878	0.5655	0.5688	0.5421	0.6096	0.6984	0.568
1.0e-6	0.5741	0.5655	NA	0.5415	0.5802	0.6996	0.565
1.0e-7	0.5579	0.5655	NA	0.5415	0.5665	0.7031	NA
				C4.5			
0.15	0.6069	0.4345	0.4312	0.4981	0.6174	0.6136	0.561
0.1	0.6171	0.4345	0.4312	0.5006	0.6341	0.6026	0.584
0.05	0.6189	0.4345	0.4312	0.5019	0.6102	0.6206	0.578
0.01	0.6075	0.4345	0.4312	0.5217	0.6120	0.6339	0.572
0.0010	0.6004	0.5484	0.5647	0.4963	0.6120	0.6490	0.569
1.0e-4	0.5842	0.5684	0.5047	0.5384	0.6246	0.6601	0.565
1.0e-5	0.5866	0.5661	0.5688	0.5415	0.6269	0.6781	0.574
1.0e-6	0.5741	0.5666	NA	0.5415	0.6383	0.6874	0.565
1.0e-7	0.5579	0.5655	NA	0.5415	0.6072	0.6897	NA
				20RF			
0.15	0.5579	0.5655	0.5688	0.5415	0.5569	0.5700	0.565
0.1	0.5579	0.5655	0.5688	0.5415	0.5569	0.5700	0.565
0.05	0.5579	0.5655	0.5688	0.5415	0.5569	0.5700	0.565
0.01	0.5579	0.5655	0.5688	0.5415	0.5569	0.5700	0.565
0.0010	0.5579	0.5655	0.5688	0.5415	0.5569	0.5700	0.565
1.0e-4	0.5579	0.5655	0.5688	0.5403	0.5569	0.5706	0.565
1.0e-5	0.5741	0.5655	0.5688	0.5421	0.5569	0.5735	0.565
1.0e-6	0.5741	0.5649	NA	0.5415	0.5569	0.5828	0.565
1.0e-7	0.5579	0.5678	NA	0.5415	0.5569	0.5845	NA

Table S85: Overall accuracy obtained by all the algorithms under the haplotype-based approach with 4-SNP haplotype length, dominant genetic model and holdout sampling. Results for each p-value threshold (column 1) are shown. The maximum p-value threshold used was 0.15.

p-value threshold	BD	CAD	HT	IBD	RA	T1D	T2D
				NBC			
0.15	0.6045	0.4345	0.4312	0.4851	0.6263	0.6200	0.549
0.1	0.5974	0.4345	0.4312	0.4796	0.6144	0.6182	0.563
0.05	0.5914	0.4345	0.4312	0.5043	0.6150	0.6357	0.548
0.01	0.5729	0.4345	0.4312	0.4585	0.6186	0.6252	0.533
0.0010	0.5609	0.4345	0.4307	0.4820	0.6234	0.6194	0.539
1.0e-4	0.5585	0.5637	0.4307	0.4802	0.6228	0.6241	0.490
1.0e-5	0.5364	0.5484	0.5851	0.4827	0.6186	0.6264	0.497
1.0e-6	0.4928	0.5643	NA	0.4901	0.6228	0.6328	0.454
1.0e-7	0.4940	0.5919	NA	0.4981	0.6168	0.6328	NA
				sSVM			
0.15	0.5848	0.5655	0.5688	0.5409	0.5880	0.6177	0.549
0.1	0.5759	0.5655	0.5688	0.5291	0.5737	0.6194	0.554
0.05	0.5621	0.5666	0.5688	0.5186	0.5731	0.6136	0.538
0.01	0.5424	0.4404	0.5449	0.5192	0.5593	0.6264	0.517
0.0010	0.5311	0.4839	0.4983	0.4969	0.5886	0.6787	0.506
1.0e-4	0.5663	0.5637	0.4417	0.5062	0.6341	0.7374	0.522
1.0e-5	0.6069	0.5167	0.5991	0.5353	0.6425	0.7513	0.522
1.0e-6	0.6081	0.5167	NA	0.5335	0.6431	0.7362	0.508
1.0e-7	0.5759	0.5731	NA	0.5415	0.6449	0.7350	NA
				AdaBoostM1			
0.15	0.5382	0.5531	0.4557	0.4783	0.6216	0.6769	0.521
0.1	0.5400	0.4345	0.4854	0.5074	0.6186	0.6618	0.525
0.05	0.5502	0.4404	0.5035	0.4882	0.6162	0.6554	0.525
0.01	0.5454	0.4334	0.5280	0.4721	0.5898	0.6293	0.541
0.0010	0.5717	0.4979	0.5029	0.4603	0.6060	0.6304	0.531
1.0e-4	0.5430	0.5702	0.4394	0.4752	0.6455	0.6851	0.497
1.0e-5	0.5245	0.5690	0.5787	0.4827	0.6377	0.6973	0.478
1.0e-6	0.4934	0.5713	NA	0.4901	0.6066	0.6990	0.454
1.0e-7	0.4940	0.6048	NA	0.4981	0.6000	0.6822	NA
				C4.5			
0.15	0.5699	0.4345	0.4312	0.4585	0.5713	0.5555	0.511
0.1	0.5854	0.4345	0.4312	0.4585	0.5898	0.5503	0.529
0.05	0.5675	0.4345	0.4312	0.4622	0.5647	0.5944	0.545
0.01	0.5812	0.4345	0.4312	0.4703	0.6102	0.6270	0.528
0.0010	0.5556	0.5073	0.5006	0.4622	0.5862	0.6078	0.511
1.0e-4	0.5693	0.5473	0.4429	0.4833	0.6198	0.6380	0.508
1.0e-5	0.5269	0.5344	0.4312	0.4827	0.6204	0.6566	0.479
1.0e-6	0.4421	0.4339	NA	0.4901	0.6150	0.6688	0.458
1.0e-7	0.4421	0.4345	NA	0.4585	0.6263	0.6589	NA
				20RF			
0.15	0.4421	0.4345	0.4324	0.4585	0.4431	0.4300	0.434
0.1	0.4421	0.4357	0.4307	0.4585	0.4431	0.4300	0.434
0.05	0.4421	0.4363	0.4318	0.4585	0.4431	0.4300	0.434
0.01	0.4421	0.4345	0.4353	0.4585	0.4431	0.4300	0.434
0.0010	0.4421	0.4345	0.4312	0.4585	0.4431	0.4381	0.434
1.0e-4	0.4433	0.4369	0.4312	0.4591	0.4766	0.4648	0.438
1.0e-5	0.4462	0.4351	0.6014	0.4808	0.5174	0.4770	0.454
1.0e-6	0.4922	0.4662	NA	0.4901	0.5347	0.5061	0.454
1.0e-7	0.4940	0.4281	NA	0.4981	0.5461	0.5247	NA

Table S86: Overall accuracy obtained by all the algorithms under the haplotype-based approach with 4-SNP haplotype length, recessive genetic model and holdout sampling. Results for each p-value threshold (column 1) are shown. The maximum p-value threshold used was 0.15.

p-value threshold	BD	CAD	HT	IBD	RA	T1D	T2D
				NBC			
0.15	0.5932	0.4345	0.4312	0.5465	0.6275	0.6641	0.591
0.1	0.6117	0.4345	0.4312	0.5582	0.6383	0.6717	0.594
0.05	0.5944	0.4345	0.4312	0.5093	0.6455	0.6787	0.576
0.01	0.5998	0.4369	0.4312	0.4845	0.6575	0.6827	0.578
0.0010	0.5998	0.4345	0.4312	0.5384	0.6419	0.6717	0.580
1.0e-4	0.6171	0.4339	0.4411	0.5514	0.6419	0.6688	0.562
1.0e-5	0.6344	0.5631	0.5688	0.5539	0.6371	0.6653	0.575
1.0e-6	0.6201	0.5661	NA	0.5421	0.6269	0.6769	0.565
1.0e-7	0.5902	0.5655	NA	0.5415	0.6401	0.6810	NA
				sSVM			
0.15	0.4319	0.5655	0.5688	0.4195	0.4425	0.4573	0.380
0.1	0.3978	0.5655	0.5688	0.4628	0.4180	0.4375	0.364
0.05	0.3740	0.5655	0.5688	0.3804	0.3880	0.4067	0.329
0.01	0.3459	0.2560	0.2786	0.3061	0.3653	0.4160	0.312
0.0010	0.3292	0.3359	0.4557	0.2757	0.3928	0.4823	0.304
1.0e-4	0.3632	0.2590	0.5530	0.3228	0.4359	0.5648	0.296
1.0e-5	0.5281	0.5655	0.5688	0.3055	0.4826	0.5985	0.356
1.0e-6	0.5412	0.5655	NA	0.3922	0.4760	0.6037	0.565
1.0e-7	0.5412	0.5655	NA	0.5415	0.4856	0.6008	NA
				AdaBoostM1			
0.15	0.5956	0.4909	0.4505	0.5130	0.6317	0.7176	0.548
0.1	0.5812	0.5373	0.5169	0.5682	0.6305	0.7223	0.568
0.05	0.5836	0.4651	0.4895	0.5217	0.6329	0.7164	0.546
0.01	0.5795	0.4463	0.4563	0.5037	0.6024	0.6827	0.550
0.0010	0.6117	0.4557	0.4324	0.5130	0.6240	0.6862	0.574
1.0e-4	0.6195	0.5302	0.4330	0.5452	0.6701	0.7472	0.579
1.0e-5	0.6338	0.5161	0.5688	0.5787	0.6754	0.7565	0.575
1.0e-6	0.6195	0.5420	NA	0.5421	0.6814	0.7577	0.565
1.0e-7	0.5902	0.5655	NA	0.5415	0.6772	0.7571	NA
				C4.5			
0.15	0.5030	0.4345	0.4312	0.4523	0.4611	0.5439	0.472
0.1	0.4642	0.4345	0.4312	0.3426	0.5216	0.5450	0.468
0.05	0.5382	0.4345	0.4312	0.3556	0.5078	0.5549	0.478
0.01	0.5048	0.4345	0.4312	0.4572	0.5299	0.5270	0.419
0.0010	0.4809	0.4310	0.4301	0.4095	0.5174	0.5322	0.451
1.0e-4	0.5448	0.4322	0.5117	0.4919	0.5275	0.5892	0.533
1.0e-5	0.6213	0.5702	0.5688	0.5682	0.5425	0.6124	0.559
1.0e-6	0.6189	0.5655	NA	0.5421	0.5347	0.6031	0.565
1.0e-7	0.5902	0.5655	NA	0.5415	0.5922	0.5904	NA
				20RF			
0.15	0.5544	0.5496	0.5548	0.5279	0.5545	0.5723	0.559
0.1	0.5556	0.5555	0.5548	0.5397	0.5521	0.5700	0.567
0.05	0.5591	0.5508	0.5600	0.5366	0.5551	0.5712	0.564
0.01	0.5591	0.4950	0.5227	0.5242	0.5599	0.5863	0.557
0.0010	0.5681	0.4539	0.5152	0.5291	0.5964	0.6833	0.565
1.0e-4	0.6069	0.5649	0.5350	0.5539	0.6329	0.7089	0.567
1.0e-5	0.6207	0.5825	0.5688	0.5793	0.6515	0.7164	0.579
1.0e-6	0.6201	0.5696	NA	0.5421	0.6509	0.7182	0.565
1.0e-7	0.5902	0.5672	NA	0.5415	0.6647	0.7170	NA

Table S87: Overall accuracy obtained by all the algorithms under the haplotype-based approach with 5-SNP haplotype length, additive genetic model and holdout sampling. Results for each p-value threshold (column 1) are shown. The maximum p-value threshold used was 0.15.

p-value threshold	BD	CAD	HT	IBD	RA	T1D	T2D
			NBC				
0.15	0.5824	0.4345	0.4312	0.5582	0.6102	0.6438	0.589
0.1	0.5872	0.4345	0.4312	0.5514	0.6138	0.6485	0.587
0.05	0.5860	0.4322	0.4312	0.5310	0.6204	0.6671	0.570
0.01	0.5986	0.4339	0.4312	0.5260	0.6413	0.7031	0.570
0.0010	0.5944	0.4345	0.4336	0.5564	0.6545	0.6822	0.587
1.0e-4	0.6010	0.4363	0.5758	0.5434	0.6431	0.6862	0.569
1.0e-5	0.5944	0.5655	0.5688	0.5458	0.6347	0.6856	0.575
1.0e-6	0.5765	0.5655	NA	0.5415	0.6365	0.6851	0.565
1.0e-7	0.5627	0.5655	NA	0.5415	0.6317	0.6932	NA
			sSVM				
0.15	0.5651	0.5655	0.5688	0.5465	0.5731	0.5967	0.574
0.1	0.5657	0.5655	0.5688	0.5440	0.5790	0.6072	0.575
0.05	0.5681	0.5655	0.5688	0.5446	0.5760	0.6078	0.565
0.01	0.5693	0.5408	0.5589	0.5446	0.5778	0.6171	0.561
0.0010	0.5645	0.4651	0.5693	0.5496	0.5934	0.6781	0.570
1.0e-4	0.5765	0.5338	0.5693	0.5465	0.6102	0.7176	0.565
1.0e-5	0.5896	0.5655	0.5688	0.5489	0.6377	0.7316	0.574
1.0e-6	0.5765	0.5655	NA	0.5421	0.6359	0.7310	0.565
1.0e-7	0.5627	0.5655	NA	0.5415	0.6329	0.7298	NA
			AdaBoostM1				
0.15	0.5866	0.5414	0.5000	0.5372	0.5976	0.6764	0.561
0.1	0.5800	0.5625	0.5490	0.5582	0.6042	0.6793	0.577
0.05	0.5920	0.5032	0.5239	0.5483	0.6096	0.6711	0.558
0.01	0.5735	0.4651	0.4860	0.5452	0.5868	0.6415	0.568
0.0010	0.6004	0.4962	0.4406	0.5434	0.6066	0.6734	0.582
1.0e-4	0.6033	0.5790	0.4324	0.5384	0.5988	0.7002	0.582
1.0e-5	0.5896	0.5696	0.5688	0.5421	0.5946	0.6955	0.573
1.0e-6	0.5765	0.5696	NA	0.5415	0.5760	0.6984	0.565
1.0e-7	0.5627	0.5655	NA	0.5415	0.5647	0.6949	NA
			C4.5				
0.15	0.6027	0.4345	0.4312	0.4988	0.5784	0.6339	0.567
0.1	0.5998	0.4345	0.4312	0.5198	0.6108	0.6304	0.563
0.05	0.6189	0.4345	0.4312	0.5279	0.6072	0.6386	0.577
0.01	0.6159	0.4345	0.4330	0.4944	0.6090	0.6374	0.552
0.0010	0.5842	0.4345	0.4318	0.5316	0.5802	0.6554	0.561
1.0e-4	0.5818	0.4334	0.5606	0.5372	0.5964	0.6717	0.569
1.0e-5	0.5890	0.5655	0.5688	0.5409	0.5952	0.6787	0.578
1.0e-6	0.5765	0.5655	NA	0.5415	0.6030	0.6758	0.565
1.0e-7	0.5627	0.5655	NA	0.5415	0.6060	0.6740	NA
			20RF				
0.15	0.5579	0.5655	0.5688	0.5415	0.5569	0.5700	0.565
0.1	0.5579	0.5655	0.5688	0.5415	0.5569	0.5700	0.565
0.05	0.5579	0.5655	0.5688	0.5415	0.5569	0.5700	0.565
0.01	0.5579	0.5649	0.5688	0.5415	0.5569	0.5700	0.565
0.0010	0.5579	0.5655	0.5688	0.5415	0.5569	0.5700	0.565
1.0e-4	0.5579	0.5655	0.5688	0.5415	0.5569	0.5700	0.565
1.0e-5	0.5729	0.5655	0.5688	0.5397	0.5569	0.5729	0.566
1.0e-6	0.5765	0.5655	NA	0.5415	0.5569	0.5787	0.565
1.0e-7	0.5627	0.5655	NA	0.5415	0.5569	0.5834	NA

Table S88: Overall accuracy obtained by all the algorithms under the haplotype-based approach with 5-SNP haplotype length, dominant genetic model and holdout sampling. Results for each p-value threshold (column 1) are shown. The maximum p-value threshold used was 0.15.

p-value threshold	BD	CAD	HT	IBD	RA	T1D	T2D
			NBC				
0.15	0.5896	0.4345	0.4312	0.5211	0.6150	0.6351	0.550
0.1	0.5944	0.4345	0.4312	0.5452	0.6138	0.6351	0.542
0.05	0.5753	0.4345	0.4312	0.4864	0.6144	0.6334	0.543
0.01	0.5544	0.4351	0.4312	0.4672	0.6234	0.6246	0.525
0.0010	0.5550	0.4345	0.4312	0.4950	0.6228	0.6136	0.522
1.0e-4	0.5329	0.4345	0.4307	0.5235	0.6198	0.6235	0.487
1.0e-5	0.5161	0.4662	0.4312	0.4888	0.6186	0.6223	0.474
1.0e-6	0.4922	0.4698	NA	0.4845	0.6156	0.6316	0.434
1.0e-7	0.4934	0.4345	NA	0.4963	0.6216	0.6316	NA
			sSVM				
0.15	0.5783	0.5655	0.5688	0.5415	0.5850	0.6223	0.552
0.1	0.5818	0.5655	0.5688	0.5508	0.5904	0.6165	0.545
0.05	0.5484	0.5655	0.5688	0.5409	0.5868	0.5933	0.517
0.01	0.5376	0.4739	0.4936	0.5143	0.5677	0.6304	0.512
0.0010	0.5293	0.4422	0.5321	0.5012	0.6024	0.6694	0.515
1.0e-4	0.5573	0.4498	0.5798	0.5260	0.6365	0.7223	0.508
1.0e-5	0.6195	0.5655	0.5688	0.5335	0.6449	0.7385	0.537
1.0e-6	0.6189	0.5655	NA	0.5304	0.6413	0.7420	0.565
1.0e-7	0.5902	0.5655	NA	0.5415	0.6425	0.7316	NA
			AdaBoostM1				
0.15	0.5478	0.4504	0.4347	0.4715	0.5982	0.6676	0.522
0.1	0.5484	0.4909	0.4662	0.5304	0.6000	0.6589	0.514
0.05	0.5466	0.4422	0.4645	0.4796	0.5838	0.6485	0.511
0.01	0.5239	0.4386	0.4400	0.4696	0.5707	0.6206	0.520
0.0010	0.5609	0.4504	0.4312	0.4907	0.6000	0.6119	0.526
1.0e-4	0.5257	0.4850	0.4307	0.5087	0.6275	0.6740	0.483
1.0e-5	0.5096	0.5167	0.5682	0.4789	0.6138	0.6874	0.468
1.0e-6	0.4928	0.5167	NA	0.4845	0.5916	0.6839	0.474
1.0e-7	0.4934	0.4345	NA	0.4963	0.5970	0.6694	NA
			C4.5				
0.15	0.5520	0.4345	0.4312	0.4591	0.5539	0.5822	0.526
0.1	0.5466	0.4345	0.4312	0.5025	0.5826	0.5880	0.529
0.05	0.5573	0.4345	0.4312	0.4746	0.5922	0.5718	0.525
0.01	0.5687	0.4345	0.4312	0.4672	0.5958	0.5694	0.507
0.0010	0.5352	0.4345	0.4312	0.4789	0.5826	0.6002	0.518
1.0e-4	0.5621	0.4345	0.4936	0.4870	0.5922	0.6310	0.491
1.0e-5	0.4421	0.5778	0.4312	0.4789	0.5892	0.6438	0.470
1.0e-6	0.4421	0.4345	NA	0.4845	0.5683	0.6229	0.434
1.0e-7	0.4421	0.4345	NA	0.4585	0.6006	0.6427	NA
			20RF				
0.15	0.4421	0.4339	0.4301	0.4585	0.4431	0.4300	0.434
0.1	0.4421	0.4369	0.4324	0.4585	0.4431	0.4300	0.434
0.05	0.4421	0.4351	0.4301	0.4585	0.4431	0.4300	0.434
0.01	0.4421	0.4345	0.4312	0.4585	0.4431	0.4300	0.434
0.0010	0.4421	0.4345	0.4312	0.4585	0.4443	0.4364	0.434
1.0e-4	0.4421	0.4498	0.4312	0.4591	0.4832	0.4643	0.435
1.0e-5	0.4582	0.4345	0.5717	0.4777	0.5162	0.4898	0.453
1.0e-6	0.4922	0.4715	NA	0.4845	0.5335	0.4904	0.434
1.0e-7	0.4934	0.4328	NA	0.4963	0.5431	0.5044	NA

Table S89: Overall accuracy obtained by all the algorithms under the haplotype-based approach with 5-SNP haplotype length, recessive genetic model and holdout sampling. Results for each p-value threshold (column 1) are shown. The maximum p-value threshold used was 0.15.
